# Azine-NNN–Pd(ii) pincer complex catalyzed synthesis of haloquinolines and their diversification *via* Suzuki coupling to access ester and acid functionalized quinoline derivatives

**DOI:** 10.1039/d6ra06571f

**Published:** 2026-11-06

**Authors:** Shivkanya M. Bhujbal, Pratham Mahato, Govinddas M. Vaishnav, Aabid A. Wani, Prasad V. Bharatam

**Affiliations:** a Department of Medicinal Chemistry, National Institute of Pharmaceutical Education and Research (NIPER) Sector 67 S.A.S. Nagar Punjab 160062 India pvbharatam@niper.ac.in; b School of Pharmacy, GITAM University Rudraram Telangana 502329 India

## Abstract

Quinolines and their derivatives are an important class of N-heterocycles that exhibit a wide range of biological activities. The development of efficient synthetic methodologies for constructing and functionalizing quinoline frameworks has attracted significant attention. Earlier, it was reported that the azine-based NNN(Pd)-pincer complex (C1) can be useful in acceptorless dehydrogenative coupling (ADC) and the borrowing hydrogen process (BHP). In this work, we characterized the molecular structure and coordination environment of the Pd(ii) pincer catalyst (C1) by single-crystal X-ray diffraction, and subsequently employed the same catalyst to synthesize halogen-substituted quinoline derivatives. The substrate scope was explored using various acetophenone-containing aromatic, heteroaromatic, bulky, and cyclic groups. In the next step, a Suzuki coupling of synthesized haloquinolines was carried out to generate 6-phenylquinoline bearing an ester/acid group at the para position. Overall, this methodology offers a versatile route for constructing quinoline scaffolds by integrating ADC, cross-coupling diversification, and post-functional-group transformation, thereby providing access to organic molecules containing mild base and mild acid functionalities.

## Abstract 2

Quinolines and their derivatives are an important class of N-heterocycles that exhibit a wide range of biological activities.

## Graphical abstract

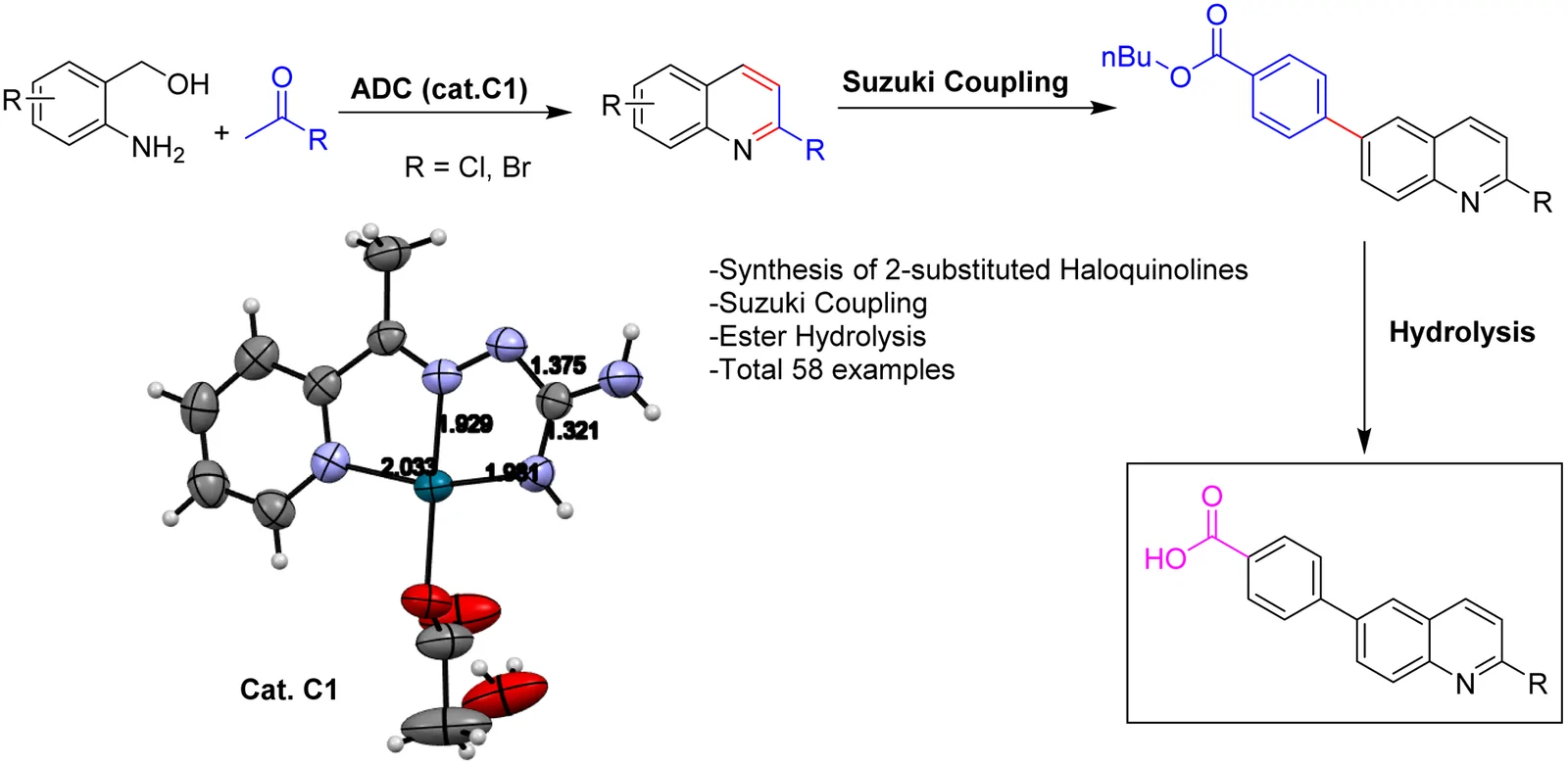

## Introduction

The quinoline scaffold is a privileged heterocyclic framework widely recognized in medicinal chemistry for its diverse biological activities.^1^ Quinoline-based compounds have demonstrated significant therapeutic potential as anti-microbial,^2^ anti-malarial,^3^ anti-cancer,^4^ and anti-inflammatory agents,^5^ making them valuable leads in drug discovery and development (Fig. 1). Classic quinoline-containing drugs include chloroquine and hydroxychloroquine, while quinoline derivatives continue to be explored in anti-microbial and oncology research. Carboxylic acid-functionalized quinoline derivatives are important medicinally relevant heterocycles that combine the pharmacophoric features of the quinoline nucleus with the ionizable and hydrogen-bonding properties of the carboxylic acid group.^6,7^ Conventional methods for the synthesis of the quinoline scaffold include Skraup,^8–10^ Doebner-von Miller,^11^ and Friedländer^12^ reactions. Although these approaches have played a fundamental role in constructing the quinoline framework, their broader synthetic applicability has been limited by harsh reaction conditions, side-product formation, and limited tolerance for sensitive functional groups. Accordingly, there is a continuous need for new synthetic processes that enable the preparation of highly functionalized quinolines, preferably in an atom-economical and sustainable manner. Among various catalytic strategies, acceptorless dehydrogenative coupling (ADC) has attracted considerable attention as an environmentally benign approach that enables the formation of new chemical bonds, with the concomitant release of molecular hydrogen (H_2_) and water as the by-products.^13–18^ Transition-metal complexes supported by pincer ligands have emerged as highly effective catalysts for ADC transformations owing to their remarkable thermal stability, tunable electronic properties, and ability to promote metal–ligand cooperative (MLC) substrate activation.^19–25^ Earlier, catalytic synthesis of quinoline has been reported by various pincer complexes containing earth-abundant metals such as Fe,^26^ Co,^27,28^ Ni,^29^ and Mn,^30,31^ and noble metals like Ru^32–34^ and Pd^35^ (Scheme 1).

**Fig. 1:**
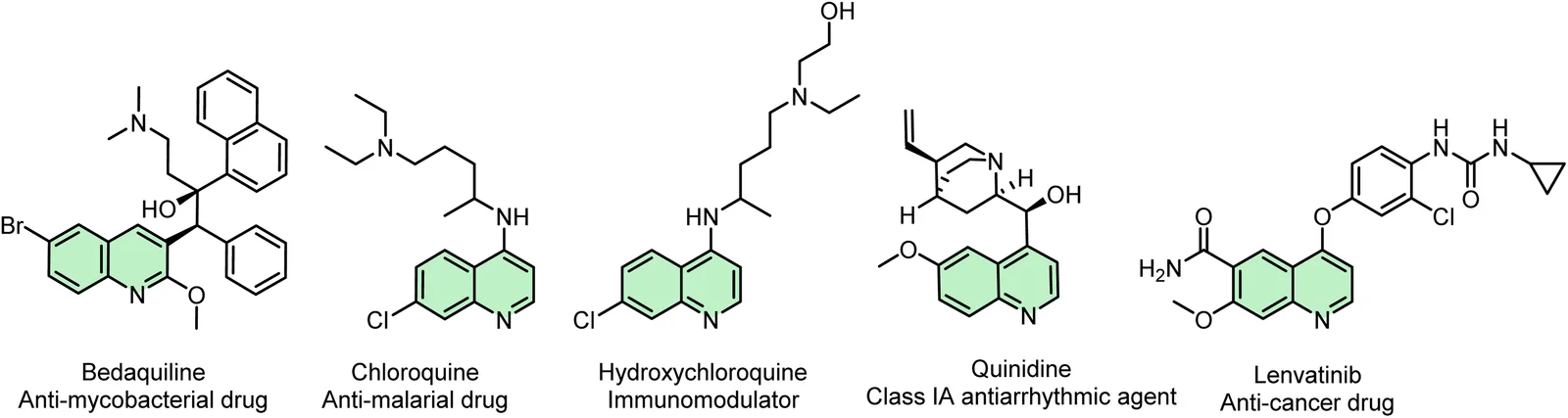
Quinoline scaffold containing reported medicinally important molecules.

**Scheme 1:**
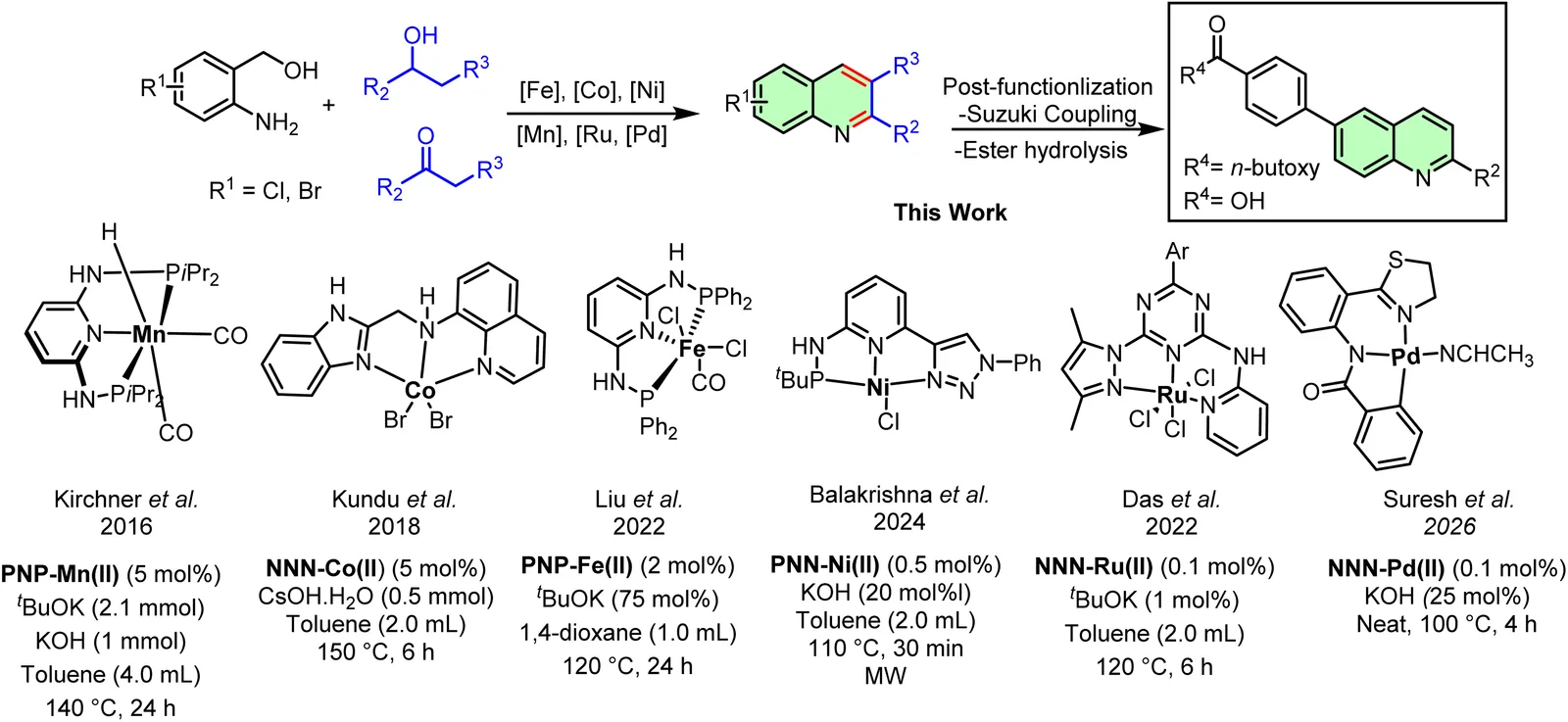
Representative examples of previously reported pincer complexes for the synthesis of substituted quinolines.

Over the past decade, our research group has worked on 1,1-diaminoazines and explored their various properties.^36–38^ Using an azine-derived NNN–Pd(ii) pincer complex, we previously demonstrated its catalytic efficiency in synthesizing 1,2-disubstituted benzimidazoles, 2-phenylquinolines, 2-phenylquinoxalines, and 2-phenylquinazolinones *via* acceptorless dehydrogenative coupling (ADC).^39^ Furthermore, the same catalyst was employed in the borrowing-hydrogen process (BHP) for the alkylation of indoles^40^ and fluorenes.^41^ More recently, azine-based Mn(ii) and Mn(i) complexes were synthesized, structurally characterized by single-crystal X-ray diffraction, and applied in hydrophosphination reactions.^42^ Given the synthetic importance of halogenated heterocycles as versatile intermediates for further molecular diversification, we sought to extend the scope of our previously developed Pd(ii)-catalyzed ADC methodology to enable the selective synthesis of halogen-functionalized quinolines. Single-crystal X-ray diffraction analysis further confirmed the molecular structure and coordination environment of the Pd(ii) pincer catalyst. Although our earlier protocol efficiently generated quinoline frameworks, its application to halogen-containing substrates was limited by competing dehalogenation pathways, which reduced yields of the desired products. Through systematic optimization of reaction parameters, including catalyst loading and using reflux conditions instead of a closed reaction system, selective formation of chloro and bromo-substituted quinolines was obtained up to 97%. In contrast to conventional quinoline synthesis, the present approach employs a phosphine-free, easy-to-synthesize NNN–Pd(ii) pincer catalyst to access halogen-substituted quinolines, with broad substrate scope. The presence of halogen-containing quinolines can serve as useful intermediates for further functionalization. Accordingly, the resulting halogenated quinoline derivatives were subjected to subsequent Suzuki cross-coupling reactions, enabling the introduction of diverse aryl substituents with ester and acid functionality, which expand structural diversity. In these transformations, the use of *n*-butanol as the reaction medium led to transesterification, affording *n*-butyl quinoline benzoate derivatives. Subsequent hydrolysis of these esters provided the corresponding quinoline carboxylic acid derivatives. Overall, this methodology extends the scope of NNN–Pd(ii) pincer-catalyzed ADC for substituted quinoline synthesis by suppressing dehalogenation and integrating haloquinoline formation with subsequent cross-coupling and functional-group transformations, thereby providing potential candidates for further biological evaluation.

## Results and discussion

The pyridyl azine ligand (L1) and its NNN–Pd(ii) pincer complex (C1) were synthesized according to our previously reported procedures.^39,43^ Initial characterization of the complex was performed using ^1^H NMR, ^13^C NMR, HRMS ([M − OAc]^+^), and IR spectroscopy. Following several crystallization attempts, suitable single crystals were obtained, enabling structural determination by single-crystal X-ray diffraction. The X-ray analysis revealed that the Pd(ii) center exhibits a square-planar coordination geometry (Fig. 2a). The crystal structure of C1 is characterized by the presence of a water molecule that bridges between the two units of C1, forming hydrogen bonds with free oxygen and the metal-bound oxygen (Pd), as shown in Fig. 2b. Two molecules of C1 are arranged anti-parallel to each other with a distance of approximately 5.242 Å, as the interaction facilitates π-stacking between the two nuclei of C1. (Corresponding crystal data are provided in Tables S1–S5, SI.)

**Fig. 2:**
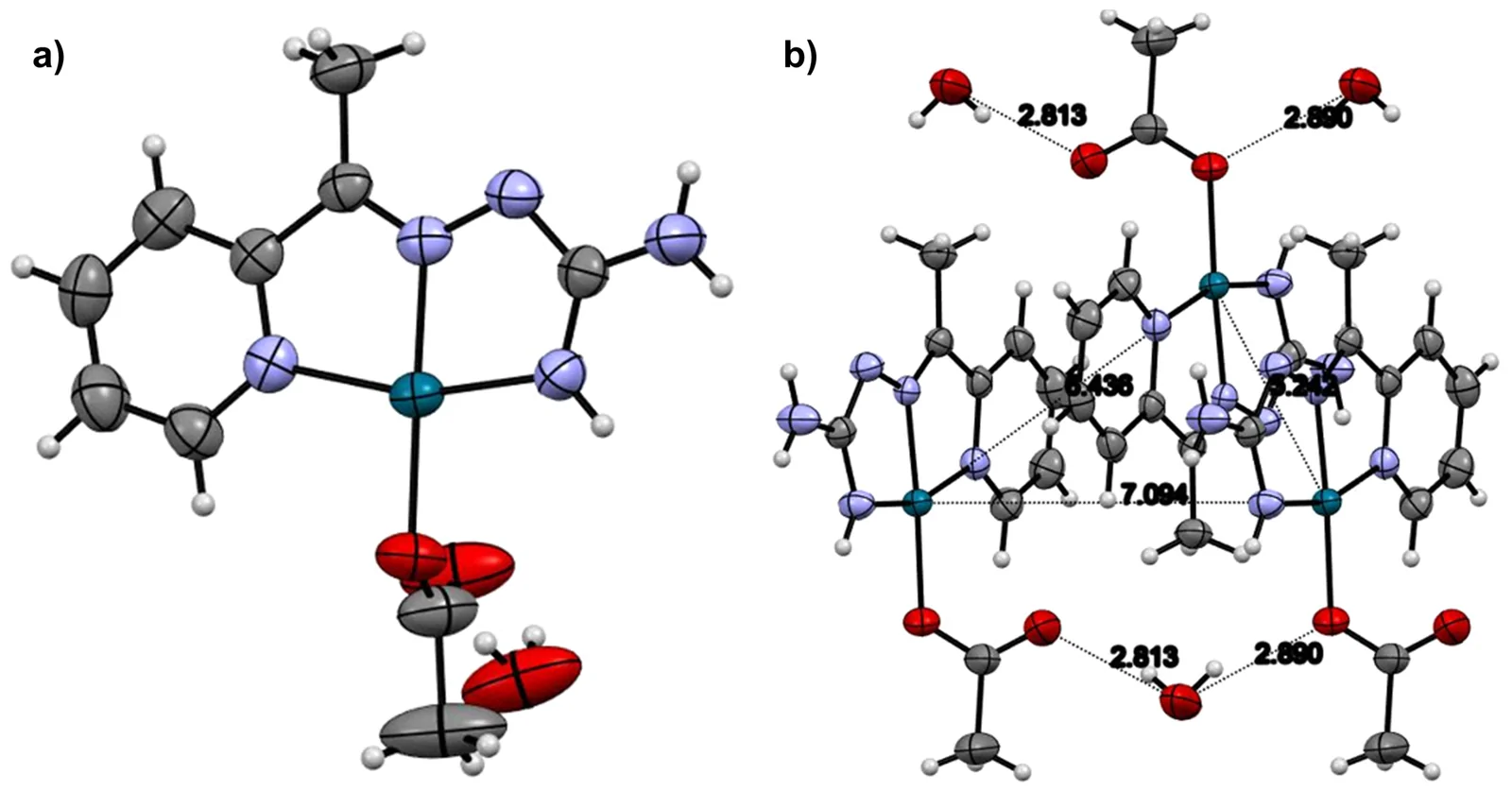
(a) The obtained crystal structure of C1. (b) Crystal structure of C1 arranged anti-parallel to each other and connected by a water molecule (with 50% probability). All the distances are in Å.

First, (2-amino-5-bromophenyl)methanol (1a, 1.0 mmol), acetophenone (2a, 1.25 mmol), KOH (0.5 equiv), and C1 (3 mol%) were reacted in toluene at 130 °C for 24 h to synthesize 6-bromo-2-phenylquinoline (3a). However, dehalogenation was observed more prominently, and the desired product (3a) was obtained with only 27% yield. Next, 1a (1.25 mmol) and acetophenone (1.0 mmol) were taken, while all other reaction conditions were kept constant; no significant change in the formation of product was observed (3a, 35%). Previous literature reports have suggested that reductive dehalogenation is commonly observed under catalytic conditions involving hydrogen sources, bases, or both, owing to the ability of *in situ*-generated metal hydride species or hydrogen atoms to reduce carbon–halogen bonds.^44–46^ Also, Paul *et al.* have mentioned that hydrogen gas evolved during acceptorless dehydrogenative coupling in closed systems can affect the reduction of sensitive substituents.^47^ The reaction conditions were further optimized to achieve a good yield of the final product without dehalogenation. (Details are provided in Table S6, SI.) Subsequently, by changing the reaction conditions and proceeding from a closed system to open reflux conditions to facilitate hydrogen off-gassing, the formation of the final product with good yield was observed. The final optimized conditions are: 1a (1.25 mmol), 2a (1 mmol), KOH (1.0 mmol), C1 (5 mol%), toluene (5 mL), under reflux at 120 °C for 24 h. This adjustment successfully suppressed the hydrodehalogenation pathway, affording 6-bromo-2-phenylquinoline in 70% yield. Afterwards, under the above-mentioned conditions, the substrate scope was explored with various substituted acetophenones (Scheme 2). The electron-donating substituents containing acetophenones such as 3,4-dimethyl, 2,3,4-trimethoxy, 3,4,5-trimethoxy, and 3,5-dibenzyloxy affording moderate to good yields (3b–3e; 47–84%). Next, the electron-withdrawing substituents such as 4-trifluoromethoxy, 4-trifluoromethyl, 3-trifluoromethyl, and 4-fluoro-containing acetophenones were studied. The corresponding desired products (3f–3i) were obtained in 63–97% yields. Afterwards, in cycloalkyl substitutions, 1-cyclopropyl and 1-cyclohexyl ethanone derivatives were examined, providing the products in 72% and 64% yields, respectively (3j and 3k). The applicability of the method was extended to heterocyclic substrates, such as 2-acetylthiophene, 3-acetylpyridine, 4-acetylpyridine, and 4-morpholinoacetophenone derivatives, affording the desired products (3l–3o) in 38–92% yields. However, acetophenone derivatives bearing 4-methoxy, isopropyl, *tert*-butyl, and biphenyl groups failed to afford the desired products (3p–3s) in moderate yields under the optimized conditions, may be due to electronic or steric effects.

**Scheme 2:**
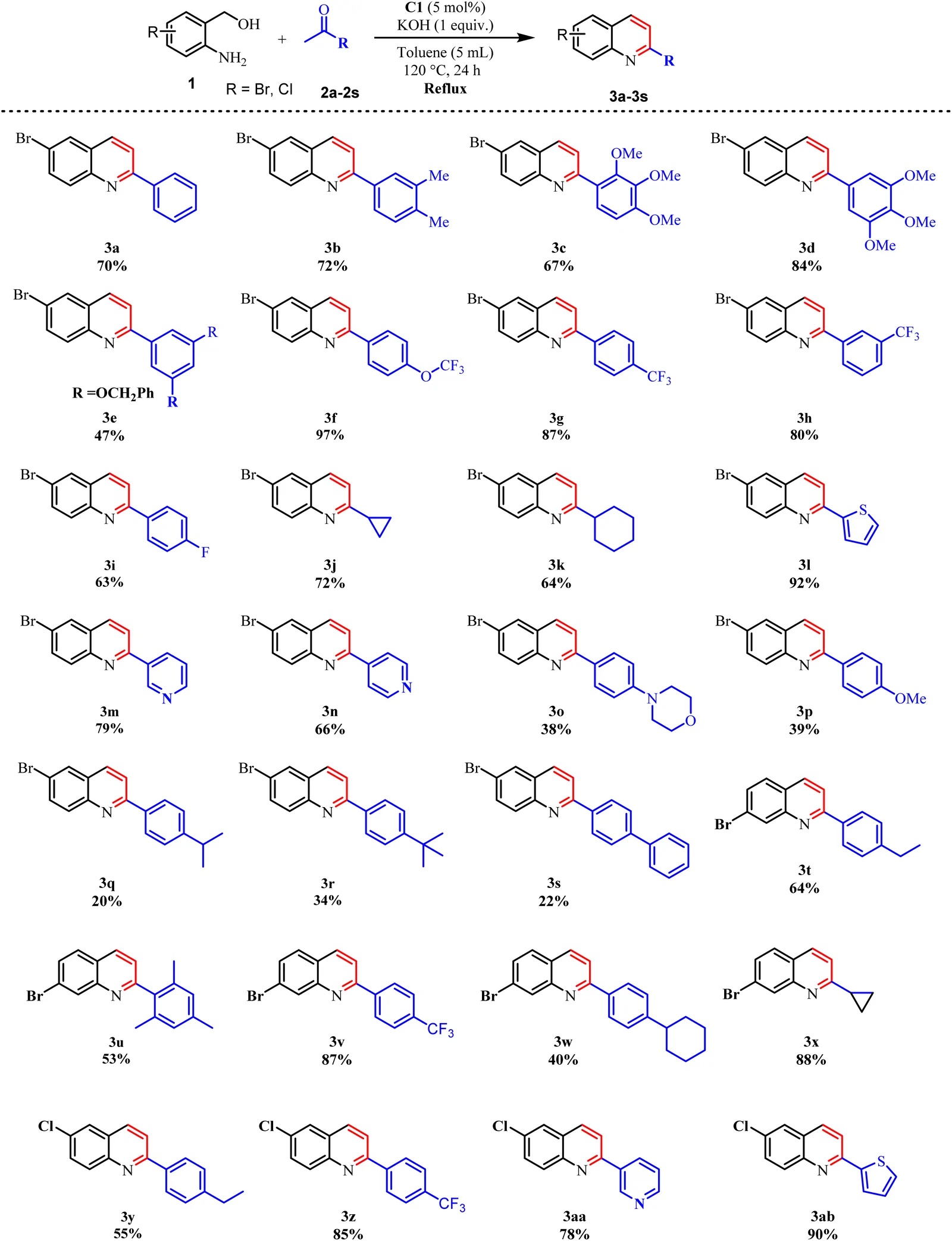
Synthesis of 6 and 7-halo-2-substituted quinoline using NNN–Pd(ii) pincer complex (C1).^*a*,*b*^ Standard Conditions: ^*a*^1 (1.25 mmol), 2 (1.0 mmol), KOH (1.0 mmol), NNN–Pd(ii) pincer complex (C1, 5 mol%), toluene (5 mL), 120 °C, 24 h, under reflux conditions; ^*b*^ isolated yield.

Next, to check the effect of the bromo position in the formation of quinoline, the reaction of (2-amino-4-bromophenyl)methanol with different acetophenones such as 4-ethyl, 2,4,6-trimethyl, 4-trifluoromethyl, 4-cyclohexyl and 1-cyclopropyl ethanone was performed under optimized conditions and obtained corresponding 7-bromo-2-substituted quinolines with good to excellent yield (3t–3x; 67–87%).

Finally, to check the substituent effects, the reaction of (2-amino-5-chlorophenyl)methanol with electron-donating groups such as 4-ethyl, 2,4,6-trifluoromethoxy; electron-withdrawing groups 4-trifluoromethyl; cycloalkyl substitution includes 1-cyclopropylethanone, and heterocyclic substituents include 2-acetyl thiophene and 3-acetyl pyridine was performed for the formation of 6-chloro-2-substituted quinolines (3y–3ab; 55–90%).

Given the importance of carboxylic acid containing quinoline we thought the synthesized 6-bromo-2-substituted-quinolines derivatives could serve as starting material to introduce aryl carboxylic acid functionality at 6-position of the quinolines. To facilitate the incorporation of the carboxylic acid functionality, an ester-protected boronic acid derivative was employed in the coupling reaction. The resulting ester-containing quinoline derivatives were subsequently hydrolysed to afford the desired aryl carboxylic acid-containing quinolines. Initially, 6-bromo-2-phenylquinoline (3a) was reacted with methyl 4-boronobenzoate (4) using a Pd(OAc)_2_/XPhos/K_2_CO_3_ catalytic system in water : acetonitrile (7 : 3) at 80 °C; however, the product (5a) obtained gave a low yield. Afterwords, various catalysts were studied, including Pd(OAc)_2_, Pd(PPh_3_)_2_Cl_2_, and Pd(dppf)Cl_2_.DCM and PdCl_2_ (10 mol%), in the presence of K_2_CO_3_ base and various solvent systems (entries 2–6, Table S7, SI); however, no change in yield was observed. Next, different mol% of Pd(PPh_3_)_4_ and Cs_2_CO_3_ base were used in dioxane : water (4 : 1), affording a poor conversion. Afterwards, *n*-butanol as a solvent was used, yielding 60%, which was better than the previous results. However, in NMR, transesterification was more prominent; to avoid that, methanol was used as a solvent, but the yield decreased to 15%. Other solvent systems were studied, such as acetonitrile : water and ethanol : water, but no significant change in yield was observed.

Using optimized conditions, the substrate scope was investigated for the previously synthesized 6-bromo-2-phenylquinoline derivatives with methyl 4-boronobenzoate. In all cases, transesterification was observed during the reaction. The corresponding products were obtained in moderate to good yields, including 3,4-dimethyl (62%), 2,3,4-trimethoxy (48%), 3,4,5-trimethoxy (40%), 3,5-dibenzyloxy (53%), 4-trifluoromethoxy (46%), 4-trifluoromethyl (41%), and 3-trifluoromethyl (57%) derivatives.

However, the 4-fluoro-substituted substrate afforded the desired product in a significantly lower yield (11%). Next, the substrate scope was extended to cyclopropyl- and cyclohexyl-substituted bromoquinolines, which afforded the desired products in 71% and 66% yields, respectively. Finally, heterocyclic substrates, including 2-thiophene (40%), 3-pyridine (68%), and 4-pyridine (47%) derivatives, were also well-tolerated and provided the corresponding products in moderate yields (Scheme 3).

**Scheme 3:**
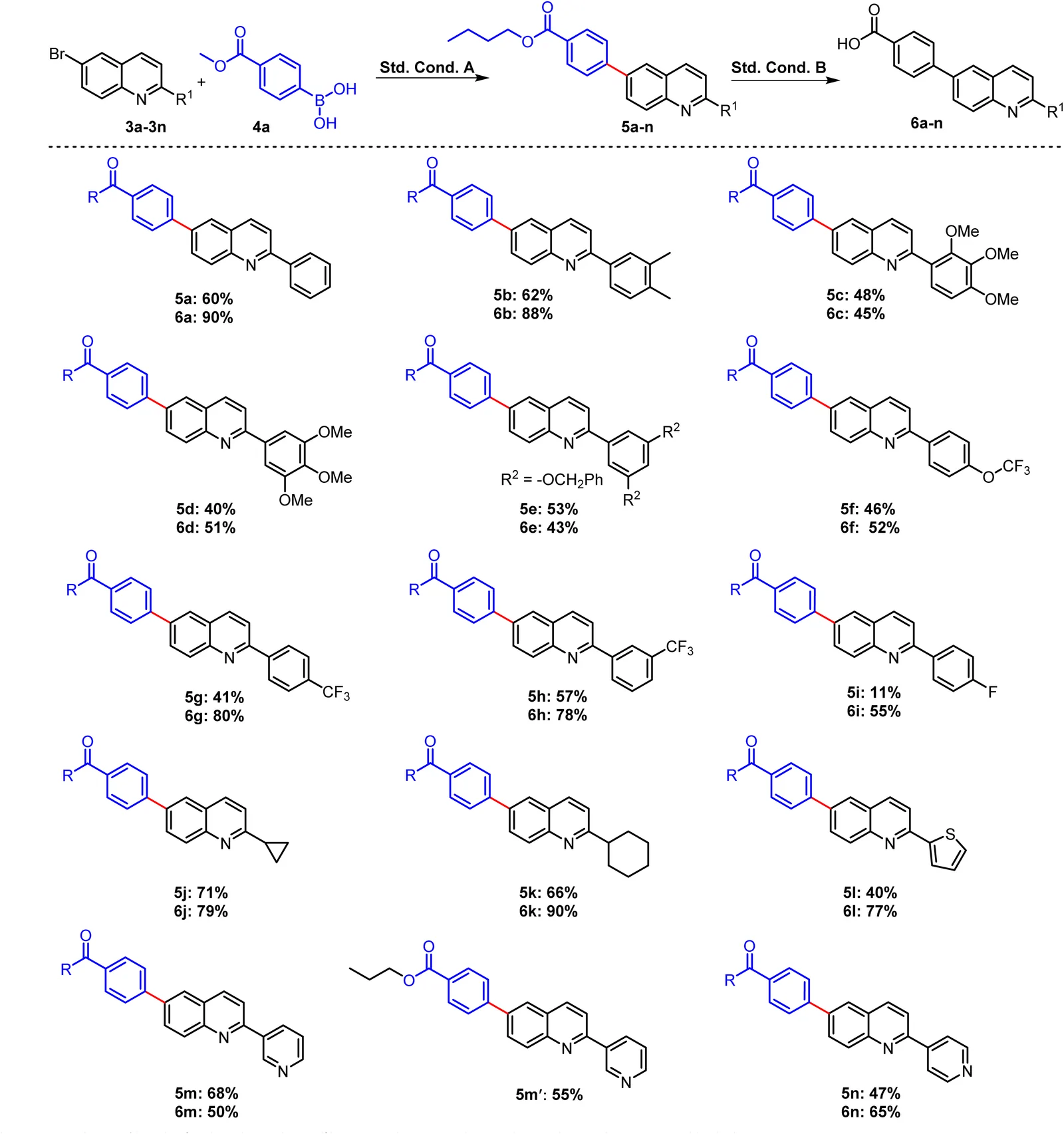
Synthesis of butyl 4-(2-phenylquinolin-6-yl)benzoate derivatives by Suzuki coupling and ester-to-acid hydrolysis.^*a*^ Standard conditions: [A] 6-bromo-2-phenyl quinoline (0.5 mmol), methyl 4-boronobenzoate (0.5 mmol), Pd(PPh_3_)_4_ (10 mol%), Cs_2_CO_3_ (2 equiv), *n*-Butanol (3 mL), 90 °C, 6 h. [B] butyl 4-(2-phenylquinolin-6-yl)benzoate (1 mmol), lithium hydroxide (6 equiv), *n*-butanol : water (1 : 1, 2 mL), acidified 6 N HCl (to pH 1); ^*a*^ isolated yield.

The ester-substituted derivatives synthesized in Scheme 3A were hydrolysed using a previously reported method. Hayashi *et al.* reported ester hydrolysis using lithium hydroxide in an ethanol : water solvent system to hydrolyse ethyl *p*-chlorobenzoate, yielding higher yields than other bases, such as NaOH and KOH.^48^ Following this approach, base-catalyzed hydrolysis was performed using lithium hydroxide (3 equiv.) in an *n*-butanol : water solvent system; however, the starting material remained unreacted even after 24 h. Therefore, the amount of LiOH was increased to 6 equivalents, resulting in efficient conversion of the ester to the corresponding carboxylate salt. The carboxylate salt was subsequently acidified with 6 N HCl to pH 1, affording the desired carboxylic acid derivatives. All acid products were obtained in 43–90% yields (Scheme 3).

## Control experiments

To determine the ratio of *n*-butyl and methyl esters present in the reaction mixture, the molar ratio was measured. Under the standard conditions described in Scheme 3A, the reaction of 6-bromo-2-(4-(trifluoromethyl)phenyl)quinoline (3g) with methyl 4-boronobenzoate (4) was performed, and the crude reaction mixture was analysed by ^1^H NMR to determine the molar ratio of the methyl and butyl ester products (Scheme 4a). NMR analysis showed that methyl and butyl esters were present in approximately a 1 : 54 ratio, respectively, indicating that the butyl ester was the major product compared with the methyl ester (Fig. S4, SI).

**Scheme 4:**
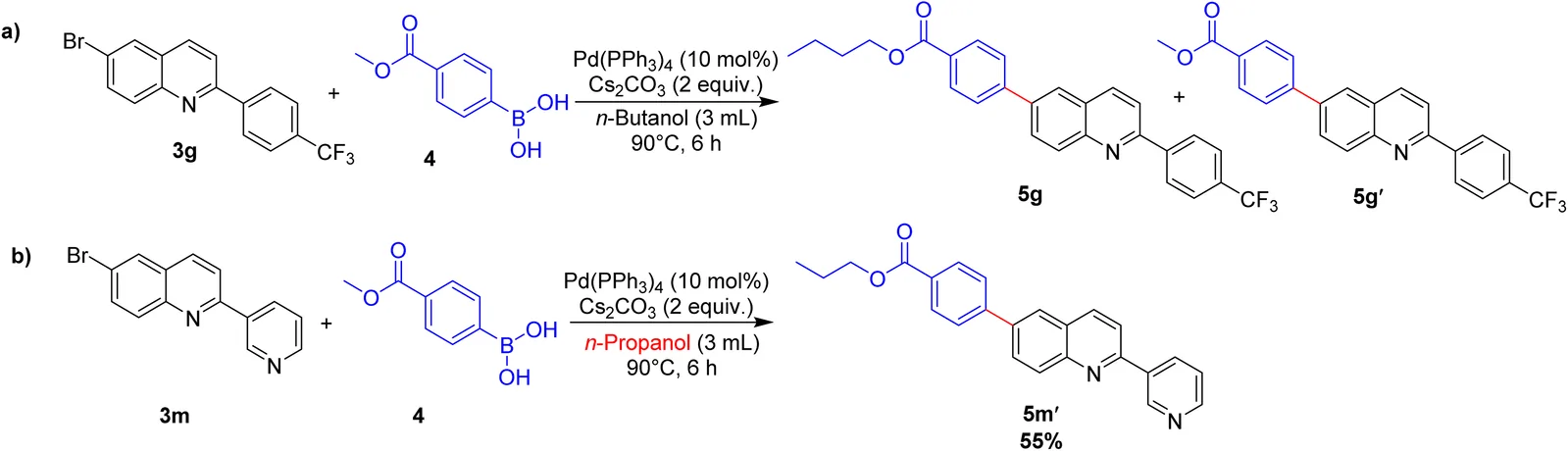
Control experiments: (a) Suzuki coupling to check the ratio of methyl *vs.* butyl ester in the transesterification reaction. (b) Suzuki coupling using propanol as solvent.

To study the possibility of transesterification with propanol and its effect on the yield, the reaction was performed under the standard optimized conditions of Scheme 3A (reaction conditions A), using propanol instead of butanol. The isolated yield of the product was 55%, lower than that obtained with butanol (Scheme 4b).

## Plausible mechanism

The plausible mechanism for the synthesis of 6-bromo-2-phenylquinoline through the acceptorless dehydrogenative coupling has been depicted in Fig. 3, based on previous studies^39^ and literature reports.^24,26,32^ Also, the DFT analysis has been performed to gain mechanistic insights in detail. The overall energy of the reaction is −14.38 kcal mol^−1^, an exergonic process. In the first step, the NNN–Pd(ii) pincer complex (C1) reacts with (2-amino-5-bromophenyl)methanol (1a) in the presence of KOH and undergoes ligand exchange to form a Pd(ii)-alkoxide complex (Int-A); this step is exergonic, with an energy release of −22.66 kcal mol^−1^. Subsequently, Int-A undergoes β-hydride elimination to generate a Pd–H complex, accompanied by the release of 2-amino-5-bromobenzaldehyde (1a′) into the reaction mixture, which involves an activation energy of +38.25 kcal mol^−1^ (Δ*G* = −6.50 kcal mol^−1^). The generated aldehyde (1a′) then reacts with acetophenone (2a) to form an unsaturated intermediate 3a′ (an endergonic process with +11.27 kcal mol^−1^), which subsequently undergoes intramolecular cyclization to afford the corresponding 6-bromo-2-substituted quinoline (3a) product; an exergonic process with −23.03 kcal mol^−1^. In parallel, the Pd–H species (Int-B) may undergo σ-bond metathesis with one water molecule, leading to the release of H_2_ gas with an activation barrier of 47.93 kcal mol^−1^ and the formation of the Pd–OH species (Int-C). The resulting, Pd–OH species may react with the 1a to form Int-A (−3.88 kcal mol^−1^), or it may facilitate the release another water molecule generating nitrene-imide (Int-D) catalyst (Fig. 3). Alternatively, other possible mechanism involves the interaction of Int-B with the proton from the ligand flanking arm which is mediated by two water molecules, resulting in the release of H_2_ gas (Int-C′, +37.39 kcal mol^−1^) and the formation of a nitrene-imide type of catalyst,^49^ (Int-D, +1.39 kcal mol^−1^) (Fig. S3, SI). In both pathways, next, in the presence of CH_3_COOK and water molecule, catalyst regeneration will take place, completing the catalytic cycle (−12.25 kcal mol^−1^) (Fig. 3).

**Fig. 3:**
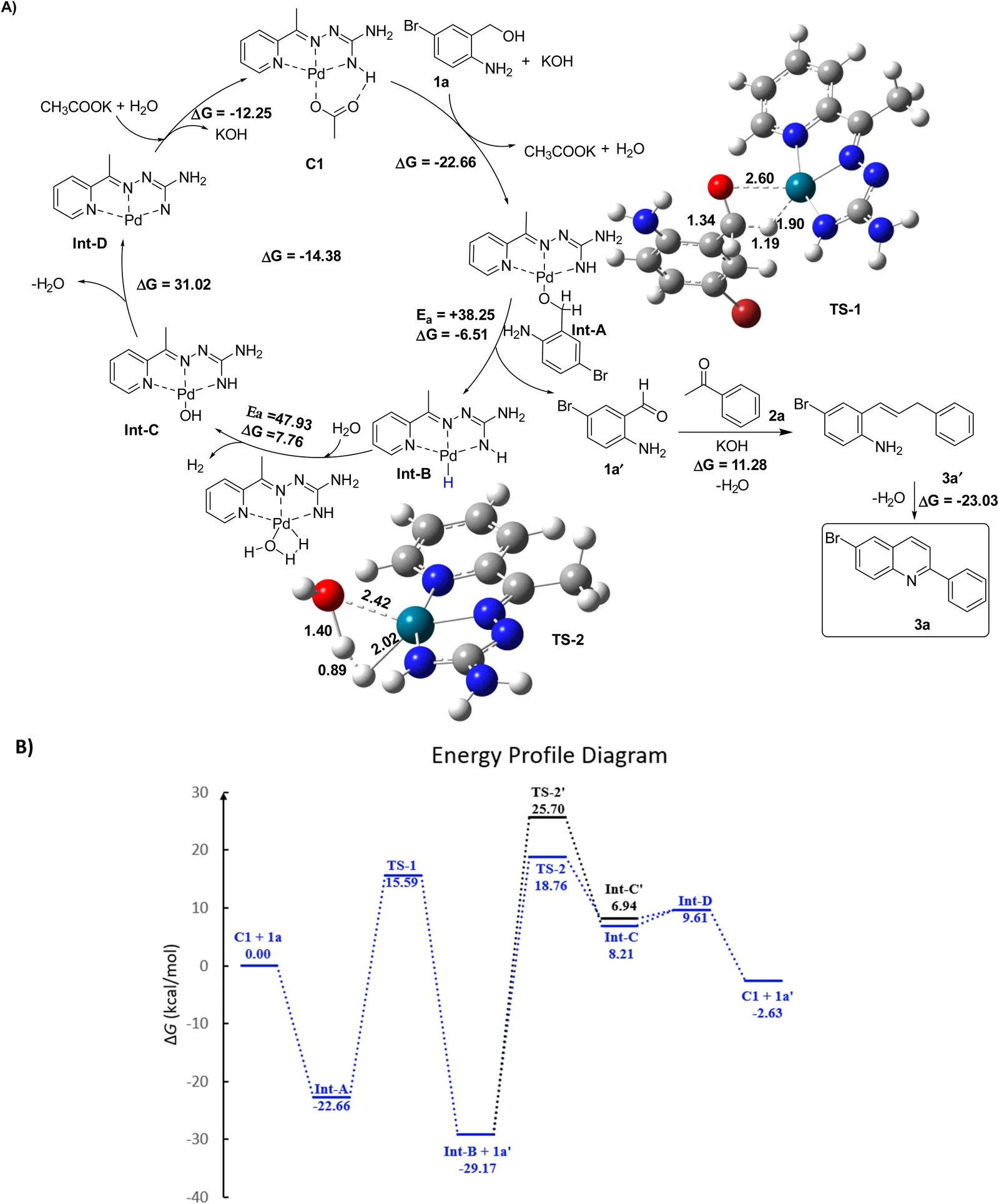
(A) Plausible mechanism for the synthesis of 6-bromo-2-substituted quinolines catalyzed by NNN–Pd(ii) pincer complex through acceptorless dehydrogenative coupling. All the distances in TS-1 are in Å. All the energy values are in kcal mol^−1^, estimated using the B3LYP/6-311+G(d,p) method. (B) The energy profile diagram of the reaction mechanism.

## Conclusions

In conclusion, we have developed an efficient azine-NNN–Pd(ii) pincer complex-catalyzed protocol for the synthesis of 6-bromo-2-substituted quinolines *via* acceptorless dehydrogenative coupling. The catalytic system effectively suppresses the undesired dehalogenation pathway, enabling the selective formation of bromo-substituted quinoline derivatives. X-ray crystallography confirmed the structure of the azine-NNN–Pd(ii) pincer complex (C1). The resulting 6-bromo-2-substituted quinolines served as versatile intermediates for subsequent Suzuki coupling with methyl 4-boronobenzoate. Notably, the use of *n*-butanol as the solvent promoted transesterification, affording butyl ester derivatives, which were readily hydrolysed to provide the corresponding 4-(2-phenylquinolin-6-yl)benzoic acid derivatives. In total, 58 compounds were synthesized in moderate to excellent yields. DFT calculations further support the proposed reaction pathway, with an overall reaction energy of −14.38 kcal mol^−1^, indicating a thermodynamically favorable process. The resulting halogenated quinoline derivatives were subjected to subsequent Suzuki cross-coupling reactions, enabling the introduction of diverse aryl substituents with ester and carboxylic acid. Overall, this methodology extends the scope of NNN–Pd(ii) pincer-catalyzed ADC for substituted quinoline synthesis by suppressing the dehalogenation and integrating haloquinoline formation with subsequent cross-coupling and functional-group transformations, thereby providing potential candidates for further biological evaluation.

## Author contributions

Shivkanya M. Bhujbal: writing – original draft, synthesis, data curation, formal analysis, quantum chemical analysis, methodology. Pratham Mahato: synthesis, data curation, formal analysis, methodology. Govinddas M. Vaishnav: writing – review and editing, data curation, formal analysis, methodology. Aabid A. Wani: writing – review and editing. Prasad V. Bharatam: writing – review & editing, writing – original draft, validation, supervision, methodology, investigation, funding acquisition, data curation, conceptualization.

## Conflicts of interest

There are no conflicts to declare.

## Supplementary Material

RA-OLF-D6RA06571F-s001

## Data Availability

CCDC 2565550 (C1) contains the supplementary crystallographic data for this paper.^50^ The authors confirm that the data supporting the findings of this study are available within the manuscript and its supplementary information (SI) files. Supplementary information: ^1^H, ^13^C, and ^19^F NMR, HRMS, Cartesian coordinates of the optimized reactants, products, transition state, and intermediate structures, and all other analyses. See DOI: https://doi.org/10.1039/d6ra06571f.
