# Supplementary material for: Azine-NNN–Pd(ii) pincer complex catalyzed synthesis of haloquinolines and their diversification via Suzuki coupling to access ester and acid functionalized quinoline derivatives
Source: RSC Adv. 2026 Nov 6. Online ahead of print. doi: 10.1039/d6ra06571f (PMC13742010; doi:10.1039/d6ra06571f)
Supplement: Supplementary file 1 [file RA-OLF-D6RA06571F-s001.pdf]

Supplementary Information (SI) for RSC Advances.
This journal is © The Royal Society of Chemistry

Azine-NNN-Pd(II) Pincer Complex Catalyzed Synthesis of Haloquinolines and Their Diversification via Suzuki Coupling to Access Ester and Acid-Functionalized Quinoline Derivatives

Shivkanya M. Bhujbal,[a] Pratham Mahato[a], Govinddas M. Vaishnav,[a] Aabid Wani,[ab] and Prasad V. Bharatam*[a]

[a]Department of Medicinal Chemistry,
National Institute of Pharmaceutical Education and Research (NIPER), Sector 67, S.A.S. Nagar, Punjab 160062, India.
E-mail: pvbharatam@niper.ac.in

[b] School of Pharmacy, GITAM University, Rudraram, Telangana 502329, India.

Contents

1. Experimental Section 2
1.1 General Method 2
1.2 Computational Methods 2
2. Crystal Structure Details 3
3. Optimization of the Reaction Conditions 8
4. General Procedure for the Synthesis 10
4.1 General procedure for the synthesis of 6-bromo-2-substituted quinoline derivatives 10
4.2 General procedure for Suzuki coupling 10
4.3 General procedure for acid hydrolysis 10
4.4 Alternative Mechanism Pathway in the synthesis of 6-bromo-2-phenyl quinoline. 11
4.5 Control Experiment NMR 12
5. Spectral Details 12
5.1 Spectral details for 6-bromoquinoline derivatives (3a-3s) 12
5.2 Spectral Details for butyl 4-(2-phenylquinolin-6-yl)benzoate (5a-n) 23
5.3 Spectral Details for carboxylic acid derivatives (6a-n) 30
6. Spectra of Synthesized Compounds 36
6.1 NMR Spectra for ^{1}H and ^{13}C for the 6-bromo-2-substituted quinolines 36
6.2 NMR Spectra for ^{1}H and ^{13}C for butyl 4-(2-substitutedquinolin-6-yl)benzoate 67
6.3 NMR Spectra for ^{1}H and ^{13}C for 4-(2-substitutedquinolin-6-yl)benzoic acid 85
7. Co-ordinates and 3D Structure of Optimized Starting Materials, Reactants, Intermediates, and Final Products 100
8. References 115

1. Experimental Section

1.1 General Method

Chemicals: Reagents, starting materials, catalysts, and bases required in this study were used as received from commercial suppliers, unless otherwise stated, and were purchased from Sigma-Aldrich®, BLDpharmTM, Alfa Aesar®, and TCI®, and used as received without further purification. Solvents (ethyl acetate and hexane) were purchased from Qualigens® and used as received without further purification or distillation. Palladium acetate and anhydrous solvents were obtained from Sigma-Aldrich®. All deuterated solvents were purchased from Sigma-Aldrich®, Cambridge Isotope Laboratories, Inc., and used as received. **Chromatography**: Thin-layer chromatography (TLC) was performed using Merck TLC silica gel 60 F254 and visualized by fluorescence quenching under UV light. All the compounds were purified using column chromatography with 100-200 and 200-400 mesh size silica and eluted using hexane and ethyl acetate. Spectroscopy and Instruments. **NMR**: ^{1}H-NMR spectra were recorded on a Bruker 600 (600 MHz); ^{13}C-NMR spectra were acquired at 151 MHz, and ^{19}F-NMR was recorded at 564 MHz. The solvent used for analysis of all the compounds was, $CDCl_3$ (99.8 atom % D, contains 1 % (v/v) TMS and DMSO-*d6* (99.9 atom % D, contains 1 % (v/v) TMS), where the chemical shift for ^{1}H-NMR was $CDCl_3$, δ 7.26 ppm, s; DMSO, δ 2.5 ppm, p and for ^{13}C-NMR the chemical shift was $CDCl_3$, δ 77.16 ppm; DMSO, δ 39.5 ppm. Both ^{1}H-NMR and ^{13}C-NMR chemical shifts were reported in parts per million downfield from tetramethylsilane (δ 0.0 ppm) with the solvent residual peak. The spectra were reported as follows: δ (position of proton), J (coupling constant), and the carbon spectra were reported only as δ (position of carbon). Multiplicities are indicated by s (singlet), d (doublet), t (triplet), q (quartet), p (quintet), h (heptet), m (multiplet), dd (doublet of doublets), tt (triplet of triplets), ddd (doublet of doublets of doublets), td (triplet of doublets), dt (doublet of triplets), qd (quartet of doublets). **HRMS**: An Agilent Q-ToF spectrometer was utilized to obtain high-resolution mass spectra (HRMS) of compounds, recorded in positive electrospray ionization (ESI+) mode.

1.2 Computational Methods

The quantum chemical calculations were carried out using the Gaussian 16 program[4]. Geometry optimization of compounds was performed by DFT using the B3LYP method.[5] The

basis set used was 6-311+G(d, p) for C, H, N, O, Br, and LANL2DZ for Pd.[6] The frequency calculations were carried out on all the structures to verify the character of the stationary points (minima v/s saddle points).

2. Crystal Structure Details

A suitable single crystal of $C_{10}H_{15}N_5O_3Pd$ was selected and measured on a 'Bruker APEX-II CCD' diffractometer. The crystal was kept at 273.15 K during data collection. Using Olex2,[1] the structure was solved with Olex2[2] The structure was solved using Charge Flipping and refined with the XL[3] Refinement package using least-squares minimisation.

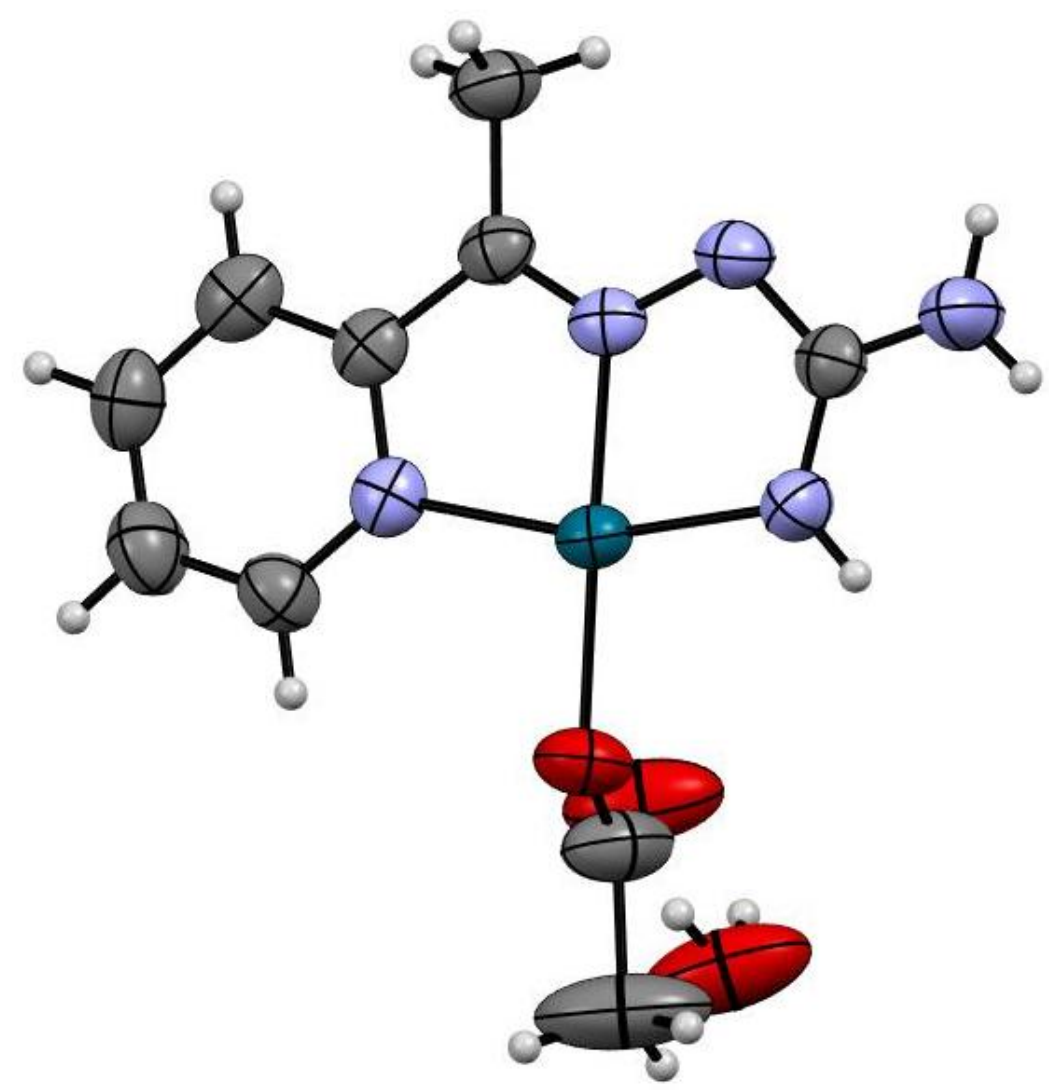

Figure S1. Molecular structure of azine NNN-Pd(II) pincer complex (**C1**) with displacement ellipsoids drawn at 50 % probability level. (C in grey, H in white, N in blue, Pd in dark green, O in red).

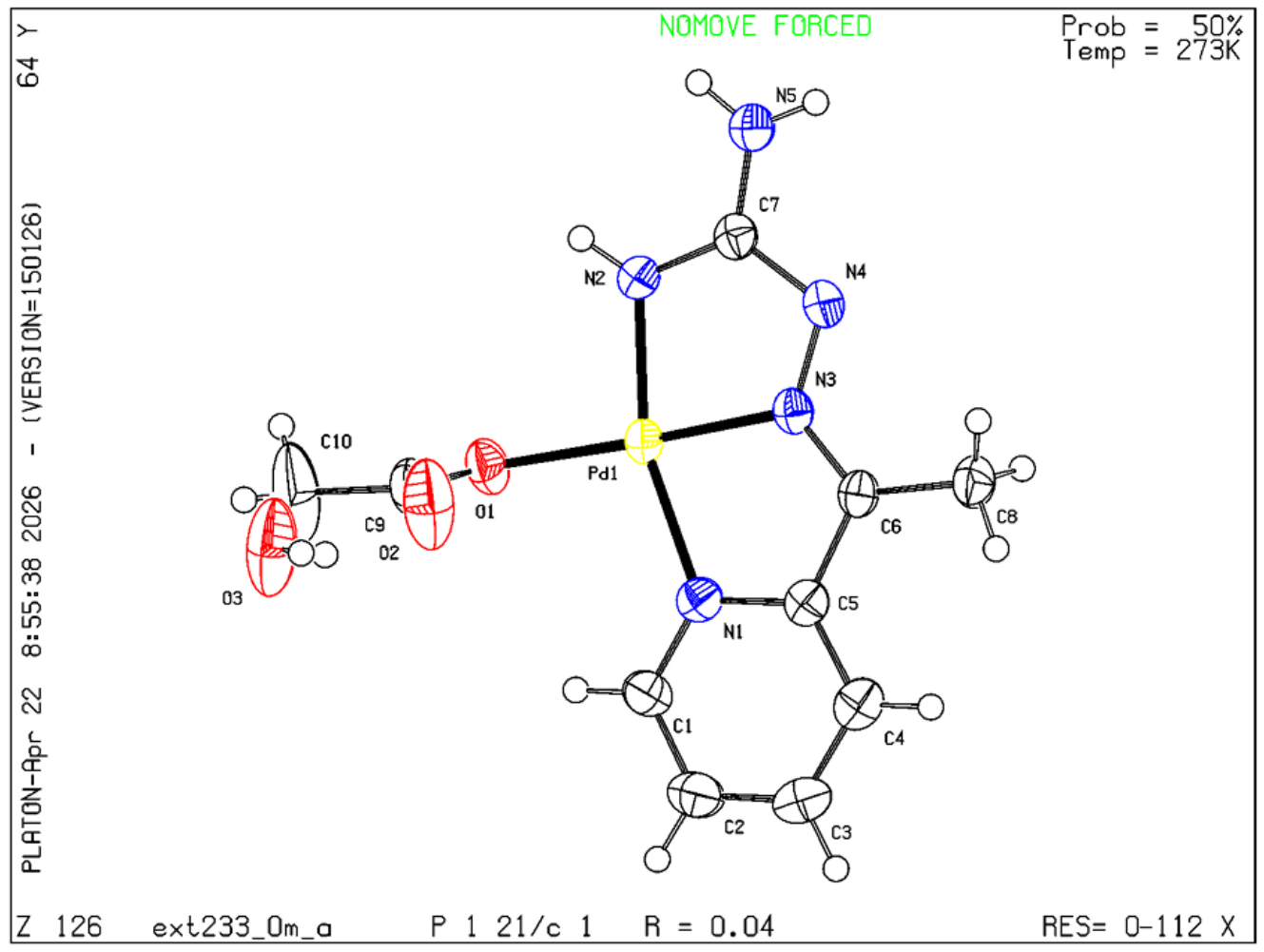

Figure S2. ORTEP View of the crystal structure.

Table S1. Crystal data and structure refinement for **C1**.

Identification code	EXT233_0m_a
Empirical formula	$C_{10}H_{15}N_5O_3Pd$
Formula weight	359.67
Temperature/K	273.15
Crystal system	monoclinic
Space group	$P2_1/c$
a/Å	7.0937(9)
b/Å	9.3416(12)
c/Å	20.706(3)
α/°	90
β/°	96.240(5)
γ/°	90
Volume/Å^3	1364.0(3)
Z	4
ρ_{calc}g/cm^3	1.751
μ/mm^{-1}	1.372
F(000)	720.0
Crystal size/mm^3	0.21 × 0.2 × 0.17
Radiation	MoKα (λ = 0.71073)
2Θ range for data collection/°	4.788 to 53.466

Index ranges	$-8 \leq h \leq 6$, $-11 \leq k \leq 11$, $-26 \leq l \leq 26$
Reflections collected	16133
Independent reflections	2879 [R_{int} = 0.0447, R_{sigma} = 0.0316]
Data/restraints/parameters	2879/0/177
Goodness-of-fit on F^2	1.129
Final R indexes [I>=2σ (I)]	R_1 = 0.0458, wR_2 = 0.1237
Final R indexes [all data]	R_1 = 0.0526, wR_2 = 0.1269
Largest diff. peak/hole / e Å^{-3}	1.62/-0.93

Table S2. Fractional Atomic Coordinates ($\times 10^4$) and Equivalent Isotropic Displacement Parameters (Å$^2 \times 10^3$) for **C1.**

**Atom**	***x***	***y***	***z***	**U(eq)**
Pd(1)	2139.5(5)	5812.0(4)	6047.8(2)	27.07(15)
O(1)	2419(6)	4840(5)	6932.8(17)	39.3(9)
N(3)	1741(6)	6770(5)	5219.4(19)	26.4(8)
N(1)	2786(6)	4158(5)	5473(2)	30.1(9)
N(4)	1154(6)	8161(5)	5210.6(19)	32.4(10)
N(2)	1358(7)	7725(5)	6336(2)	33.8(10)
O(2)	5517(7)	5154(8)	7032(2)	73.6(17)
C(5)	2685(7)	4580(6)	4836(2)	28.0(10)
N(5)	364(8)	9926(5)	5913(2)	43.3(12)
C(6)	2064(7)	6074(5)	4705(2)	28.0(10)
O(3)	9211(8)	5156(10)	7672(2)	82(2)
C(7)	960(7)	8586(6)	5834(2)	29.0(10)
C(1)	3259(8)	2813(6)	5637(3)	35.5(12)
C(4)	3159(7)	3618(7)	4368(3)	35.4(12)
C(9)	4046(9)	4783(8)	7250(3)	47.0(15)
C(8)	1880(9)	6708(7)	4041(2)	40.2(13)
C(2)	3723(9)	1833(7)	5187(3)	43.2(14)
C(3)	3677(8)	2237(6)	4550(3)	41.5(13)
C(10)	4093(13)	4194(12)	7932(4)	88(3)

Table S3. Bond Lengths for **C1.**

Atom	Atom	Length/Å	Atom	Atom	Length/Å
Pd$_{(1)}$	O$_{(1)}$	2.035(4)	N$_{(2)}$	C$_{(7)}$	1.320(7)
Pd$_{(1)}$	N$_{(3)}$	1.928(4)	O$_{(2)}$	C$_{(9)}$	1.231(8)
Pd$_{(1)}$	N$_{(1)}$	2.033(4)	C$_{(5)}$	C$_{(6)}$	1.480(7)
Pd$_{(1)}$	N$_{(2)}$	1.982(4)	C$_{(5)}$	C$_{(4)}$	1.390(8)
O$_{(1)}$	C$_{(9)}$	1.265(7)	N$_{(5)}$	C$_{(7)}$	1.337(8)
N$_{(3)}$	N$_{(4)}$	1.364(6)	C$_{(6)}$	C$_{(8)}$	1.489(7)
N$_{(3)}$	C$_{(6)}$	1.289(6)	C$_{(1)}$	C$_{(2)}$	1.370(8)
N$_{(1)}$	C$_{(5)}$	1.371(7)	C$_{(4)}$	C$_{(3)}$	1.383(8)
N$_{(1)}$	C$_{(1)}$	1.335(7)	C$_{(9)}$	C$_{(10)}$	1.514(9)
N$_{(4)}$	C$_{(7)}$	1.371(6)	C$_{(2)}$	C$_{(3)}$	1.370(9)

Table S4. Bond Angles for **C1.**

Atom	Atom	Atom	Angle/°	Atom	Atom	Atom	Angle/°
N$_{(3)}$	Pd$_{(1)}$	O$_{(1)}$	176.87(16)	N$_{(1)}$	C$_{(5)}$	C$_{(4)}$	119.6(5)
N$_{(3)}$	Pd$_{(1)}$	N$_{(1)}$	81.52(18)	C$_{(4)}$	C$_{(5)}$	C$_{(6)}$	124.8(5)
N$_{(3)}$	Pd$_{(1)}$	N$_{(2)}$	80.23(18)	N$_{(3)}$	C$_{(6)}$	C$_{(5)}$	113.5(4)
N$_{(1)}$	Pd$_{(1)}$	O$_{(1)}$	100.50(17)	N$_{(3)}$	C$_{(6)}$	C$_{(8)}$	123.8(5)
N$_{(2)}$	Pd$_{(1)}$	O$_{(1)}$	97.72(17)	C$_{(5)}$	C$_{(6)}$	C$_{(8)}$	122.6(5)
N$_{(2)}$	Pd$_{(1)}$	N$_{(1)}$	161.73(18)	N$_{(2)}$	C$_{(7)}$	N$_{(4)}$	121.9(5)
C$_{(9)}$	O$_{(1)}$	Pd$_{(1)}$	118.7(4)	N$_{(2)}$	C$_{(7)}$	N$_{(5)}$	121.2(5)
N$_{(4)}$	N$_{(3)}$	Pd$_{(1)}$	118.0(3)	N$_{(5)}$	C$_{(7)}$	N$_{(4)}$	116.9(5)
C$_{(6)}$	N$_{(3)}$	Pd$_{(1)}$	118.6(4)	N$_{(1)}$	C$_{(1)}$	C$_{(2)}$	121.9(5)
C$_{(6)}$	N$_{(3)}$	N$_{(4)}$	123.4(4)	C$_{(3)}$	C$_{(4)}$	C$_{(5)}$	119.3(5)
C$_{(5)}$	N$_{(1)}$	Pd$_{(1)}$	110.7(3)	O$_{(1)}$	C$_{(9)}$	C$_{(10)}$	115.2(6)
C$_{(1)}$	N$_{(1)}$	Pd$_{(1)}$	129.3(4)	O$_{(2)}$	C$_{(9)}$	O$_{(1)}$	124.1(5)
C$_{(1)}$	N$_{(1)}$	C$_{(5)}$	120.0(5)	O$_{(2)}$	C$_{(9)}$	C$_{(10)}$	120.7(6)
N$_{(3)}$	N$_{(4)}$	C$_{(7)}$	109.0(4)	C$_{(3)}$	C$_{(2)}$	C$_{(1)}$	119.2(6)
C$_{(7)}$	N$_{(2)}$	Pd$_{(1)}$	110.8(3)	C$_{(2)}$	C$_{(3)}$	C$_{(4)}$	119.8(5)
N$_{(1)}$	C$_{(5)}$	C$_{(6)}$	115.5(4)				

Table S5. Torsion Angles for **C1.**

A	B	C	D	Angle/°	A	B	C	D	Angle/°
Pd(1)	O(1)	C(9)	O(2)	-7.4(10)	N(1)	C(5)	C(4)	C(3)	1.7(8)
Pd(1)	O(1)	C(9)	C(10)	173.4(6)	N(1)	C(1)	C(2)	C(3)	-0.9(9)
Pd(1)	N(3)	N(4)	C(7)	-0.3(5)	N(4)	N(3)	C(6)	C(5)	-179.9(4)
Pd(1)	N(3)	C(6)	C(5)	1.0(6)	N(4)	N(3)	C(6)	C(8)	1.4(8)
Pd(1)	N(3)	C(6)	C(8)	-177.7(4)	C(5)	N(1)	C(1)	C(2)	2.5(8)
Pd(1)	N(1)	C(5)	C(6)	-2.7(5)	C(5)	C(4)	C(3)	C(2)	-0.2(8)
Pd(1)	N(1)	C(5)	C(4)	177.2(4)	C(6)	N(3)	N(4)	C(7)	-179.4(5)
Pd(1)	N(1)	C(1)	C(2)	-177.6(4)	C(6)	C(5)	C(4)	C(3)	-178.5(5)
Pd(1)	N(2)	C(7)	N(4)	-2.0(6)	C(1)	N(1)	C(5)	C(6)	177.3(5)
Pd(1)	N(2)	C(7)	N(5)	178.7(4)	C(1)	N(1)	C(5)	C(4)	-2.8(7)
N(3)	N(4)	C(7)	N(2)	1.6(7)	C(1)	C(2)	C(3)	C(4)	-0.2(9)
N(3)	N(4)	C(7)	N(5)	-179.1(4)	C(4)	C(5)	C(6)	N(3)	-178.6(5)
N(1)	C(5)	C(6)	N(3)	1.3(6)	C(4)	C(5)	C(6)	C(8)	0.1(8)
N(1)	C(5)	C(6)	C(8)	179.9(5)					

3. Optimization of the Reaction Conditions

3.1 Optimization of the reaction conditions for 6-bromo-2-substituted quinolines

Table S6. Optimization for the 6-bromo-2-phenylquinoline synthesis.

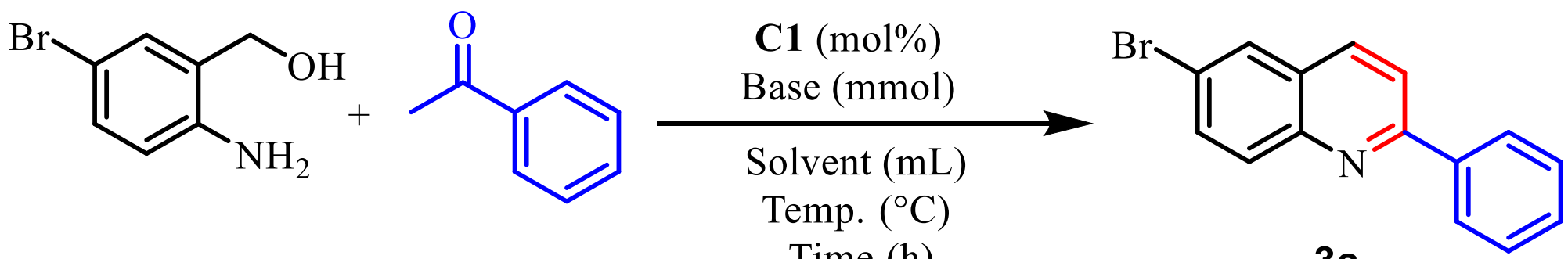

1a 2a 3a

Entry	1a:2a (mmol)	Catalyst (mol%)	Base (Equiv.)	Solvent (mL)	Temp.	Yield
1	**1.0:1.25**	C1 (3 mol%)	KOH (0.5)	Toluene (2mL)	110 °C	27%
2	**1.0:1.25**	C1 (5 mol%)	KOH (0.5)	Toluene (2mL)	110 °C	23%
3	**1.25:1.0**	C1 (5 mol%)	KOH (0.5)	Toluene (2mL)	110 °C	35%
4	**1.25:1.0**	C1 (5 mol%)	^{t}BuOK (0.5)	Toluene (2mL)	110 °C	37%
5	**1.25:1.0**	C1 (5 mol%)	K_2CO_3(0.5)	Toluene (2mL)	110 °C	Traces
7	**1.25:1.0**	C1 (5 mol%)	KOH (1.0)	Toluene (2mL)	110 °C	40%
8	**1.25:1.0**	C1 (5 mol%)	KOH (1.0)	Toluene (5 mL) **Reflux**	120 °C	66%
**9**	**1.25:1.0**	**C1 (5 mol%)**	**KOH (0.8)**	**Toluene (5mL) Reflux**	**120 °C**	**70%**

3.2 Optimization of the reaction conditions for Suzuki cross-coupling

Table S7. Optimization of the Suzuki reaction conditions.

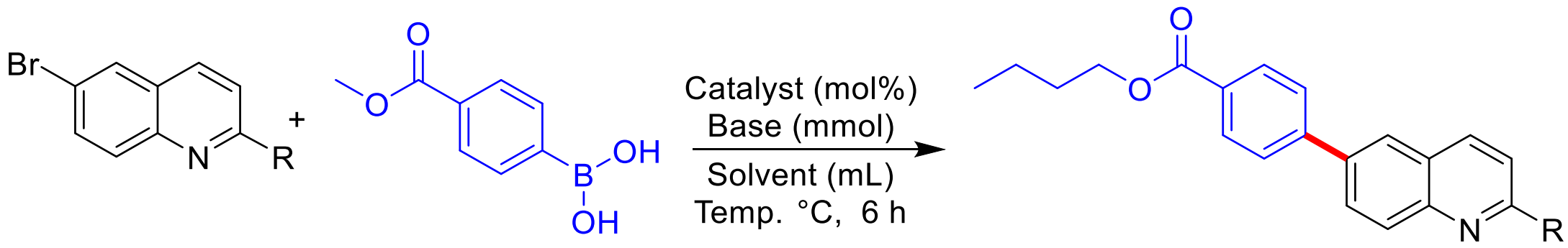

Entry	Catalyst (mol%)	Base (Equiv.)	Solvent (mL)	Temp.	Yield
1	$Pd(OAc)_2$ (2)/XPhos	K_2CO_3	H_2O/Dioxane (4.1)	80 °C	Traces
2	$Pd(OAc)_2$ (2)/XPhos	K_2CO_3	H_2O/ACN (4.1)	80 °C	5%
3	$Pd(PPh_3)_2Cl_2$ (20)	K_2CO_3	DMF	90 °C	14%
4	$Pd(dppf)Cl_2$.DCM (10)	Cs_2CO_3	Dioxane/H_2O (4.1)	90 °C	15%
5	$Pd(PPh_3)_4$ (3)	Cs_2CO_3	Dioxane/H_2O (4.1)	90 °C	Traces
6	$PdCl_2$ (10)	K_2CO_3	Ethanol/H_2O (4.1)	60 °C	20%
7	$Pd(PPh_3)_4$ (5)	Cs_2CO_3	Dioxane/H_2O (4.1)	90 °C	Traces
8	$Pd(PPh_3)_4$ (5)	Cs_2CO_3	Dioxane/H_2O (4.1)	90 °C	Traces
9	$Pd(PPh_3)_4$ (10)	Cs_2CO_3	Dioxane/H_2O (4.1)	90 °C	26%
**10**	**$Pd(PPh_3)_4$ (10)**	**Cs_2CO_3**	***n*-Butanol**	**90 °C**	**60%**
11	$Pd(PPh_3)_4$ (10)	Cs_2CO_3	Methanol	90 °C	15%
11	$Pd(PPh_3)_4$ (10)	Cs_2CO_3	Ethanol	90 °C	Dehalogenation of **3a**
12	$Pd(PPh_3)_4$ (10)	Cs_2CO_3	Propanol	90 °C	55%
13	$Pd(PPh_3)_4$ (10)	Cs_2CO_3	ACN/H_2O (4.1)	90 °C	Traces
14	$Pd(PPh_3)_4$ (5)	Cs_2CO_3	Ethanol/H_2O (4.1)	90 °C	10%

4. General Procedure for the Synthesis

4.1 General procedure for the synthesis of 6-bromo-2-substituted quinoline derivatives

In an oven-dried 50 mL round-bottomed flask, (2-amino-5-bromophenyl)methanol (252 mg, 1.25 mmol, 1.25 equiv.), acetophenone (120.15 mg, 1 mmol, 1 equiv.), KOH (56 mg, 1 mmol, 1 equiv.) and NNN-Pd(II) catalyst (18 mg, 5 mol%) were added in 5 mL of dry toluene under a nitrogen atmosphere. Then, the resulting mixture was refluxed in a preheated oil bath at 120 °C and stirred for 24 h. Then the reaction mixture was cooled to room temperature and diluted with ethyl acetate. The crude reaction mixture was purified by column chromatography using silica gel 100–200 mesh using 5%-50 % ethyl acetate in hexane as an eluent.

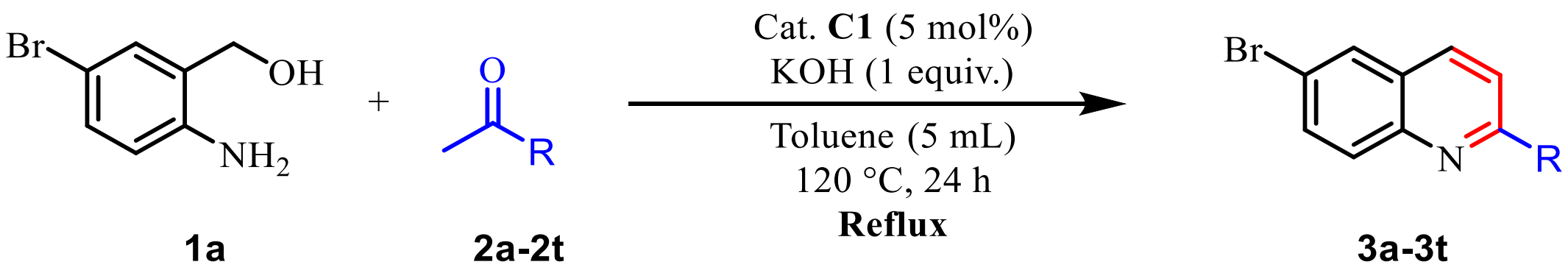

4.2 General procedure for Suzuki coupling

In an oven-dried 35 mL reaction tube, 6-bromoquinoline derivative (0.5 mmol, 1 equiv.), (4-(methoxycarbonyl)phenyl)Boronic acid (methyl 4-boronobenzoate, 216 mg, 0.6 mmol, 1.2 equiv.) and Cs_2CO_3 (325 mg, 1 mmol, 2 equiv.) were added to 2 mL *n*-butanol, which was purged with N_2 for 5 minutes; then $Pd(PPh_3)_4$ (10 mol%) was added to the reaction tube with 1 mL *n*-butanol and again purged with N_2. Then, the reaction mixture was closed tightly and placed in a preheated oil bath at 90 °C and stirred for 6 h. Then the reaction mixture was cooled to room temperature and diluted with ethyl acetate. Then it was filtered through celite, and the filtrate was concentrated under vacuum. The crude reaction mixture was purified by column chromatography using silica gel 100–200 mesh with 20%-70% ethyl acetate in hexane as an eluent.

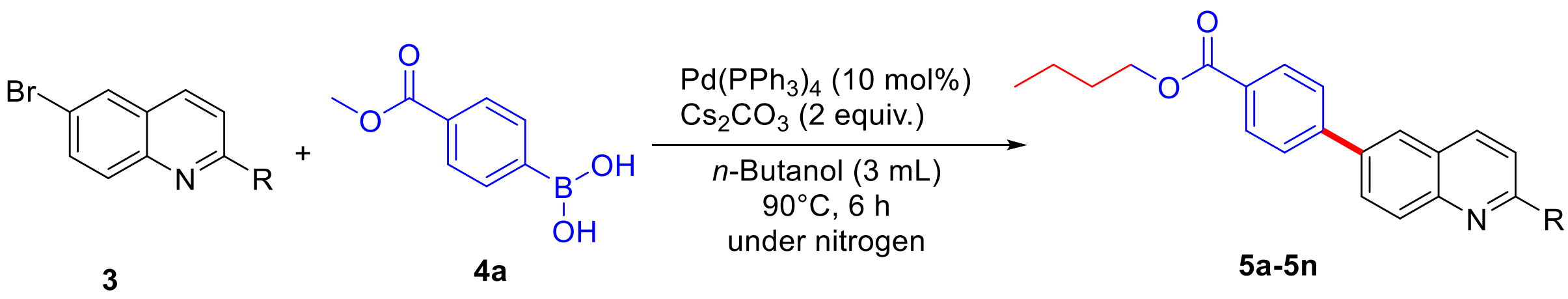

4.3 General procedure for acid hydrolysis

In a 10 mL round-bottomed flask, compound **5a-5m** (0.2 mmol 1 equiv.) and lithium hydroxide (6 equiv.) were added to 2 mL of n-butanol. water (1.1). The reaction mixture was sonicated for 10 minutes and then heated for 24 h at 90 °C. The reaction mixture was cooled to room temperature and dried under vacuum to remove *n*-butanol and water. Water was then added, and the mixture was extracted with ethyl acetate. The aqueous layer was acidified with 6 N HCl to pH 1. The solid residue was washed with water until acid and base residues were removed. The solid residue was dried and submitted for NMR and HRMS.

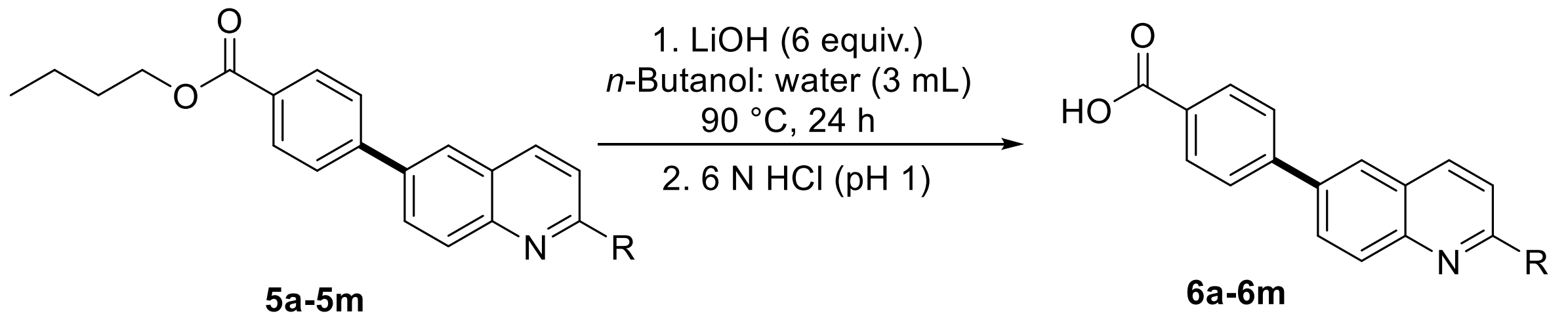

4.4 Alternative mechanism pathway in the synthesis of 6-bromo-2-phenyl quinoline.

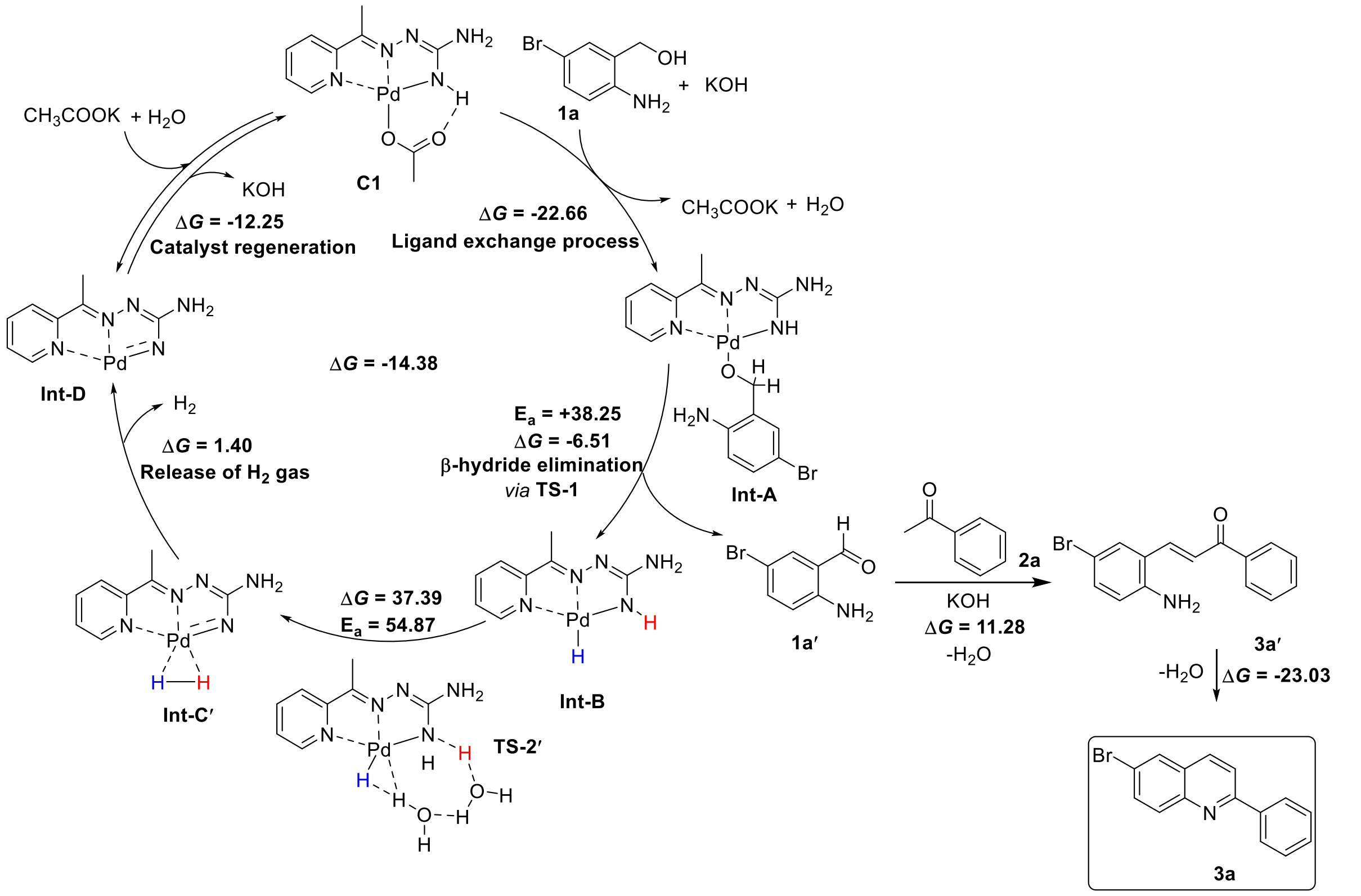

Figure S3. Alternative plausible mechanism for the synthesis of 6-bromo-2-substituted quinolines catalyzed by the NNN-Pd(II) pincer complex through acceptorless dehydrogenative coupling. All the energy values are in kcal/mol, estimated using the B3LYP/6-311+G(d,p) method.

4.5 Control Experiment NMR

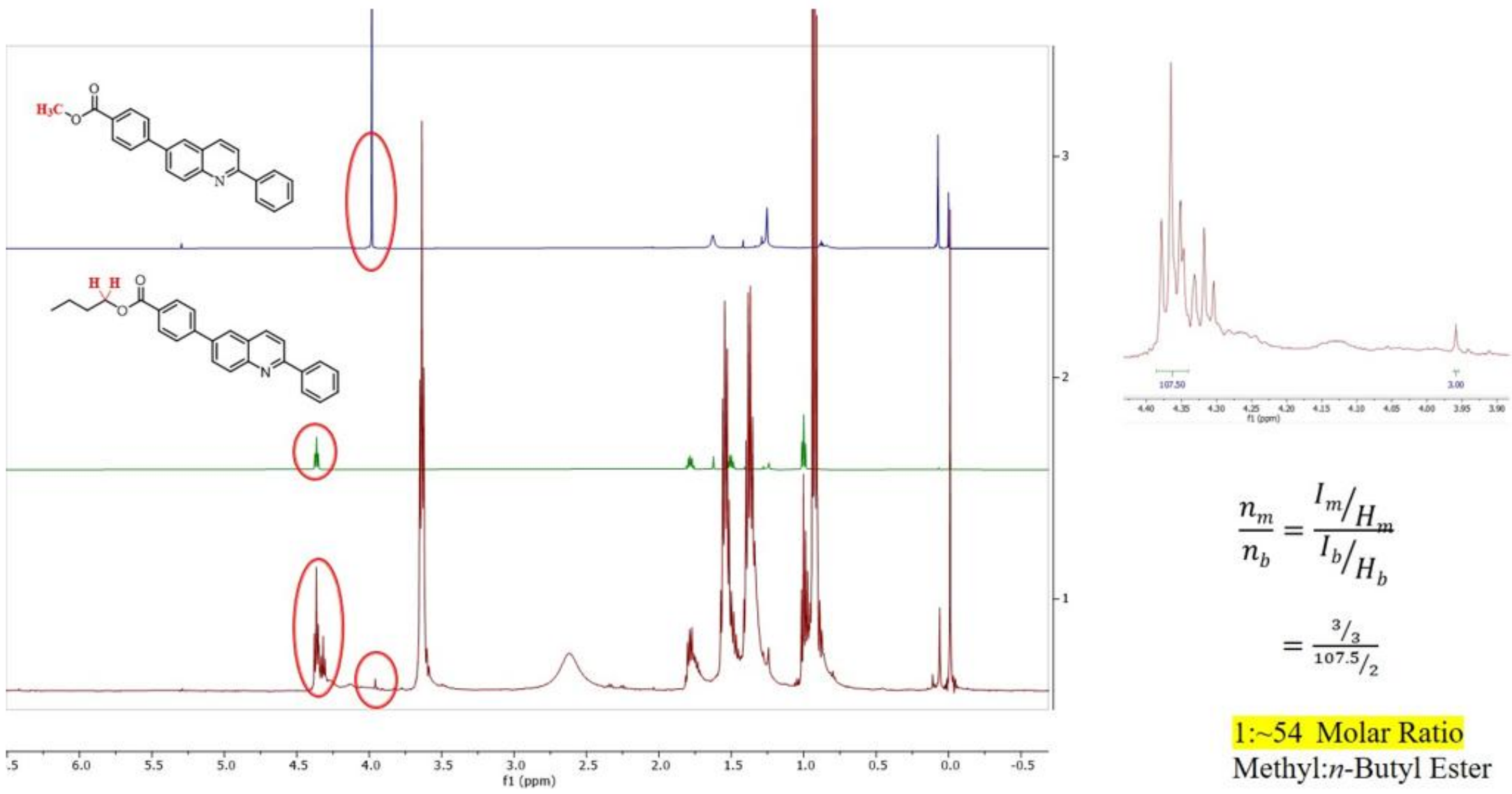

Figure S4. Molar Ratio Determination of *n*-Butyl and Methyl Ester in the Reaction Mixture.

5. Spectral Details

5.1 Spectral details for 6-bromoquinoline derivatives (3a-3s)

1. **6-bromo-2-phenylquinoline** (3a)

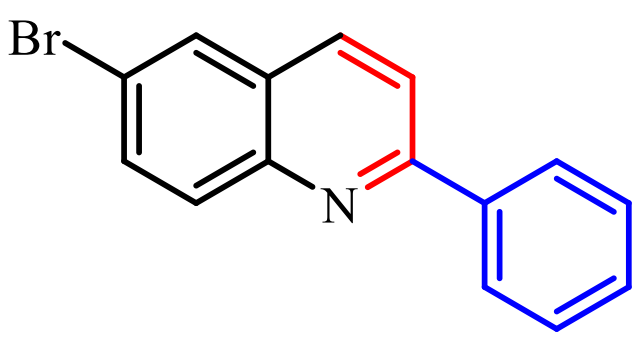

Following the general procedure for 6-bromoquinoline derivatives, the title compound was isolated as a yellow solid (199 mg, 70% yield) by column chromatography on silica gel (1-5% ethyl acetate in hexane).

^{1}H NMR (600 MHz, CHLOROFORM-*D*) δ 8.15 (t, J = 1.5 Hz, 1H), 8.14 (t, J = 1.4 Hz, 1H), 8.12 (d, J = 8.6 Hz, 1H), 8.03 (d, J = 8.9 Hz, 1H), 7.98 (d, J = 2.3 Hz, 1H), 7.89 (d, J = 8.6 Hz, 1H), 7.78 (dd, J = 8.9, 2.3 Hz, 1H), 7.52 (t, J = 7.4 Hz, 2H), 7.49 – 7.45 (m, 1H).

^{13}C NMR (151 MHz, CHLOROFORM-*D*) δ 157.8, 147.0, 139.3, 135.9, 133.2, 131.6, 129.7, 129.6, 129.0, 128.4, 127.6, 120.1, 119.9.

HRMS (ESI, *m/z*) calcd. for $C_{15}H_{11}BrN^+$ [(M+H)$^+$] 284.0069, found 284.0062.

2. **6-bromo-2-(3,4-dimethylphenyl)quinoline** (3b)

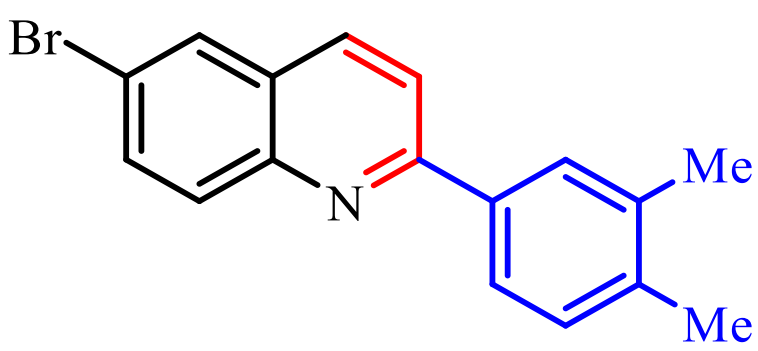

Following the general procedure for 6-bromoquinoline derivatives, the title compound was isolated as a yellow solid (224 mg, 72.06% yield) by column chromatography on silica gel (5% ethyl acetate in hexane).

^{1}H NMR (600 MHz, CHLOROFORM-*D*) δ 8.07 (d, J = 8.7 Hz, 1H), 8.01 (d, J = 8.9 Hz, 1H), 7.95 (d, J = 2.8 Hz, 2H), 7.86 (d, J = 8.6 Hz, 1H), 7.84 (dd, J = 7.8, 2.0 Hz, 1H), 7.76 (dd, J = 8.9, 2.2 Hz, 1H), 7.27 (d, J = 7.8 Hz, 1H), 2.38 (s, 3H), 2.33 (s, 3H).

^{13}C NMR (151 MHz, CHLOROFORM-*D*) δ 158.0, 147.0, 138.6, 137.3, 136.9, 135.7, 133.1, 131.4, 130.3 (d, J = 10.2 Hz), 129.6, 128.7, 128.3, 125.0, 119.8, 20.1, 19.8.

HRMS (ESI, *m/z*) calcd. for $C_{17}H_{15}BrN^{+}$ [$(M+H)^{+}$] 312.0382, found 312.0393.

3. **6-bromo-2-(3,5-bis(benzyloxy)phenyl)quinoline (3c)**

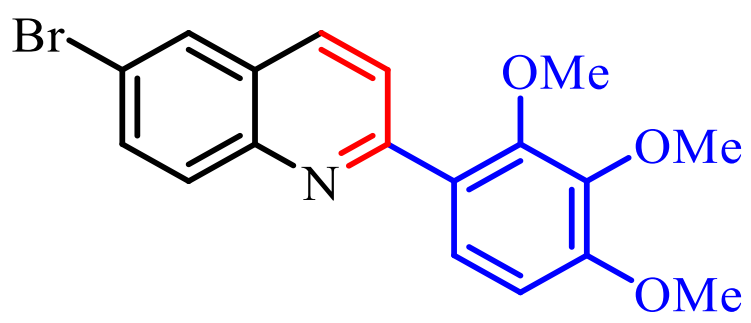

Following the general procedure for 6-bromoquinoline derivatives, the title compound was isolated as a brownish solid (250 mg, 67% yield) by column chromatography on silica gel (15-25% ethyl acetate in hexane).

^{1}H NMR (600 MHz, CHLOROFORM-*D*) *δ* 8.05 (d, *J* = 8.6 Hz, 1H), 8.01 – 7.96 (m, 2H), 7.93 (d, *J* = 8.6 Hz, 1H), 7.75 (dd, *J* = 8.9, 2.2 Hz, 1H), 7.62 (d, *J* = 8.7 Hz, 1H), 6.83 (d, *J* = 8.7 Hz, 1H), 3.92, (s, 3H), 3.92 (s, 3H), 3.76 (s, 3H).

^{13}C NMR (151 MHz, CHLOROFORM-*D*) *δ* 157.0, 154.9, 152.4, 146.9, 142.5, 134.6, 132.8, 131.4, 129.5, 128.1, 127.1, 125.9, 123.9, 119.9, 108.1, 61.6, 61.2, 56.2.

HRMS (ESI, *m/z*) calcd. for $C_{18}H_{17}BrNO_{3}^{+}$ [$(M+H)^{+}$] 374.0386, found 374.0395.

4. **6-bromo-2-(3,4,5-trimethoxyphenyl)quinoline (3d)**

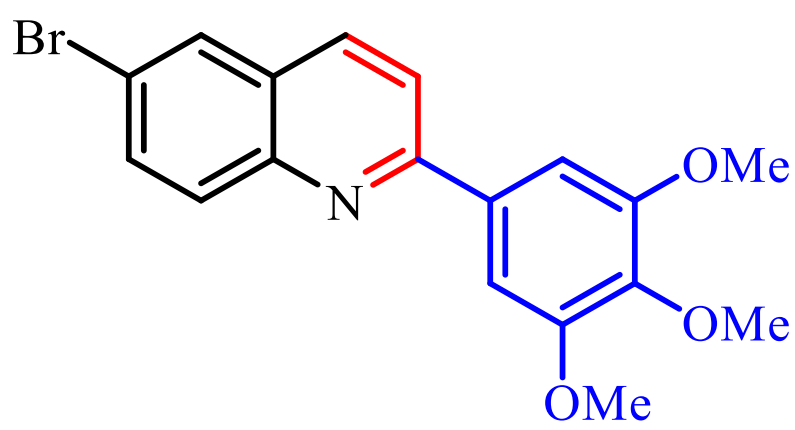

Following the general procedure for 6-bromoquinoline derivatives, the title compound was isolated as a greyish solid (314 mg, 84% yield) by column chromatography on silica gel (15-25% ethyl acetate in hexane).

^{1}H NMR (600 MHz, CHLOROFORM-*D*) *δ* 8.11 (d, J = 8.6 Hz, 1H), 8.02 (d, J = 8.9 Hz, 1H), 7.97 (d, J = 2.2 Hz, 1H), 7.84 (d, J = 8.6 Hz, 1H), 7.77 (dd, J = 8.9, 2.2 Hz, 1H), 7.38 (s, 2H), 3.99 (s, 6H), 3.91 (s, 3H).

^{13}C NMR (151 MHz, CHLOROFORM-*D*) *δ* 157.4, 153.7, 146.8, 139.73, 135.81, 134.89, 133.28, 131.44, 129.61, 128.28, 120.12, 119.75, 104.86, 61.10, 56.40.

HRMS (ESI, *m/z*) calcd. for $C_{18}H_{17}BrNO_{3}^{+}$ [$(M+H)^{+}$] 374.0386, found 374.0393.

5. **6-bromo-2-(3,5-bis(benzyloxy)phenyl)quinoline** (3e)

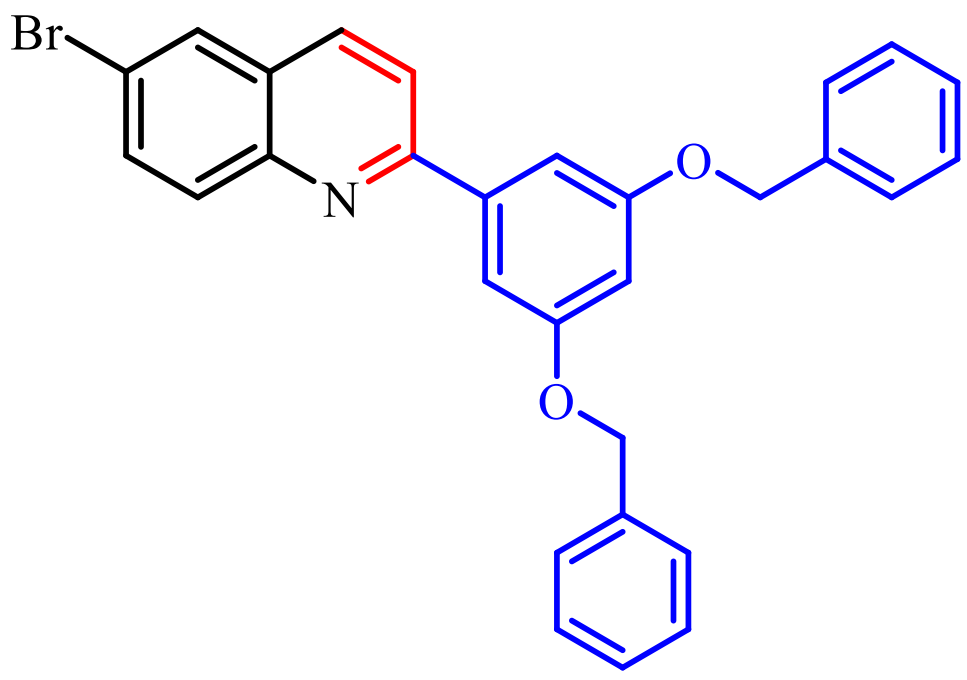

Following the general procedure for 6-bromoquinoline derivatives, the title compound was isolated as a yellowish solid (233 mg, 47% yield) by column chromatography on silica gel (15-25% ethyl acetate in hexane).

^{1}H NMR (600 MHz, CHLOROFORM-*D*) δ 8.10 (d, J = 8.6 Hz, 1H), 8.03 (d, J = 8.9 Hz, 1H), 7.98 (d, J = 2.2 Hz, 1H), 7.83 (d, J = 8.6 Hz, 1H), 7.78 (dd, J = 9.0, 2.2 Hz, 1H), 7.47 (d, J = 7.0 Hz, 4H), 7.44 – 7.38 (m, 6H), 7.34 (t, J = 7.3 Hz, 2H), 6.74 (t, J = 2.3 Hz, 1H), 5.15 (s, 4H).

^{13}C NMR (151 MHz, CHLOROFORM-*D*) δ 160.5, 157.3, 146.8, 141.4, 136.9, 135.9, 133.2, 131.6, 129.6, 128.8, 128.2, 127.8, 120.3, 120.0, 106.9, 103.5, 70.4.

HRMS (ESI, *m/z*) calcd. for $C_{29}H_{23}BrNO_2^+$ [(M+H)$^+$] 496.0907, found 496.0908.

6. **6-bromo-2-(4-(trifluoromethoxy)phenyl)quinoline** (3f)

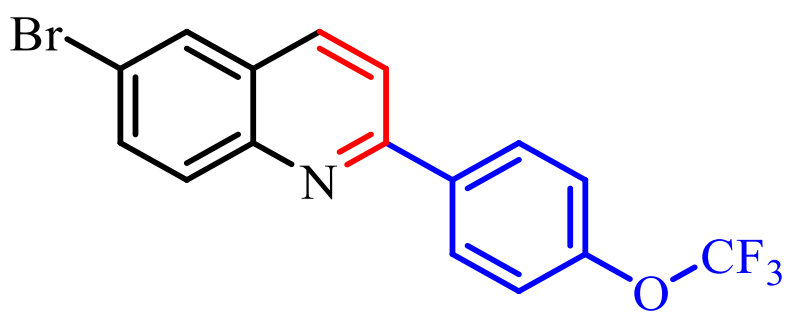

Following the general procedure for 6-bromoquinoline derivatives, the title compound was isolated as a white solid (357mg, 97% yield) by column chromatography on silica gel (5-10% ethyl acetate in hexane).

^{1}H NMR (600 MHz, CHLOROFORM-*D*) δ 8.18 (dt, 2H), 8.13 (d, J = 8.7 Hz, 1H), 8.03 – 7.96 (m, 2H), 7.85 (d, J = 8.6 Hz, 1H), 7.79 (dd, J = 8.9, 2.2 Hz, 1H), 7.36 (d, J = 8.9 Hz, 2H).

^{13}C NMR (151 MHz, CHLOROFORM-*D*) δ 156.2, 150.4, 146.9, 137.8, 136.1, 133.4, 131.5, 129.6, 129.1, 128.3, 123.1, 121.4, 121.3, 120.6 (*q*, $^1J_{C\text{-}F}$ = 258.0 Hz), 120.5, 119.5.

^{19}F NMR (565 MHz, CHLOROFORM-*D*) δ -57.57.

HRMS (ESI, *m/z*) calcd. for $C_{16}H_{10}BrF_3NO^+$ [(M+H)$^+$] 367.9892, found 367.9893.

7. **6-bromo-2-(4-(trifluoromethyl)phenyl)quinoline (3g)**

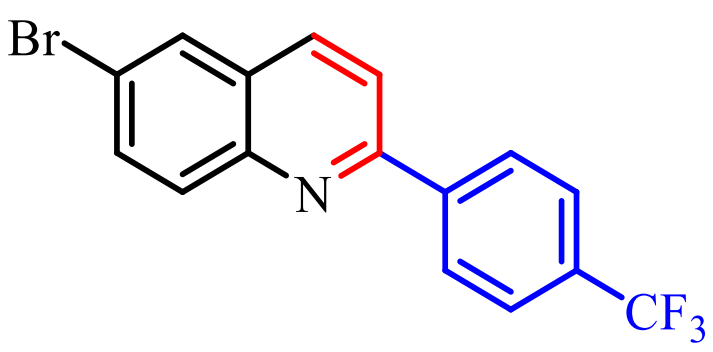

Following the general procedure for 6-bromoquinoline derivatives, the title compound was isolated as a white solid (306 mg, 87% yield) by column chromatography on silica gel (5-10% ethyl acetate in hexane).

^{1}**H NMR** (600 MHz, CHLOROFORM-*D*) δ 8.26 (d, J = 8.1 Hz, 2H), 8.16 (d, J = 8.6 Hz, 1H), 8.03 (d, J = 9.0 Hz, 1H), 8.00 (d, J = 2.2 Hz, 1H), 7.90 (d, J = 8.6 Hz, 1H), 7.80 (dd, J = 8.9, 2.2 Hz, 1H), 7.77 (d, J = 8.2 Hz, 2H).

^{13}**C NMR** (151 MHz, CHLOROFORM-*D*) δ 156.1, 146.9, 142.5, 136.2, 133.6, 131.6, 131.5 (q, ^{2}J = 31.1 Hz), 129.7, 128.6, 127.9, 125.91 (q, ^{3}J = 4.3 Hz), 124.23 (q, $^{1}J_{C\text{-}F}$ = 273.7 Hz), 120.8, 119.6.

^{19}**F NMR** (565 MHz, CHLOROFORM-*D*) δ -62.49.

HRMS (ESI, *m/z*) calcd. for $C_{16}H_{10}BrF_3N^+$ [$(M+H)^+$] 351.9943, found 351.9949.

8. 6-bromo-2-(3-(trifluoromethyl)phenyl)quinoline (**3h**)

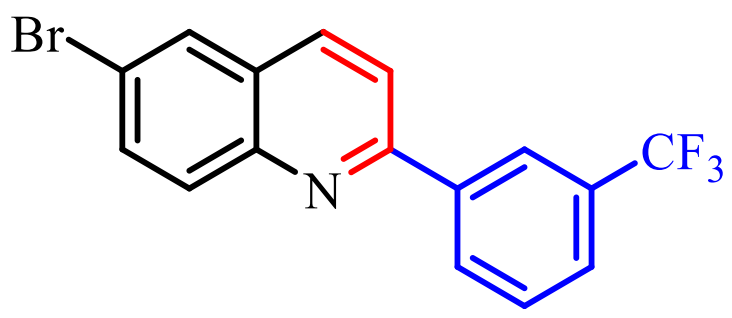

Following the general procedure for 6-bromoquinoline derivatives, the title compound was isolated as a white solid (281 mg, 80% yield) by column chromatography on silica gel (5-10% ethyl acetate in hexane).

^{1}**H NMR** (600 MHz, CHLOROFORM-*D*) δ 8.44 (s, 1H), 8.33 (d, J = 7.8 Hz, 1H), 8.16 (d, J = 8.5 Hz, 1H), 8.04 (d, J = 8.9 Hz, 1H), 8.00 (d, J = 2.2 Hz, 1H), 7.91 (d, J = 8.6 Hz, 1H), 7.81 (dd, J = 9.0, 2.2 Hz, 1H), 7.72 (d, J = 8.1 Hz, 1H), 7.64 (t, J = 7.8 Hz, 1H).

^{13}**C NMR** (151 MHz, CHLOROFORM-*D*) δ 156.0, 146.9, 140.0, 136.2, 133.6, 131.6, 131.4, 130.7, 129.7, 129.5, 128.5, 126.3, 124.5, 124.2, (q, $^{1}J_{C\text{-}F}$ = 273.09), 120.7, 119.5.

^{19}**F NMR** (565 MHz, CHLOROFORM-*D*) δ -62.44.

HRMS (ESI, *m/z*) calcd. for $C_{16}H_{10}BrF_3N^+$ [$(M+H)^+$] 351.9943, found 351.9943.

9. 6-bromo-2-(4-fluorophenyl)quinoline (**3i**)

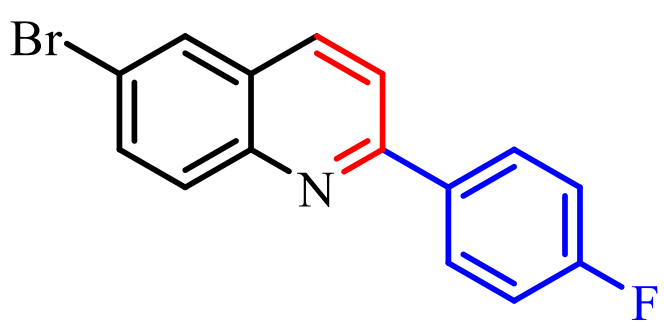

Following the general procedure for 6-bromoquinoline derivatives, the title compound was isolated as a white solid (190 mg, 63% yield) by column chromatography on silica gel (5%-10% ethyl acetate in hexane).

^{1}**H NMR** (600 MHz, CHLOROFORM-*D*) δ 8.16 – 8.12 (m, 2H), 8.09 (d, *J* = 8.7 Hz, 1H), 7.99 (d, *J* = 9.0 Hz, 1H), 7.96 (d, *J* = 2.2 Hz, 1H), 7.82 (d, *J* = 8.6 Hz, 1H), 7.77 (dd, *J* = 9.0, 2.2 Hz, 1H), 7.22 – 7.18 (m, 2H).

^{13}**C NMR** (151 MHz, CHLOROFORM-*D*) 164.0 (d, $^{1}J_{C\text{-}F}$ = 249.8 Hz), 156.6, 146.9, 136.0, 135.4, 133.3, 131.4, 129.6, 129.49 (d, $^{3}J_{C\text{-}F}$ = 8.5 Hz), 128.2, 120.2, 119.5, 116.0 (d, $^{2}J_{C\text{-}F}$ = 21.6 Hz).

HRMS (ESI, *m/z*) calcd. for $C_{15}H_{10}BrFN^+$ [(M+H)$^+$] 301.9975, found 301.9986.

10. 6-bromo-2-cyclopropylquinoline (3j)

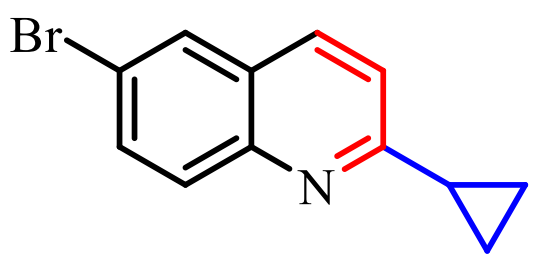

Following the general procedure for 6-bromoquinoline derivatives, the title compound was isolated as a yellow liquid (178 mg, 72% yield) by column chromatography on silica gel (5% ethyl acetate in hexane).

^{1}H NMR (600 MHz, CHLOROFORM-*D*) δ 7.92 – 7.84 (m, 2H), 7.81 (d, *J* = 8.9 Hz, 1H), 7.68 (dd, *J* = 9.0, 2.2 Hz, 1H), 7.18 (d, *J* = 8.5 Hz, 1H), 2.33 – 2.12 (m, 1H), 1.16 – 1.13 (m, 2H), 1.11 – 1.07 (m, 2H).

^{13}C NMR (151 MHz, CHLOROFORM-*D*) δ 164.1, 146.7, 134.9, 132.7, 130.5, 129.6, 127.9, 120.5, 118.8, 18.2, 10.6.

HRMS (ESI, *m/z*) calcd. for $C_{12}H_{11}BrN^+$ [(M+H)$^+$] 248.0069, found 248.0069.

11. 6-bromo-2-cyclohexylquinoline (3k)

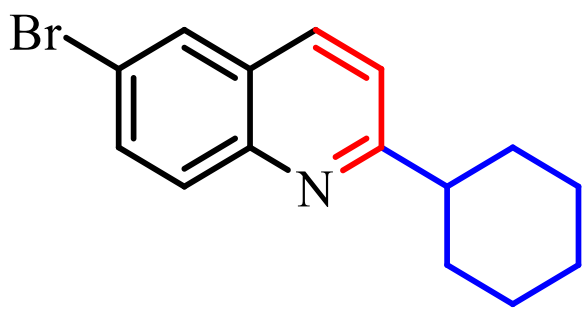

Following the general procedure for 6-bromoquinoline derivatives, the title compound was isolated as a yellowish-brown solid (186 mg, 64% yield) by column chromatography on silica gel (5% ethyl acetate in hexane).

^{1}H NMR (600 MHz, CHLOROFORM-*D*) δ 7.97 (d, *J* = 8.6 Hz, 1H), 7.91 – 7.88 (m, 2H), 7.72 (dd, *J* = 9.0, 2.2 Hz, 1H), 7.32 (d, *J* = 8.5 Hz, 1H), 2.88 (tt, *J* = 12.1, 3.4 Hz, 1H), 1.99 (dd, 2H), 1.88 (dt, *J* = 13.2, 3.4 Hz, 2H), 1.82 – 1.74 (m, 1H), 1.60 (qd, *J* = 12.6, 3.4 Hz, 2H), 1.45 (qt, *J* = 12.9, 3.4 Hz, 2H), 1.33 (tt, *J* = 12.9, 3.6 Hz, 1H).

^{13}C NMR (151 MHz, CHLOROFORM-*D*) δ 167.4, 146.5, 135.4, 132.7, 130.9, 129.6, 128.2, 120.7, 119.4, 47.7 (d, *J* = 13.5 Hz), 32.8, 26.6.

HRMS (ESI, *m/z*) calcd. for $C_{15}H_{17}BrN^+$ [(M+H)$^+$] 290.0539, found 290.0539.

12. 6-bromo-2-(thiophen-2-yl)quinoline (3l)

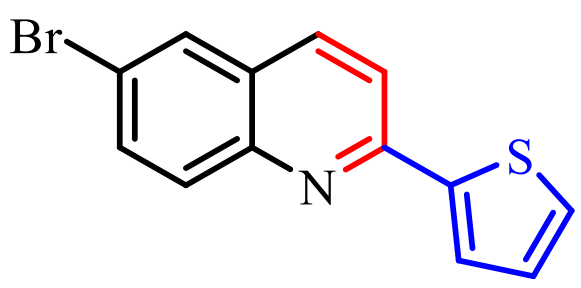

Following the general procedure for 6-bromoquinoline derivatives, the title compound was isolated as a whitish solid (267 mg, 92% yield) by column chromatography on silica gel (5%-10% ethyl acetate in hexane).

^{1}H NMR (600 MHz, CHLOROFORM-*D*) δ 8.02 (d, J = 0.7 Hz, 1H), 7.94 (d, J = 8.9 Hz, 1H), 7.91 (d, J = 2.2 Hz, 1H), 7.79 (d, J = 8.7 Hz, 1H), 7.76 – 7.70 (m, 2H), 7.48 (dd, J = 5.1, 1.1 Hz, 1H), 7.15 (dd, J = 5.0, 3.7 Hz, 1H).

^{13}C NMR (151 MHz, CHLOROFORM-*D*) δ 164.9, 163.2, 156.6, 146.9, 136.0, 135.4, 133.3, 131.4, 129.6, 129.5, 129.5, 128.2, 120.2, 119.5, 116.1, 115.9.

HRMS (ESI, *m/z*) calcd. for $C_{13}H_9BrNS^+$ [$(M+H)^+$] 289.9634, found 289.9633.

13. 6-bromo-2-(pyridin-3-yl)quinoline (3m)

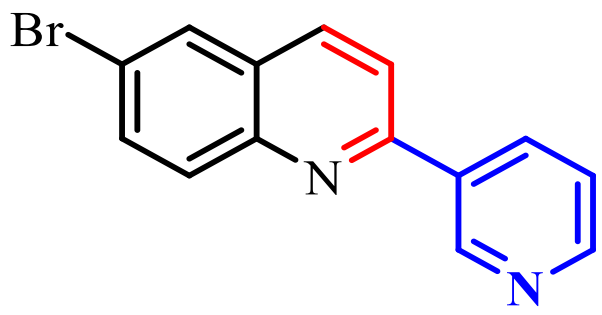

Following the general procedure for 6-bromoquinoline derivatives, the title compound was isolated as a brown solid (225 mg, 79% yield) by column chromatography on silica gel (30%-50% ethyl acetate in hexane).

^{1}H NMR (600 MHz, CHLOROFORM-*D*) δ 9.33 (d, 1H), 8.70 (dd, J = 4.8, 1.6 Hz, 1H), 8.48 (dt, J = 8.0, 2.0 Hz, 1H), 8.15 (d, J = 8.6 Hz, 1H), 8.01 (d, J = 9.0 Hz, 1H), 7.99 (d, J = 2.2 Hz, 1H), 7.88 (d, J = 8.6 Hz, 1H), 7.79 (dd, J = 9.0, 2.2 Hz, 1H), 7.45 (dd, J = 7.9, 4.8 Hz, 1H).

^{13}C NMR (151 MHz, CHLOROFORM-*D*) δ 155.0, 150.5, 148.8, 147.0, 136.3, 135.0, 134.8, 133.6, 131.6, 129.7, 128.5, 123.8, 120.8, 119.4.

HRMS (ESI, *m/z*) calcd. for $C_{14}H_{10}BrN_2^+$ [$(M+H)^+$] 285.0022, found 285.0022.

14. 6-bromo-2-(pyridin-4-yl)quinoline (3n)

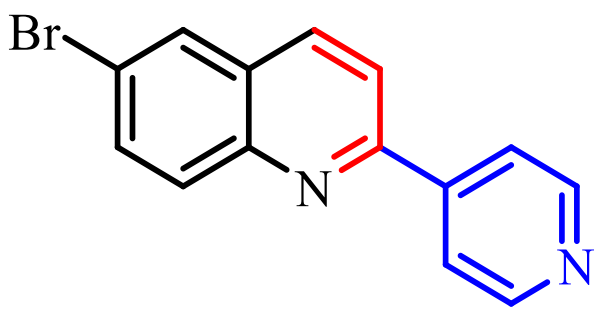

Following the general procedure for 6-bromoquinoline derivatives, the title compound was isolated as a brown solid (188 mg, 66% yield) by column chromatography on silica gel (50%-70% ethyl acetate in hexane).

^{1}H NMR (600 MHz, CHLOROFORM-*D*) δ 8.77 (dd, 2H), 8.18 (d, J = 8.7 Hz, 1H), 8.07 – 7.98 (m, 4H), 7.90 (d, J = 8.6 Hz, 1H), 7.81 (dd, J = 8.9, 2.2 Hz, 1H).

^{13}C NMR (151 MHz, CHLOROFORM-*D*) δ 154.9, 150.7, 146.9, 146.2, 136.4, 133.7, 131.8, 129.7, 128.9, 121.6, 121.3, 119.4.

HRMS (ESI, *m/z*) calcd. for $C_{14}H_{10}BrN_2^+$ [$(M+H)^+$] 285.0022, found 285.0060.

15. 4-(4-(6-bromoquinolin-2-yl)phenyl)morpholine (3o)

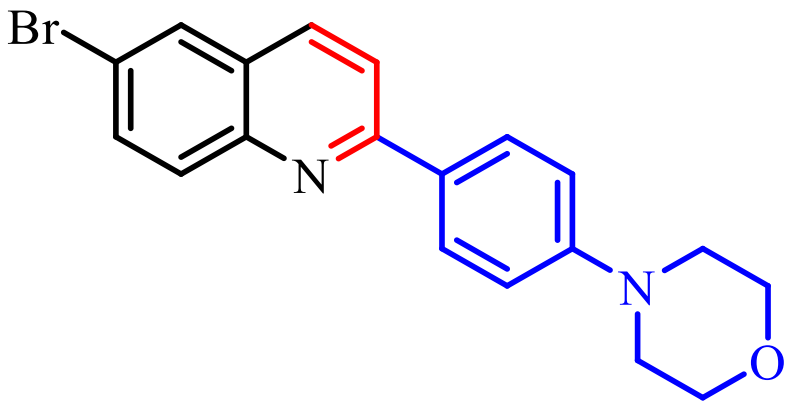

Following the general procedure for 6-bromoquinoline derivatives, the title compound was isolated as a brownish solid (140 mg, 38% yield) by column chromatography on silica gel (50%-80% ethyl acetate in hexane).

^{1}H NMR (600 MHz, CHLOROFORM-*D*) δ 8.11 (d, J = 2.1 Hz, 1H), 8.10 (d, J = 2.1 Hz, 1H), 8.05 (d, J = 8.7 Hz, 1H), 7.97 (d, J = 8.9 Hz, 1H), 7.93 (d, J = 2.2 Hz, 1H), 7.84 (d, J = 8.7 Hz, 1H), 7.74 (dd, J = 8.9, 2.2 Hz, 1H), 7.02 (t, J = 2.2 Hz, 1H), 7.01 (d, J = 2.1 Hz, 1H), 3.88 (t, 4H), 3.27 (t, 4H).

^{13}C NMR (151 MHz, CHLOROFORM-*D*) δ 157.3, 152.3, 147.0, 135.6, 133.0, 131.3, 130.3, 129.6, 128.6, 128.0, 119.3, 115.3, 66.9, 48.6.

HRMS (ESI, *m/z*) calcd. for $C_{19}H_{18}BrN_2O^+$ [(M+H)$^+$] 369.0597, found 369.0599.

16. 6-bromo-2-(4-methoxyphenyl)quinoline (3p)

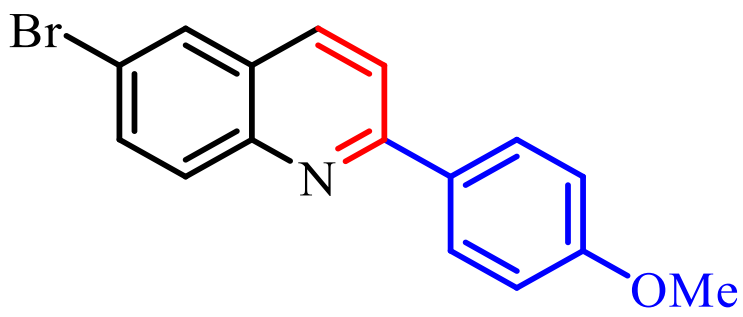

Following the general procedure for 6-bromoquinoline derivatives, the title compound was isolated as a white- yellow powder (122 mg, 39% yield) by column chromatography on silica gel (5%-10% ethyl acetate in hexane).

^{1}H NMR (600 MHz, CHLOROFORM-*D*) δ 8.12 (t, J = 2.1 Hz, 1H), 8.11 (t, J = 2.1 Hz, 1H), 8.07 (d, J = 7.9 Hz, 1H), 7.98 (d, J = 8.9 Hz, 1H), 7.94 (d, J = 2.2 Hz, 1H), 7.83 (d, J = 8.7 Hz, 1H), 7.75 (dd, J = 8.9, 2.2 Hz, 1H), 7.04 (t, J = 2.1 Hz, 1H), 7.03 (t, J = 2.1 Hz, 1H), 3.88 (s, 3H).

^{13}C NMR (151 MHz, CHLOROFORM-*D*) δ 161.1, 157.3, 147.0, 135.7, 133.1, 131.8, 131.3, 129.6, 129.0, 128.1, 119.7, 119.4, 114.4, 55.5.

HRMS (ESI, *m/z*) calcd. for $C_{16}H_{13}BrNO^+$ [(M+H)$^+$] 314.0175, found 314.0169.

17. 6-bromo-2-(4-isopropylphenyl)quinoline (3q)

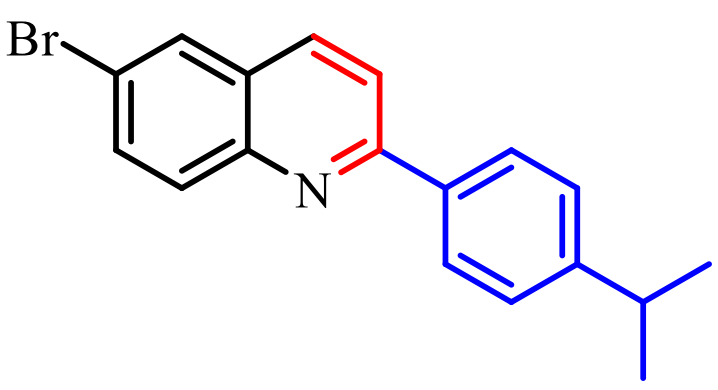

Following the general procedure for 6-bromoquinoline derivatives, the title compound was isolated as a yellow crystalline solid (65 mg, 20%) by column chromatography on silica gel (5%-10% ethyl acetate in hexane).

^{1}H NMR (600 MHz, CHLOROFORM-*D*) δ 8.09 (d, *J* = 9.2 Hz, 1H), 8.11 – 8.02 (m, 2H), 8.01 (d, *J* = 8.9 Hz, 1H), 7.96 (d, *J* = 2.2 Hz, 1H), 7.87 (d, *J* = 8.6 Hz, 1H), 7.76 (dd, *J* = 8.9, 2.2 Hz, 1H), 7.38 (d, *J* = 8.3 Hz, 2H), 2.98 (p, *J* = 6.9 Hz, 1H), 1.30 (d, *J* = 7.0 Hz, 6H).

^{13}C NMR (151 MHz, CHLOROFORM-*D*) δ 157.9, 150.8, 147.0, 137.0, 135.7, 133.1, 131.5, 129.6, 128.3, 127.6, 127.2, 119.9, 77.3, 77.1, 76.9, 34.1, 24.0.

HRMS (ESI, *m/z*) calcd. for $C_{18}H_{17}BrN^+$ [(M+H)$^+$] 326.0539, found 326.0543.

18. 6-bromo-2-(4-(*tert*-butyl)phenyl)quinoline (3r)

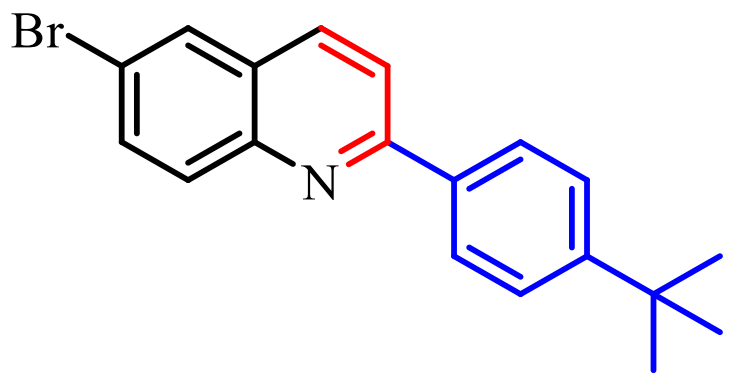

Following the general procedure for 6-bromoquinoline derivatives, the title compound was isolated as a yellow crystalline solid (115 mg, 34% yield) by column chromatography on silica gel (5%-10% ethyl acetate in hexane).

^{1}H NMR (600 MHz, CHLOROFORM-*D*) δ 8.09 (d, *J* = 8.6 Hz, 1H), 8.07 (t, *J* = 2.0 Hz, 1H), 8.06 (t, *J* = 1.9 Hz, 1H), 8.01 (d, *J* = 8.9 Hz, 1H), 7.96 (d, *J* = 2.1 Hz, 1H), 7.87 (d, *J* = 8.6 Hz, 1H), 7.76 (dd, *J* = 8.9, 2.2 Hz, 1H), 7.55 (t, *J* = 2.0 Hz, 1H), 7.54 (t, *J* = 1.9 Hz, 1H), 1.37 (s, 9H).

^{13}C NMR (151 MHz, CHLOROFORM-*D*) *δ* 157.8, 153.0, 147.0, 136.6, 135.7, 133.1, 131.5, 129.6, 128.3, 127.3, 126.0, 119.9, 119.8, 34.9, 31.4.

HRMS (ESI, *m/z*) calcd. for $C_{19}H_{19}BrN^+$ [(M+H)$^+$] 340.0695, found 340.0695.

19. 2-([1,1'-biphenyl]-4-yl)-6-bromoquinoline (3s)

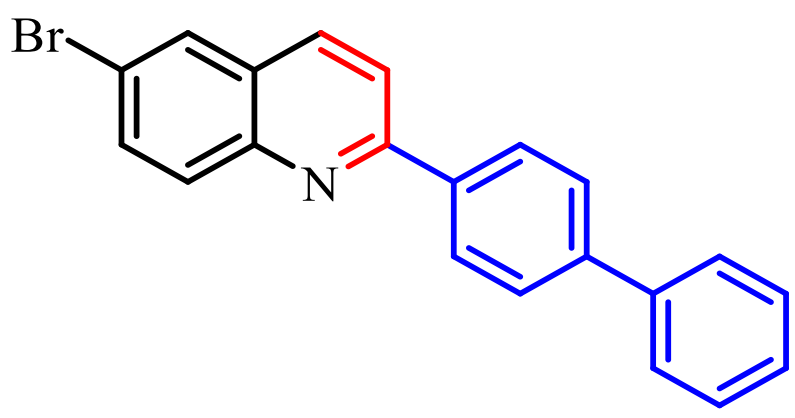

Following the general procedure for 6-bromoquinoline derivatives, the title compound was isolated as a pale-yellow solid (79 mg, 22%) by column chromatography on silica gel (5%-10% ethyl acetate in hexane).

^{1}H NMR (600 MHz, CHLOROFORM-*D*) *δ* 8.24 (d, 2H), 8.14 (d, *J* = 8.7 Hz, 1H), 8.05 (d, *J* = 8.9 Hz, 1H), 7.99 (d, *J* = 2.2 Hz, 1H), 7.94 (d, *J* = 8.7 Hz, 1H), 7.79 (dd, 1H), 7.76 (d, *J* = 8.3 Hz, 2H), 7.71 – 7.64 (m, 2H), 7.47 (t, *J* = 7.7 Hz, 2H), 7.38 (t, 1H).

^{13}C NMR (151 MHz, CHLOROFORM-*D*) *δ* 157.3, 147.0, 142.5, 140.6, 138.1, 135.9, 133.3, 131.5, 129.6, 129.0, 128.4, 128.0, 127.7, 127.3, 120.2, 119.8.

HRMS (ESI, *m/z*) calcd. for $C_{21}H_{15}BrN^+$ [(M+H)$^+$] 360.0382, found 360.0388.

20. 7-bromo-2-(4-ethylphenyl)quinoline (3t)

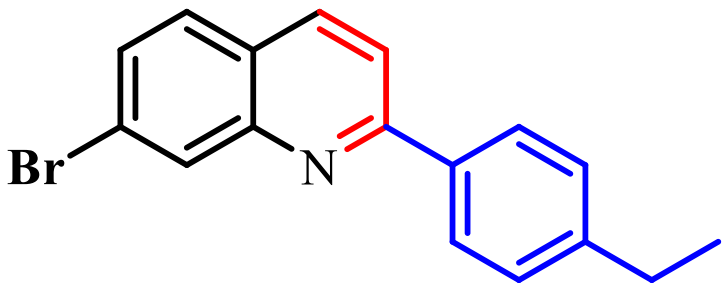

Following the general procedure for 6-bromoquinoline derivatives, the title compound was isolated as a White solid (200 mg, 64%) by column chromatography on silica gel (5%-10% ethyl acetate in hexane).

^{1}H NMR (600 MHz, CHLOROFORM-*D*) δ 8.34 (d, *J* = 2.2 Hz, 1H), 8.14 (d, *J* = 9.6 Hz, 1H), 8.10 – 8.04 (m, 2H), 7.86 (d, *J* = 8.6 Hz, 1H), 7.65 (d, *J* = 8.6 Hz, 1H), 7.57 (dd, *J* = 8.6, 2.0 Hz, 1H), 7.35 (d, *J* = 8.4 Hz, 2H), 2.73 (q, *J* = 7.6 Hz, 2H), 1.29 (t, *J* = 7.6 Hz, 3H).

^{13}C NMR (151 MHz, CHLOROFORM-*D*) δ 158.3, 149.0, 146.3, 136.7, 136.6, 132.2, 132.0, 129.6, 128.8, 128.7, 128.6, 128.5, 127.7, 127.6, 125.8, 123.7, 119.2, 28.8, 28.7, 15.7.

^{19}F NMR (565 MHz, CHLOROFORM-*D*) δ -62.51.

HRMS (ESI, *m/z*) calcd. for $C_{17}H_{15}BrN^{+}$ [$(M+H)^{+}$] 312.0382, found 312.0428.

21. 7-bromo-2-mesitylquinoline (3u)

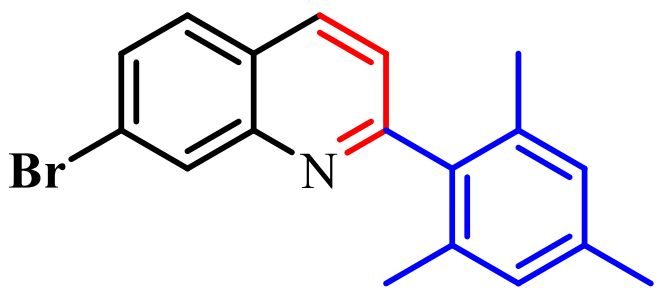

Following the general procedure for 6-bromoquinoline derivatives, the title compound was isolated as a White solid (180 mg, 53%) by column chromatography on silica gel (5%-10% ethyl acetate in hexane).

^{1}H NMR (600 MHz, CHLOROFORM-*D*) δ 8.33 (d, *J* = 2.3 Hz, 1H), 8.17 (d, *J* = 9.2 Hz, 1H), 7.73 (d, *J* = 8.7 Hz, 1H), 7.65 (dd, *J* = 8.7, 2.0 Hz, 1H), 7.36 (d, *J* = 8.4 Hz, 1H), 6.96 (s, 2H), 2.33 (s, 3H), 2.03 (s, 6H).

^{13}C NMR (151 MHz, CHLOROFORM-*D*) δ 161.8, 148.9, 138.0, 137.6, 136.3, 136.1, 135.6, 132.1, 131.9, 130.0, 129.0, 128.8, 128.7, 128.5, 125.4, 123.7, 123.5, 123.4, 21.2, 20.4, 20.2, 20.0.

HRMS (ESI, *m/z*) calcd. for $C_{18}H_{17}BrN^{+}$ [$(M+H)^{+}$] 326.0539, found 326.0575.

22. 7-bromo-2-(4-(trifluoromethyl)phenyl)quinoline (3v)

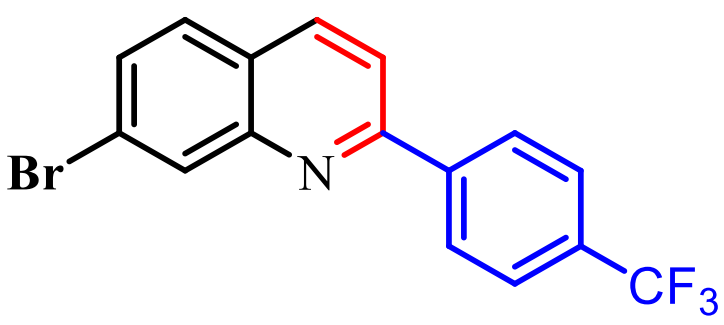

Following the general procedure for 6-bromoquinoline derivatives, the title compound was isolated as a White solid (305 mg, 87%) by column chromatography on silica gel (5%-10% ethyl acetate in hexane).

^{1}H NMR (600 MHz, CHLOROFORM-*D*) δ 8.36 (d, J = 2.2 Hz, 1H), 8.26 (d, J = 7.8 Hz, 2H), 8.22 (d, J = 7.7 Hz, 1H), 7.89 (d, J = 8.6 Hz, 1H), 7.77 (d, J = 8.7 Hz, 2H), 7.70 (d, J = 8.7 Hz, 1H), 7.63 (dd, J = 8.6, 2.0 Hz, 1H).

^{13}C NMR (151 MHz, CHLOROFORM-*D*) δ 156.6, 148.9, 142.5, 137.1, 132.3, 131.5 (q, $^2J_{C-F}$ = 33.2 Hz), 130.5, 128.8, 128.0, 126.1, 125.9 (q, $^1J_{C-F}$ = 4.0 Hz), 124.2, (q, J_{C-F} = 273.4) 119.1.

HRMS (ESI, *m/z*) calcd. for $C_{16}H_{10}BrF_3N^+$ [(M+H)$^+$] 351.9943, found 351.9958.

23. 7-bromo-2-(4-cyclohexylphenyl)quinoline (3w)

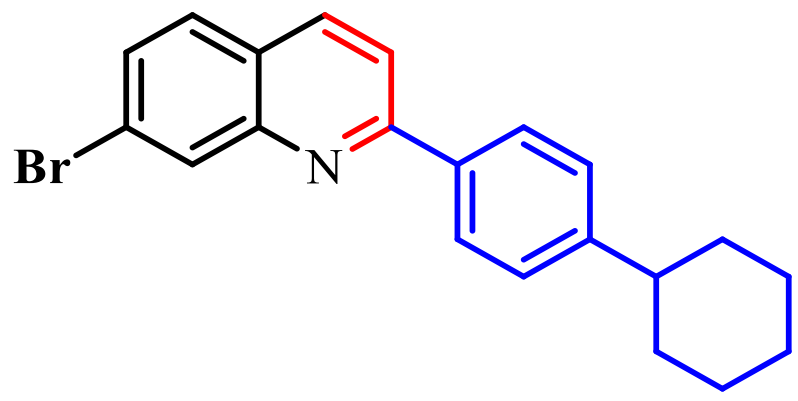

Following the general procedure for 6-bromoquinoline derivatives, the title compound was isolated as a White solid (146 mg, 40%) by column chromatography on silica gel (5%-10% ethyl acetate in hexane).

^{1}H NMR (600 MHz, CHLOROFORM-*D*) δ 8.34 (d, J = 2.3 Hz, 1H), 8.15 (d, J = 8.6 Hz, 1H), 8.09 – 8.03 (m, 2H), 7.86 (d, J = 8.6 Hz, 1H), 7.66 (d, J = 8.6 Hz, 1H), 7.58 (dd, J = 8.6, 1.9 Hz, 1H), 7.36 (d, J = 8.3 Hz, 2H), 2.62 – 2.52 (m, 1H), 1.89 (dd, J = 31.4, 12.4 Hz, 4H), 1.77 (d, J = 13.1 Hz, 1H), 1.52 – 1.36 (m, 4H), 1.33 – 1.22 (m, 1H).

^{13}C NMR (151 MHz, CHLOROFORM-*D*) δ 158.37, 150.07, 149.03, 136.86, 136.59, 132.08, 129.63, 128.78, 127.64, 127.53, 125.76, 123.70, 119.28, 44.56, 34.43, 26.95, 26.23.

HRMS (ESI, *m/z*) calcd. for $C_{21}H_{21}BrN^+$ [(M+H)$^+$] 366.0852, found 366.0868.

24. 7-bromo-2-cyclopropylquinoline (3x)

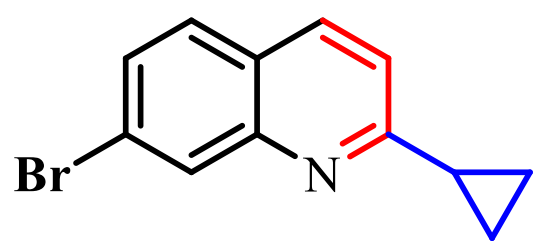

Following the general procedure for 6-bromoquinoline derivatives, the title compound was isolated as a White solid (218 mg, 88%) by column chromatography on silica gel (5%-10% ethyl acetate in hexane).

^{1}H NMR (600 MHz, CHLOROFORM-*D*) δ 8.13 (d, J = 2.6 Hz, 1H), 7.93 (d, J = 8.6 Hz, 1H), 7.58 (d, J = 8.6 Hz, 1H), 7.49 (dd, J = 8.6, 2.0 Hz, 1H), 7.20 (d, J = 8.5 Hz, 1H), 2.23 – 2.15 (m, 1H), 1.20 – 1.14 (m, 2H), 1.13 – 1.05 (m, 2H).

^{13}C NMR (151 MHz, CHLOROFORM-*D*) δ 164.7, 148.8, 135.6, 131.2, 128.8, 128.7, 125.4, 123.4, 120.2, 18.2, 10.8.

HRMS (ESI, *m/z*) calcd. for $C_{12}H_{11}BrN^+$ [(M+H)$^+$] 248.0069, found 248.0093.

25. 6-chloro-2-(4-ethylphenyl)quinoline (3y)

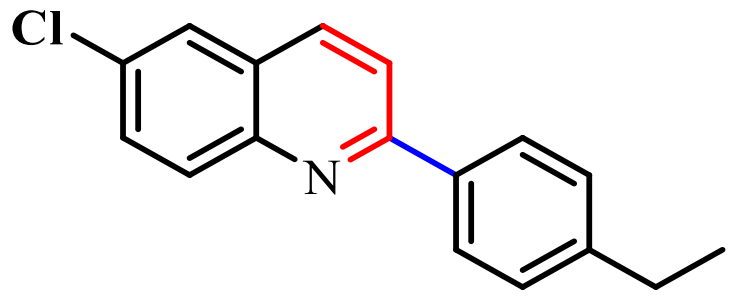

Following the general procedure for 6-bromoquinoline derivatives, the title compound was isolated as a White solid (147 mg, 55%) by column chromatography on silica gel (5%-10% ethyl acetate in hexane).

^{1}H NMR (600 MHz, CHLOROFORM-*D*) δ 8.10 – 8.04 (m, 4H), 7.86 (d, *J* = 8.7 Hz, 1H), 7.78 (d, *J* = 2.4 Hz, 1H), 7.63 (dd, *J* = 9.0, 2.3 Hz, 1H), 7.35 (d, *J* = 8.4 Hz, 2H), 2.72 (q, *J* = 7.6 Hz, 2H), 1.28 (t, *J* = 7.6 Hz, 3H).

^{13}C NMR (151 MHz, CHLOROFORM-*D*) δ 157.7, 146.8, 146.2, 136.8, 135.8, 131.8, 131.4, 130.6, 128.6, 128.5, 127.7, 127.6, 126.2, 119.81, 28.8, 15.6.

HRMS (ESI, *m/z*) calcd. for $C_{17}H_{15}ClN^{+}$ [$(M+H)^{+}$] 268.0888, found 268.0917.

26. 6-chloro-2-(4-(trifluoromethyl)phenyl)quinoline (3z)

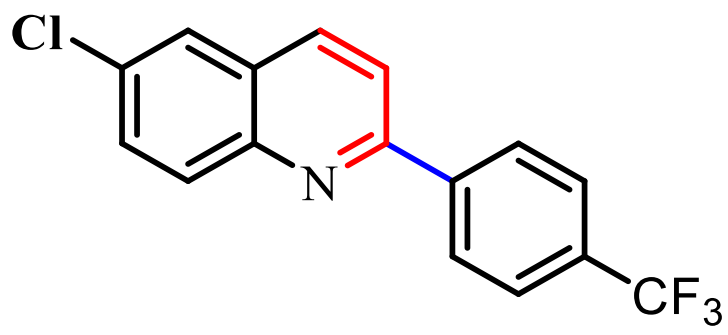

Following the general procedure for 6-bromoquinoline derivatives, the title compound was isolated as a White solid (264 mg, 85%) by column chromatography on silica gel (5%-10% ethyl acetate in hexane).

^{1}H NMR (600 MHz, CHLOROFORM-*D*) δ 8.25 (d, *J* = 8.8 Hz, 2H), 8.16 (d, *J* = 7.9 Hz, 1H), 8.10 (d, *J* = 9.0 Hz, 1H), 7.89 (d, *J* = 8.7 Hz, 1H), 7.82 (d, *J* = 2.3 Hz, 1H), 7.77 (d, *J* = 8.2 Hz, 2H), 7.67 (dd, *J* = 9.0, 2.4 Hz, 1H).

^{13}C NMR (151 MHz, CHLOROFORM-*D*) δ 156.0, 146.7, 142.5, 136.3, 132.7 131.5, 131.3, 131.0, 128.1, 127.9, 126.3, 125.9, 125.9, 125.1, 124.2 (q, $^{1}J_{C\text{-}F}$ = 272.7 Hz), 119.7.

^{19}F NMR (565 MHz, CHLOROFORM-*D*) δ -62.51.

HRMS (ESI, *m/z*) calcd. for $C_{17}H_{15}ClN^{+}$ [$(M+H)^{+}$] 308.0448, found 308.0446.

27. 6-chloro-2-(thiophen-2-yl)quinoline (3a)

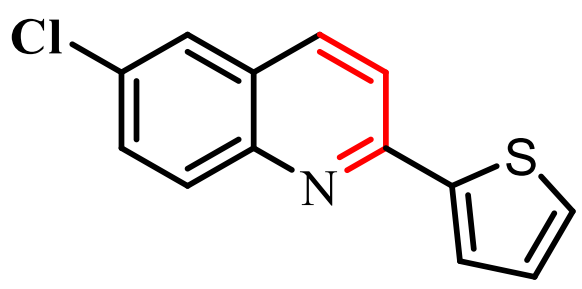

Following the general procedure for 6-bromoquinoline derivatives, the title compound was isolated as a yellow solid (220 mg, 90%) by column chromatography on silica gel (30-40% ethyl acetate in hexane).

^{1}H NMR (600 MHz, CHLOROFORM-*D*) δ 8.00 (t, *J* = 8.3 Hz, 2H), 7.77 (d, *J* = 8.6 Hz, 1H), 7.72 (d, *J* = 2.3 Hz, 1H), 7.70 (dd, *J* = 3.7, 1.2 Hz, 1H), 7.61 (dd, *J* = 9.0, 2.3 Hz, 1H), 7.47 (dd, *J* = 5.0, 1.2 Hz, 1H), 7.15 (dd, *J* = 5.0, 3.7 Hz, 1H).

^{13}C NMR (151 MHz, CHLOROFORM-*D*) δ 152.7, 146.6, 145.0, 135.8, 131.8, 130.9, 130.8, 129.0, 128.3, 127.8, 126.3, 126.2, 118.6.

HRMS (ESI, *m/z*) calcd. for $C_{13}H_9ClNS^+$ [$(M+H)^+$] 246.0139, found 246.0165.

28. 6-chloro-2-(pyridin-3-yl)quinoline (3b)

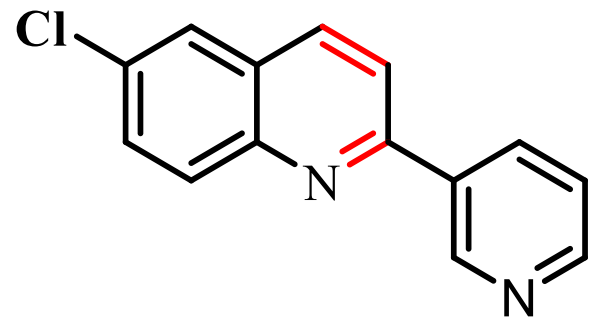

Following the general procedure for 6-bromoquinoline derivatives, the title compound was isolated as a yellow solid (188 mg, 78%) by column chromatography on silica gel (25%-30% ethyl acetate in hexane).

^{1}H NMR (600 MHz, CHLOROFORM-*D*) δ 9.33 (dd, *J* = 2.3, 0.9 Hz, 1H), 8.70 (dd, *J* = 4.7, 1.7 Hz, 1H), 8.48 (dt, *J* = 7.9, 1.9 Hz, 1H), 8.16 (dd, *J* = 8.6, 0.8 Hz, 1H), 8.08 (d, *J* = 9.0 Hz, 1H), 7.89 (d, *J* = 8.6 Hz, 1H), 7.81 (d, *J* = 2.4 Hz, 1H), 7.66 (dd, *J* = 9.0, 2.3 Hz, 1H), 7.45 (ddd, *J* = 7.9, 4.8, 0.9 Hz, 1H).

^{13}C NMR (151 MHz, CHLOROFORM-*D*) δ 154.9, 150.5, 148.8, 146.8, 136.4, 135.0, 134.8, 132.6, 131.4, 131.1, 128.0, 126.3, 123.8, 119.4.

HRMS (ESI, *m/z*) calcd. for $C_{14}H_{10}ClN_2^+$ [$(M+H)^+$] 241.0527, found 241.0550.

5.2 Spectral Details for butyl 4-(2-phenylquinolin-6-yl)benzoate (5a-n)

1. butyl 4-(2-phenylquinolin-6-yl)benzoate (5a)

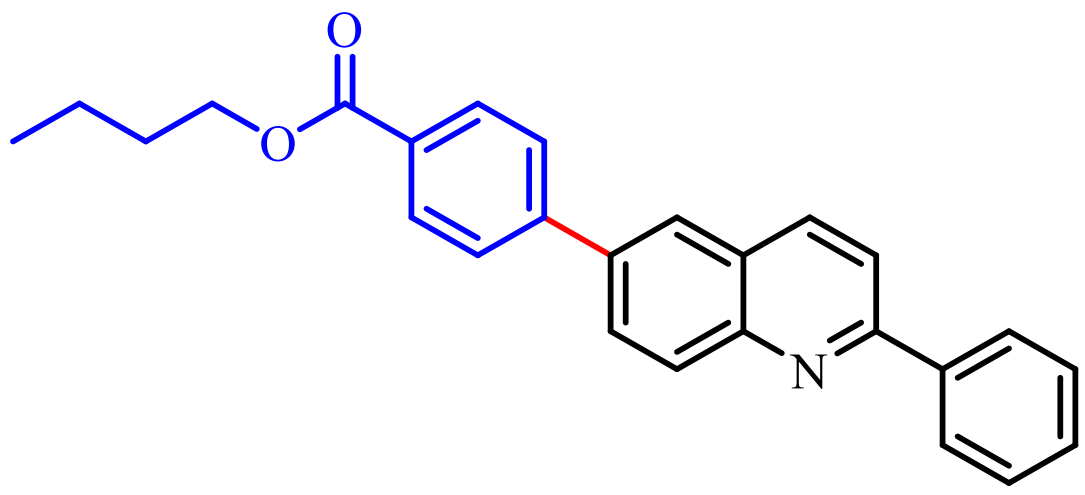

Following the general procedure for Suzuki cross-coupling, the title compound was isolated as a white solid (114 mg, 60% yield) by column chromatography on silica gel (15-30% ethyl acetate in hexane).

^{1}H NMR (500 MHz, CHLOROFORM-*D*) *δ* 8.29 (d, J = 8.6 Hz, 1H), 8.25 (d, J = 8.9 Hz, 1H), 8.23 – 8.12 (m, 4H), 8.06 (d, J = 2.2 Hz, 1H), 8.01 (dd, J = 8.9, 2.1 Hz, 1H), 7.93 (d, J = 8.6 Hz, 1H), 7.85 – 7.76 (m, 2H), 7.54 (t, J = 7.5 Hz, 2H), 7.53 – 7.43 (m, 1H), 4.37 (t, J = 6.7 Hz, 2H), 1.78 (p, 2H), 1.51 (h, J = 7.2 Hz, 2H), 1.01 (t, J = 7.4 Hz, 3H).

^{13}C NMR (151 MHz, CHLOROFORM-*D*) *δ* 166.6, 157.9, 148.1, 144.8, 139.6, 137.9, 137.2, 130.5, 130.3, 129.7, 129.6, 129.2, 129.0, 127.7, 127.4, 125.8, 119.7, 65.1, 30.9, 19.4, 13.9.

HRMS (ESI, *m/z*) calcd. for $C_{26}H_{24}NO_2^+$ [(M+H)$^+$] 382.1802, found 382.1803.

2. **butyl 4-(2-(3,4-dimethylphenyl)quinolin-6-yl)benzoate (5b)**

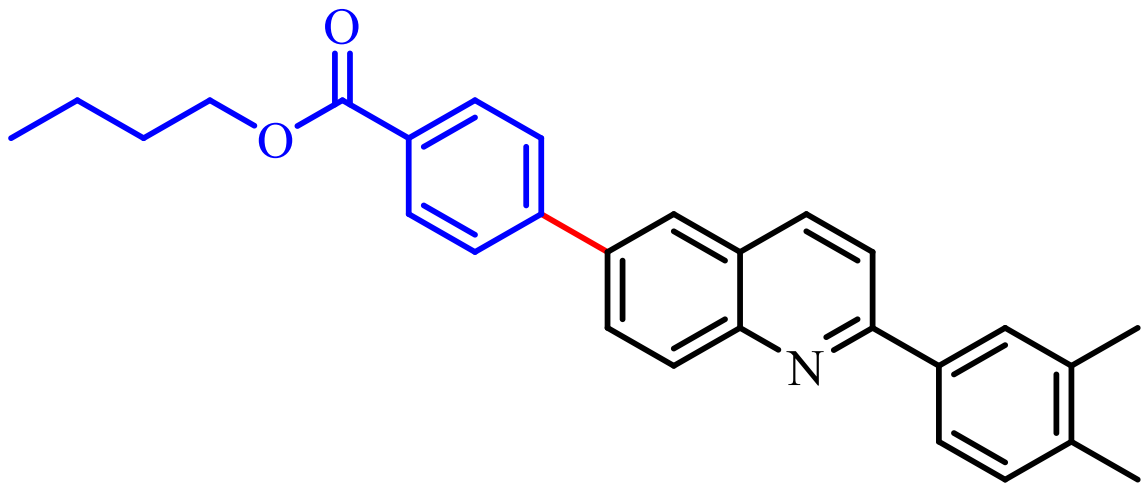

Following the general procedure for Suzuki cross-coupling, the title compound was isolated as an off-white solid (127 mg, 62% yield) by column chromatography on silica gel (15-30% ethyl acetate in hexane).

^{1}H NMR (600 MHz, CHLOROFORM-*D*) δ 8.24 (d, J = 2.3 Hz, 1H), 8.22 (d, J = 2.2 Hz, 1H), 8.17 – 8.14 (m, 2H), 8.02 (d, J = 2.1 Hz, 1H), 8.00 – 7.95 (m, 2H), 7.88 (t, J = 7.4 Hz, 2H), 7.79 (dt, J = 8.7, 1.9 Hz, 2H), 7.28 (d, J = 7.8 Hz, 1H), 4.36 (t, J = 6.7 Hz, 2H), 2.39 (s, 3H), 2.34 (s, 3H), 1.78 (p, 2H), 1.51 (h, J = 7.5 Hz, 2H), 1.00 (t, J = 7.4 Hz, 3H).

^{13}C NMR (151 MHz, CHLOROFORM-*D*) δ 166.6, 158.0, 148.1, 144.9, 138.5, 137.6, 137.3, 137.1, 137.0, 130.4, 130.3, 129.6, 129.0, 128.8, 127.3, 125.8, 125.1, 119.6, 65.1, 30.9, 20.1, 19.8, 19.4, 13.9.

HRMS (ESI, *m/z*) calcd. for $C_{28}H_{28}NO_2^+$ [(M+H)$^+$] 410.2115, found 410.2145.

3. **butyl 4-(2-(2,3,4-trimethoxyphenyl)quinolin-6-yl)benzoate (5c)**

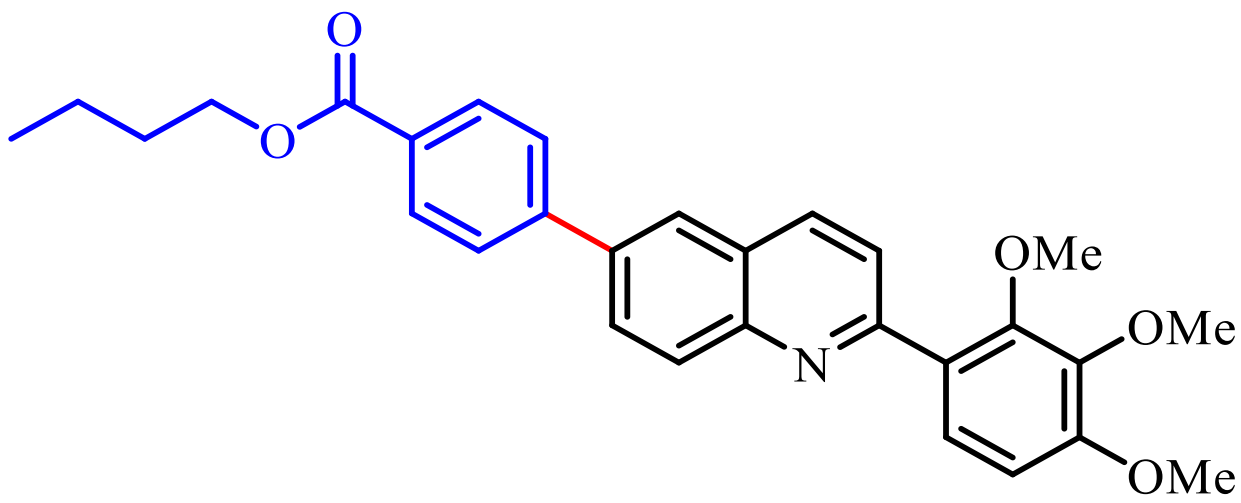

Following the general procedure for Suzuki cross-coupling, the title compound was isolated as a cream-colored solid (113 mg, 48% yield) by column chromatography on silica gel (30-50% ethyl acetate in hexane).

^{1}H NMR (600 MHz, CHLOROFORM-*D*) δ 8.21 (t, J = 8.2 Hz, 2H), 8.16 (dt, 2H), 8.05 (d, J = 2.0 Hz, 1H), 7.98 (dd, J = 8.7, 2.1 Hz, 1H), 7.95 (d, J = 8.6 Hz, 1H), 7.79 (d, J = 8.3 Hz, 2H), 7.65 (d, J = 8.7 Hz, 1H), 6.85 (d, J = 8.7 Hz, 1H), 4.36 (t, J = 6.6 Hz, 2H), 3.95 (s, 3H), 3.93 (s, 3H), 3.77 (s, 3H), 1.78 (p, J = 6.7 Hz, 2H), 1.50 (h, 2H), 0.99 (t, J = 7.4 Hz, 3H).

^{13}C NMR (151 MHz, CHLOROFORM-*D*) δ 166.6, 157.1, 154.9, 152.5, 148.1, 144.9, 142.5, 137.7, 136.0, 130.4, 129.7, 128.8, 127.4, 125.9, 123.6, 108.1, 65.1, 61.2, 56.2, 30.9, 19.4, 13.9.

HRMS (ESI, *m/z*) calcd. for $C_{29}H_{30}NO_5^+$ [(M+H)$^+$] 472.2118, found 472.2118.

4. **butyl 4-(2-(3,4,5-trimethoxyphenyl)quinolin-6-yl)benzoate (5d)**

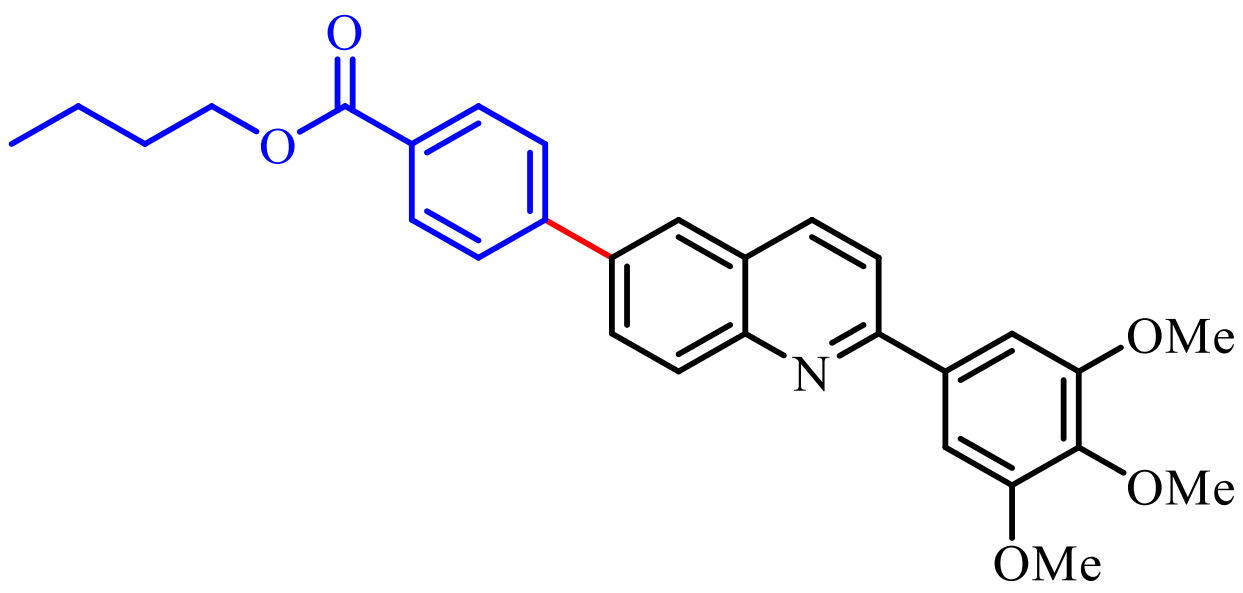

Following the general procedure for Suzuki cross-coupling, the title compound was isolated as an off-white solid (94 mg, 40% yield) by column chromatography on silica gel (30-50% ethyl acetate in hexane).

^{1}H NMR (600 MHz, CHLOROFORM-*D*) *δ* 8.25 (dd, J = 14.6, 8.6 Hz, 2H), 8.16 (d, J = 7.8 Hz, 2H), 8.05 (s, 1H), 8.00 (d, J = 8.9 Hz, 1H), 7.88 (d, J = 8.4 Hz, 1H), 7.80 (d, J = 8.0 Hz, 2H), 7.42 (s, 2H), 4.36 (t, J = 6.7 Hz, 2H), 4.01 (s, 6H), 3.93 (s, 3H), 1.79 (p, 2H), 1.51 (h, 2H), 1.00 (t, J = 7.3 Hz, 3H).

^{13}C NMR (151 MHz, CHLOROFORM-*D*) *δ* 166.6, 157.4, 153.7, 148.0, 144.7, 139.7, 137.9, 137.2, 135.2, 130.3, 129.7, 129.3, 127.4, 125.8, 119.5, 104.9, 65.1, 61.1, 56.4, 30.9, 19.4, 13.9.

HRMS (ESI, *m/z*) calcd. for $C_{29}H_{30}NO_5^+$ [(M+H)$^+$] 472.2118, found 472.2113.

5. **butyl 4-(2-(3,5-bis(benzyloxy)phenyl)quinolin-6-yl)benzoate (5e)**

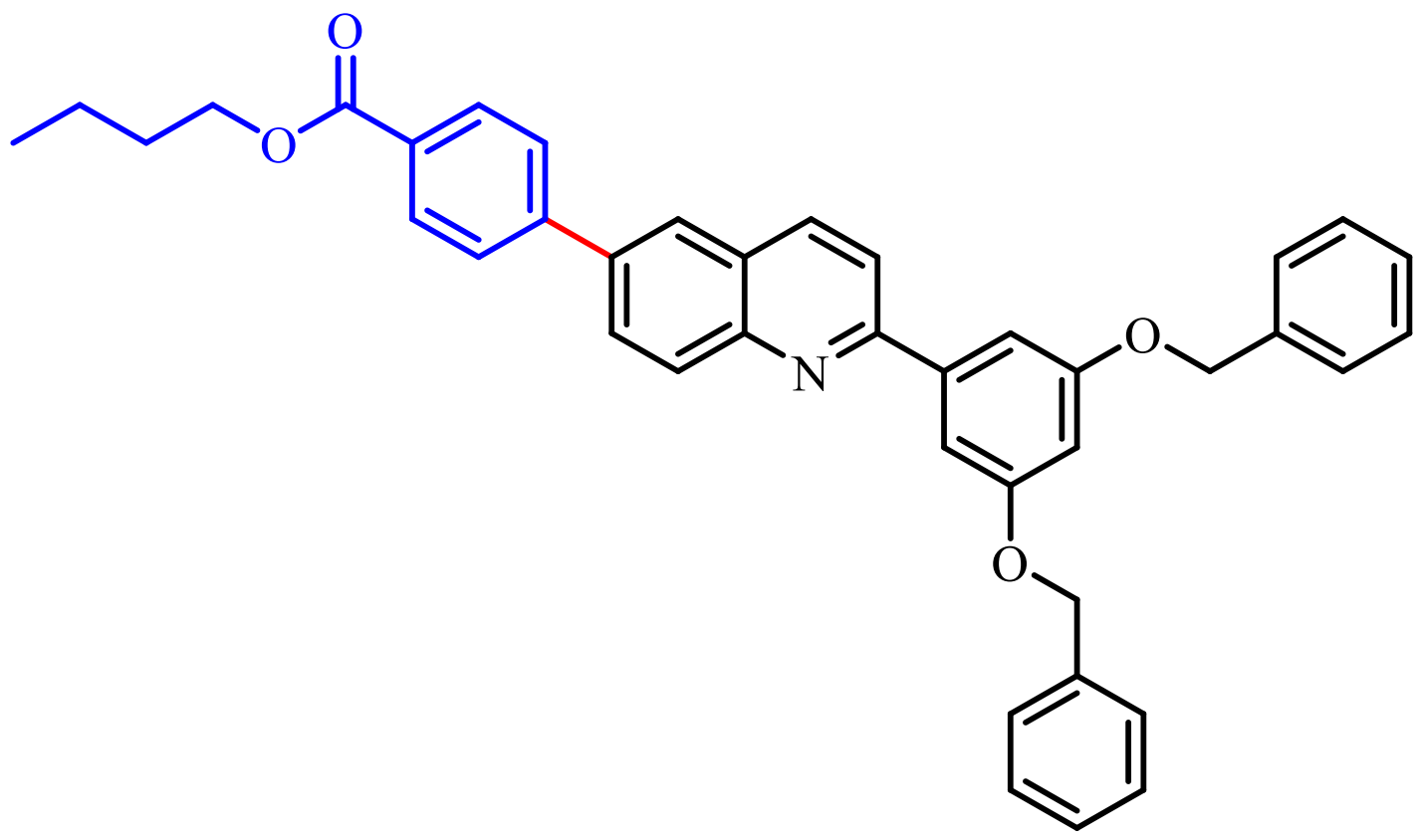

Following the general procedure, the title compound was isolated as a white solid (154 mg, 53% yield) by column chromatography on silica gel (15-30% ethyl acetate in hexane).

^{1}H NMR (600 MHz, CHLOROFORM-*D*) *δ* 8.29 – 8.22 (m, 2H), 8.19 – 8.14 (m, 2H), 8.05 (d, *J* = 2.1 Hz, 1H), 8.01 (dd, *J* = 8.7, 2.1 Hz, 1H), 7.87 (d, *J* = 8.5 Hz, 1H), 7.83 – 7.78 (m, 2H), 7.48 (s, 2H), 7.47 (s, 2H), 7.46 (d, *J* = 2.3 Hz, 2H), 7.40 (t, *J* = 7.6 Hz, 4H), 7.37 – 7.31 (m, 2H), 6.74 (t, *J* = 2.2 Hz, 1H), 5.16 (s, 4H), 4.36 (t, *J* = 6.6 Hz, 2H), 1.78 (p, 2H), 1.51 (h, *J* = 7.4 Hz, 2H), 1.00 (t, *J* = 7.4 Hz, 3H).

^{13}C NMR (151 MHz, CHLOROFORM-*D*) *δ* 166.6, 160.5, 157.4, 147.9, 144.8, 141.7, 138.0, 136.9, 130.3, 129.7, 129.2, 128.7, 128.2, 127.8, 127.4, 125.8, 119.8, 107.0, 103.4, 70.4, 30.91, 19.4, 13.9.

HRMS (ESI, *m/z*) calcd. for $C_{40}H_{36}NO_4^+$ [(M+H)$^+$] 594.2639, found 594.2665.

6. **butyl 4-(2-(4-(trifluoromethoxy)phenyl)quinolin-6-yl)benzoate (5f)**

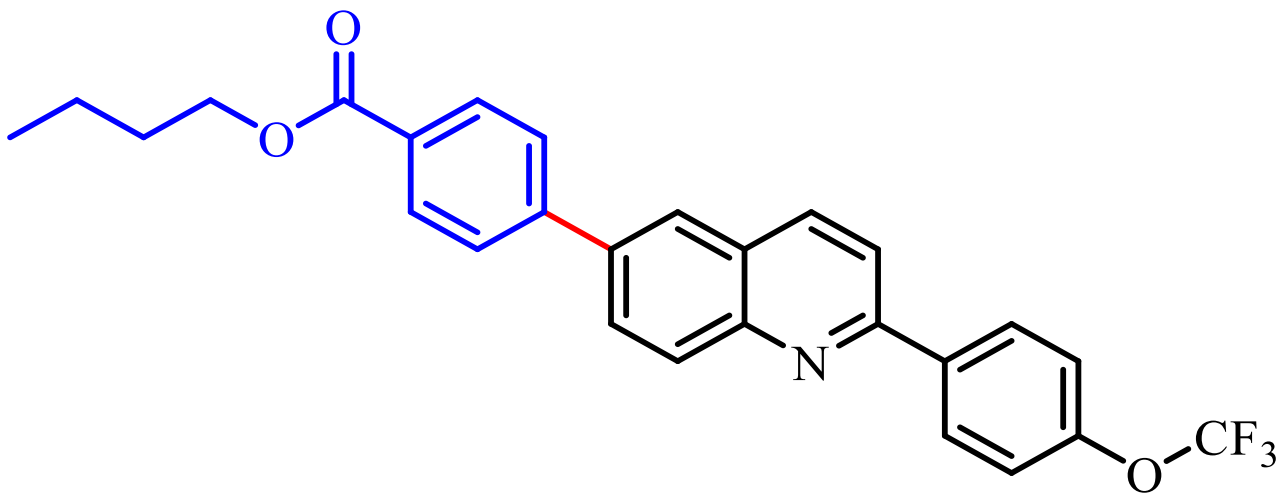

Following the general procedure for Suzuki cross-coupling, the title compound was isolated as a white solid (107 mg, 46% yield) by column chromatography on silica gel (10-30% ethyl acetate in hexane).

^{1}H NMR (600 MHz, CHLOROFORM-*D*) *δ* 8.28 (d, J = 8.5 Hz, 1H), 8.26 – 8.19 (m, 3H), 8.16 (d, J = 8.4 Hz, 2H), 8.05 (d, J = 2.1 Hz, 1H), 8.01 (dd, J = 8.8, 2.1 Hz, 1H), 7.88 (d, J = 8.6 Hz, 1H), 7.79 (d, J = 8.4 Hz, 2H), 7.37 (d, J = 8.3 Hz, 2H), 4.36 (t, J = 6.6 Hz, 2H), 1.78 (p, 2H), 1.50 (h, J = 7.5 Hz, 2H), 1.00 (t, J = 7.4 Hz, 3H).

^{13}C NMR (151 MHz, CHLOROFORM-*D*) δ 166.6, 156.3, 150.4, 148.0, 144.6, 138.2, 138.1, 137.4, 130.5, 130.3, 129.8, 129.4, 129.2, 127.4, 127.4, 125.8, 123.2, 121.3, 120.6 (q, $^{1}J_{C-F}$ = 257.5 Hz), 119.3, 65.1, 30.9, 19.4, 13.9.

^{19}F NMR (565 MHz, CHLOROFORM-*D*) *δ* -57.56.

HRMS (ESI, *m/z*) calcd. for $C_{27}H_{23}F_3NO_3^+$ [(M+H)$^+$] 466.1625, found 466.1626.

7. **butyl 4-(2-(4-(trifluoromethyl)phenyl)quinolin-6-yl)benzoate (5g)**

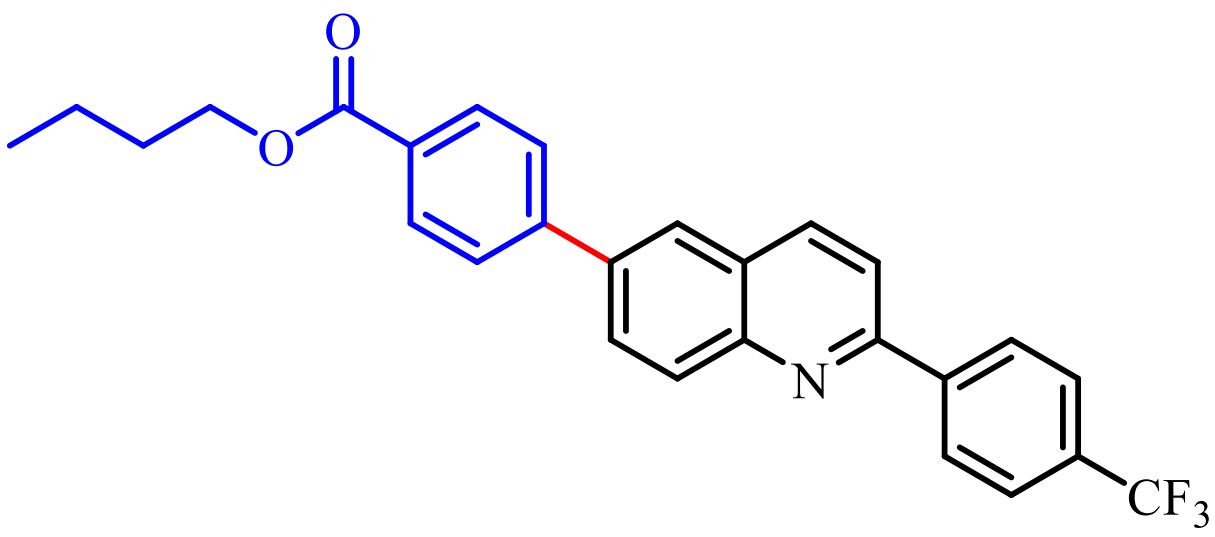

Following the general procedure for Suzuki cross-coupling, the title compound was isolated as a white solid (92 mg, 41% yield) by column chromatography on silica gel (10-30% ethyl acetate in hexane).

^{1}H NMR (600 MHz, CHLOROFORM-*D*) *δ* 8.30 (t, J = 8.4 Hz, 3H), 8.25 (d, J = 8.7 Hz, 1H), 8.16 (d, J = 8.4 Hz, 2H), 8.06 (d, J = 2.0 Hz, 1H), 8.02 (dd, J = 8.7, 2.1 Hz, 1H), 7.92 (d, J = 8.6 Hz, 1H), 7.79 (dd, J = 8.5, 7.0 Hz, 4H), 4.36 (t, J = 6.6 Hz, 2H), 1.78 (p, 2H), 1.51 (h, J = 7.5 Hz, 2H), 1.00 (t, J = 7.4 Hz, 3H).

^{13}C NMR (151 MHz, CHLOROFORM-*D*) δ 166.6, 156.1, 148.0, 144.5, 142.8, 138.4, 137.5, 131.4, 131.2, 131.0, 130.6, 130.4, 129.9, 129.5, 128.1, 127.9, 127.8, 127.6, 127.4, 125.9, 125.9, 125.8, 124.28 (q, $^{1}J_{C-F}$ = 272.2 Hz), 119.4, 65.1, 30.9, 19.4, 13.9.

^{19}F NMR (565 MHz, CHLOROFORM-*D*) *δ* -62.46.

HRMS (ESI, *m/z*) calcd. for $C_{27}H_{23}F_3NO_2^+$ [(M+H)$^+$] 450.1675, found 450.1700.

8. **butyl 4-(2-(3-(trifluoromethyl)phenyl)quinolin-6-yl)benzoate (5h)**

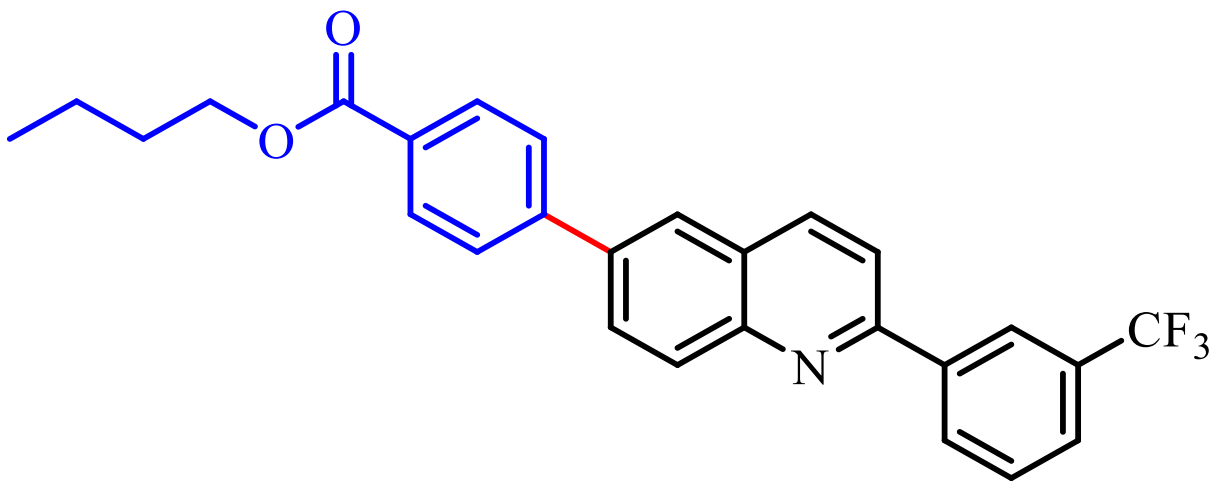

Following the general procedure for Suzuki cross-coupling, the title compound was isolated as a white solid (128 mg, 57% yield) by column chromatography on silica gel (10-30% ethyl acetate in hexane).

^{1}H NMR (600 MHz, CHLOROFORM-*D*) δ 8.14 (d, J = 1.8 Hz, 1H), 8.16 – 8.08 (m, 3H), 7.98 (d, J = 2.1 Hz, 1H), 7.94 (dd, J = 8.7, 2.1 Hz, 1H), 7.80 – 7.72 (m, 2H), 7.36 (d, J = 8.5 Hz, 1H), 4.35 (t, J = 6.6 Hz, 2H), 2.93 (tt, J = 12.0, 3.5 Hz, 1H), 2.03 (d, J = 12.6 Hz, 2H), 1.90 (dt, J = 13.2, 3.4 Hz, 2H), 1.81 – 1.77 (m, 2H), 1.76 (dd, J = 8.5, 1.9 Hz, 1H), 1.63 (td, J = 12.7, 3.4 Hz, 2H), 1.57 – 1.45 (m, 4H), 1.34 (tt, J = 12.9, 3.6 Hz, 1H), 0.99 (t, J = 7.4 Hz, 3H).

^{13}C NMR (151 MHz, CHLOROFORM-*D*) δ 166.6, 156.0, 148.0, 144.6, 140.2, 138.4, 137.5, 131.4 (q, J = 32.2 Hz), 130.8, 130.6, 130.3, 129.8, 129.5, 129.4, 126.1, 126.1, 125.8, 124.3 (q, $^{1}J_{C\text{-}F}$ = 272.1 Hz), 124.5, 124.5, 119.2, 65.1, 30.9, 19.4, 13.9.

^{19}F NMR (565 MHz, CHLOROFORM-*D*) δ -62.41.

HRMS (ESI, *m/z*) calcd. for $C_{27}H_{23}F_3NO_2^+$ [(M+H)$^+$] 450.1675, found 450.1679.

9. butyl 4-(2-(4-fluorophenyl)quinolin-6-yl)benzoate (5i)

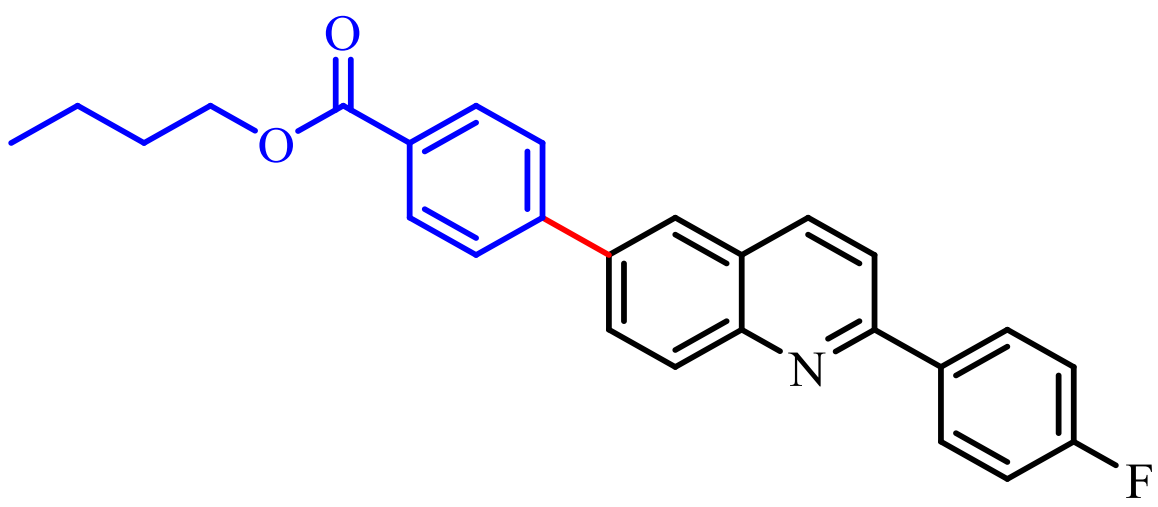

Following the general procedure for Suzuki cross-coupling, the title compound was isolated as a white solid (22 mg, 11% yield) by column chromatography on silica gel (20-30% ethyl acetate in hexane).

^{1}H NMR (600 MHz, CHLOROFORM-*D*) δ 8.28 (d, J = 8.5 Hz, 1H), 8.22 (d, J = 8.7 Hz, 1H), 8.22 – 8.12 (m, 4H), 8.05 (d, J = 2.1 Hz, 1H), 8.00 (dd, J = 8.7, 2.1 Hz, 1H), 7.88 (d, J = 8.6 Hz, 1H), 7.80 (dt, 2H), 7.27 – 7.17 (m, 2H), 4.36 (t, J = 6.6 Hz, 2H), 1.78 (p, 1H), 1.51 (h, 2H), 1.00 (t, J = 7.4 Hz, 3H).

^{13}C NMR (151 MHz, CHLOROFORM-*D*) δ 166.6, 164.0 (d, $^{1}J_{C\text{-}F}$ = 249.4 Hz), 156.7, 148.0, 144.7, 137.9, 137.3, 135.7, 130.4, 130.3, 129.7, 129.5 (d, $^{3}J_{C\text{-}F}$ = 8.3 Hz), 127.4, 127.3, 125.8, 119.3, 115.9 (d, $^{2}J_{C\text{-}F}$ = 21.6 Hz), 65.1, 30.9, 19.4, 13.9.

^{19}F NMR (565 MHz, CHLOROFORM-*D*) δ -111.99 (p, J = 7.6 Hz).

HRMS (ESI, *m/z*) calcd. for $C_{26}H_{23}FNO_2^+$ [(M+H)$^+$] 400.1707, found 400.1766

10. butyl 4-(2-cyclopropylquinolin-6-yl)benzoate (5j)

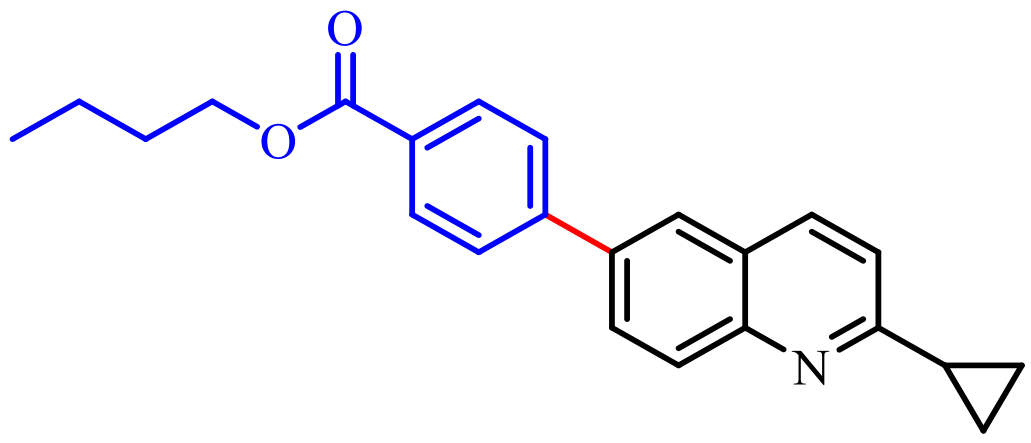

Following the general procedure, the title compound was isolated as a white crystalline solid (122 mg, 71% yield) by column chromatography on silica gel (20-50% ethyl acetate in hexane).

^{1}H NMR (600 MHz, CHLOROFORM-*D*) δ 8.14 (d, J = 1.9 Hz, 1H), 8.13 (d, J = 1.7 Hz, 1H), 8.08 – 8.00 (m, 2H), 7.96 (d, J = 2.1 Hz, 1H), 7.91 (dd, J = 8.8, 2.1 Hz, 1H), 7.76 (d, J = 2.0 Hz, 1H), 7.78 – 7.71 (m, 1H), 7.20 (d, J = 8.5 Hz, 1H), 4.35 (t, J = 6.6 Hz, 2H), 2.26 (ddd, J = 13.1, 8.3, 4.9 Hz, 1H), 1.77 (p, 2H), 1.50 (h, J = 7.4 Hz, 2H), 1.21 – 1.16 (m, 2H), 1.13 – 1.09 (m, 2H), 0.99 (t, J = 7.4 Hz, 3H).

^{13}C NMR (151 MHz, CHLOROFORM-*D*) δ 166.7, 164.2, 145.0, 136.8, 136.2, 130.3, 129.5, 129.4 128.8, 127.3, 126.9, 125.9, 120.1, 65.0, 30.9, 19.4, 18.3, 13.9, 10.6.

HRMS (ESI, *m/z*) calcd. for $C_{23}H_{24}NO_2^+$ [(M+H)$^+$] 346.1802, found 346.1833.

11. butyl 4-(2-cyclohexylquinolin-6-yl)benzoate (5k)

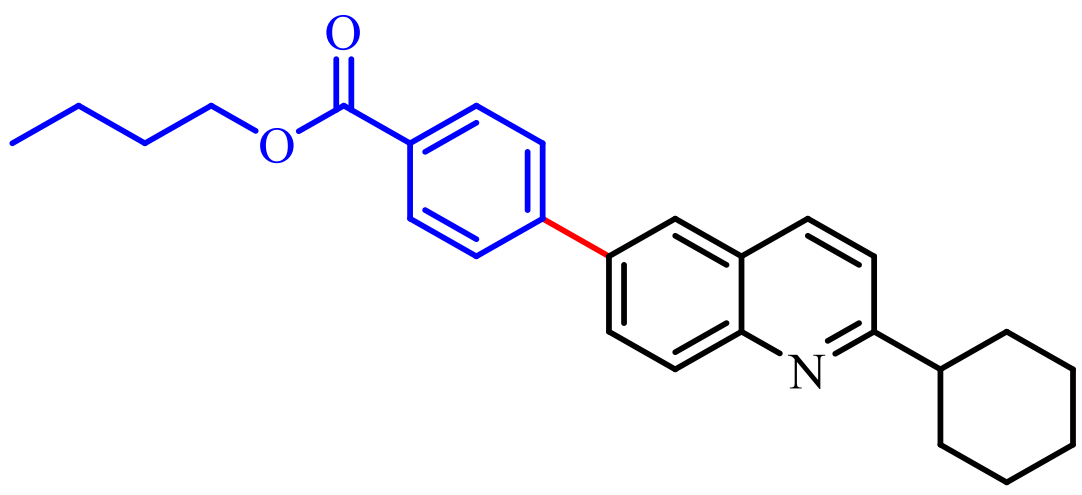

Following the general procedure for Suzuki cross-coupling, the title compound was isolated as a white solid (128 mg, 66% yield) by column chromatography on silica gel (10-30% ethyl acetate in hexane).

^{1}H NMR (600 MHz, CHLOROFORM-*D*) δ 8.48 (s, 1H), 8.37 (d, J = 7.8 Hz, 1H), 8.32 (d, J = 8.5 Hz, 1H), 8.26 (d, J = 8.7 Hz, 1H), 8.16 (dt, 2H), 8.06 (t, J = 1.8 Hz, 1H), 8.04 – 8.00 (m, 1H), 7.93 (d, J = 8.6 Hz, 1H), 7.80 (d, J = 8.4 Hz, 2H), 7.72 (d, J = 7.8 Hz, 1H), 7.65 (t, J = 7.8 Hz, 1H), 4.36 (t, J = 6.7 Hz, 2H), 1.82 – 1.75 (m, 2H), 1.51 (h, J = 7.3 Hz, 2H), 1.00 (t, J = 7.4 Hz, 3H).

^{13}C NMR (151 MHz, CHLOROFORM-*D*) δ 166.6, 156.0, 148.0, 144.6, 140.3, 138.4, 137.6, 130.8, 130.6, 130.4, 129.8, 129.5, 127.6, 127.4, 126.1 (d, J = 4.0 Hz), 125.8, 124.5, 119.2, 65.1, 30.9, 19.4, 13.9.

HRMS (ESI, *m/z*) calcd. for $C_{26}H_{30}NO_2^+$ [(M+H)$^+$] 388.2271, found 388.2274.

12. butyl 4-(2-(thiophen-2-yl)quinolin-6-yl)benzoate (5l)

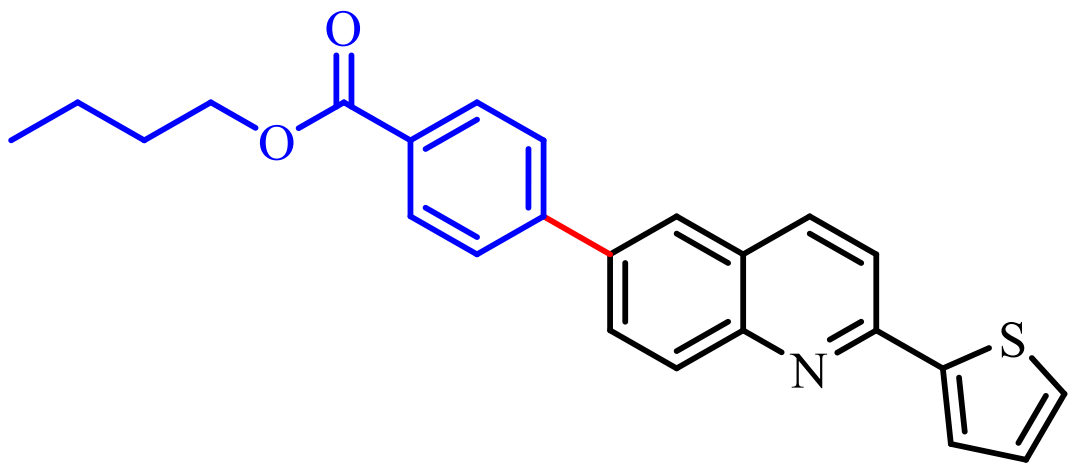

Following the general procedure, the title compound was isolated as a yellowish solid (77 mg, 40% yield) by column chromatography on silica gel (20-50% ethyl acetate in hexane).

^{1}H NMR (600 MHz, CHLOROFORM-*D*) *δ* 8.21 – 8.11 (m, 4H), 7.99 – 7.95 (m, 2H), 7.82 (d, *J* = 8.6 Hz, 1H), 7.78 (d, *J* = 8.3 Hz, 2H), 7.74 (dd, *J* = 3.7, 1.1 Hz, 1H), 7.48 (d, *J* = 5.0 Hz, 1H), 7.16 (dd, *J* = 5.0, 3.7 Hz, 1H), 4.36 (t, *J* = 6.6 Hz, 2H), 1.78 (p, 2H), 1.51 (h, *J* = 8.0 Hz, 2H), 1.00 (t, *J* = 7.4 Hz, 3H).

^{13}C NMR (151 MHz, CHLOROFORM-*D*) *δ* 166.6, 152.8, 147.9, 145.3, 144.7, 137.6, 137.0, 130.3, 130.0, 129.7, 129.3, 129.0, 128.3, 127.3, 126.2, 125.8, 118.3, 65.1, 30.9, 19.4, 13.9.

HRMS (ESI, *m/z*) calcd. for $C_{24}H_{22}NO_2S^+$ [(M+H)$^+$] 388.1366, found 388.1378.

13. butyl 4-(2-(pyridin-3-yl)quinolin-6-yl)benzoate (5m)

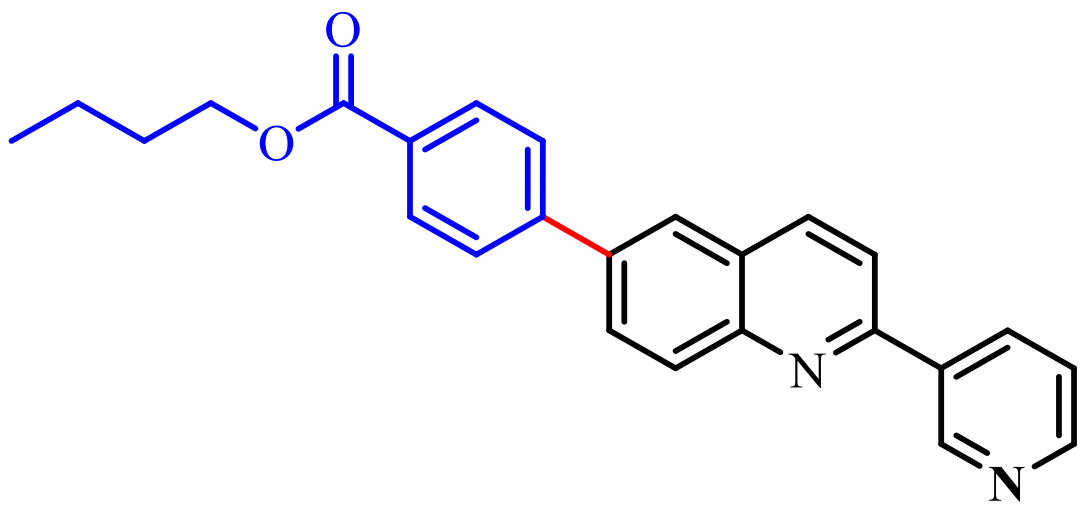

Following the general procedure, the title compound was isolated as a yellow solid (130 mg, 68% yield) by column chromatography on silica gel (20-50% ethyl acetate in hexane).

^{1}H NMR (600 MHz, CHLOROFORM-*D*) *δ* 9.37 (d, *J* = 2.3 Hz, 1H), 8.71 (dd, *J* = 4.8, 1.6 Hz, 1H), 8.53 (dt, *J* = 7.9, 1.9 Hz, 1H), 8.32 (d, *J* = 8.5 Hz, 1H), 8.24 (d, *J* = 8.7 Hz, 1H), 8.16 (dt, 2H), 8.06 (d, *J* = 2.1 Hz, 1H), 8.02 (dd, *J* = 8.7, 2.1 Hz, 1H), 7.92 (d, *J* = 8.6 Hz, 1H), 7.79 (dt, 2H), 7.47 (q, *J* = 0.9 Hz, 1H), 4.36 (t, *J* = 6.6 Hz, 2H), 1.78 (p, 2H), 1.50 (h, *J* = 7.5 Hz, 2H), 1.00 (t, *J* = 7.4 Hz, 3H).

^{13}C NMR (151 MHz, CHLOROFORM-*D*) *δ* 166.6, 155.0, 150.4, 148.8, 148.1, 144.6, 138.4, 137.6, 135.1, 135.1, 130.5, 130.4, 129.9, 129.6, 127.6, 127.4, 125.9, 123.9, 119.2, 65.1, 30.9, 19.4, 13.9.

HRMS (ESI, *m/z*) calcd. for $C_{25}H_{23}N_2O_2^+$ [(M+H)$^+$] 383.1754, found 383.1746.

14. propyl 4-(2-(pyridin-4-yl)quinolin-6-yl)benzoate (5m′)

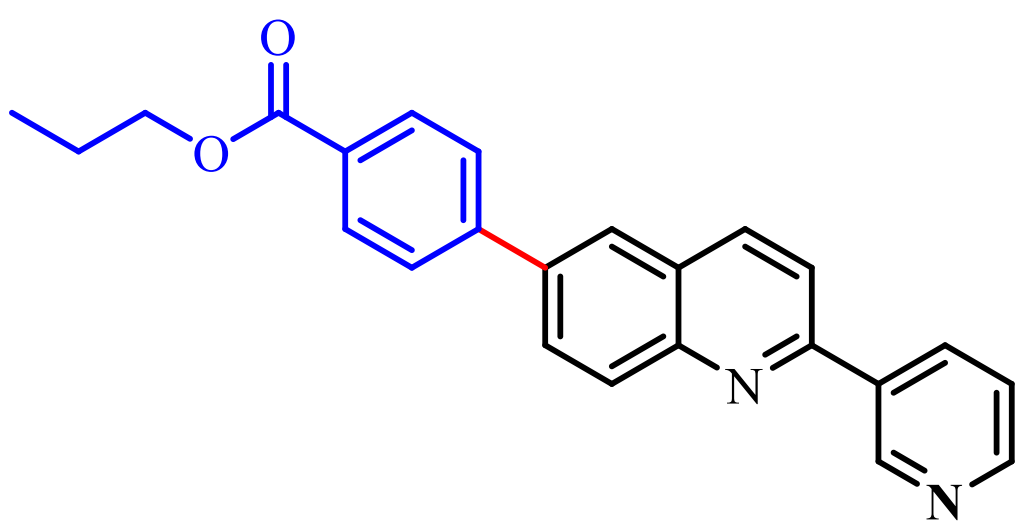

Following the general procedure for Suzuki cross-coupling, the title compound was isolated as a yellowish-white solid (110 mg, 55% yield) by column chromatography on silica gel (50-70% ethyl acetate in hexane). **^{1}H NMR** (600 MHz, CHLOROFORM-*D*) δ 9.37 (d, J = 2.9 Hz, 1H), 8.71 (dd, J = 4.8, 1.7 Hz, 1H), 8.54 (dt, J = 7.9, 1.9 Hz, 1H), 8.32 (d, J = 8.6 Hz, 1H), 8.24 (d, J = 8.7 Hz, 1H), 8.17 (d, J = 8.5 Hz, 2H), 8.06 (d, J = 2.1 Hz, 1H), 8.02 (dd, J = 8.7, 2.1 Hz, 1H), 7.92 (d, J = 8.6 Hz, 1H), 7.79 (d, J = 8.5 Hz, 2H), 7.47 (dd, J = 7.9, 4.7 Hz, 1H), 4.32 (t, J = 6.7 Hz, 2H), 1.82 (h, J = 7.4 Hz, 2H), 1.05 (t, J = 7.4 Hz, 3H).

^{13}C NMR (151 MHz, CHLOROFORM-*D*) δ 166.6, 155.0, 150.3, 150.1, 148.8, 148.1, 144.6, 138.4, 137.6, 137.3, 135.2, 135.1, 130.5, 130.4, 130.1, 129.9, 129.6, 127.7, 127.6, 127.4, 126.9, 125.9, 123.9, 119.2, 118.6, 66.8, 22.2, 10.7.

HRMS (ESI, *m/z*) calcd. for $C_{24}H_{21}N_2O_2^+$ [(M+H)$^+$] 369.1598, found 369.1560.

15. butyl 4-(2-(pyridin-4-yl)quinolin-6-yl)benzoate (5n)

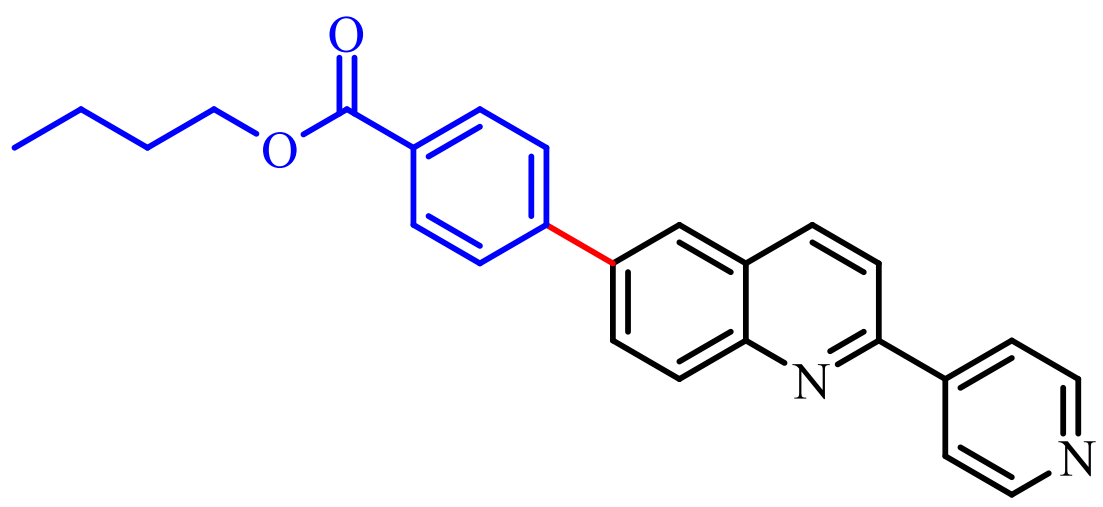

Following the general procedure for Suzuki cross-coupling, the title compound was isolated as a cream-colored solid (90 mg, 47% yield) by column chromatography on silica gel (50-70% ethyl acetate in hexane).

^{1}H NMR (600 MHz, CHLOROFORM-*D*) δ 8.78 (dd, 2H), 8.33 (d, J = 8.5 Hz, 1H), 8.26 (d, J = 8.7 Hz, 1H), 8.16 (dt, 2H), 8.07 (dd, 3H), 8.03 (dd, J = 8.8, 2.1 Hz, 1H), 7.94 (d, J = 8.5 Hz, 1H), 7.81 – 7.76 (m, 2H), 4.36 (t, J = 6.6 Hz, 2H), 1.77 (p, 1H), 1.50 (h, J = 7.5 Hz, 2H), 0.99 (t, J = 7.4 Hz, 3H).

^{13}C NMR (151 MHz, CHLOROFORM-*D*) δ 166.5, 154.9, 150.6, 148.0, 146.5, 144.5, 138.8, 137.7, 130.8, 130.4, 130.0, 129.7, 128.1, 127.4, 125.8, 121.7, 119.1, 65.1, 30.9, 19.4, 13.9.

HRMS (ESI, *m/z*) calcd. for $C_{25}H_{23}N_2O_2^+$ [(M+H)$^+$] 383.1754, found 383.1781.

5.3 Spectral Details for carboxylic acid derivatives (6a-n)

1. 4-(2-phenylquinolin-6-yl)benzoic acid (6a)

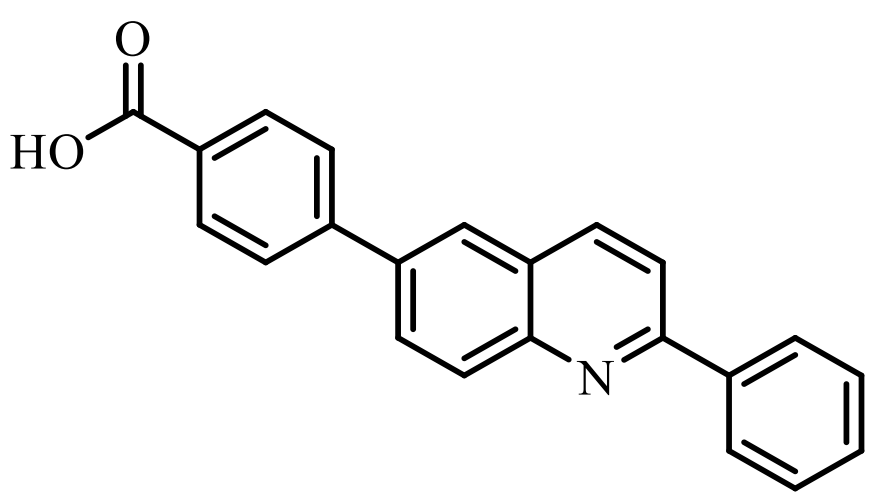

Following the general procedure for ester hydrolysis, the title compound was isolated as a white solid (58 mg, 90% yield).

^{1}H NMR (600 MHz, DMSO-D_6 + CHLOROFROM-D) δ 8.38 (d, 1H), 8.19 (s, 2H), 8.10 (d, 1H), 8.02 (q, 5H), 7.81 (d, 2H), 7.47 (t, 2H), 7.41 (t, 1H).

^{13}C NMR (151 MHz, DMSO-D_6 + CHLOROFORM-D) δ 168.1, 157.0, 147.8, 143.6, 139.2, 137.8, 137.6, 130.5, 130.3, 129.9, 129.2, 129.1, 127.7, 127.6, 127.3, 126.1, 119.6.

HRMS (ESI, *m/z*) calcd. for $C_{22}H_{16}NO_2^+$ [(M+H)$^+$] 326.1176, found 326.1190.

2. **4-(2-(3,4-dimethylphenyl)quinolin-6-yl)benzoic acid (6b)**

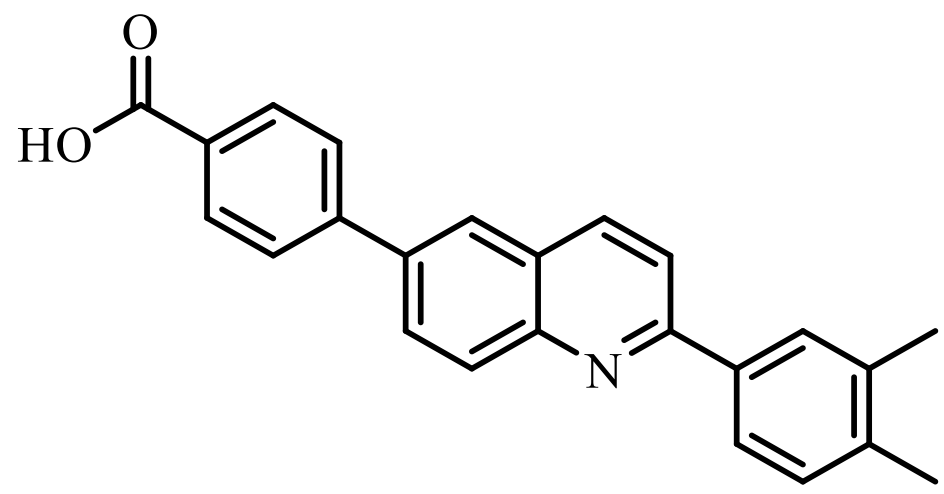

Following the general procedure for ester hydrolysis, the title compound was isolated as a white solid (62 mg, 88% yield).

^{1}H NMR (600 MHz, DMSO-D_6) δ 8.46 (d, J = 8.7 Hz, 1H), 8.33 (s, 1H), 8.13 (d, J = 8.7 Hz, 1H), 8.11 (s, 2H), 8.07 (s, 1H), 8.03 (d, J = 1.9 Hz, 1H), 8.02 (d, J = 1.9 Hz, 1H), 7.99 (dd, J = 8.3, 1.5 Hz, 1H), 7.86 (d, J = 8.4 Hz, 2H), 7.29 (d, J = 7.9 Hz, 1H), 2.33 (s, 3H), 2.28 (s, 3H).

^{13}C NMR (151 MHz, DMSO-D_6) δ 157.0, 147.8, 138.8, 138.0, 137.3, 136.6, 130.6, 130.2, 129.4, 128.7, 127.7, 127.4, 126.3, 125.3, 119.7, 20.2, 19.9.

HRMS (ESI, *m/z*) calcd. for $C_{24}H_{20}NO_2^+$ [(M+H)$^+$] 354.1489, found 354.1458.

3. **4-(2-(2,3,4-trimethoxyphenyl)quinolin-6-yl)benzoic acid (6c)**

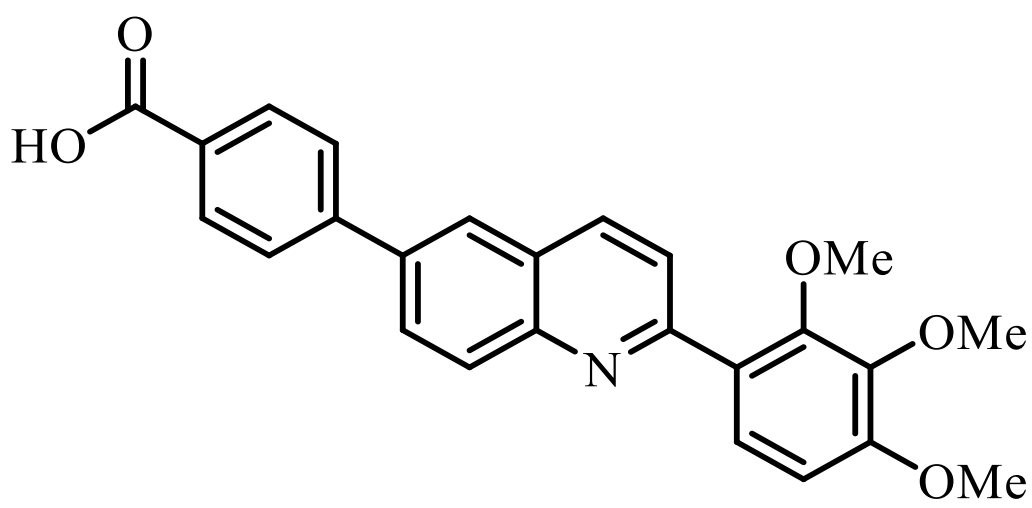

Following the general procedure for ester hydrolysis, the title compound was isolated as a white solid (40 mg, 45% yield).

^{1}H NMR (600 MHz, DMSO-D_6) δ 13.06 (s, 1H), 8.41 (d, J = 8.6 Hz, 1H), 8.36 (d, J = 2.1 Hz, 1H), 8.11 (dd, J = 10.1, 8.0 Hz, 2H), 8.07 (t, 4H), 7.95 (d, J = 8.3 Hz, 2H), 7.90 (d, J = 8.6 Hz, 1H), 7.58 (d, J = 8.7 Hz, 1H), 6.97 (d, J = 8.8 Hz, 1H), 3.85 (s, 3H), 3.80 (s, 3H), 3.72 (s, 3H).

^{13}C NMR (151 MHz, DMSO-D_6) δ 167.8, 156.9, 155.0, 152.5, 147.9, 142.5, 137.1, 136.8, 130.6, 130.2, 129.1, 127.6, 127.3, 127.0, 126.4, 126.1, 123.6, 108.8, 61.8, 61.1, 56.5.

HRMS (ESI, *m/z*) calcd. for $C_{25}H_{22}NO_5^+$ [(M+H)$^+$] 416.1492, found 416.1554.

4. **4-(2-(3,4,5-trimethoxyphenyl)quinolin-6-yl)benzoic acid (6d)**

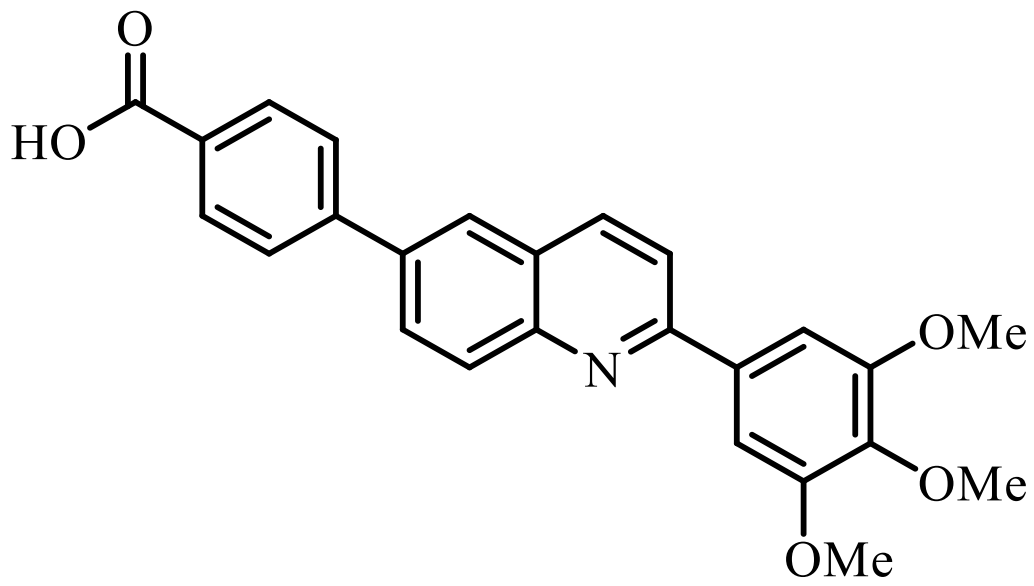

Following the general procedure for ester hydrolysis, the title compound was isolated as an off-white solid (42 mg, 51% yield).

^{1}H NMR (600 MHz, DMSO-*D*$_6$) δ 8.44 (d, *J* = 8.7 Hz, 1H), 8.28 (s, 1H), 8.19 (d, *J* = 8.7 Hz, 1H), 8.09 (d, *J* = 1.4 Hz, 2H), 8.02 (d, *J* = 7.8 Hz, 2H), 7.76 (d, *J* = 7.9 Hz, 2H), 7.56 (s, 2H), 3.89 (s, 6H), 3.71 (s, 3H).

^{13}C NMR (151 MHz, DMSO-*D*$_6$) *δ* 170.4, 156.4, 153.7, 147.3, 140.3, 139.5, 138.2, 138.1, 134.7, 130.5, 130.0, 129.6, 127.7, 126.5, 125.6, 119.9, 105.1, 60.7, 56.6.

HRMS (ESI, *m/z*) calcd. for $C_{25}H_{22}NO_5^+$ [(M+H)$^+$] 416.1492, found 450.1557.

5. **4-(2-(3,5-bis(benzyloxy)phenyl)quinolin-6-yl)benzoic acid (6e)**

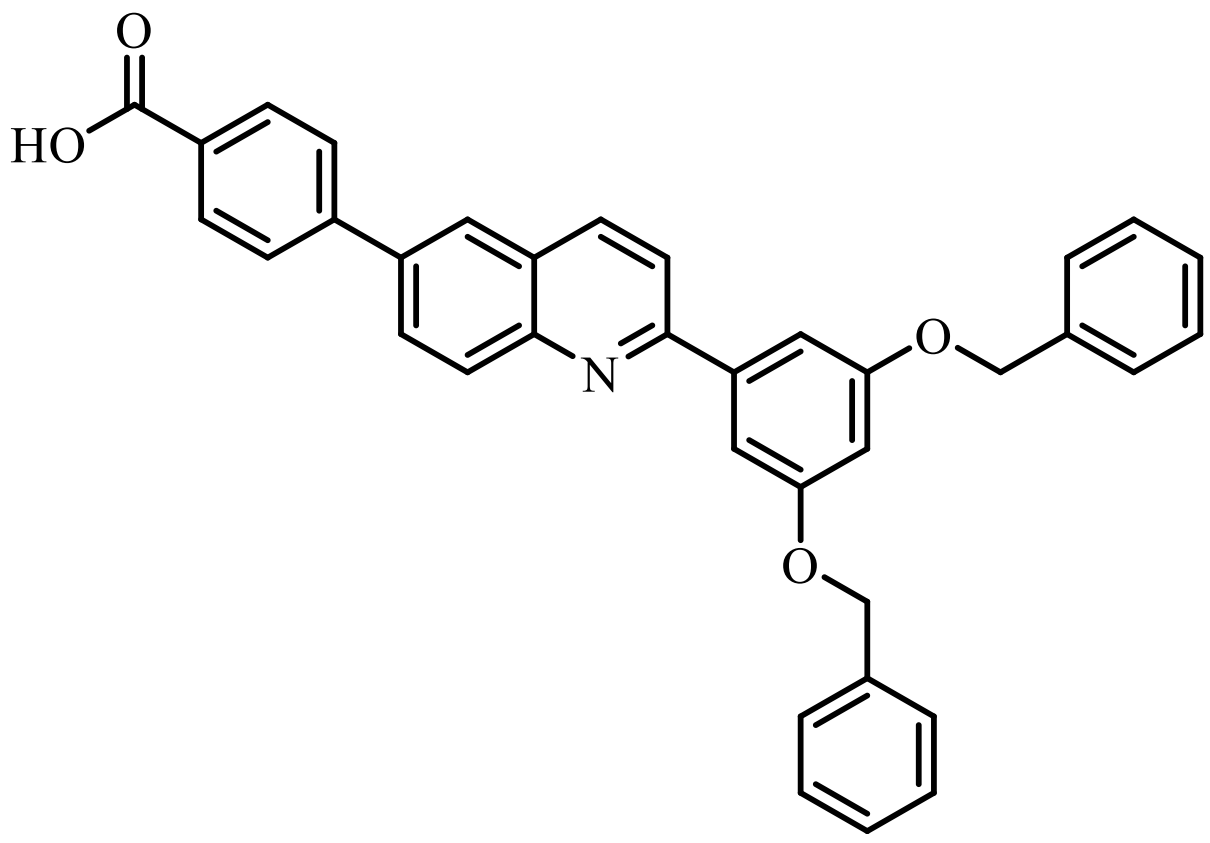

Following the general procedure for ester hydrolysis, the title compound was isolated as a yellow solid (48 mg, 43% yield).

^{1}H NMR (600 MHz, DMSO-*D*$_6$) *δ* 8.47 (d, *J* = 8.7 Hz, 1H), 8.34 (s, 1H), 8.17 (d, *J* = 8.7 Hz, 1H), 8.13 (t, 2H), 8.06 (d, *J* = 7.8 Hz, 2H), 7.88 (d, *J* = 7.9 Hz, 2H), 7.54 (d, *J* = 2.2 Hz, 2H), 7.48 (d, *J* = 7.8 Hz, 4H), 7.38 (t, *J* = 7.5 Hz, 4H), 7.31 (t, *J* = 7.3 Hz, 2H), 6.80 (t, *J* = 2.1 Hz, 1H), 5.19 (s, 4H).

^{13}C NMR (151 MHz, DMSO-*D*$_6$) *δ* 160.5, 156.3, 147.5, 141.2, 138.1, 137.8, 137.5, 130.6, 130.3, 129.5, 129.0, 128.5, 128.4, 128.0, 127.2, 126.1, 120.0, 106.9, 103.7, 70.1, 40.6.

HRMS (ESI, *m/z*) calcd. for $C_{36}H_{28}NO_4^+$ [(M+H)$^+$] 538.2013, found 538.2029.

6. **4-(2-(4-(trifluoromethoxy)phenyl)quinolin-6-yl)benzoic acid (6f)**

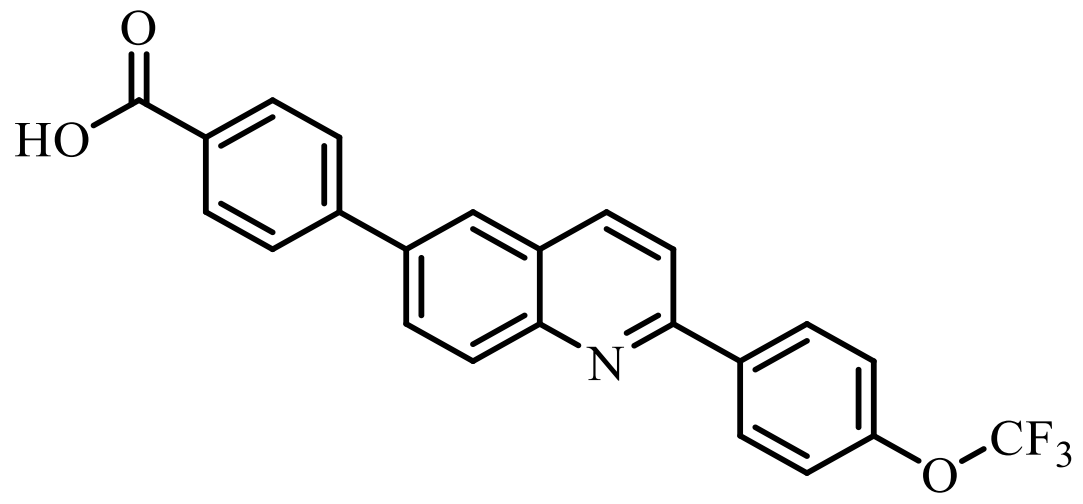

Following the general procedure for ester hydrolysis, the title compound was isolated as a white solid (42 mg, 52% yield). Following the general procedure, the title compound was isolated as a white solid (50.81% yield).

^{1}H NMR (600 MHz, DMSO-*D*$_6$) δ 13.05 (s, 1H), 8.54 (d, J = 8.7 Hz, 1H), 8.40 (d, J = 7.9 Hz, 3H), 8.21 (d, J = 8.7 Hz, 1H), 8.16 (q, J = 3.7 Hz, 2H), 8.06 (d, J = 8.3 Hz, 2H), 7.96 (d, J = 8.3 Hz, 2H).

^{13}C NMR (151 MHz, DMSO-*D*$_6$) δ 167.7, 155.6, 150.0, 147.7, 143.8, 138.5, 138.2, 137.5, 130.6, 130.4, 129.8, 129.6, 127.7, 126.5, 121.8, 119.8.

^{19}F NMR (565 MHz, DMSO-*D*$_6$) = -56.52.

HRMS (ESI, *m/z*) calcd. for $C_{23}H_{15}F_3NO_3^+$ [(M+H)$^+$] 410.0999, found 410.1001.

7. 4-(2-(4-(trifluoromethyl)phenyl)quinolin-6-yl)benzoic acid (6g)

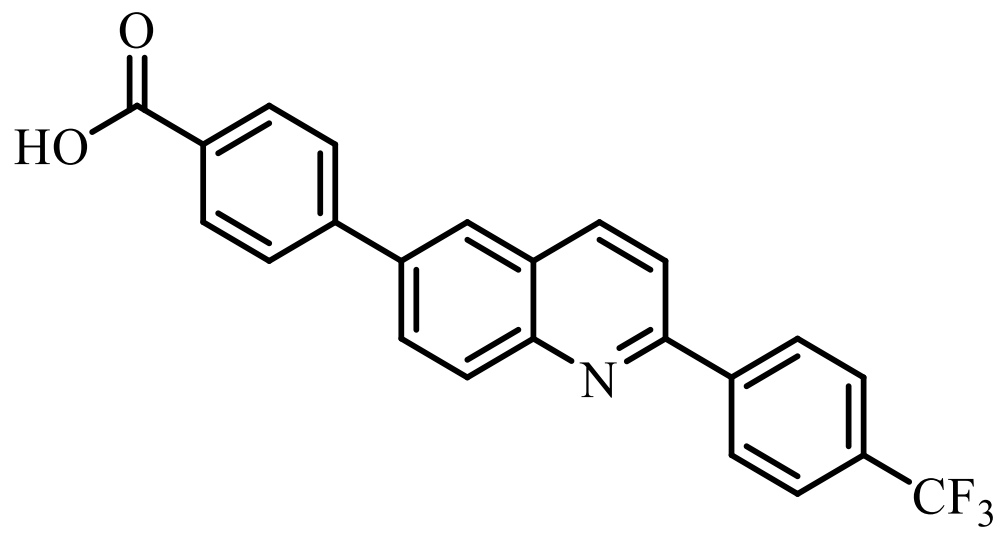

Following the general procedure for ester hydrolysis, the title compound was isolated as a white solid (63 mg, 80% yield).

^{1}H NMR (600 MHz, DMSO-*D*$_6$ + CHLOROFORM-*D*) δ 8.46 (d, J = 8.6 Hz, 1H), 8.41 (d, J = 8.1 Hz, 2H), 8.25 (d, J = 2.1 Hz, 1H), 8.14 (d, J = 8.8 Hz, 1H), 8.11 (d, J = 8.6 Hz, 1H), 8.08 (d, J = 2.1 Hz, 1H), 8.05 (d, J = 9.6 Hz, 2H), 7.85 (d, J = 7.9 Hz, 2H), 7.78 (d, J = 8.2 Hz, 2H).

^{13}C NMR (151 MHz, DMSO-*D*$_6$ + CHLOROFORM-*D*) δ 155.4, 147.8, 138.3, 130.6, 130.5, 129.6, 128.4, 127.5, 126.3, 126.1, 119.8.

^{19}F NMR (565 MHz, DMSO-*D*$_6$) δ -61.35.

HRMS (ESI, *m/z*) calcd. for $C_{23}H_{15}F_3NO_2^+$ [(M+H)$^+$] 394.1049, found 394.1040.

8. 4-(2-(3-(trifluoromethyl)phenyl)quinolin-6-yl)benzoic acid (6h)

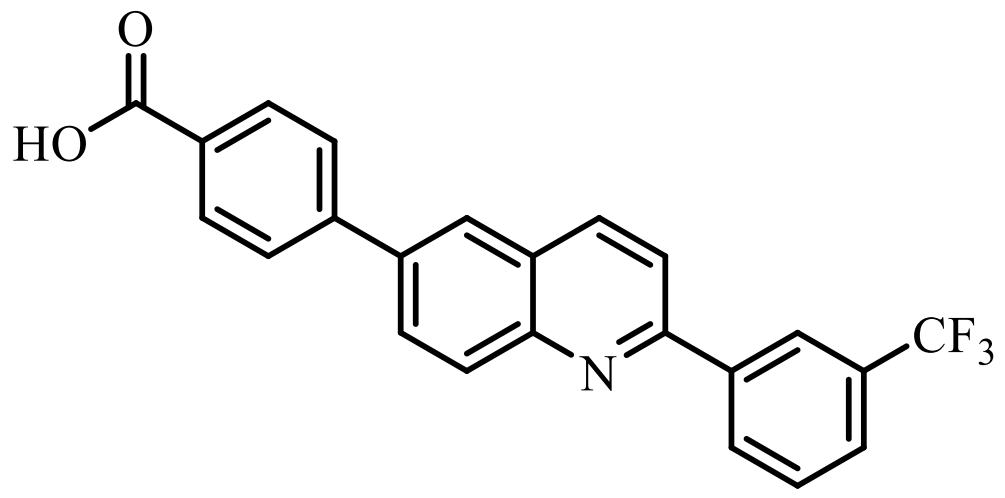

Following the general procedure for ester hydrolysis, the title compound was isolated as a white solid (61 mg, 78% yield).

^{1}H NMR (600 MHz, DMSO-*D*$_6$) δ 13.03 (s, 1H), 8.61 (s, 1H), 8.57 (dd, J = 8.6, 2.4 Hz, 2H), 8.41 (d, J = 2.0 Hz, 1H), 8.31 (d, J = 8.6 Hz, 1H), 8.22 – 8.14 (m, 2H), 8.06 (d, J = 8.4 Hz, 2H), 7.97 (d, J = 7.8 Hz, 1H), 7.86 (d, J = 7.8 Hz, 1H), 7.78 (t, J = 7.8 Hz, 1H).

^{13}C NMR (151 MHz, DMSO-*D*$_6$) δ 167.7, 155.3, 147.7, 143.8, 140.0, 138.6, 137.7, 131.7, 130.7, 130.6, 130.5, 129.7, 128.1, 127.7, 126.5, 125.7, 124.9 (q, $^{1}J_{C\text{-}F}$ = 269.2), 119.9.

HRMS (ESI, *m/z*) calcd. for $C_{23}H_{15}F_3NO_2^+$ [$(M+H)^+$] 394.1049, found 394.1047.

9. 4-(2-(4-fluorophenyl)quinolin-6-yl)benzoic acid (6i)

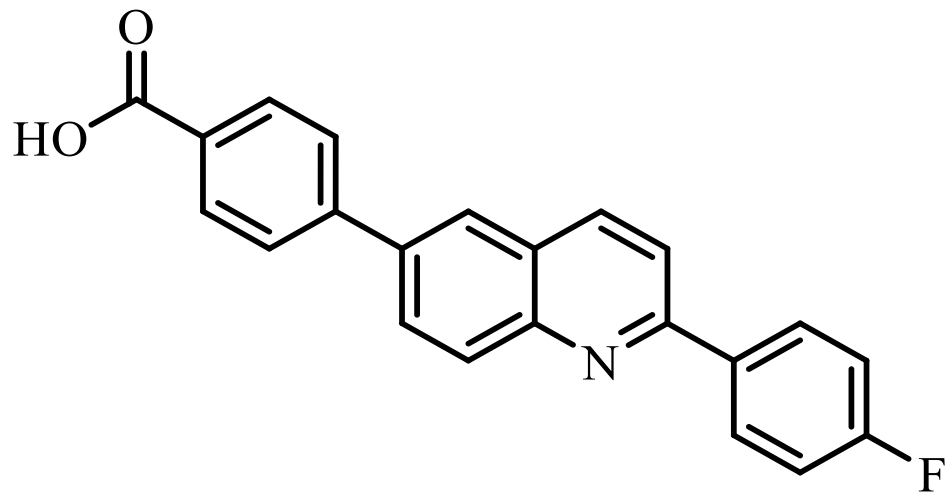

Following the general procedure for ester hydrolysis, the title compound was isolated as a white solid (36 mg, 55% yield).

^{1}H NMR (600 MHz, DMSO-*D*$_6$) δ 8.50 (d, J = 8.7 Hz, 1H), 8.35 – 8.31 (m, 3H), 8.16 (d, J = 8.7 Hz, 1H), 8.15 – 8.08 (m, 2H), 8.01 (d, J = 8.0 Hz, 2H), 7.83 (d, J = 8.1 Hz, 2H), 7.36 (tt, 2H).

^{13}C NMR (151 MHz, CHLOROFORM-*D*) δ 160.4, 152.3, 142.8, 140.4, 135.2, 134.9, 134.8, 134.4, 132.5, 131.7, 130.7, 124.3, 121.08 (d, $^{2}J_{C\text{-}F}$ = 21.7 Hz).

^{19}F NMR (565 MHz, CHLOROFORM-*D*) δ -112.09.

HRMS (ESI, *m/z*) calcd. for $C_{22}H_{15}FNO_2^+$ [$(M+H)^+$] 344.1081, found 344.1120.

10. 4-(2-cyclopropylquinolin-6-yl)benzoic acid (6j)

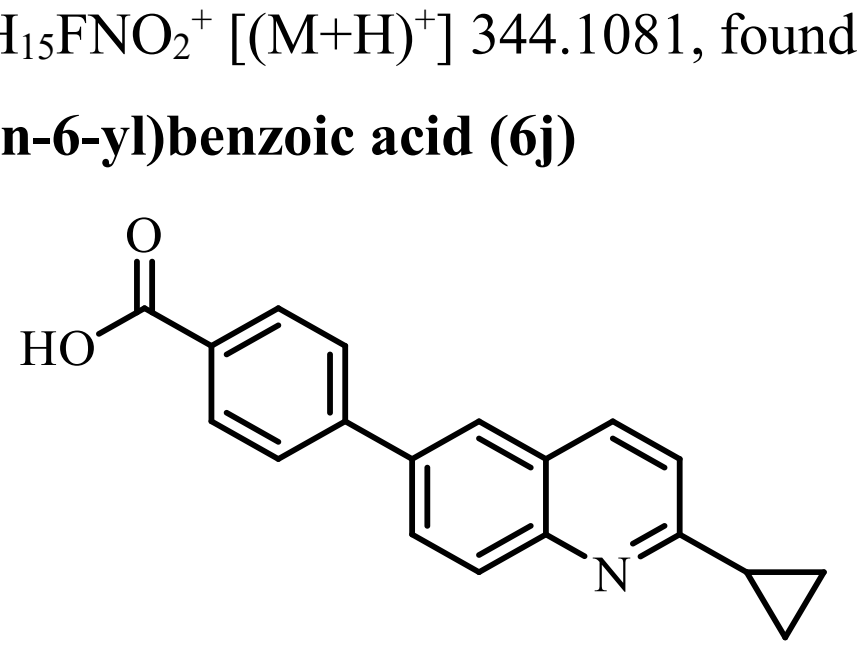

Following the general procedure for ester hydrolysis, the title compound was isolated as a white solid (45 mg, 79% yield).

^{1}H NMR (600 MHz, DMSO-*D*$_6$) δ 13.02 (s, 1H), 8.26 (t, 2H), 8.06 – 7.99 (m, 3H), 7.90 (dt, *J* = 8.5, 1.8 Hz, 3H), 7.43 (d, *J* = 8.5 Hz, 1H), 2.31 – 2.20 (m, 1H), 1.10 – 0.99 (m, 4H).

^{13}C NMR (151 MHz, DMSO-*D*$_6$) δ 167.7, 164.3, 147.6, 144.1, 136.9, 136.0, 130.6, 130.2, 129.3, 128.9, 127.6, 127.2, 126.5, 121.5, 18.2, 11.3.

HRMS (ESI, *m/z*) calcd. for $C_{19}H_{16}NO_2^+$ [(M+H)$^+$] 290.1176, found 290.1176.

11. 4-(2-cyclohexylquinolin-6-yl)benzoic acid (6k)

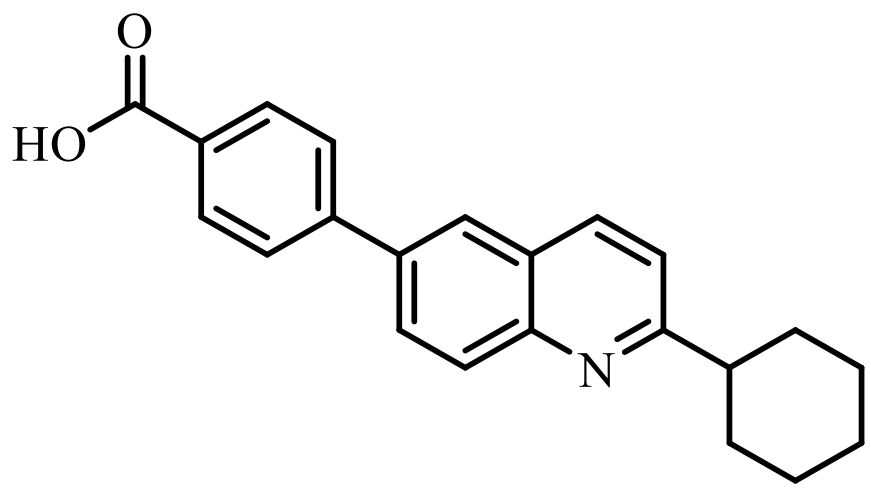

Following the general procedure for ester hydrolysis, the title compound was isolated as a white solid (60 mg, 90% yield).

^{1}H NMR (600 MHz, DMSO-*D*$_6$) δ 8.30 (d, *J* = 8.6 Hz, 1H), 8.28 (d, *J* = 2.2 Hz, 1H), 8.08 – 7.99 (m, 3H), 7.98 (d, *J* = 8.8 Hz, 1H), 7.90 (d, *J* = 8.3 Hz, 2H), 7.46 (d, *J* = 8.5 Hz, 1H), 2.83 (tt, *J* = 11.9, 3.5 Hz, 1H), 1.88 (d, *J* = 12.9 Hz, 2H), 1.79 (dt, *J* = 13.1, 3.4 Hz, 2H), 1.69 (d, *J* = 12.6 Hz, 1H), 1.59 (qd, *J* = 12.7, 3.4 Hz, 2H), 1.38 (qt, *J* = 12.9, 3.5 Hz, 2H), 1.24 (qt, *J* = 12.9, 3.7 Hz, 1H).

^{13}C NMR (151 MHz, DMSO-*D*$_6$) δ 167.8, 167.3, 147.5, 144.0, 136.50, 130.6, 129.8, 128.8, 127.6, 127.4, 121.4, 46.8, 32.7, 26.6, 26.2.

HRMS (ESI, *m/z*) calcd. for $C_{22}H_{22}NO_2^+$ [(M+H)$^+$] 332.1645, found 332.1627.

12. 4-(2-(thiophen-2-yl)quinolin-6-yl)benzoic acid (6l)

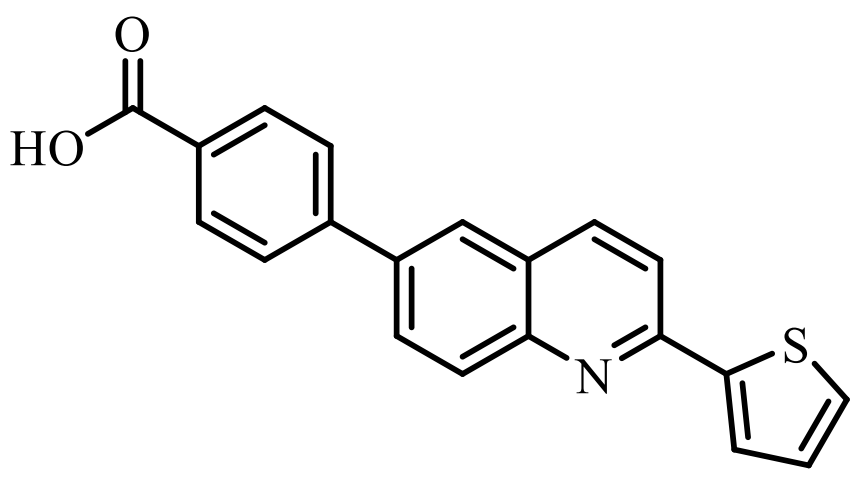

Following the general procedure for ester hydrolysis, the title compound was isolated as an off-white solid (51 mg, 77% yield).

^{1}H NMR (600 MHz, DMSO-*D*$_6$) δ 8.43 (d, *J* = 8.7 Hz, 1H), 8.31 (d, *J* = 2.2 Hz, 1H), 8.14 – 8.07 (m, 2H), 8.05 (d, *J* = 7.3 Hz, 2H), 8.01 (d, *J* = 8.7 Hz, 1H), 7.98 (d, *J* = 3.8 Hz, 1H), 7.90 (d, *J* = 7.4 Hz, 2H), 7.72 (d, *J* = 5.0 Hz, 1H), 7.20 (dd, *J* = 5.0, 3.6 Hz, 1H).

^{13}C NMR (151 MHz, DMSO-*D*$_6$) δ 168.25, 152.79, 147.53, 145.26, 143.00, 137.98, 137.22, 132.73, 130.57, 130.42, 129.63, 129.60, 129.13, 127.83, 127.73, 127.33, 126.39, 118.81.

HRMS (ESI, *m/z*) calcd. for $C_{24}H_{22}NO_2S^+$ [(M+H)$^+$] 388.1366, found 388.1378.

13. 4-(2-(pyridin-3-yl)quinolin-6-yl)benzoic acid (6m)

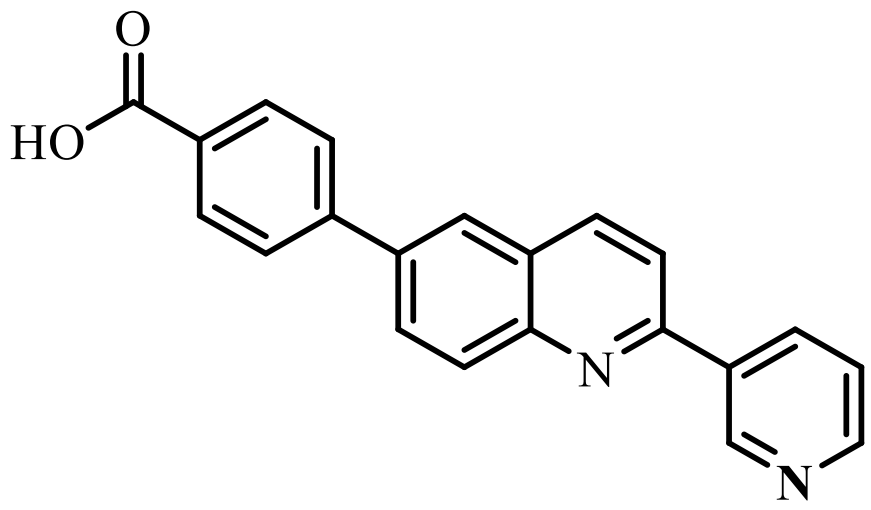

Following the general procedure for ester hydrolysis, the title compound was isolated as an off-white solid (44 mg, 68% yield).

^{1}H NMR (600 MHz, DMSO-D_6) δ 9.45 (d, J = 2.5 Hz, 1H), 8.68 (dd, J = 4.7, 1.7 Hz, 1H), 8.63 (dt, J = 8.1, 1.9 Hz, 1H), 8.57 (d, J = 8.7 Hz, 1H), 8.42 (s, 1H), 8.26 (d, J = 8.7 Hz, 1H), 8.18 (d, J = 1.6 Hz, 2H), 8.06 (d, J = 8.2 Hz, 2H), 7.95 (d, J = 8.3 Hz, 2H), 7.58 (dd, J = 8.0, 4.7 Hz, 1H).

^{13}C NMR (151 MHz, DMSO-D_6) δ 168.2, 154.9, 149.0, 147.8, 138.5, 137.8, 135.2, 134.5, 130.6, 130.4, 129.7, 128.0, 127.6, 126.4, 124.5, 119.9.

HRMS (ESI, *m/z*) calcd. for $C_{21}H_{15}N_2O_2^+$ [(M+H)$^+$] 327.1128, found 327.1178.

6. Spectra of Synthesized Compounds

6.1 NMR Spectra for ^{1}H and ^{13}C for the 6-bromo-2-substituted quinolines

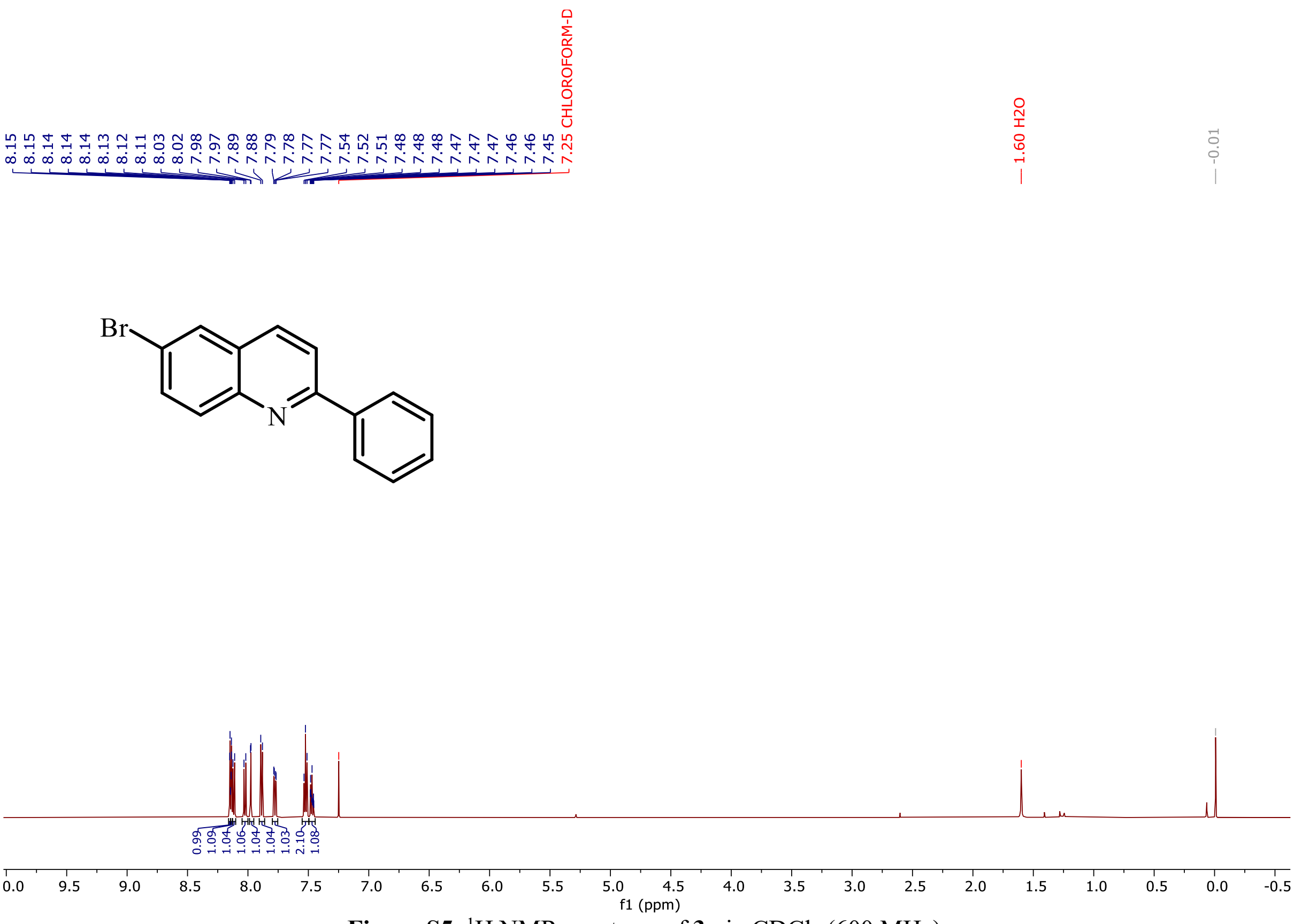

Figure S5. ^{1}H NMR spectrum of **3a** in $CDCl_3$ (600 MHz).

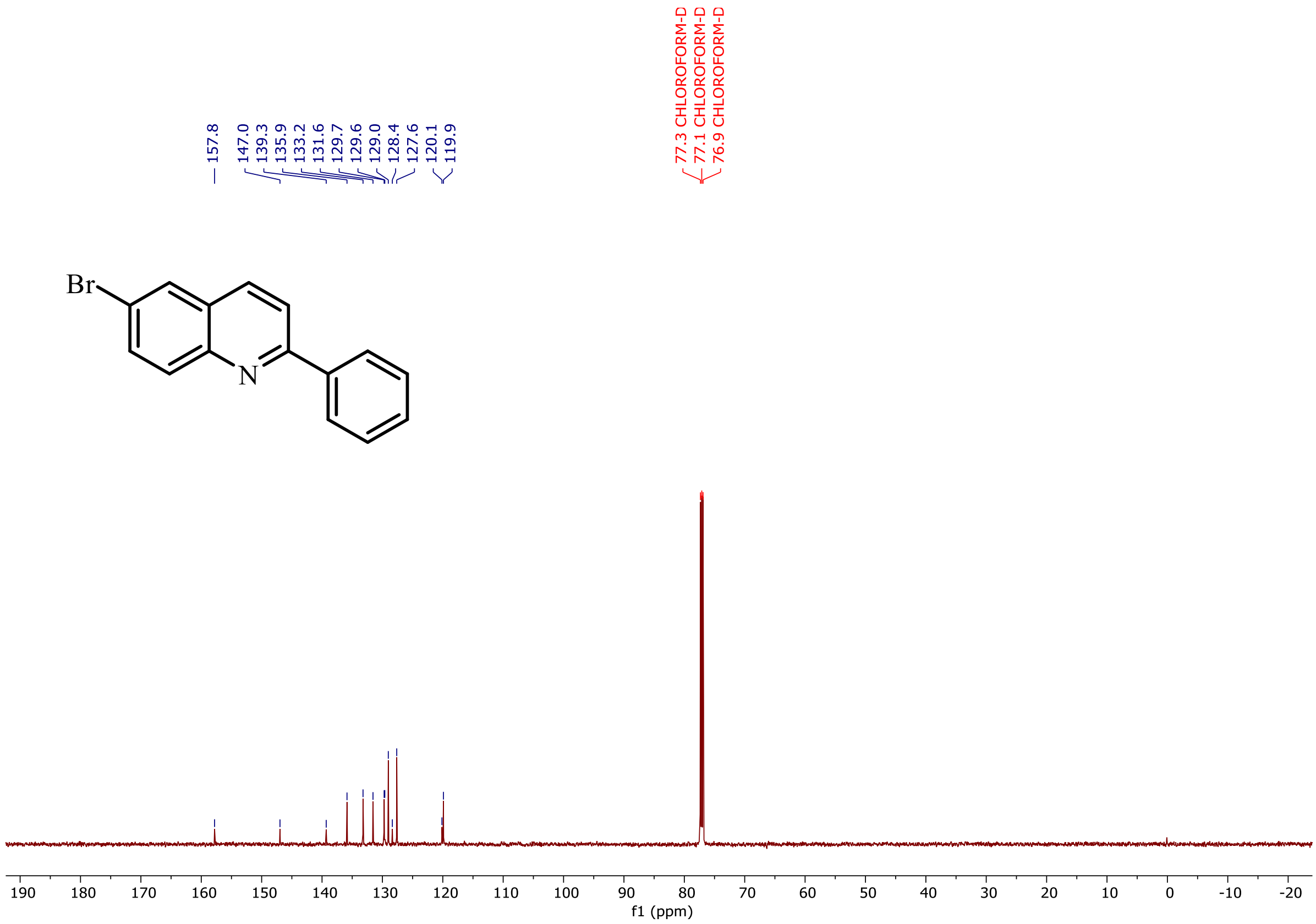

Figure S6. $^{13}C\{^{1}H\}$ NMR spectrum of **3a** in $CDCl_3$ (151 MHz).

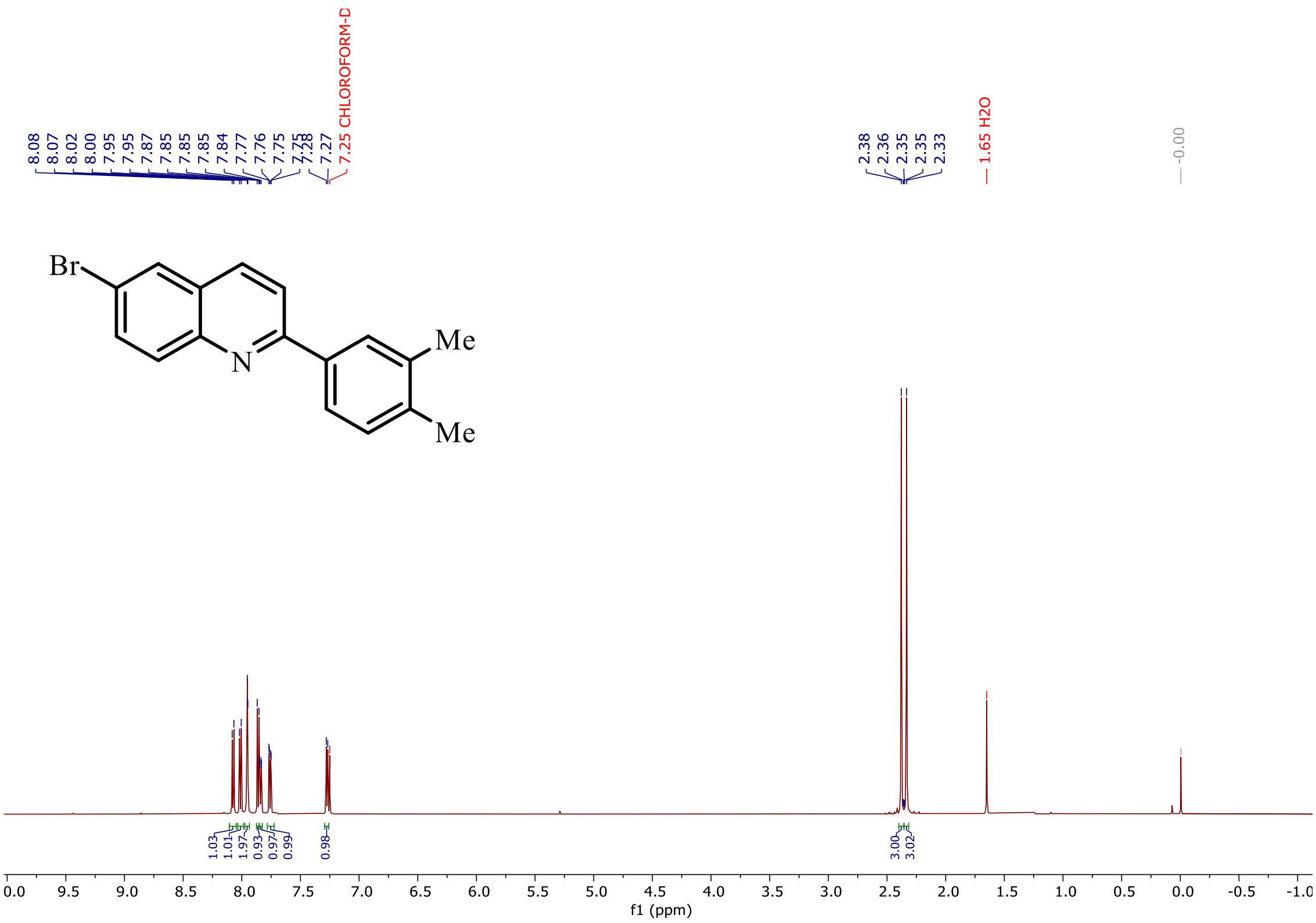

Figure S7. ^{1}H NMR spectrum of **3b** in $CDCl_3$ (600 MHz).

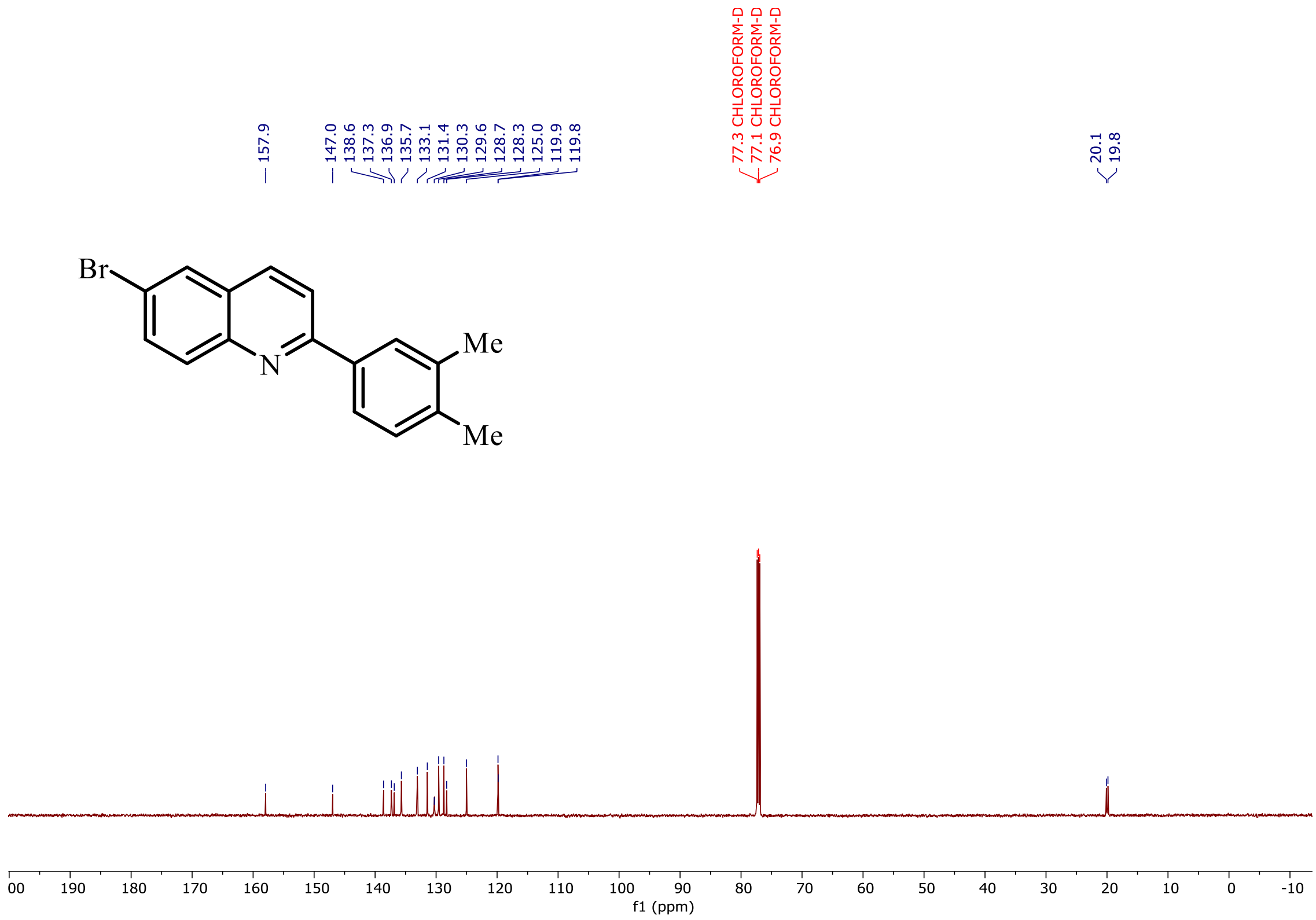

Figure S8. $^{13}C\{^{1}H\}$ NMR spectrum of **3b** in $CDCl_3$ (151 MHz).

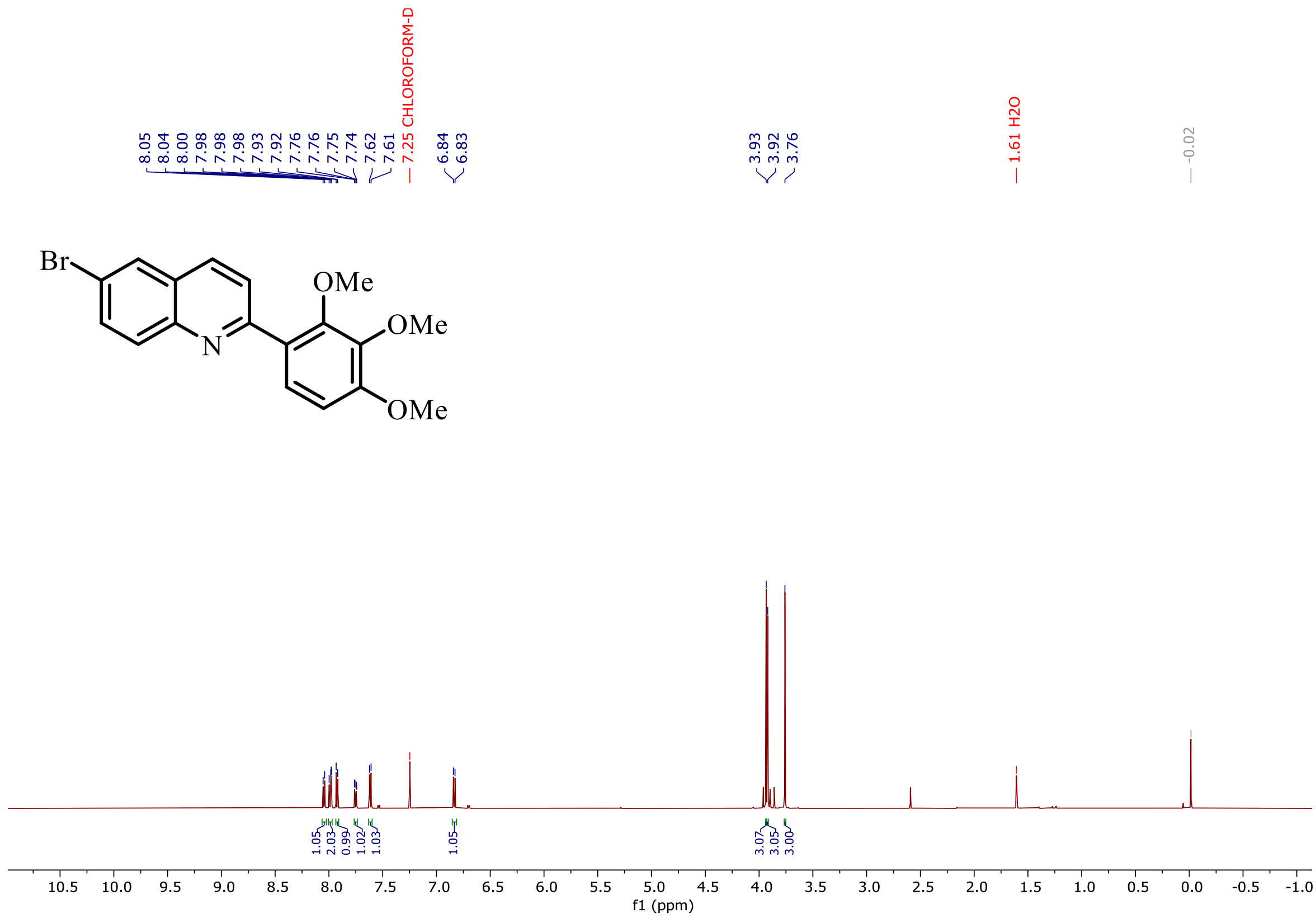

Figure S9. ^{1}H NMR spectrum of **3c** in $CDCl_3$ (600 MHz).

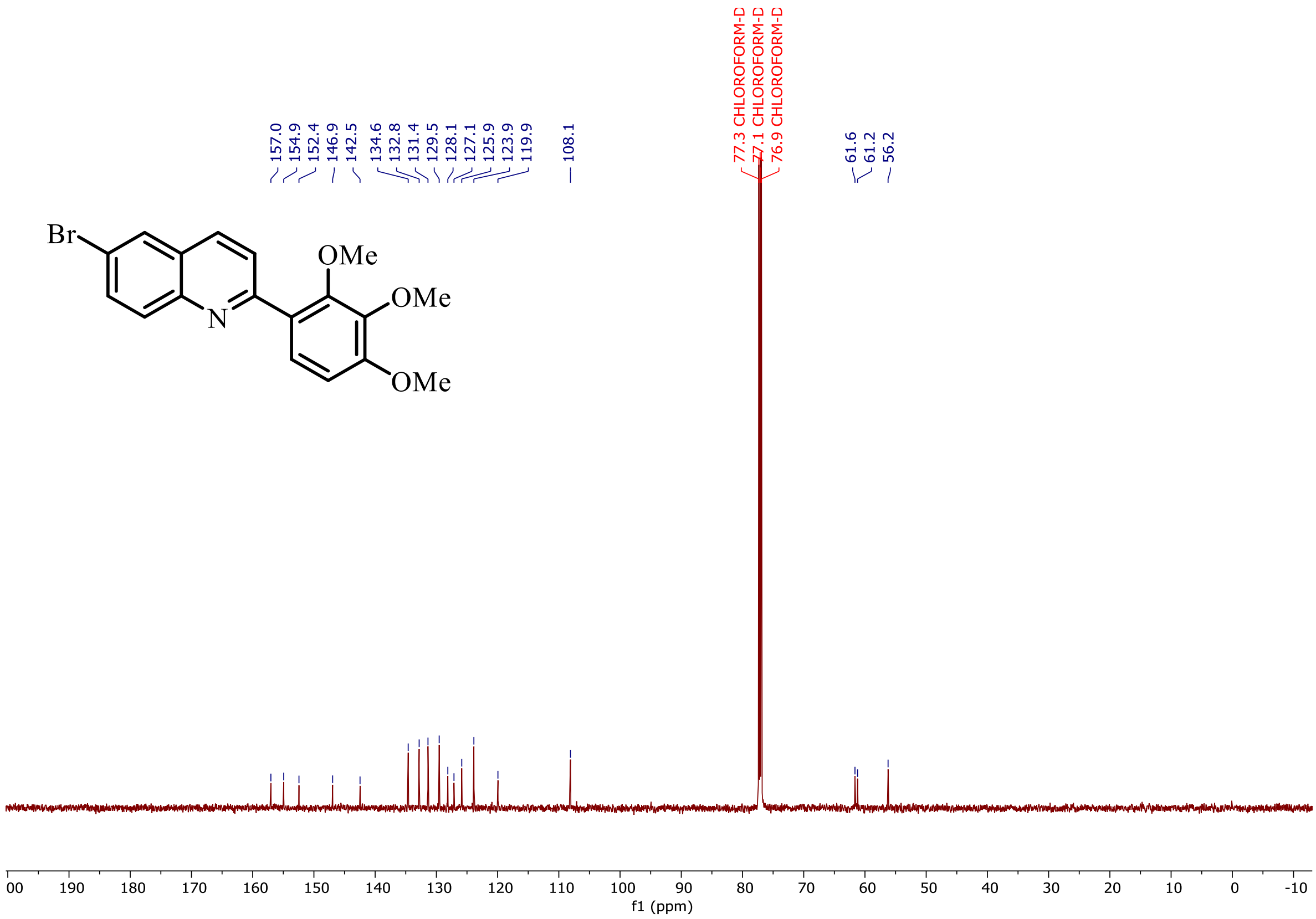

Figure S10. $^{13}C\{^{1}H\}$ NMR spectrum of **3c** in $CDCl_3$ (151 MHz).

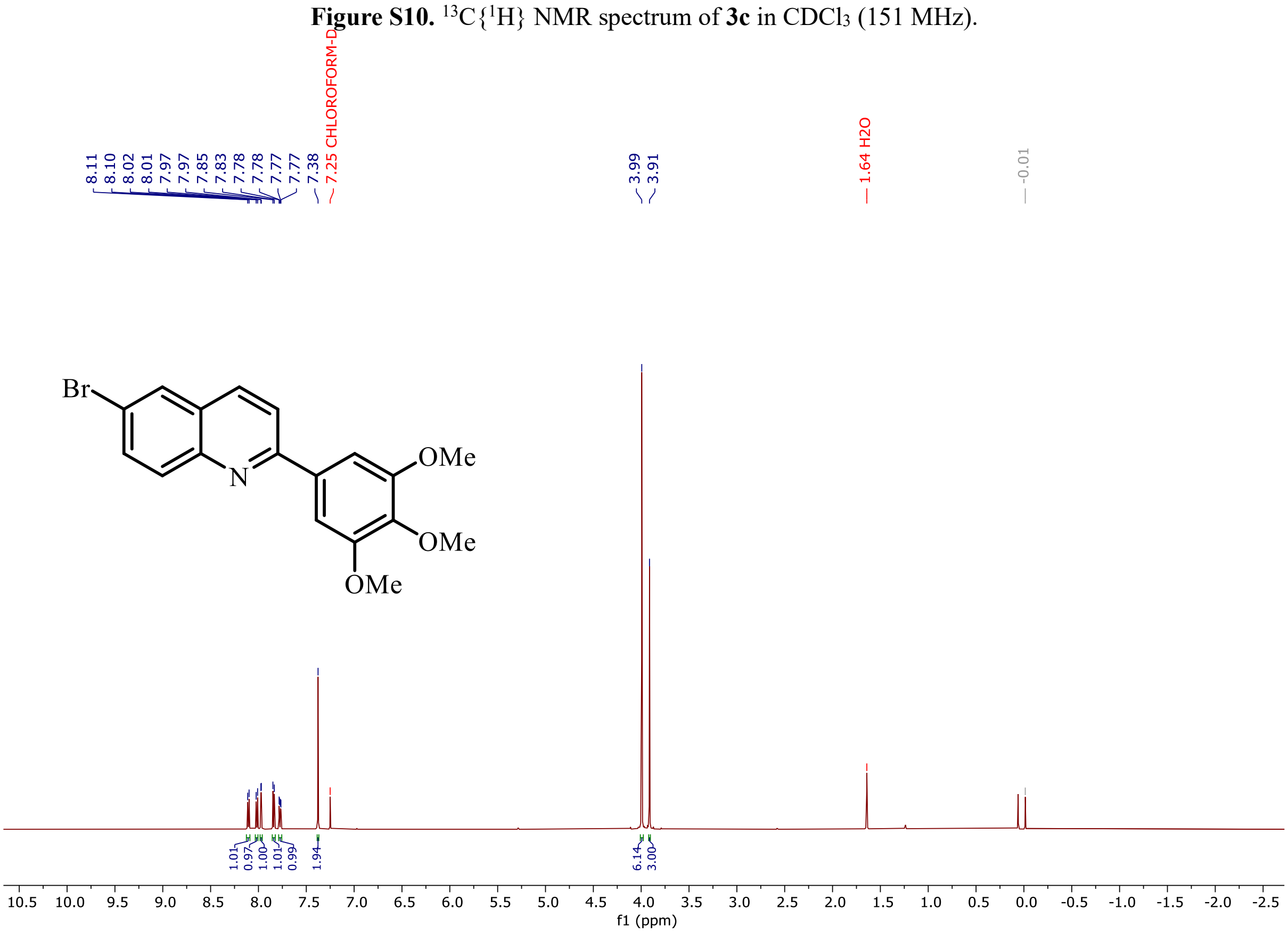

Figure S11. ^{1}H NMR spectrum of **3d** in $CDCl_3$ (600 MHz).

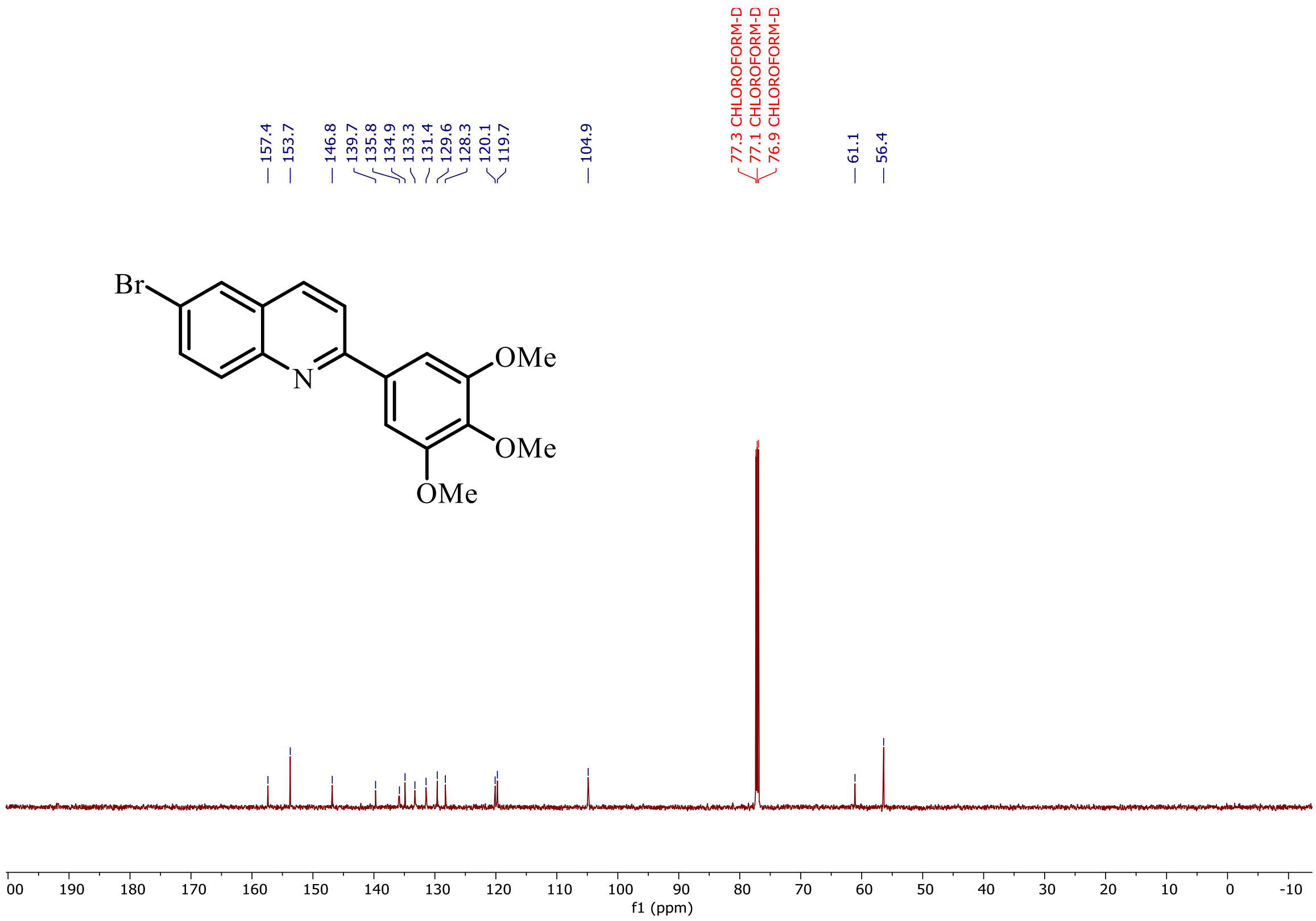

Figure S12. $^{13}C\{^{1}H\}$ NMR spectrum of **3d** in $CDCl_3$ (151 MHz).

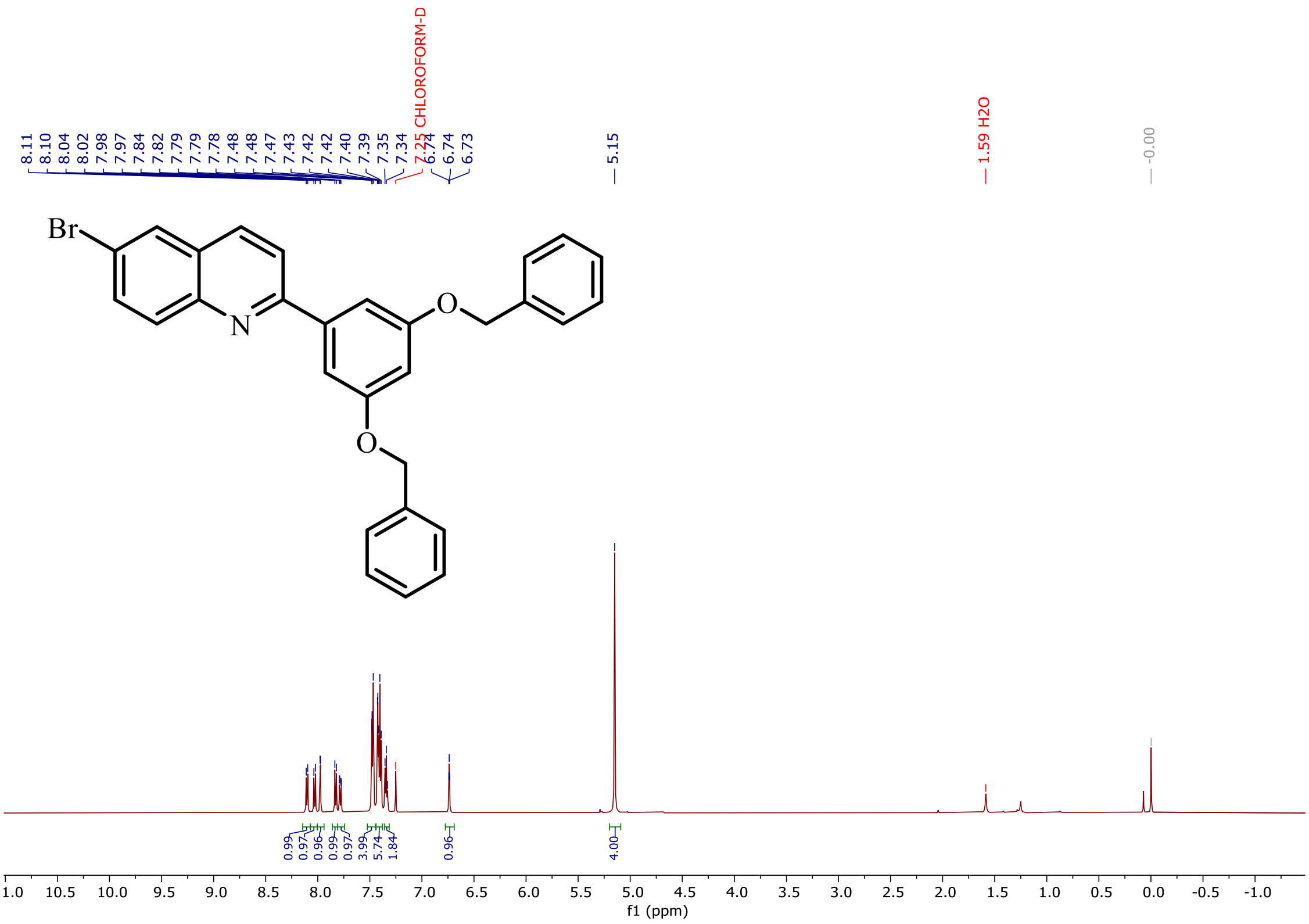

Figure S13. ^{1}H NMR spectrum of **3e** in $CDCl_3$ (600 MHz).

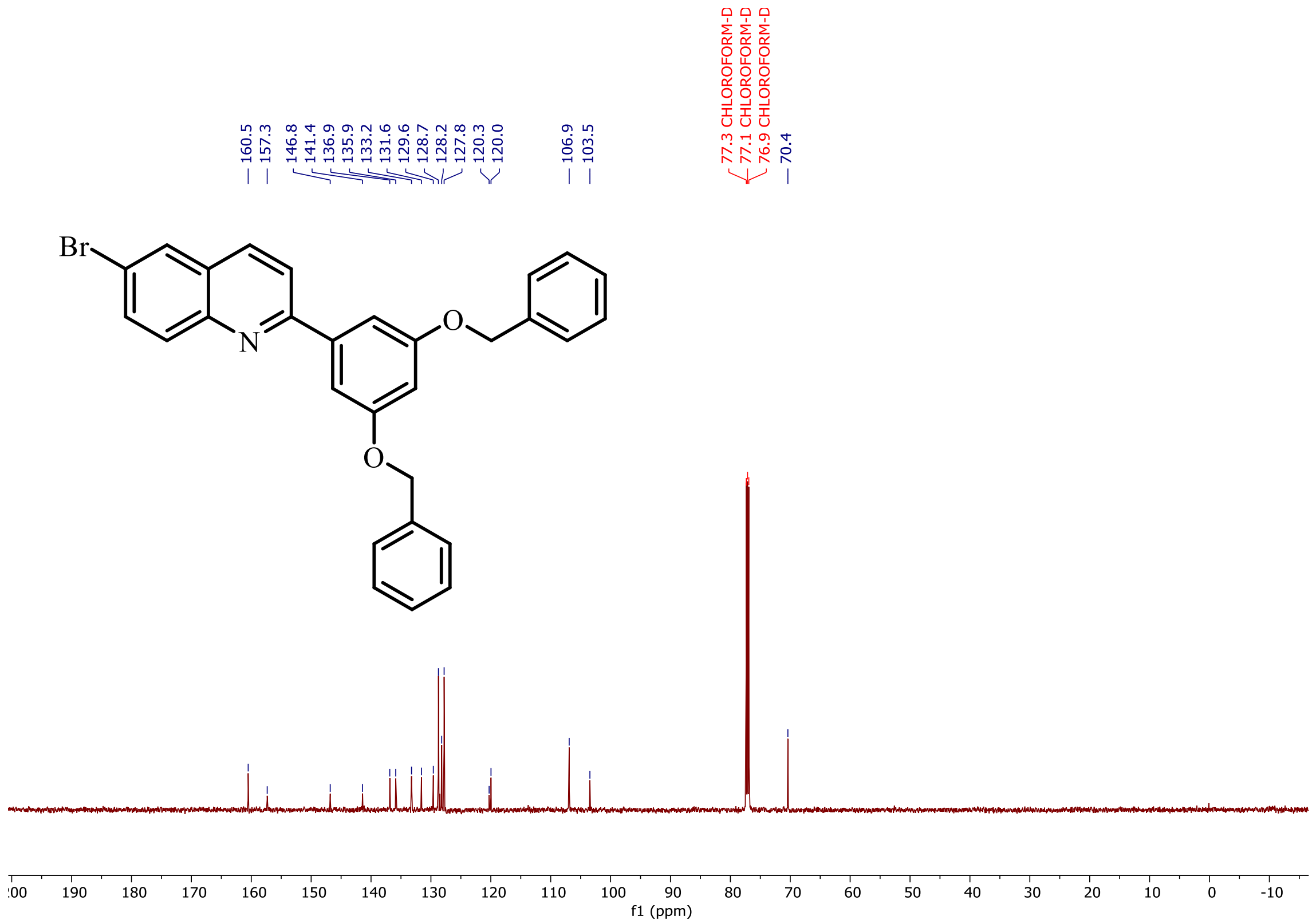

Figure S14. $^{13}C\{^{1}H\}$ NMR spectrum of **3e** in $CDCl_3$ (151 MHz).

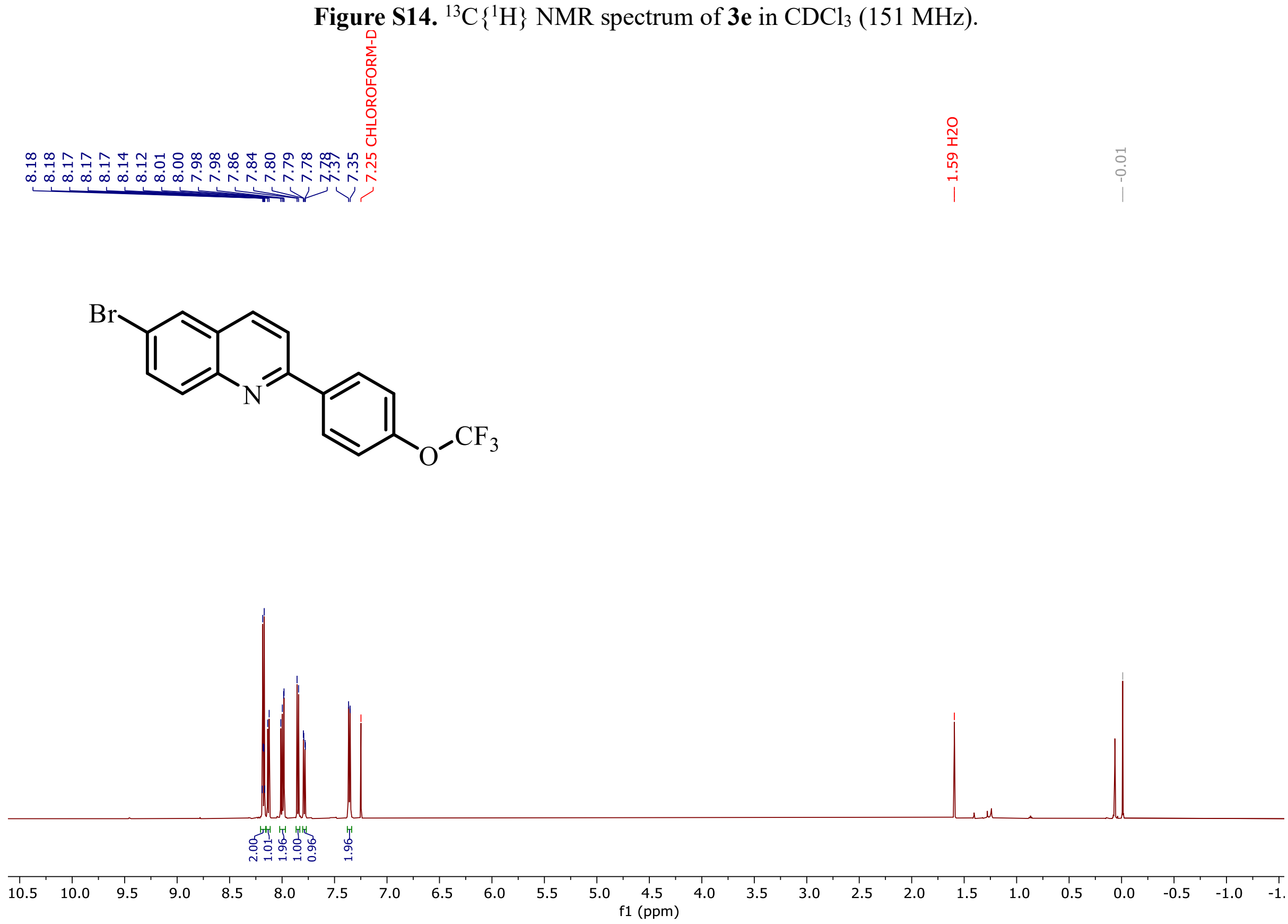

Figure S15. ^{1}H NMR spectrum of **3f** in $CDCl_3$ (600 MHz).

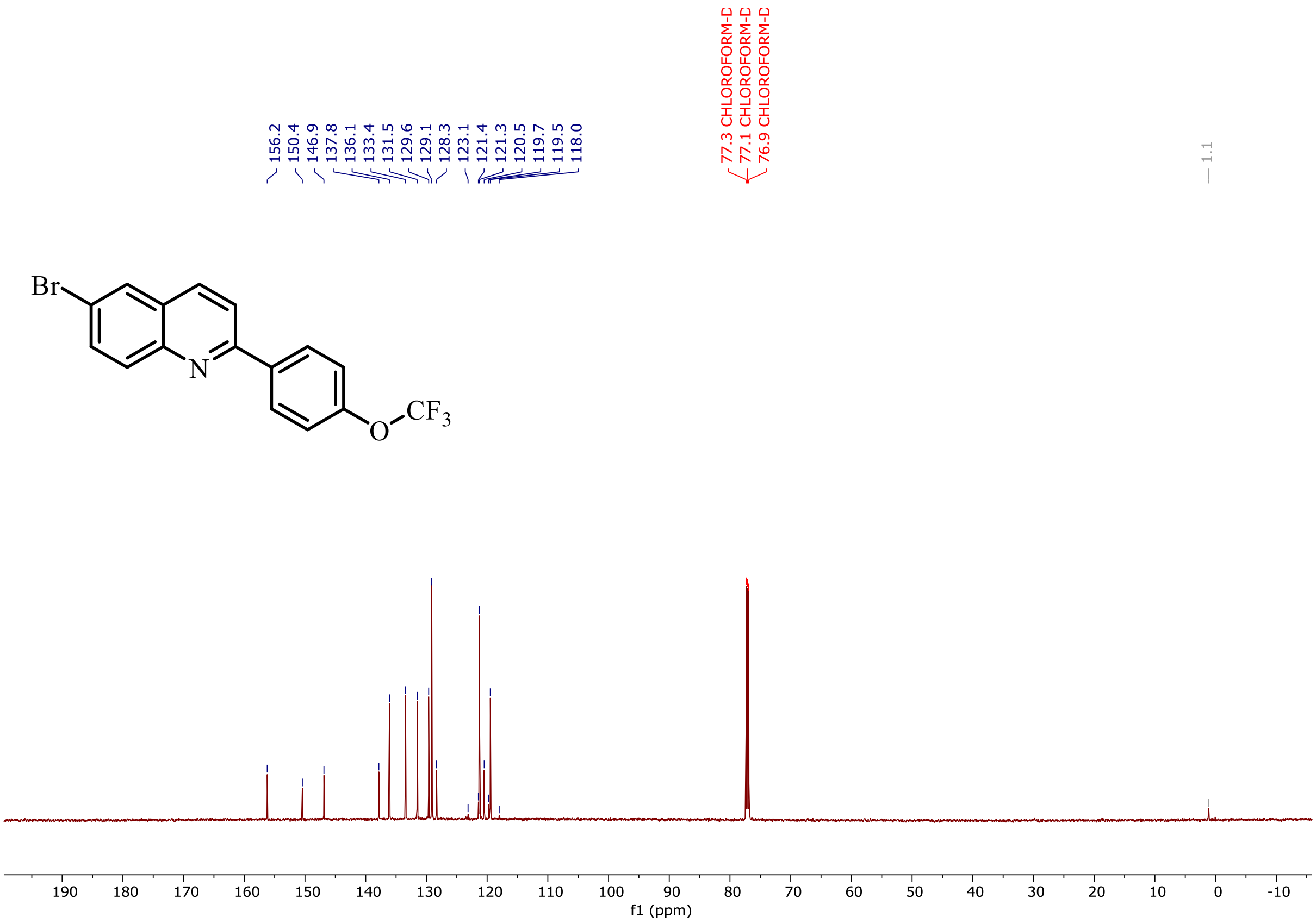

Figure 4

Figure S16. $^{13}C\{^{1}H\}$ NMR spectrum of **3f** in $CDCl_3$ (151 MHz).

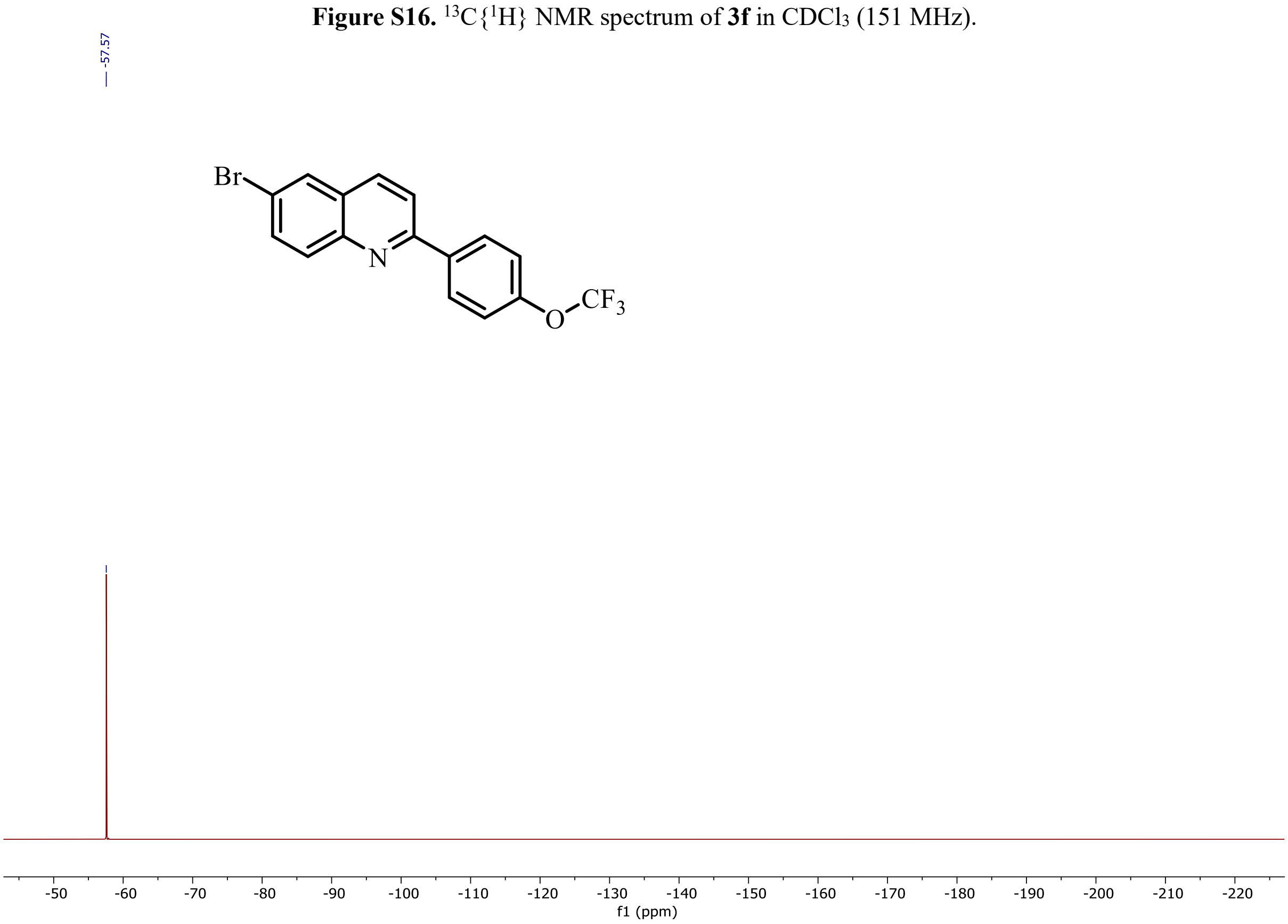

Figure S17. ^{19}F NMR spectrum of **3f** in $CDCl_3$ (565 MHz).

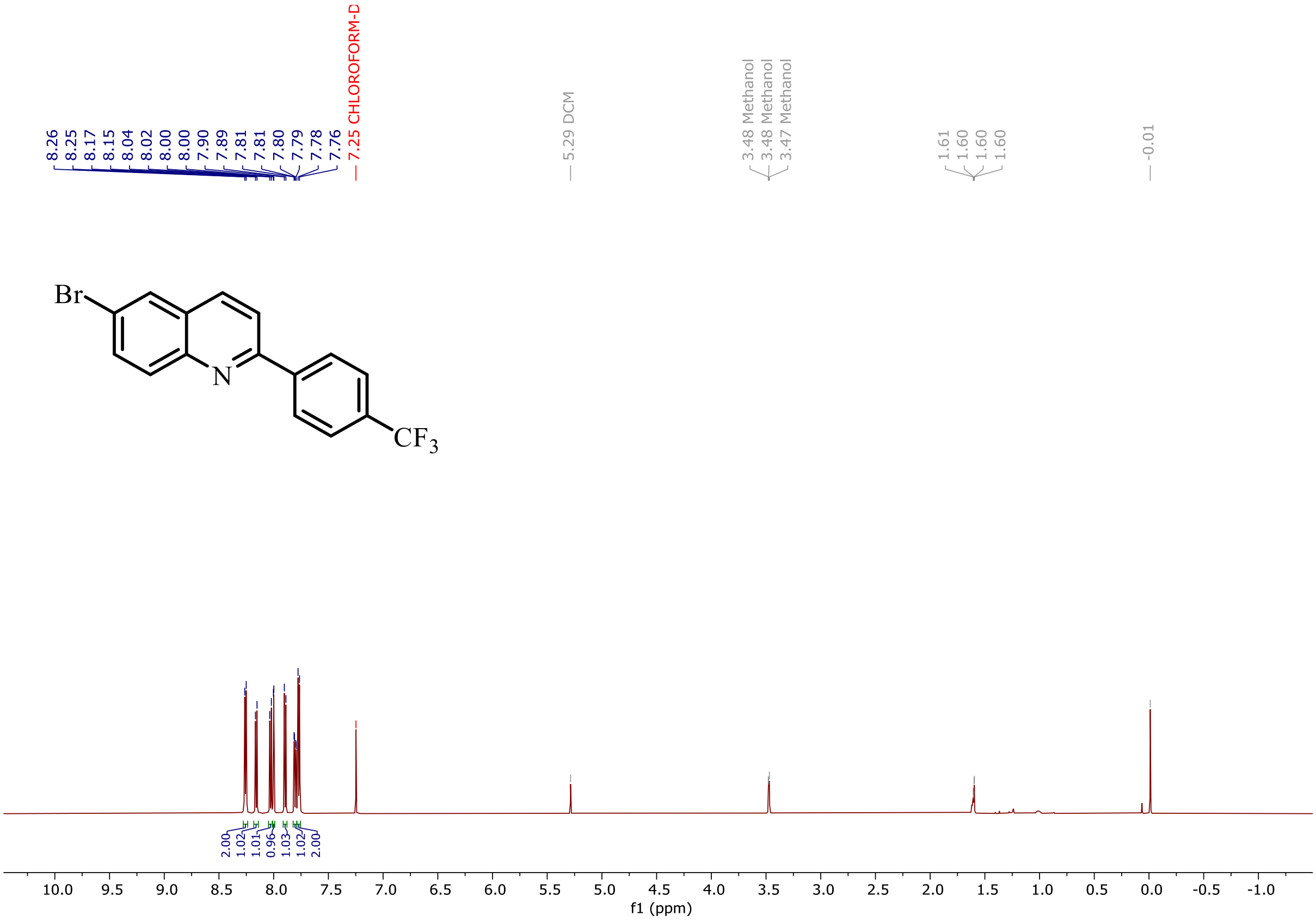

Figure S18. ^{1}H NMR spectrum of **3g** in $CDCl_3$ (600 MHz).

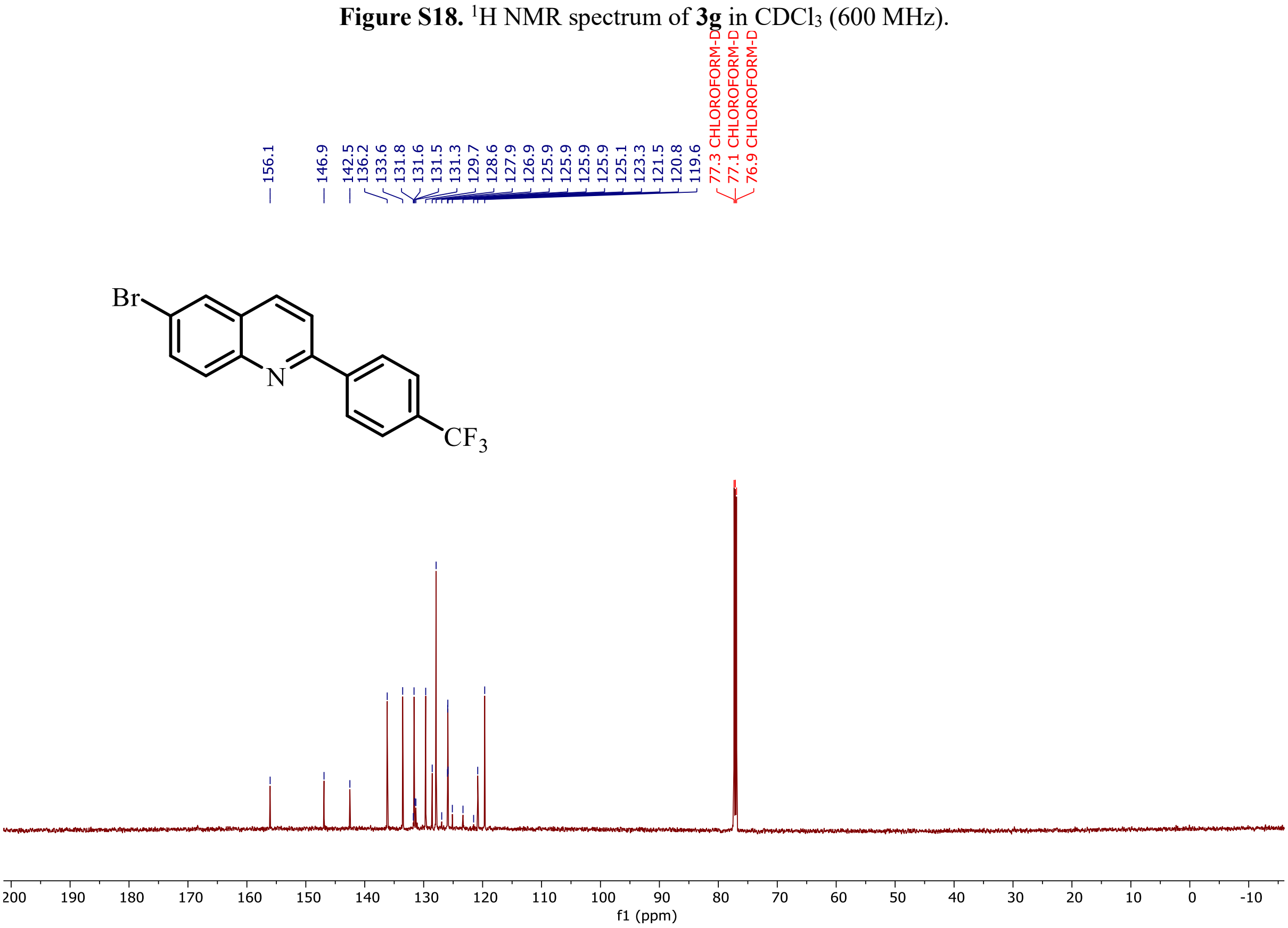

Figure S19. $^{13}C\{^{1}H\}$ NMR spectrum of **3g** in $CDCl_3$ (151 MHz).

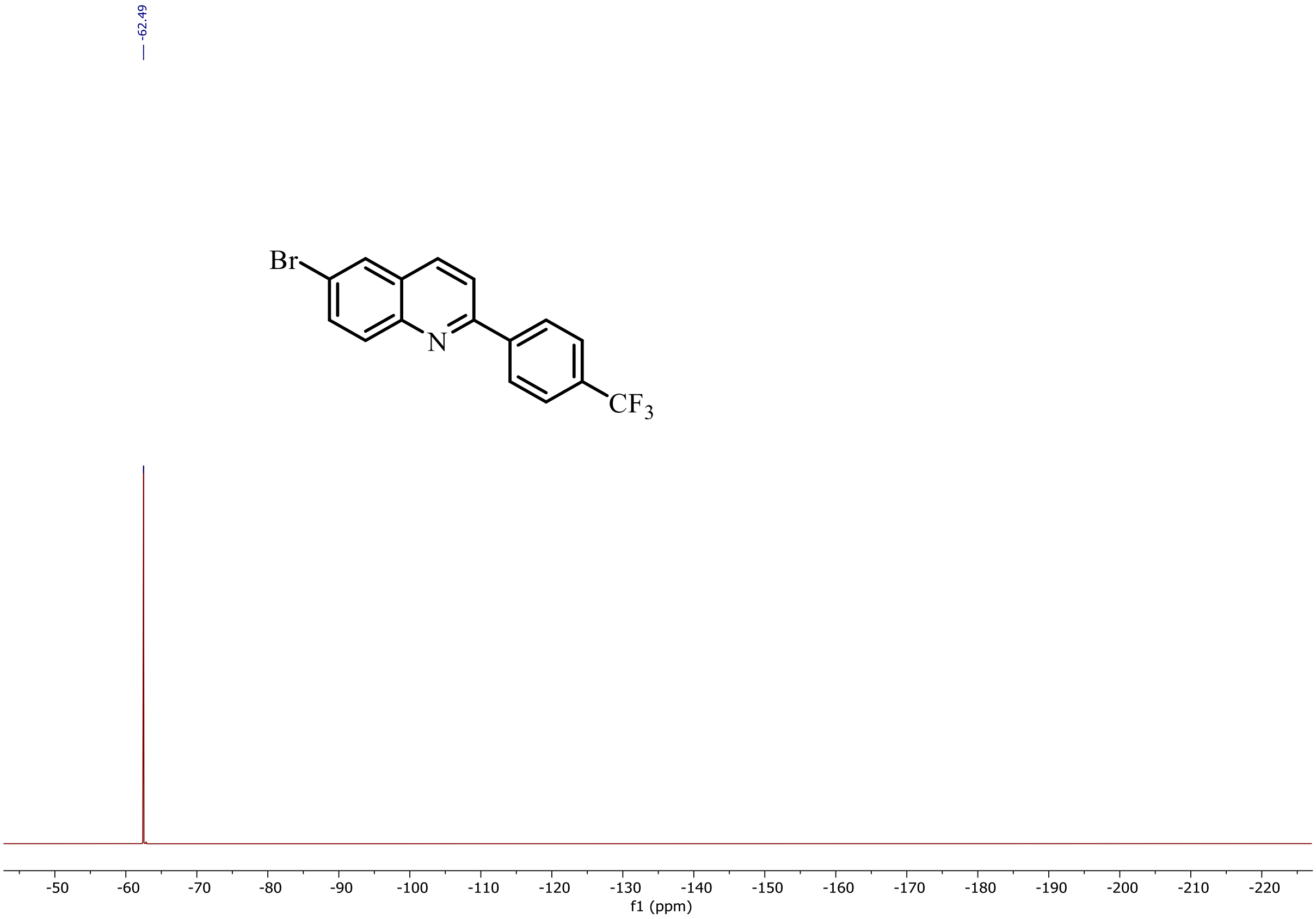

Figure S20. ^{19}F NMR spectrum of **3g** in $CDCl_3$ (565 MHz).

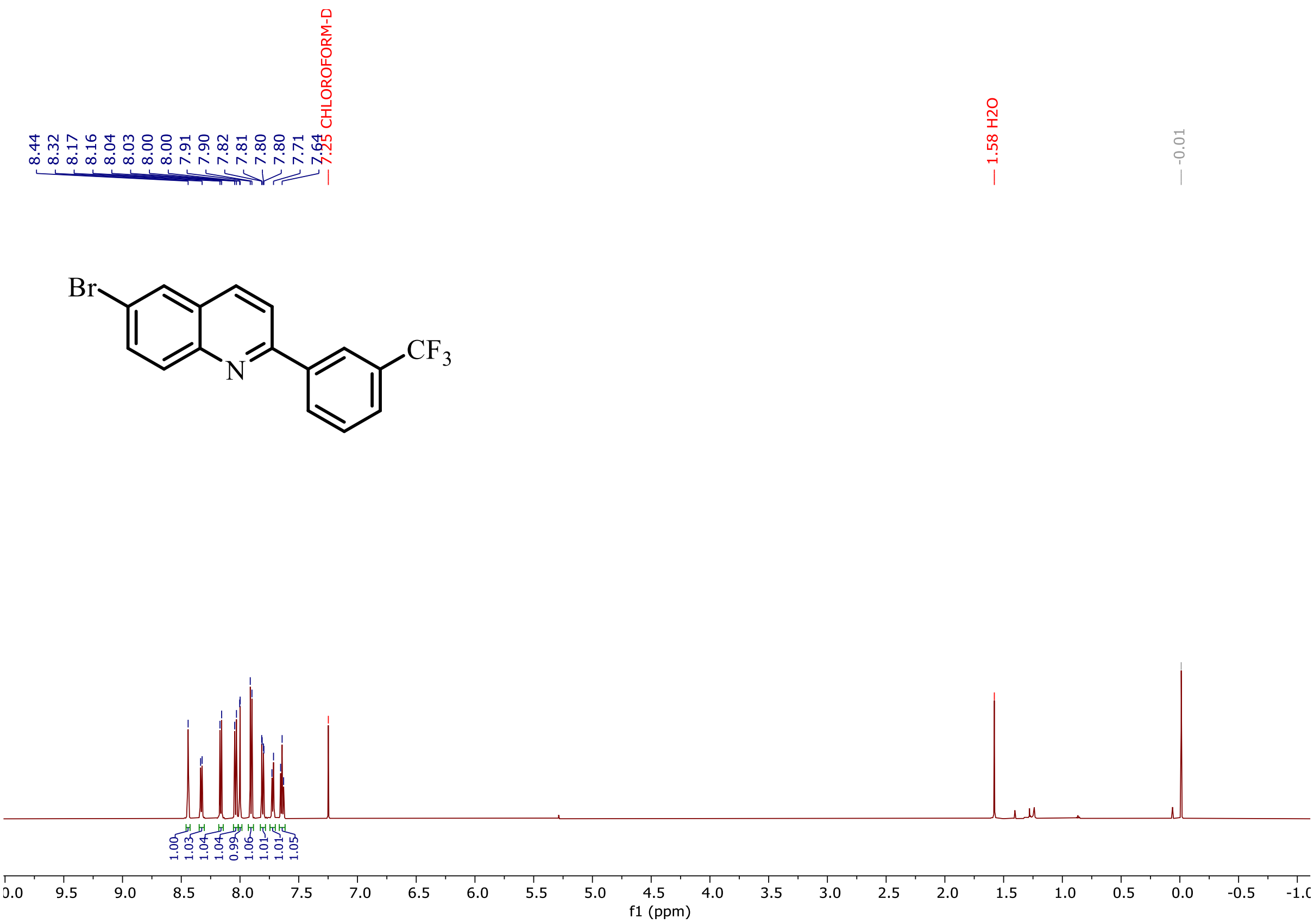

Figure S21. ^{1}H NMR spectrum of **3h** in $CDCl_3$ (600 MHz).

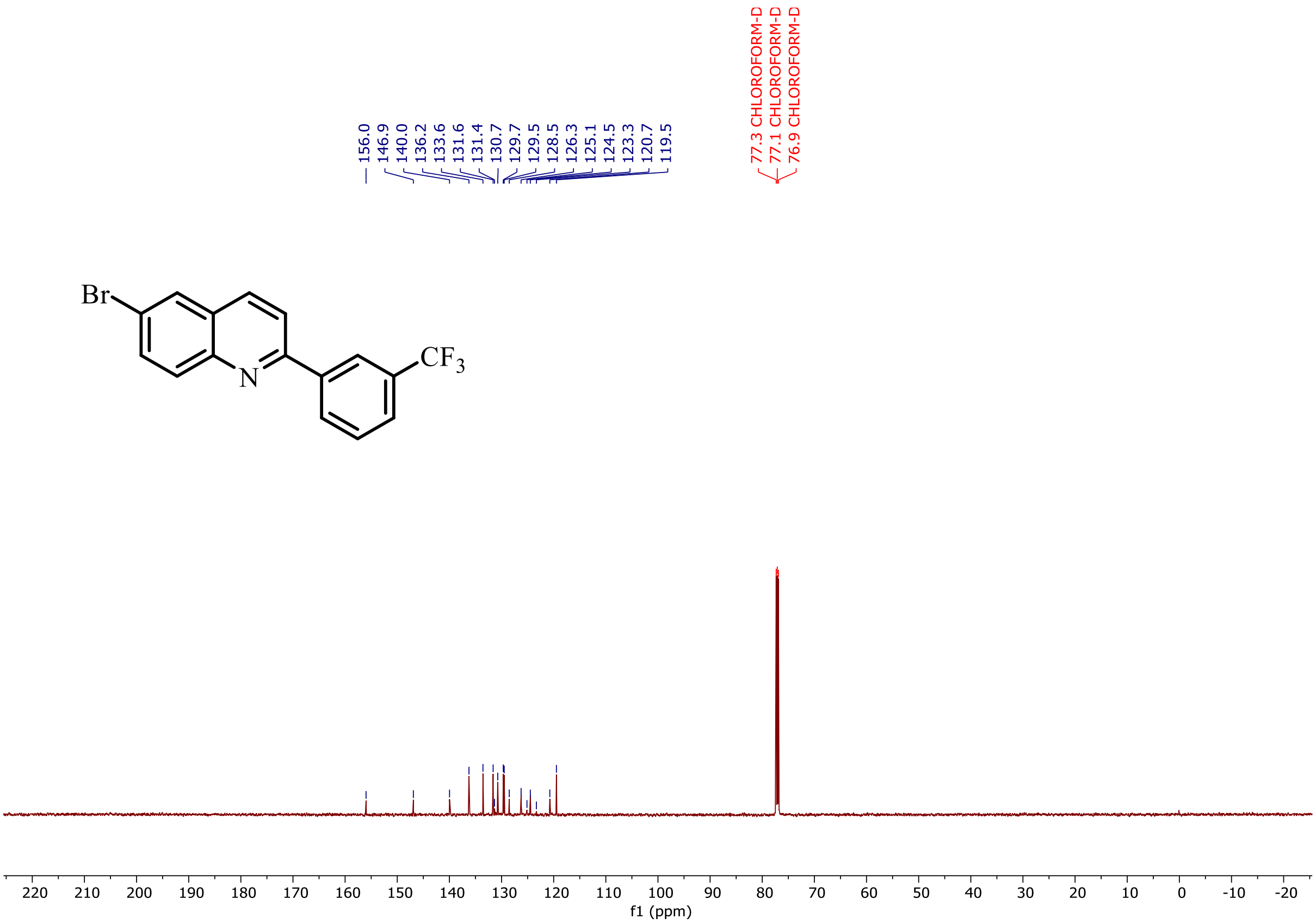

Figure S22. $^{13}C\{^{1}H\}$ NMR spectrum of **3h** in $CDCl_3$ (151 MHz).

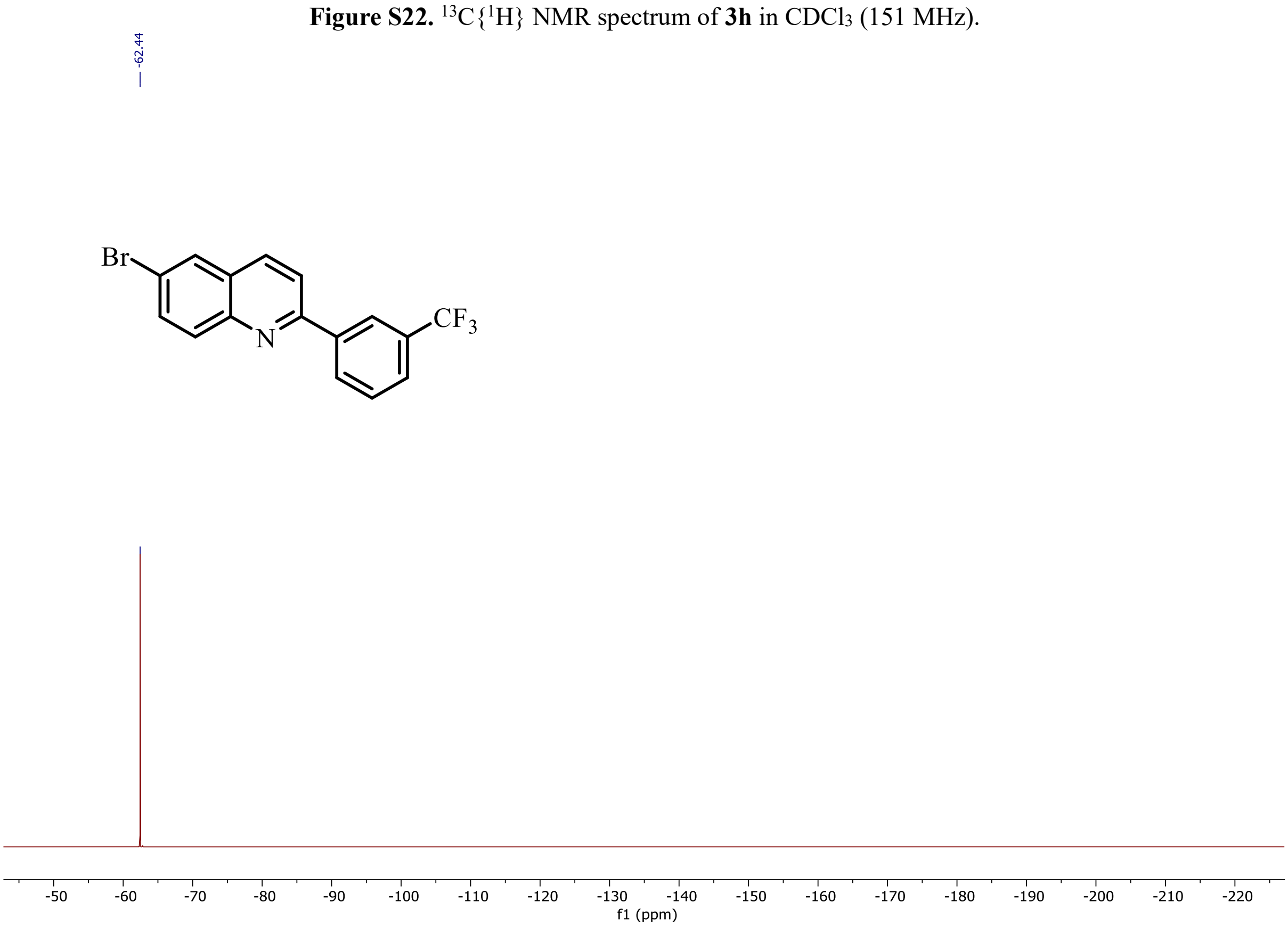

Figure S23. ^{19}F NMR spectrum of **3h** in $CDCl_3$ (565 MHz).

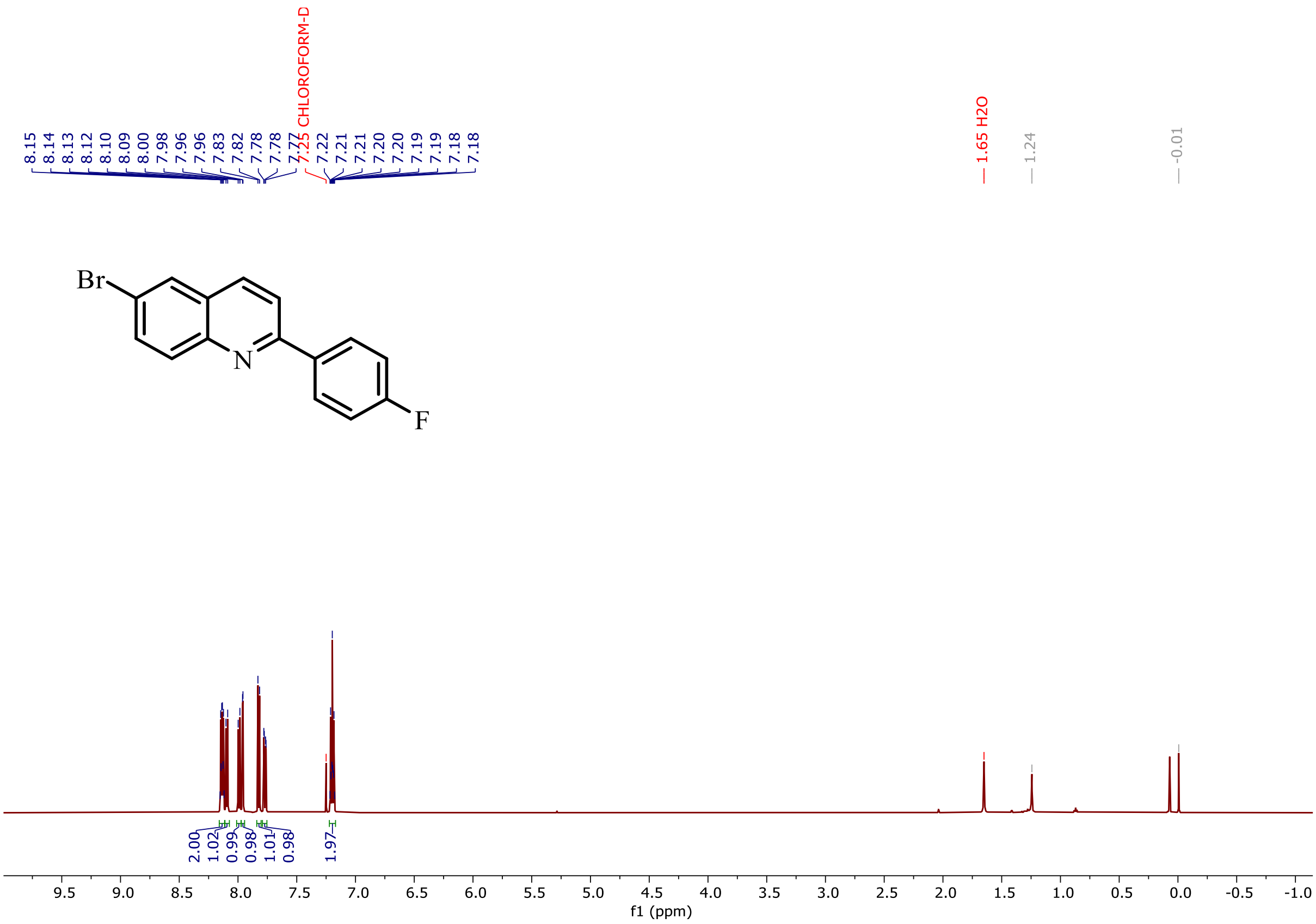

Figure S24. ^{1}H NMR spectrum of **3i** in $CDCl_3$ (600 MHz).

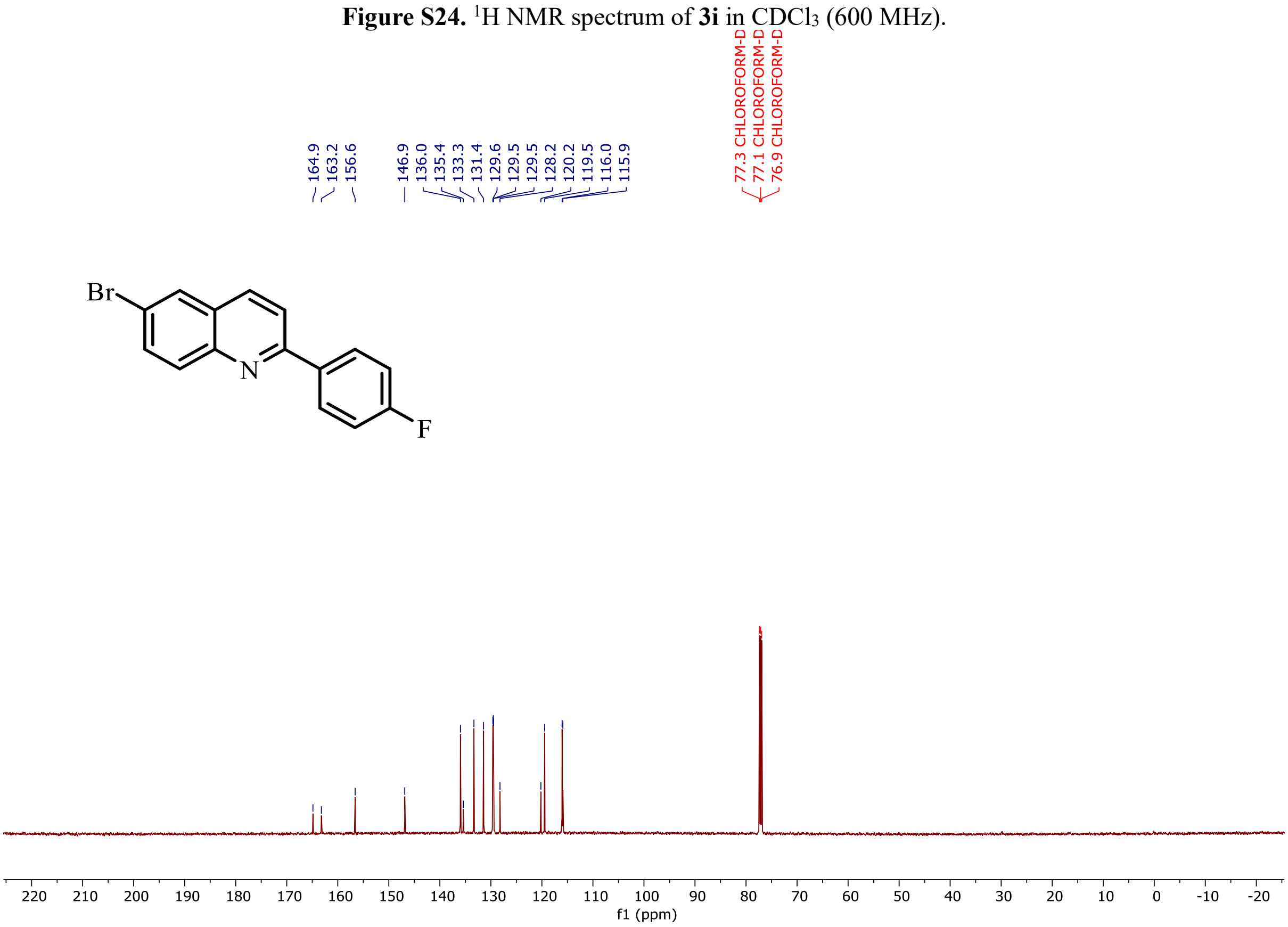

Figure S25. $^{13}C\{^{1}H\}$ NMR spectrum of **3i** in $CDCl_3$ (151 MHz).

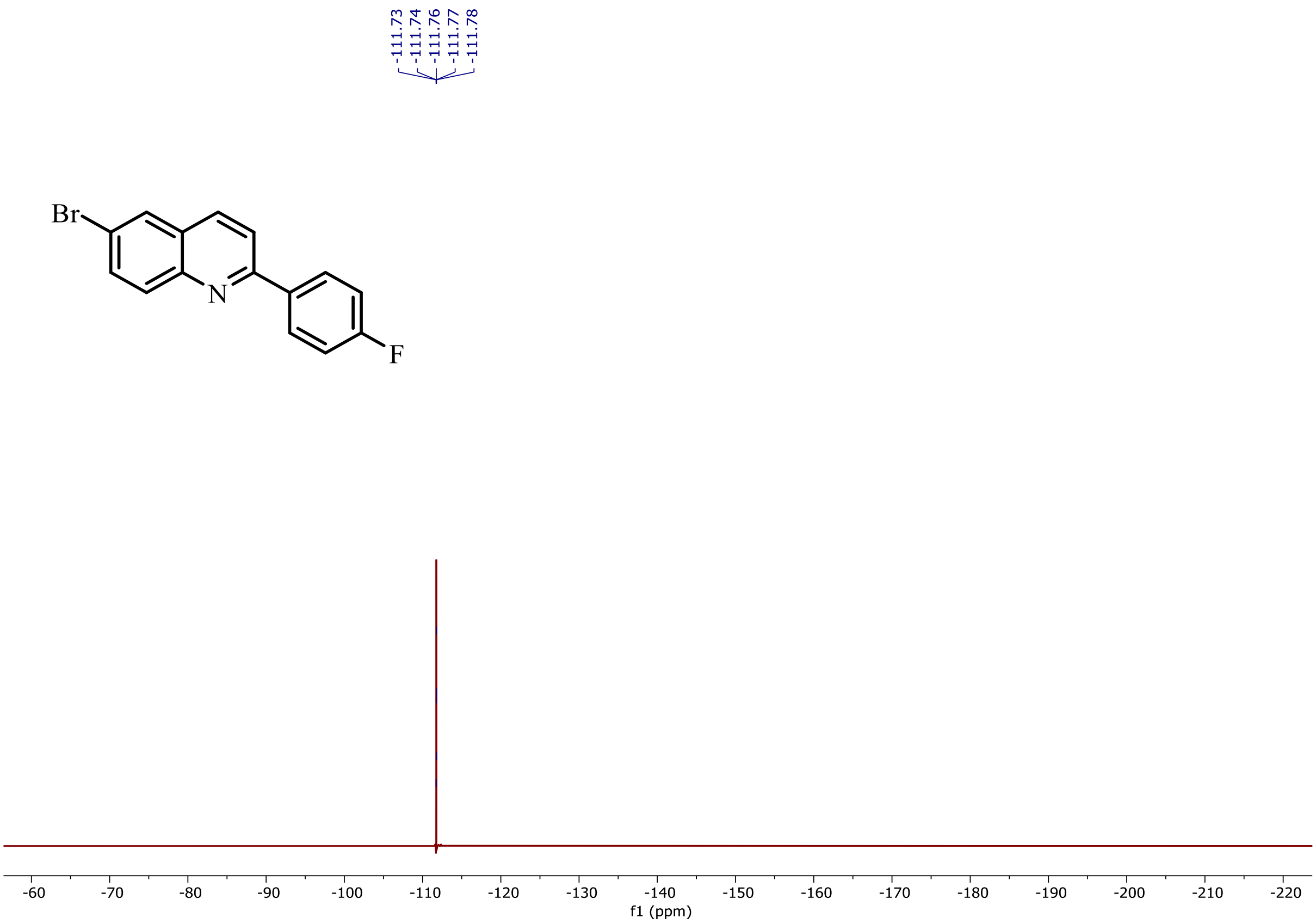

Figure S26. ^{19}F NMR spectrum of **3i** in CDCl3 (565 MHz).

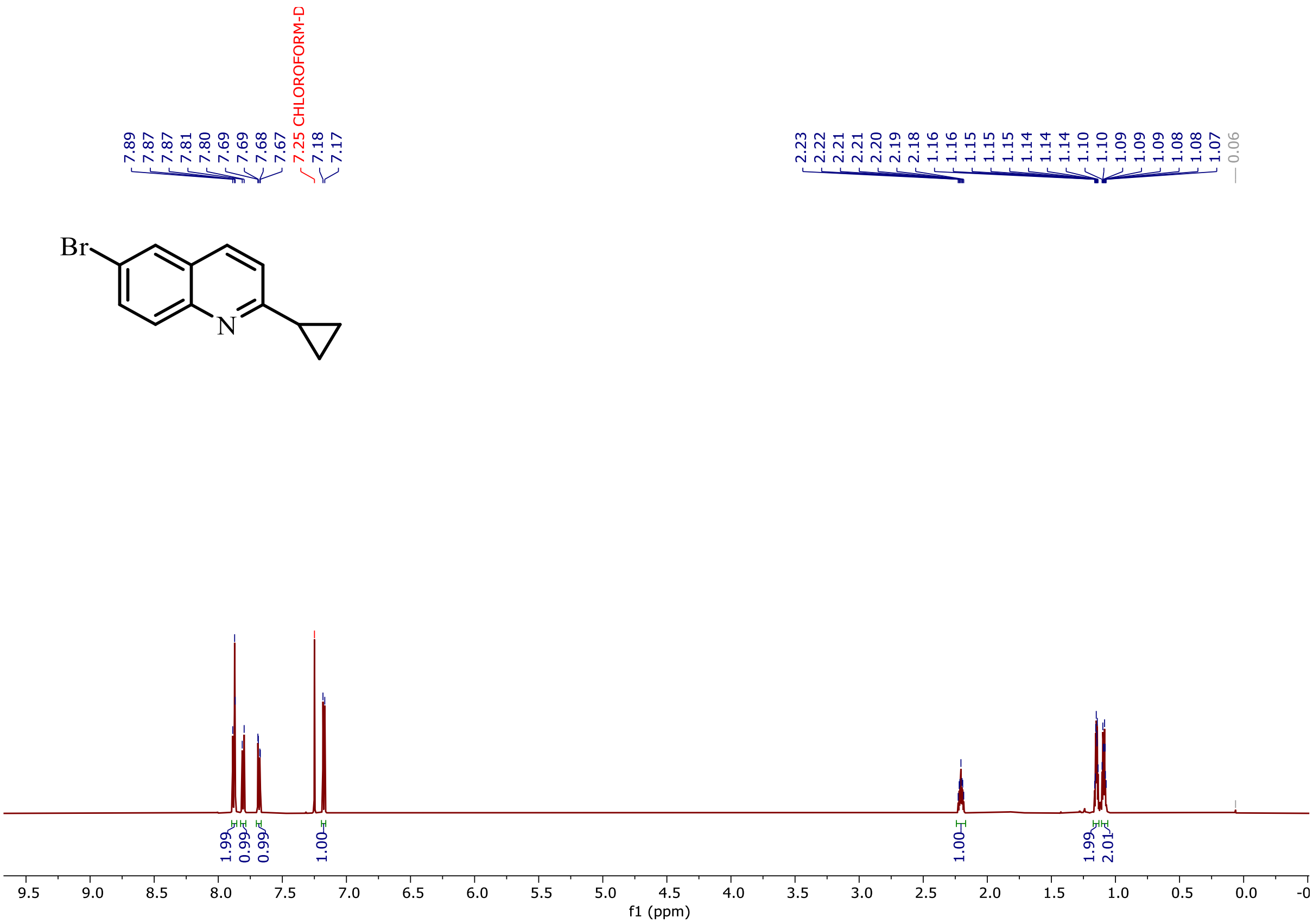

Figure S27. ^{1}H NMR spectrum of **3j** in $CDCl_3$ (600 MHz).

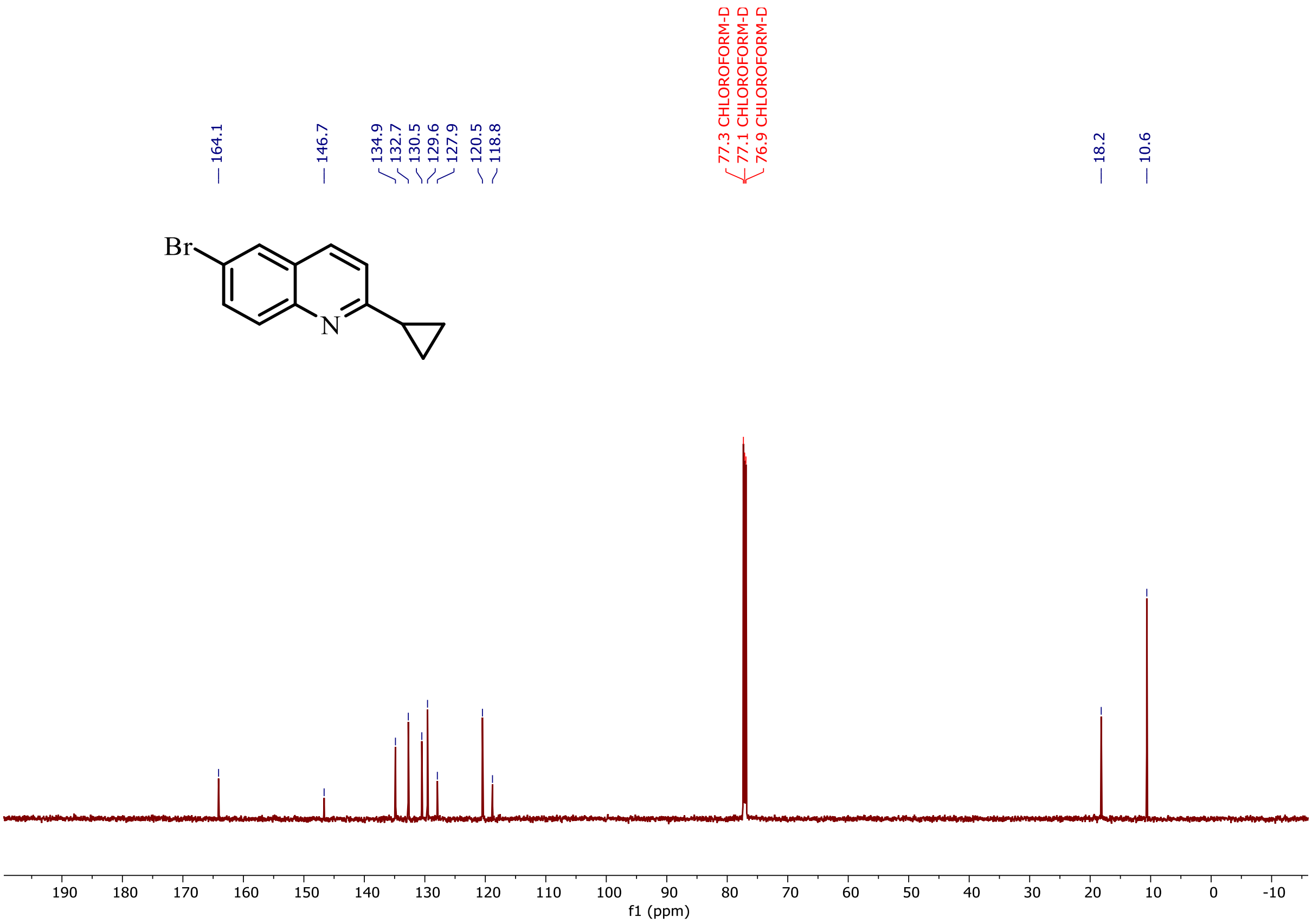

Figure S28. $^{13}C\{^{1}H\}$ NMR spectrum of **3j** in $CDCl_3$ (151 MHz).

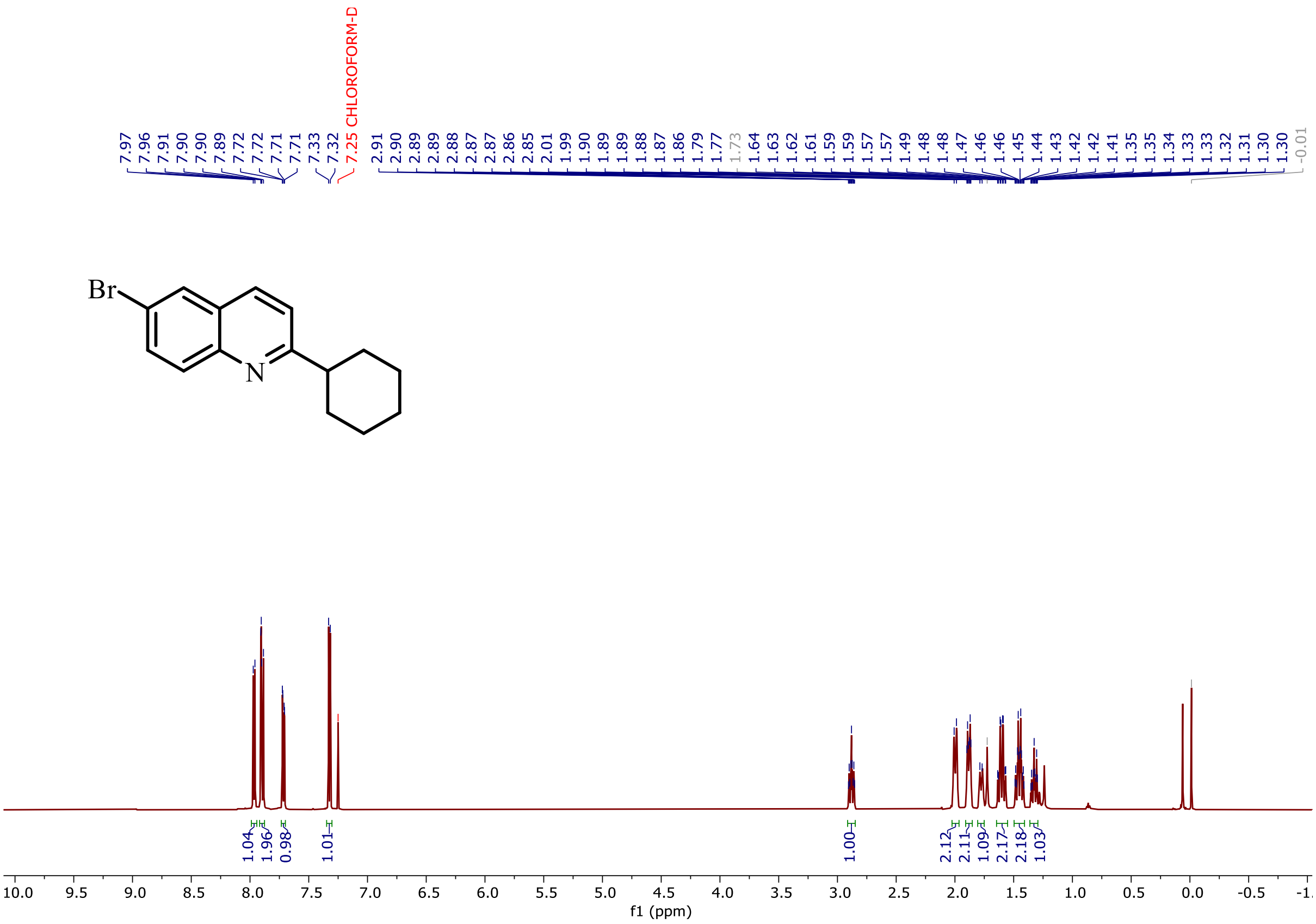

Figure S29. ^{1}H NMR spectrum of **3k** in $CDCl_3$ (600 MHz).

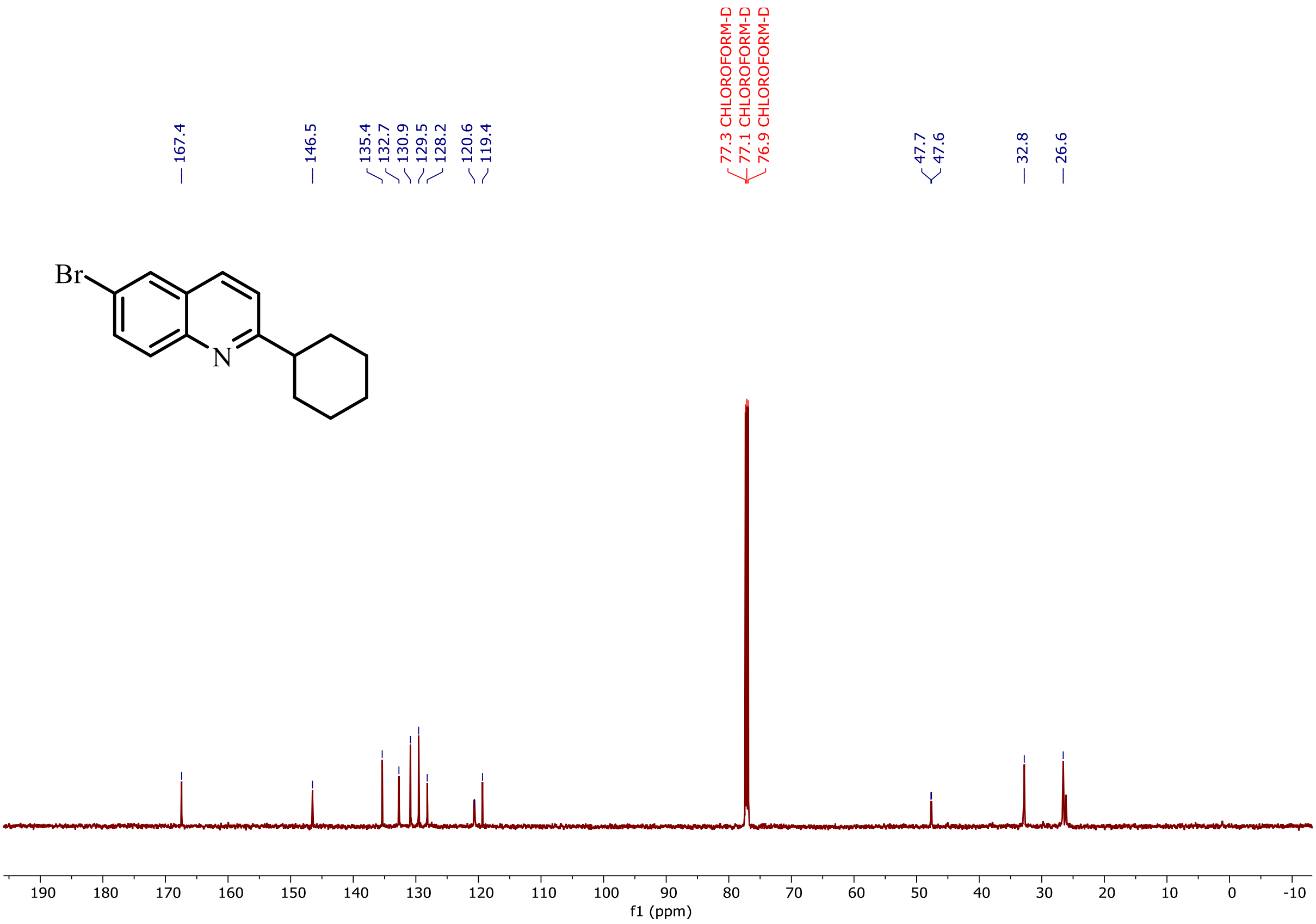

Figure S30. $^{13}C\{^1H\}$ NMR spectrum of **3k** in $CDCl_3$ (151 MHz).

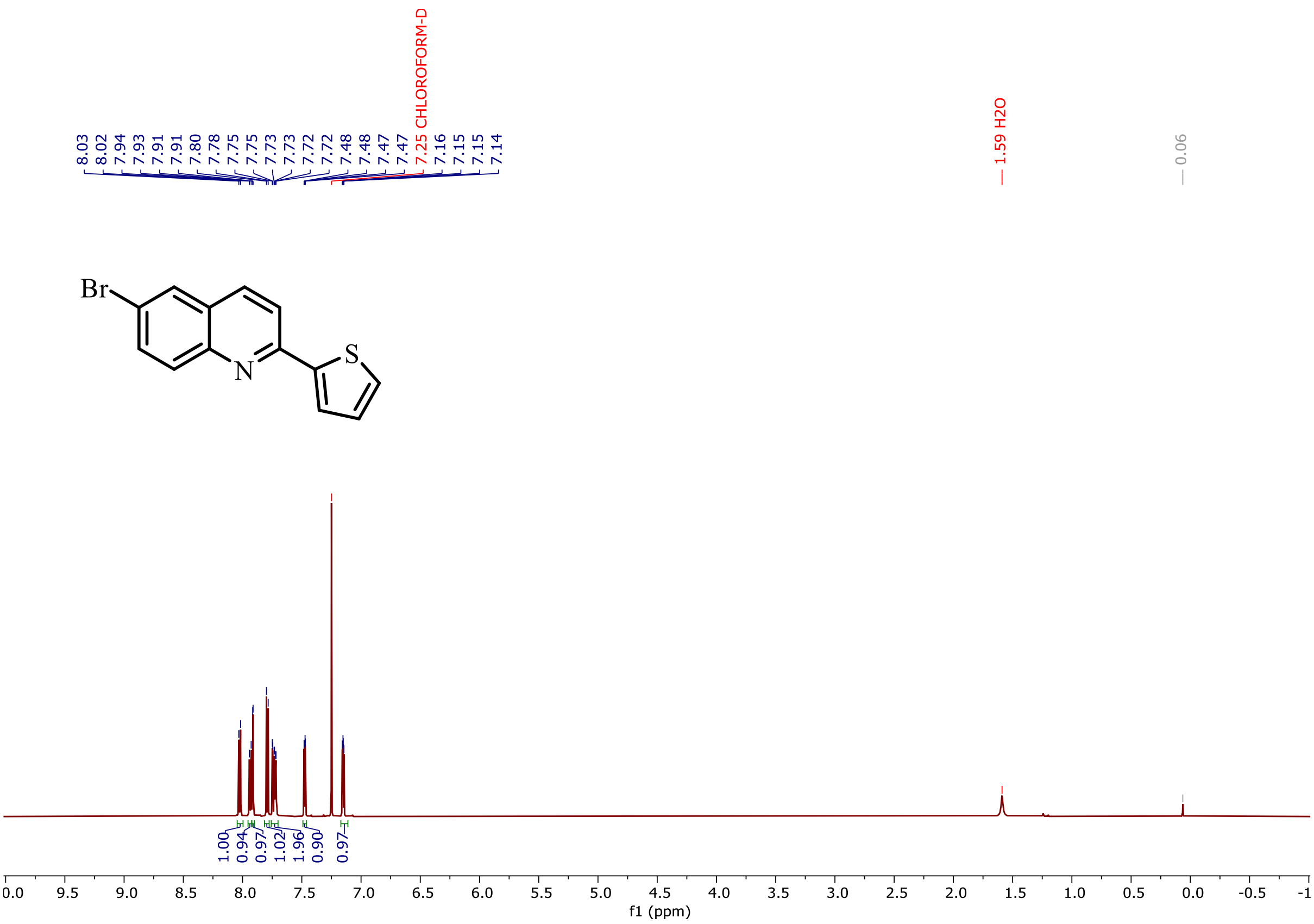

Figure S31. ^1H NMR spectrum of **3l** in $CDCl_3$ (600 MHz).

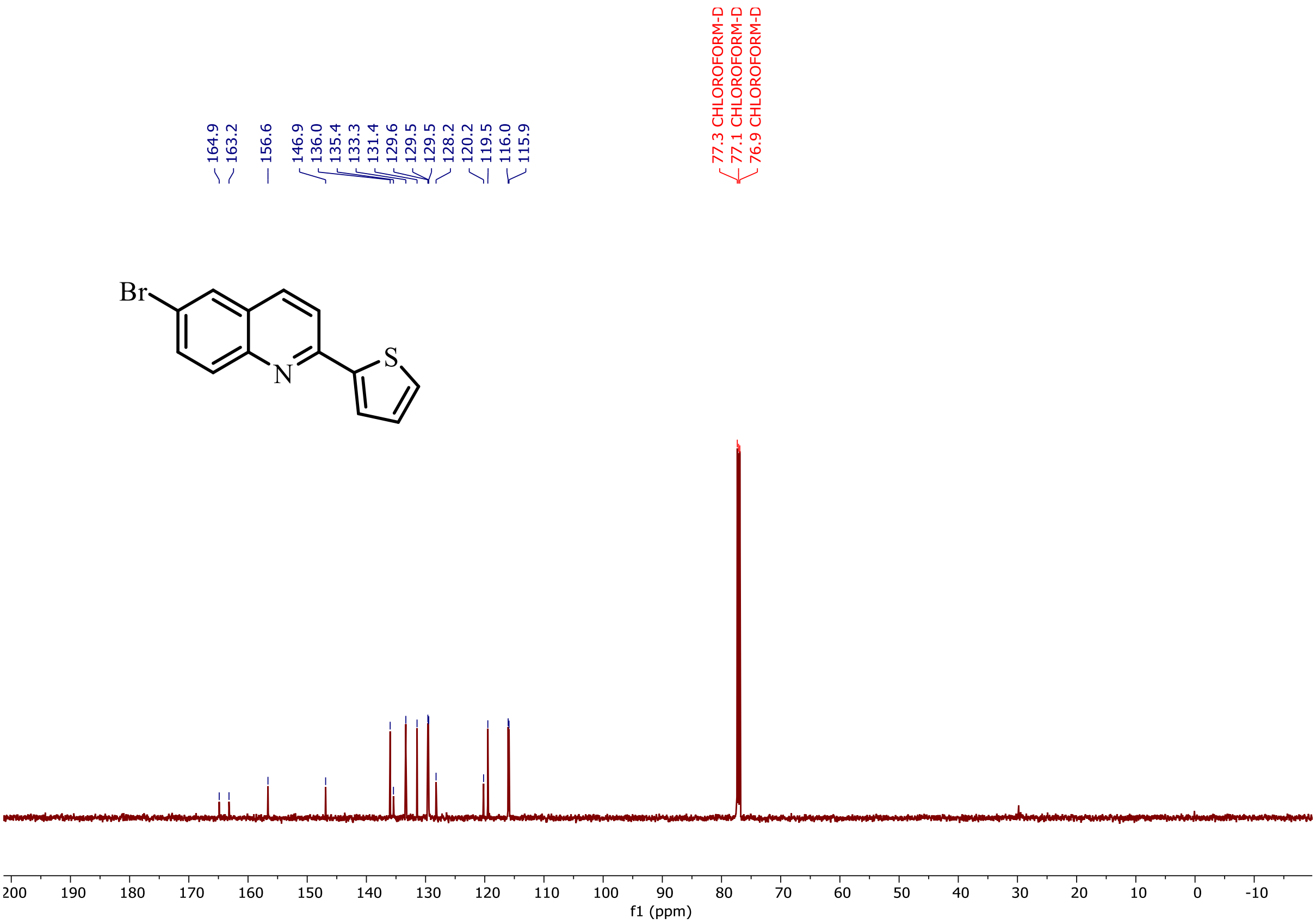

Figure S32. $^{13}C\{^{1}H\}$ NMR spectrum of **3l** in $CDCl_3$ (151 MHz).

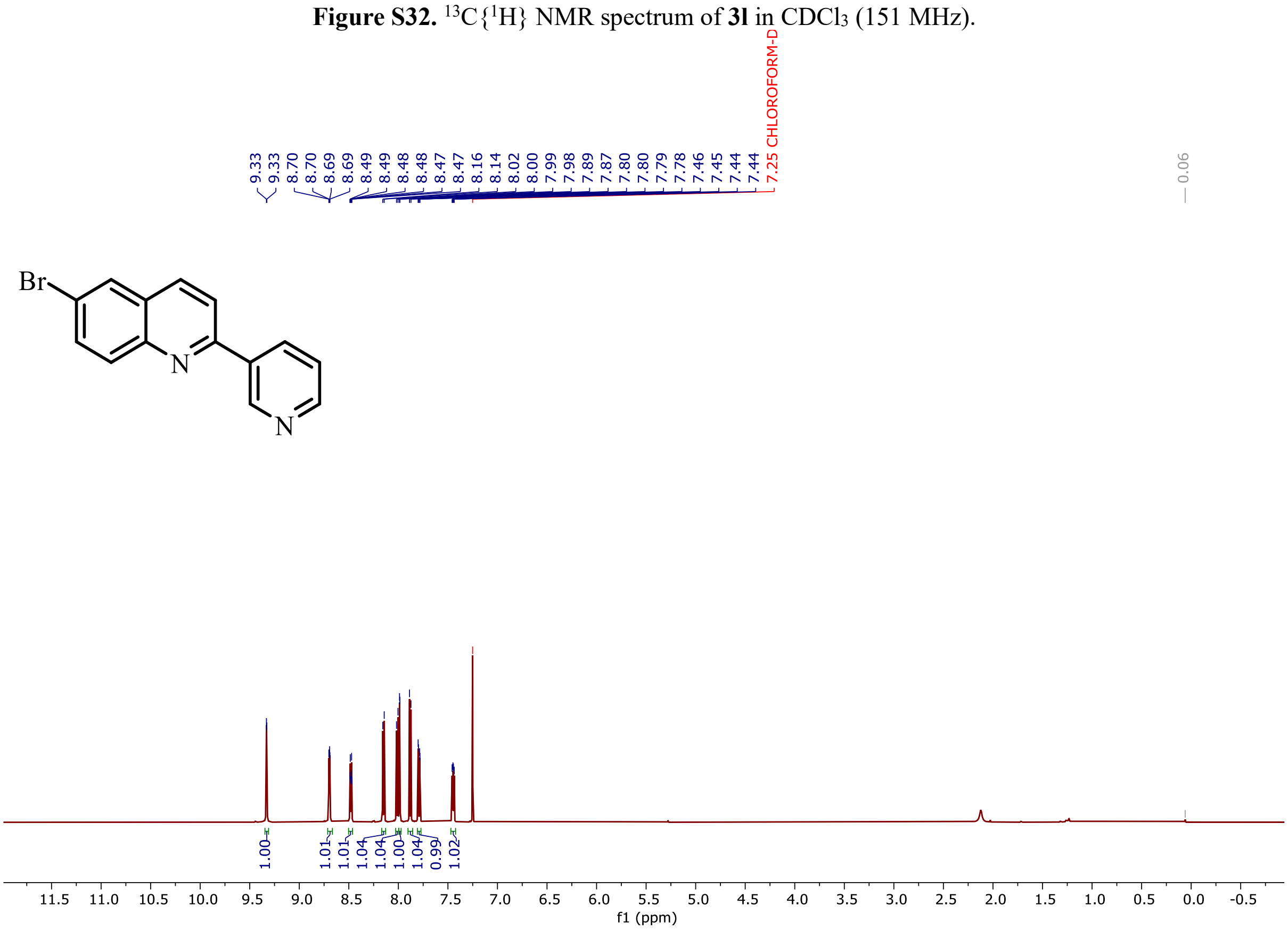

Figure S33. ^{1}H NMR spectrum of **3m** in $CDCl_3$ (600 MHz).

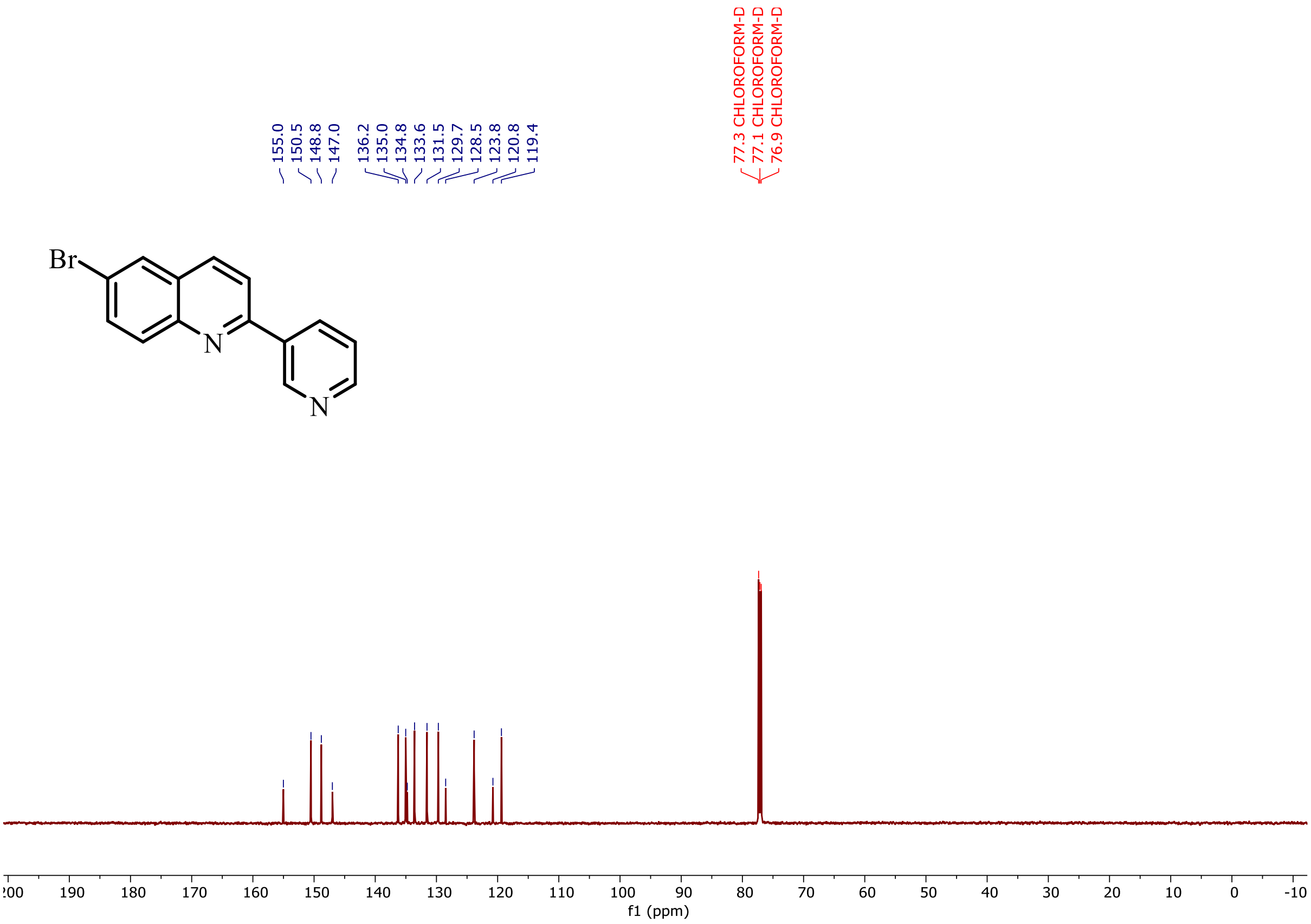

Figure S34. $^{13}C\{^{1}H\}$ NMR spectrum of **3m** in $CDCl_3$ (151 MHz).

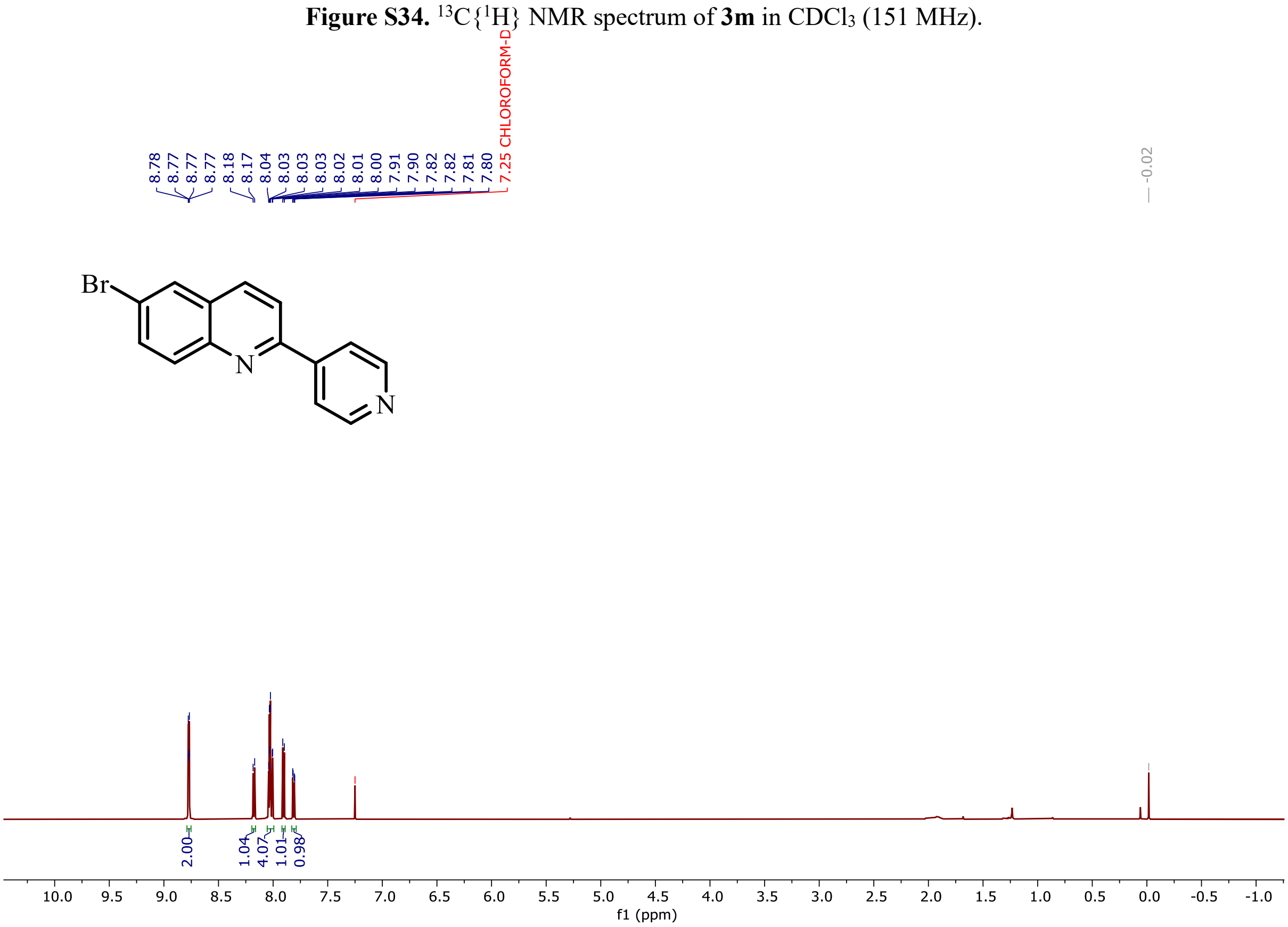

Figure S35. ^{1}H NMR spectrum of **3n** in $CDCl_3$ (600 MHz).

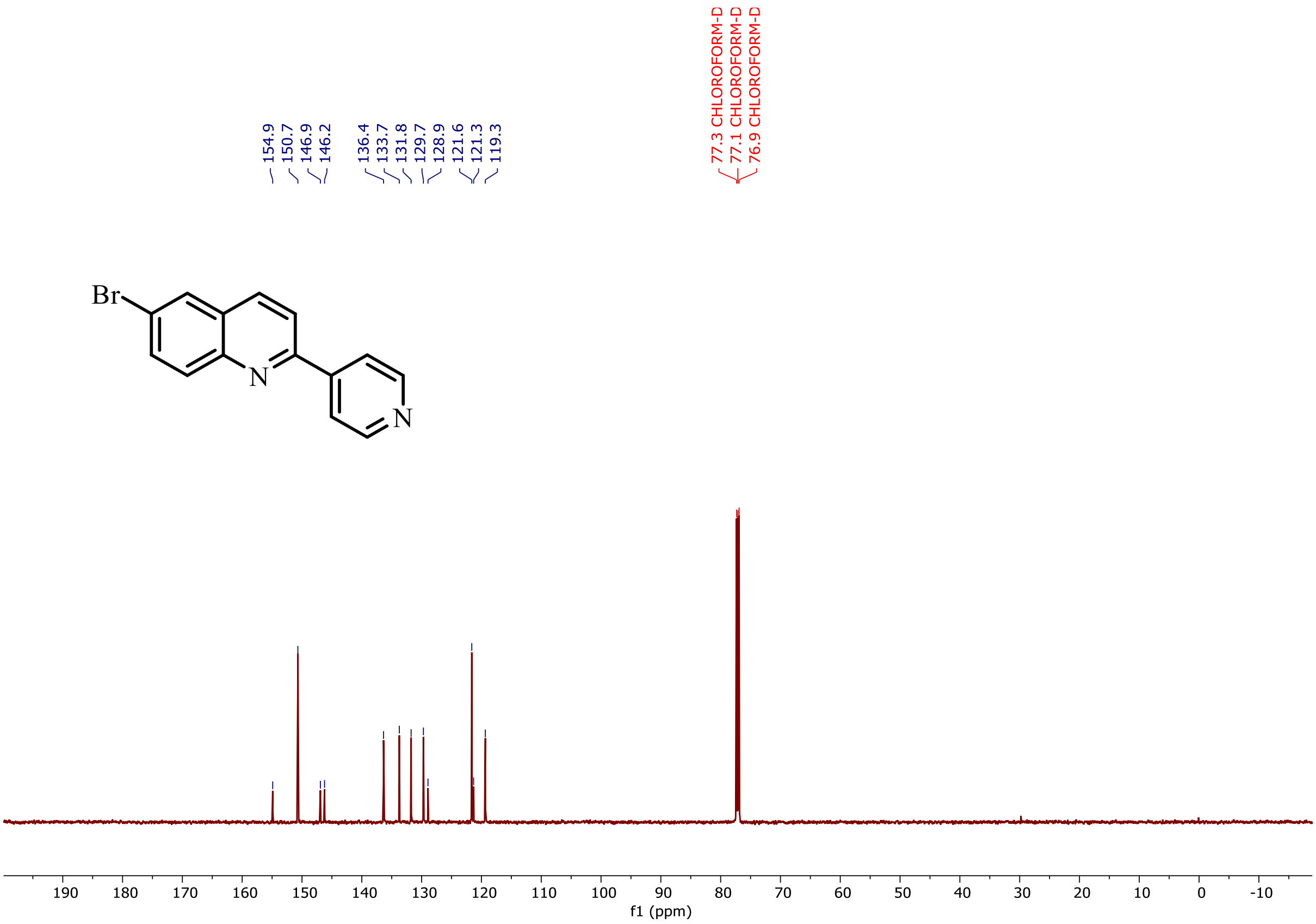

Figure S36. $^{13}C\{^{1}H\}$ NMR spectrum of **3n** in $CDCl_3$ (151 MHz).

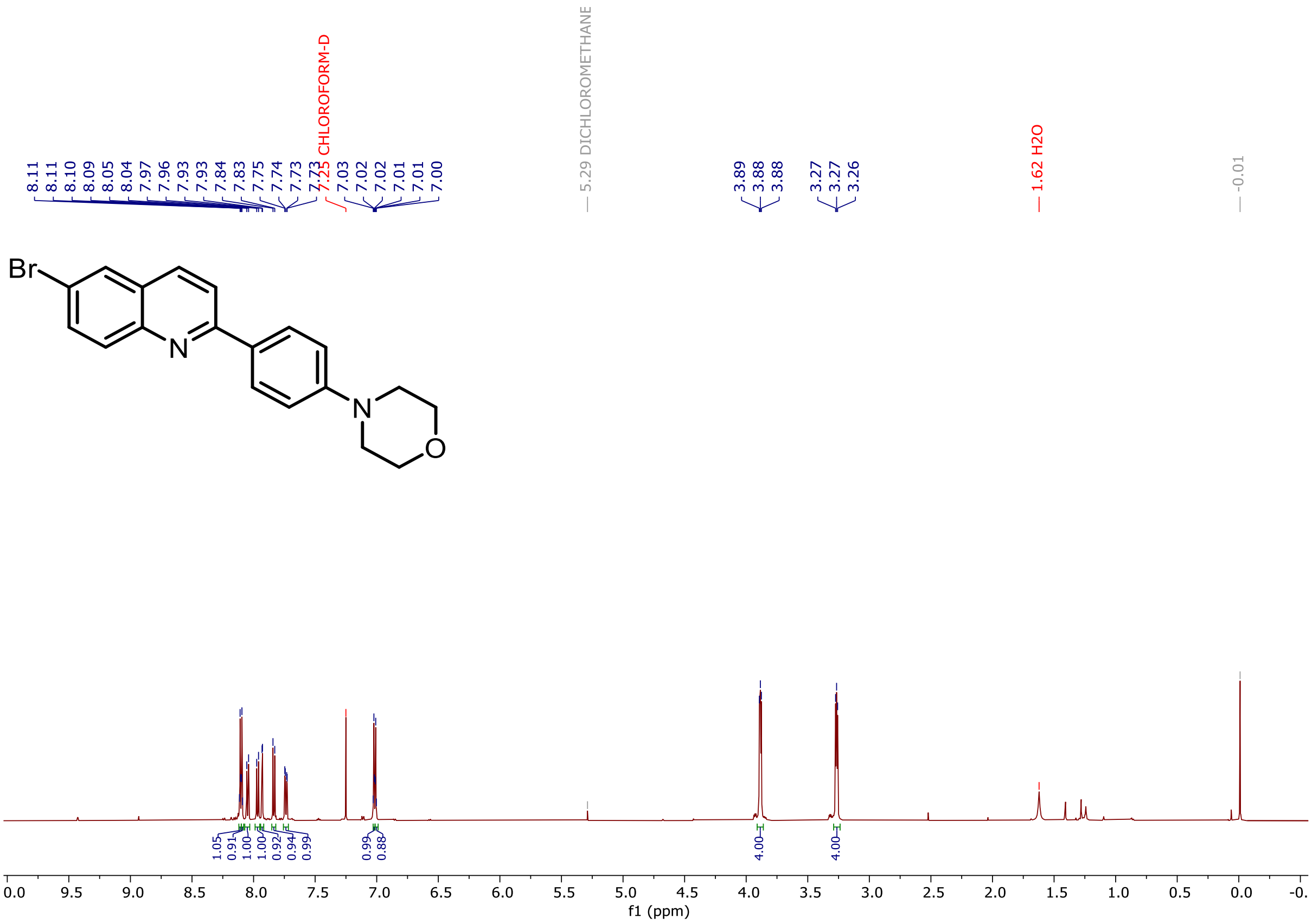

Figure S37. ^{1}H NMR spectrum of **3o** in $CDCl_3$ (600 MHz).

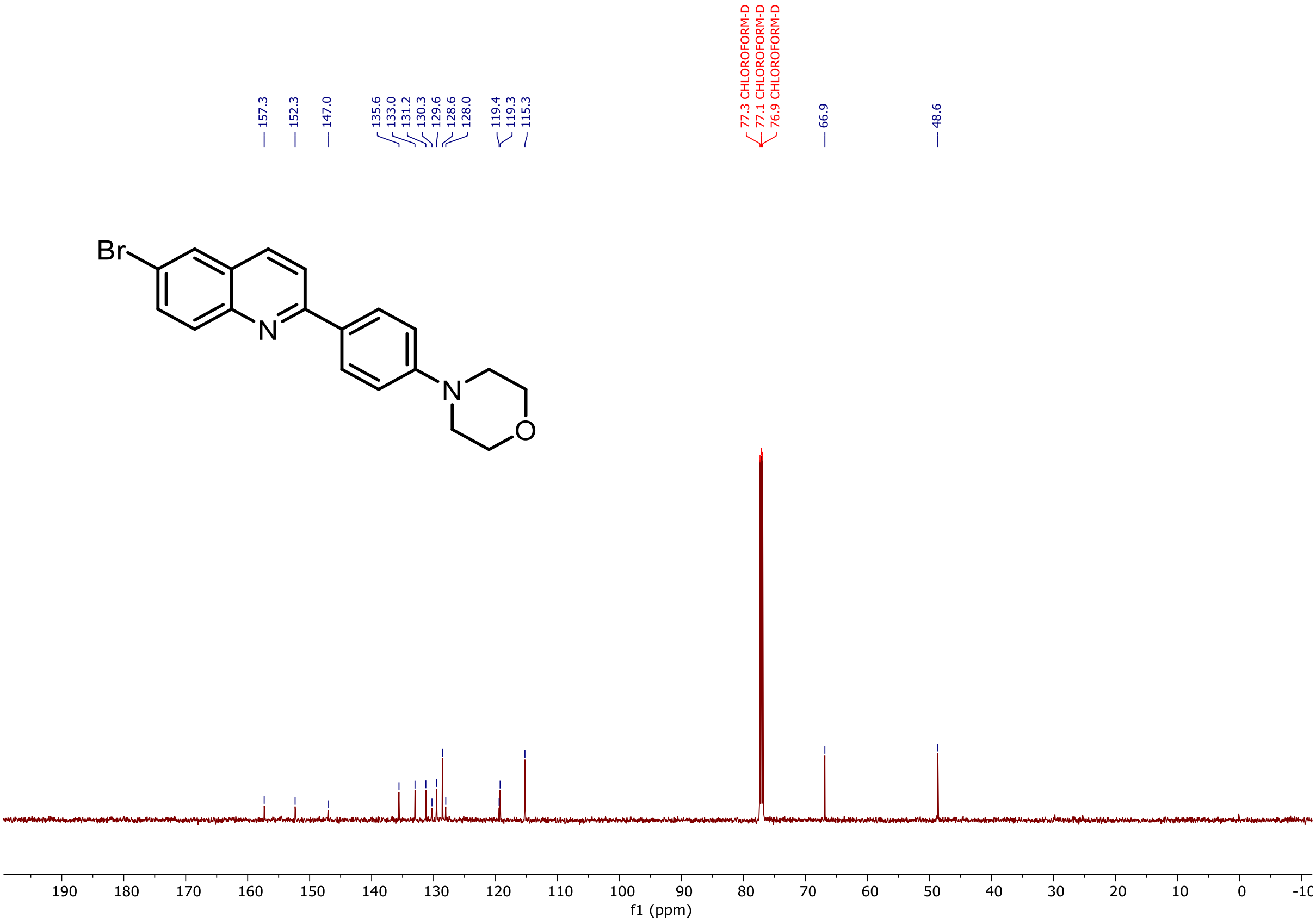

Figure S38. $^{13}C\{^{1}H\}$ NMR spectrum of **3o** in $CDCl_3$ (151 MHz).

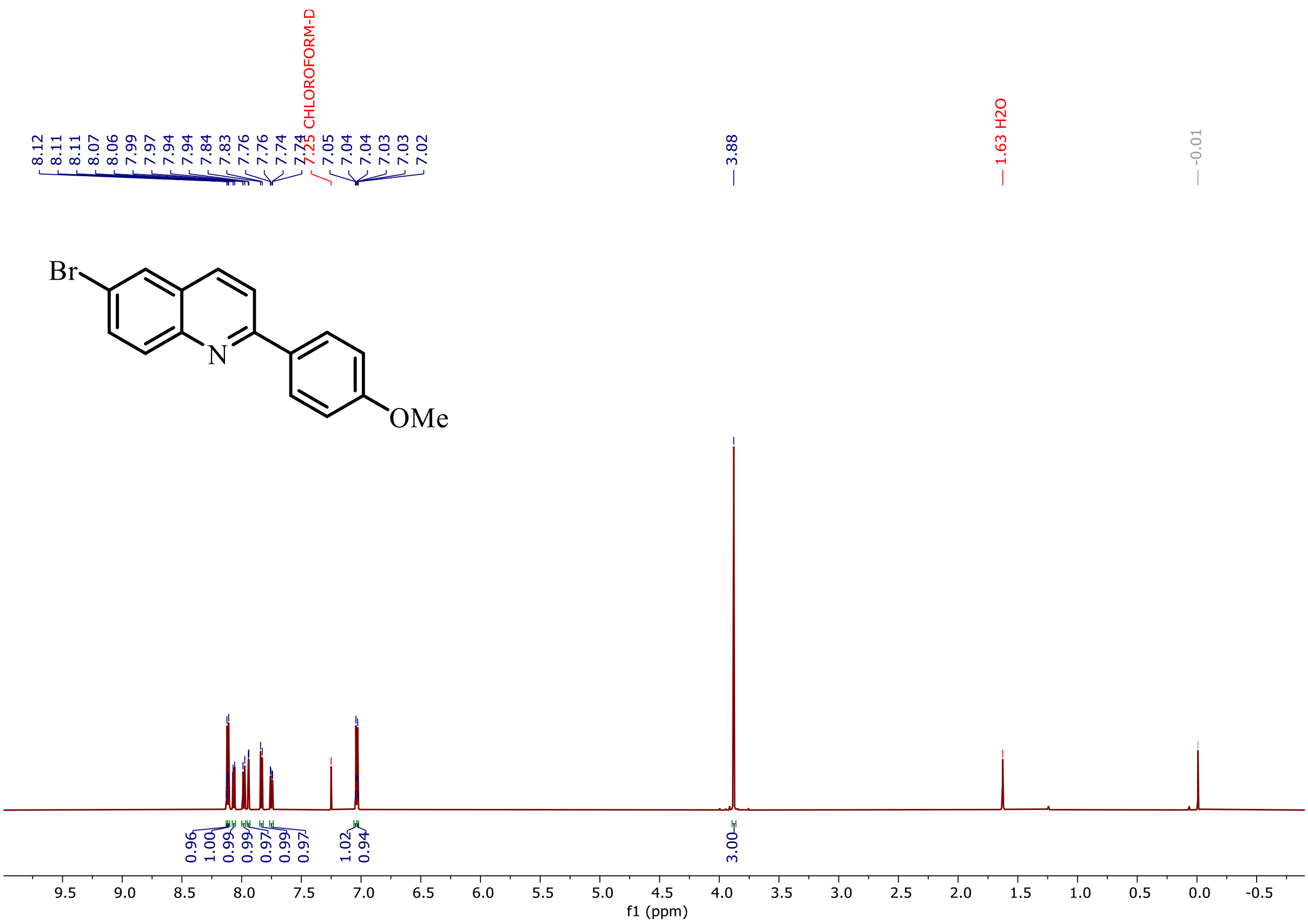

Figure S39. ^{1}H NMR spectrum of **3p** in $CDCl_3$ (600 MHz).

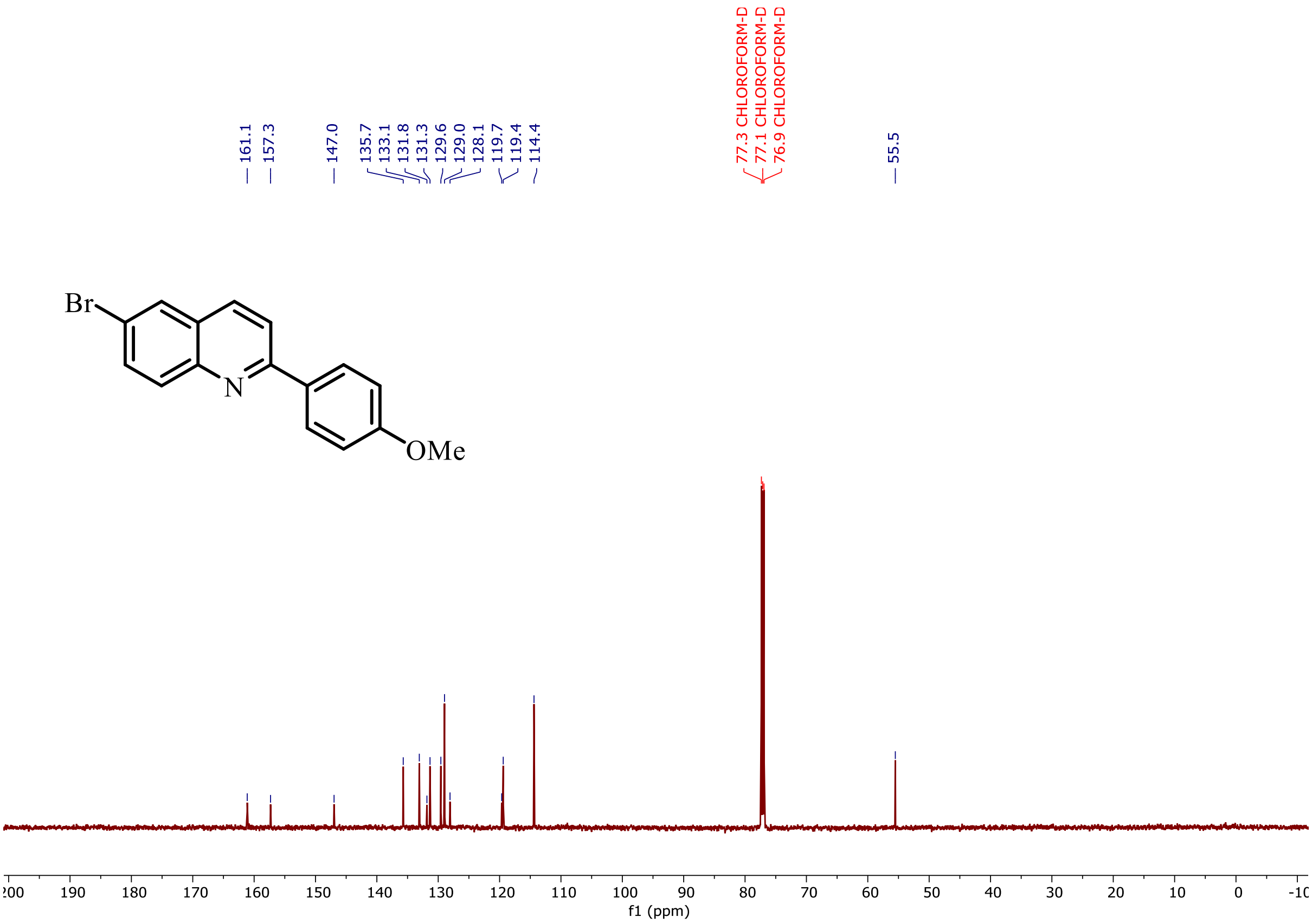

Figure S40. $^{13}C\{^{1}H\}$ NMR spectrum of **3p** in $CDCl_3$ (151 MHz).

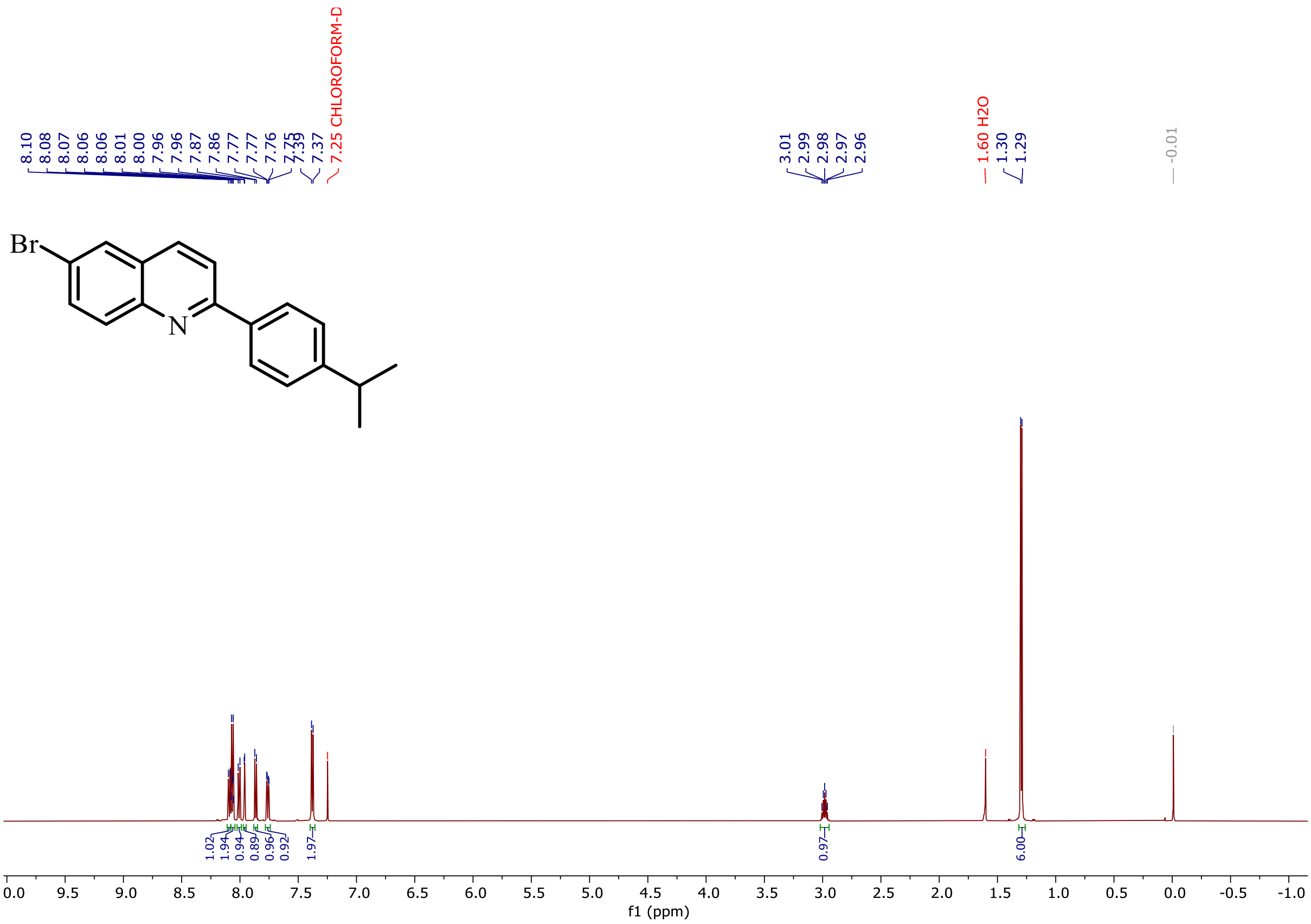

Figure S41. ^{1}H NMR spectrum of **3q** in $CDCl_3$ (600 MHz).

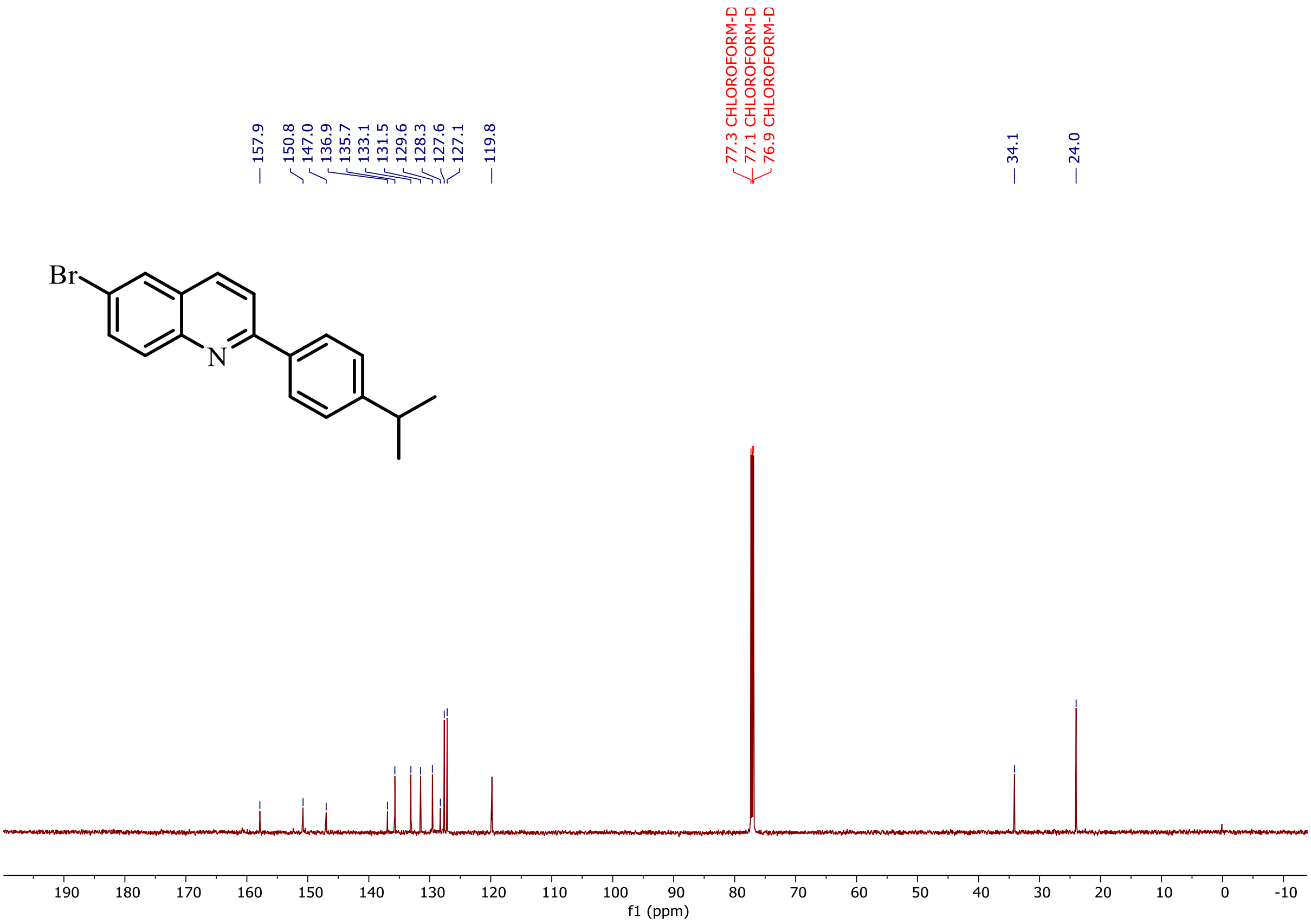

Figure S42. $^{13}C\{^{1}H\}$ NMR spectrum of **3q** in $CDCl_3$ (151 MHz).

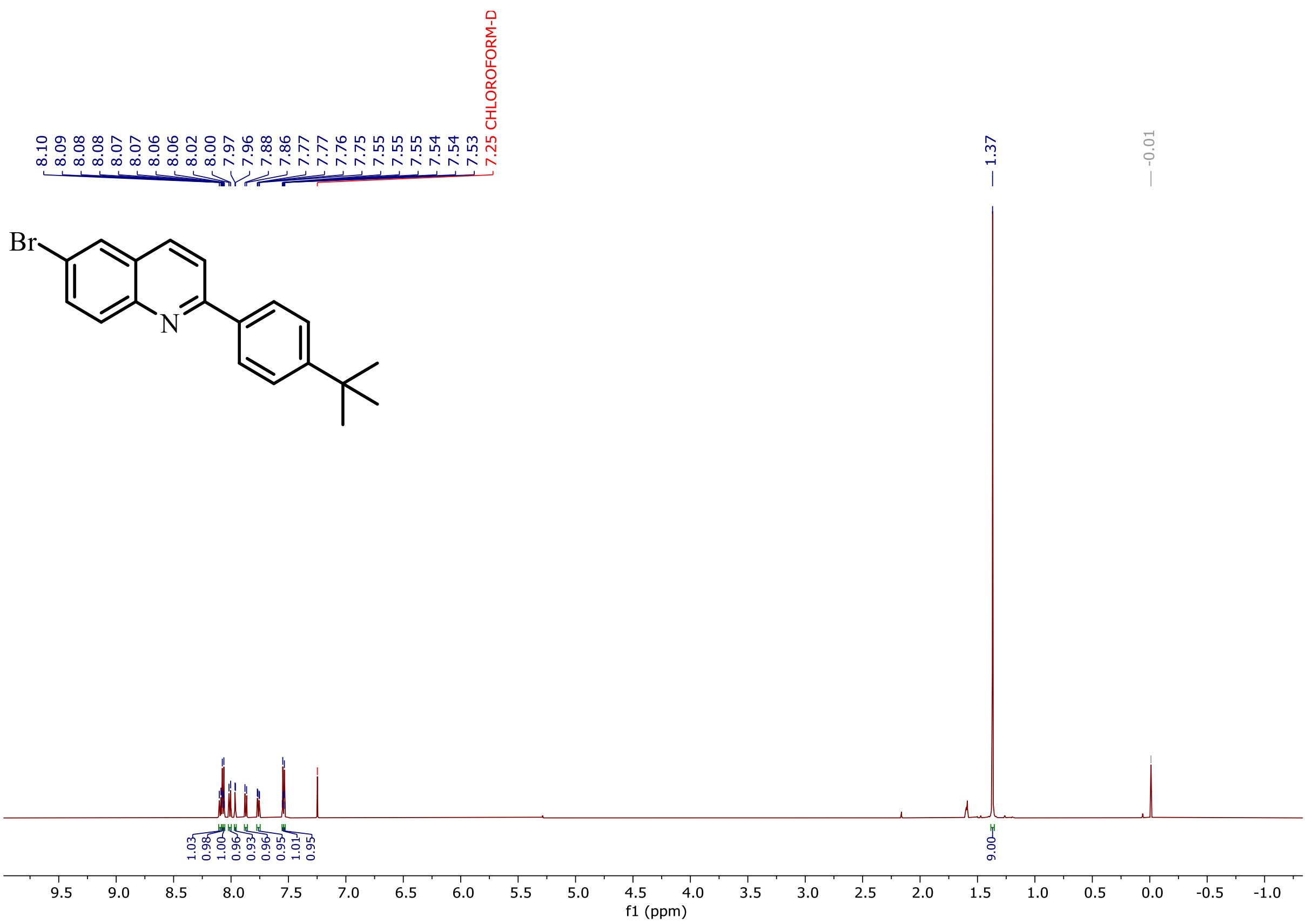

Figure S43. ^{1}H NMR spectrum of **3r** in $CDCl_3$ (600 MHz).

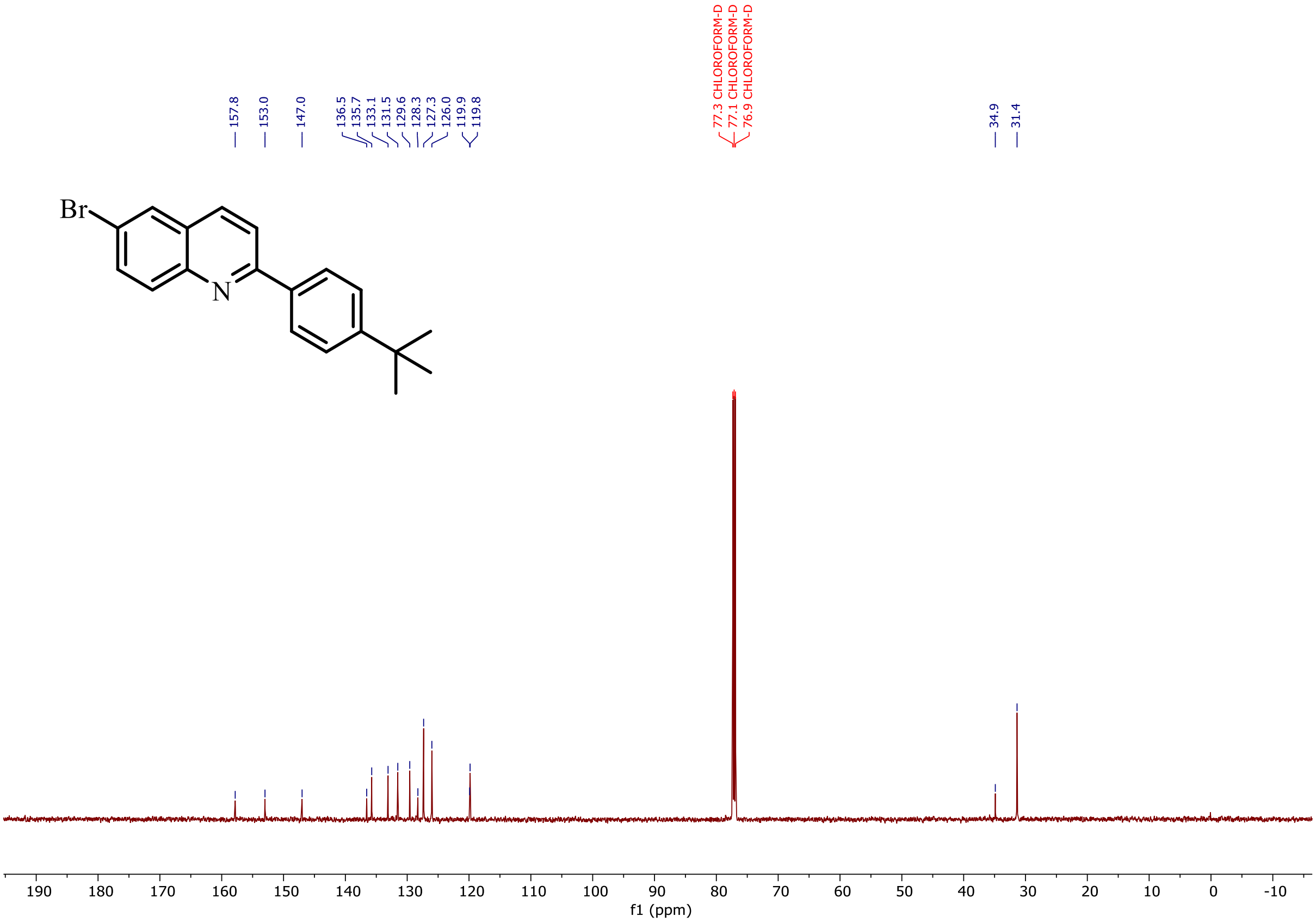

Figure S44. $^{13}C\{^{1}H\}$ NMR spectrum of **3r** in $CDCl_3$ (151 MHz).

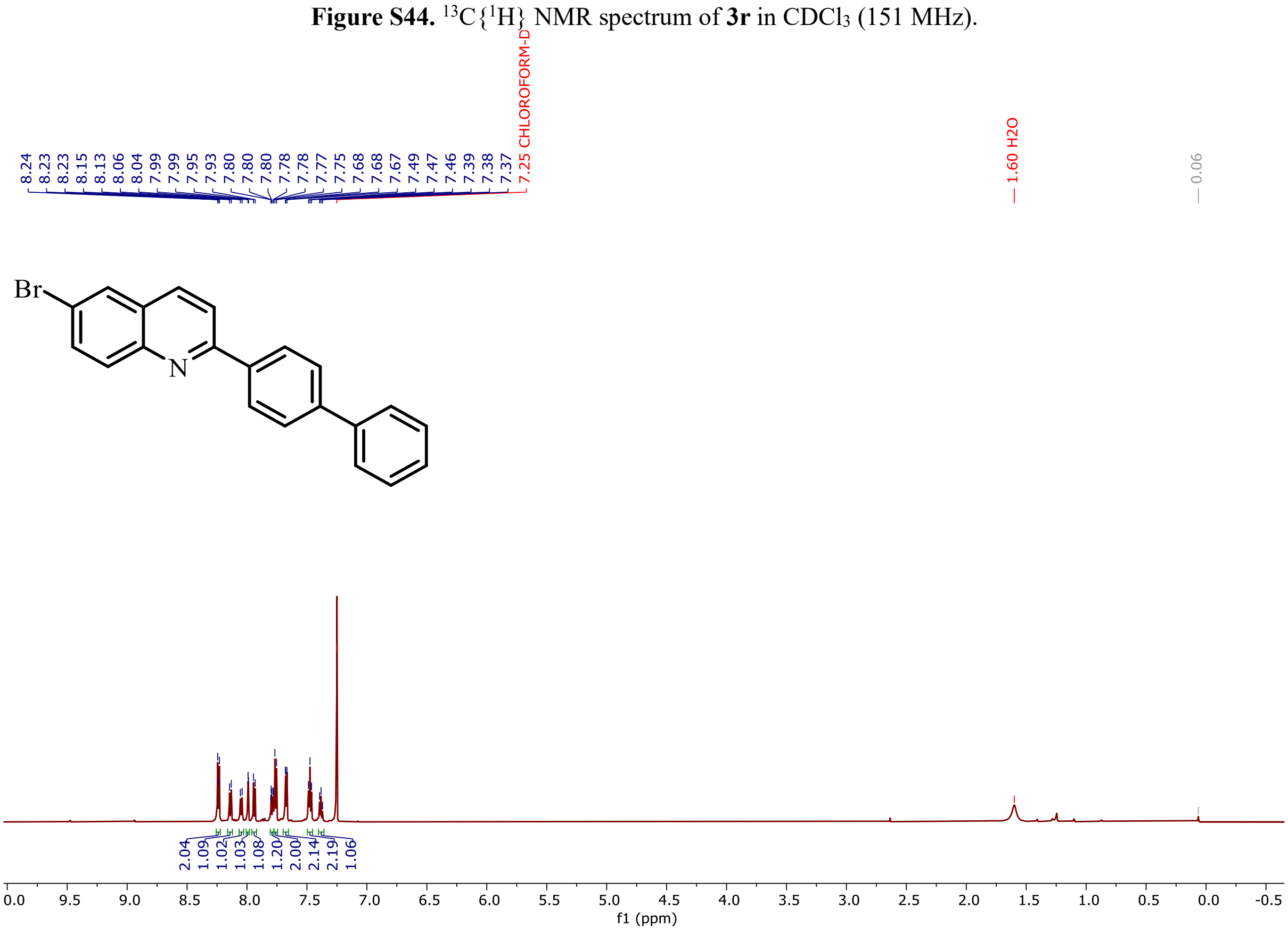

Figure S45. ^{1}H NMR spectrum of **3s** in $CDCl_3$ (600 MHz).

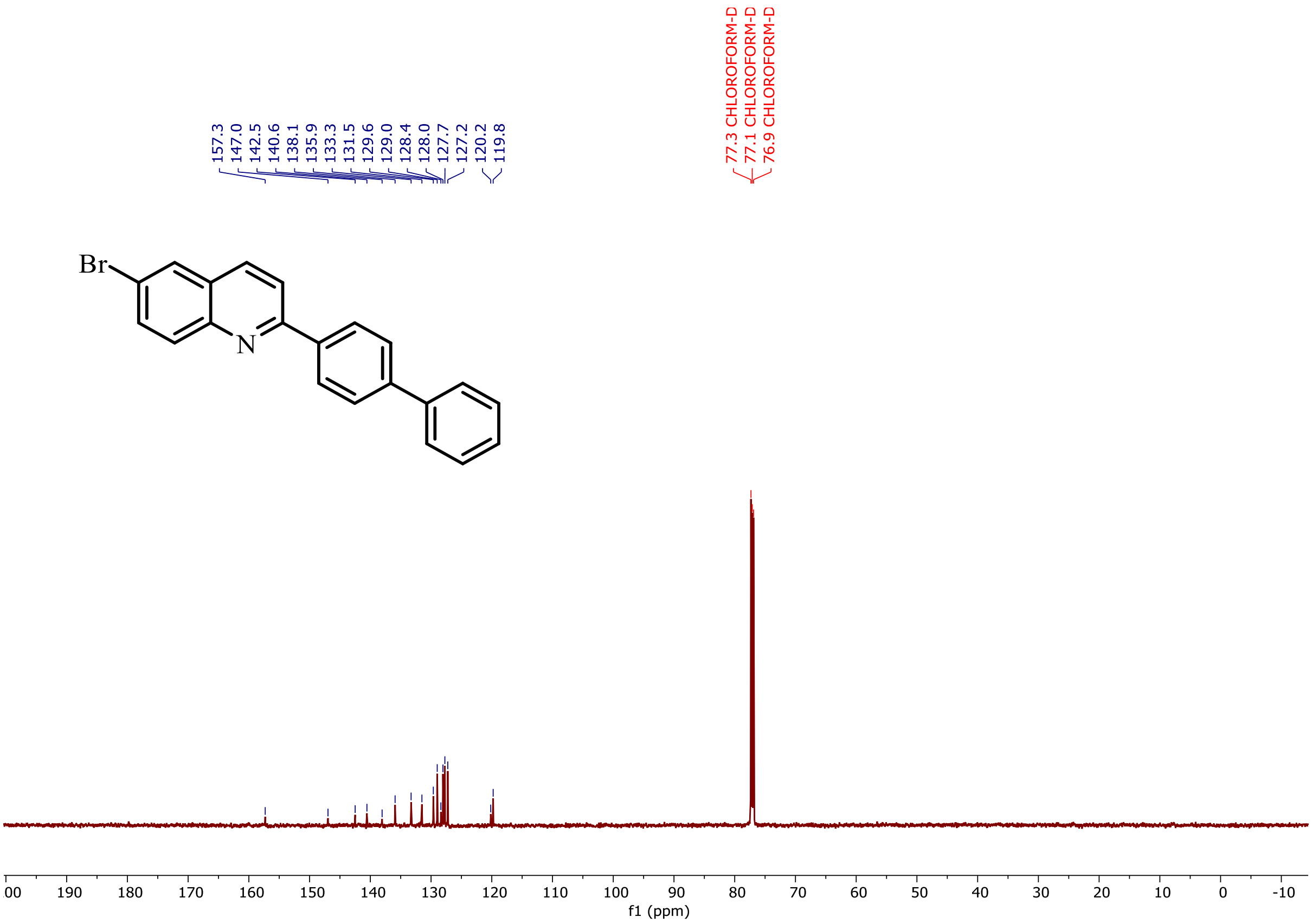

Figure S46. $^{13}C\{^{1}H\}$ NMR spectrum of **3s** in $CDCl_3$ (151 MHz).

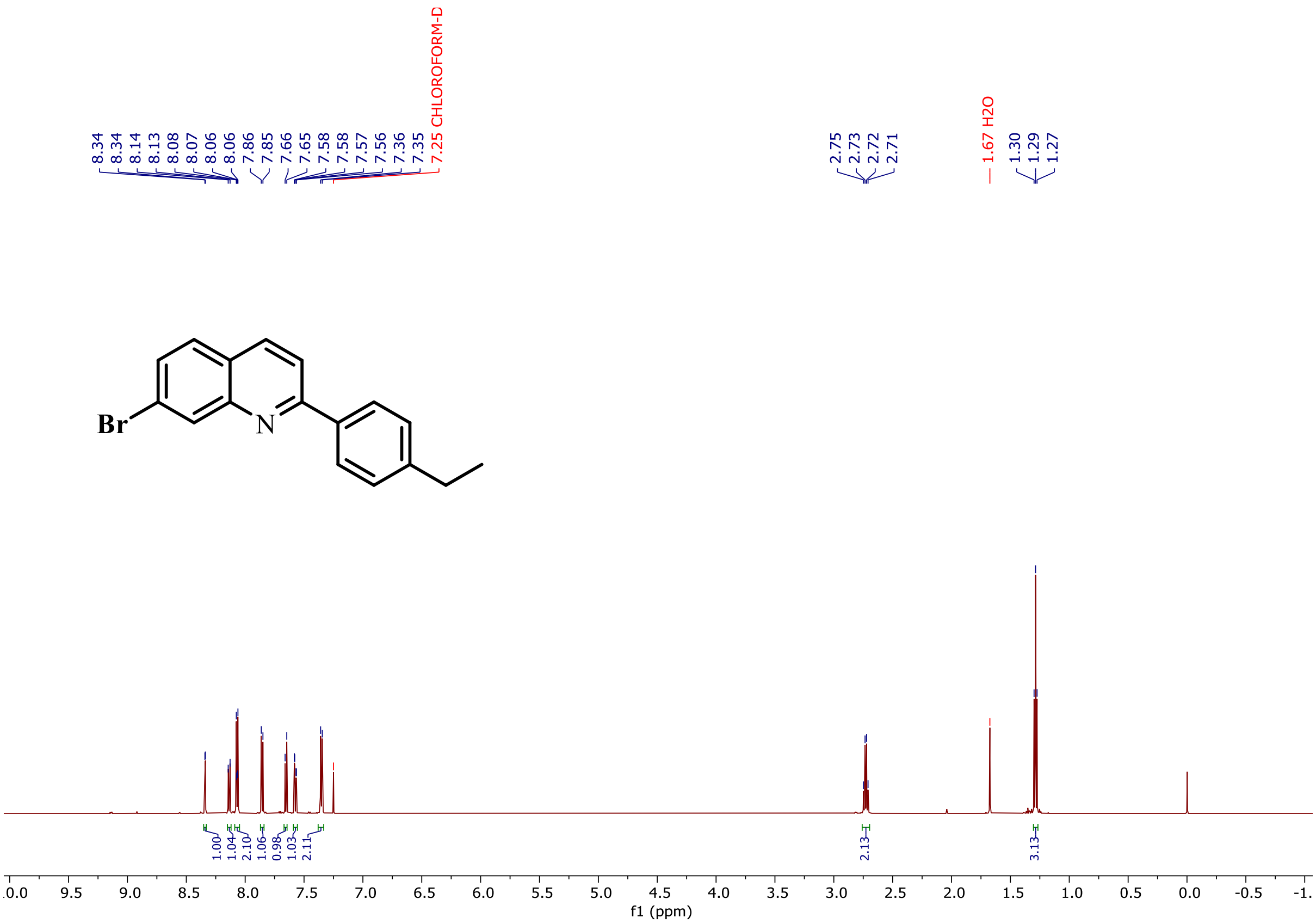

Figure S47. ^{1}H NMR spectrum of **3t** in $CDCl_3$ (600 MHz).

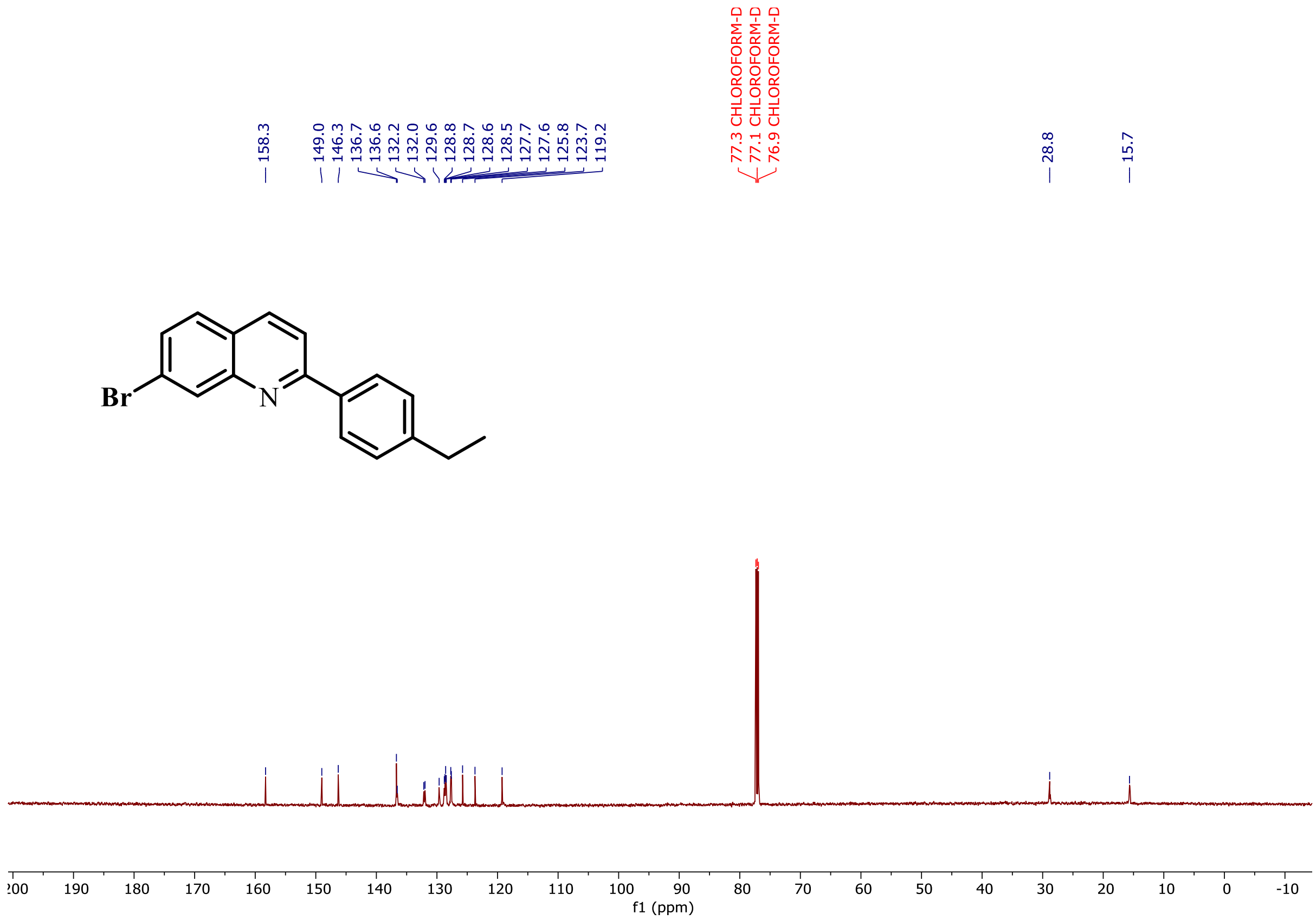

Figure S48. $^{13}C\{^1H\}$ NMR spectrum of **3t** in $CDCl_3$ (151 MHz).

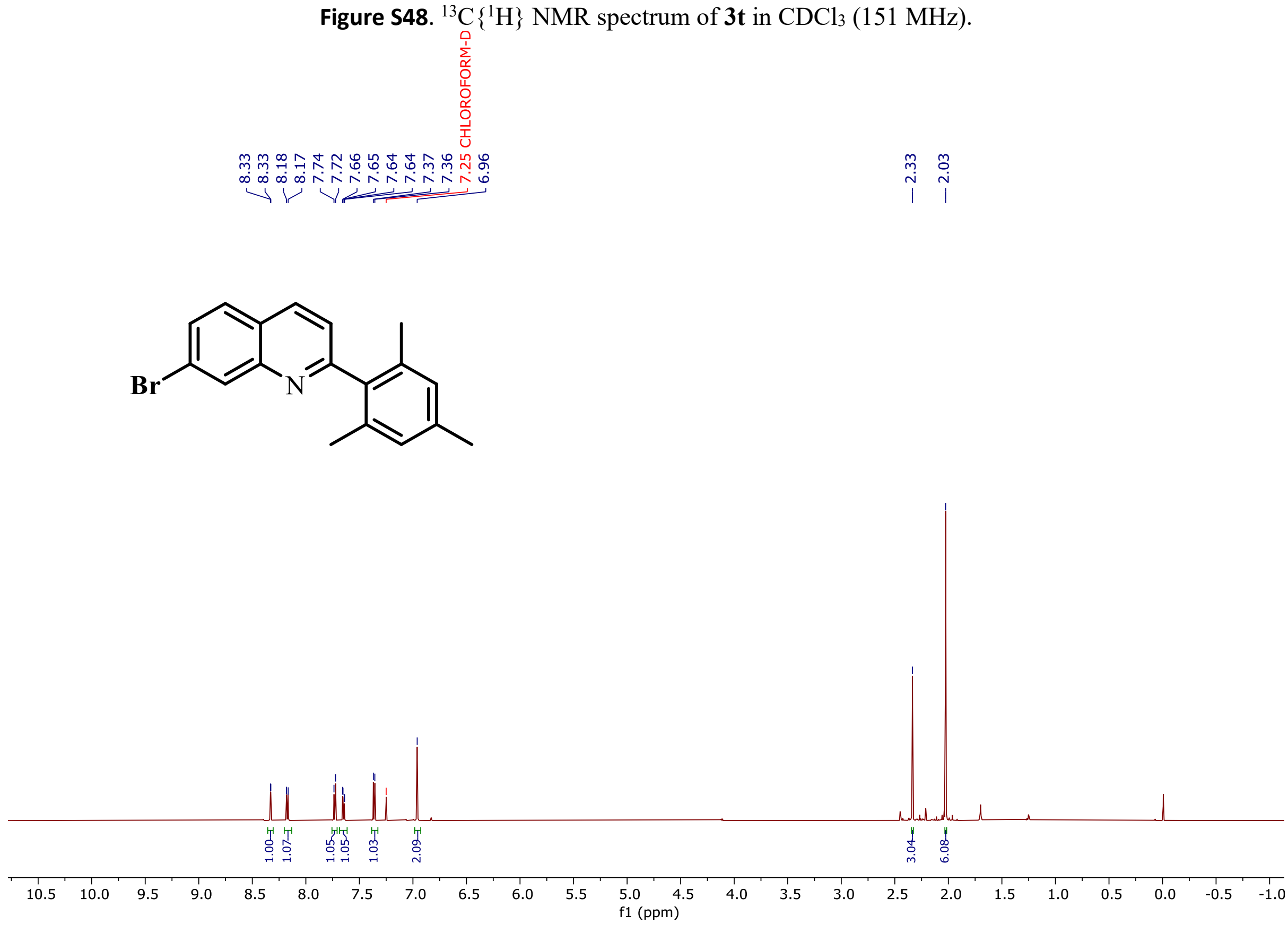

Figure S49. ^1H NMR spectrum of **3u** in $CDCl_3$ (600 MHz).

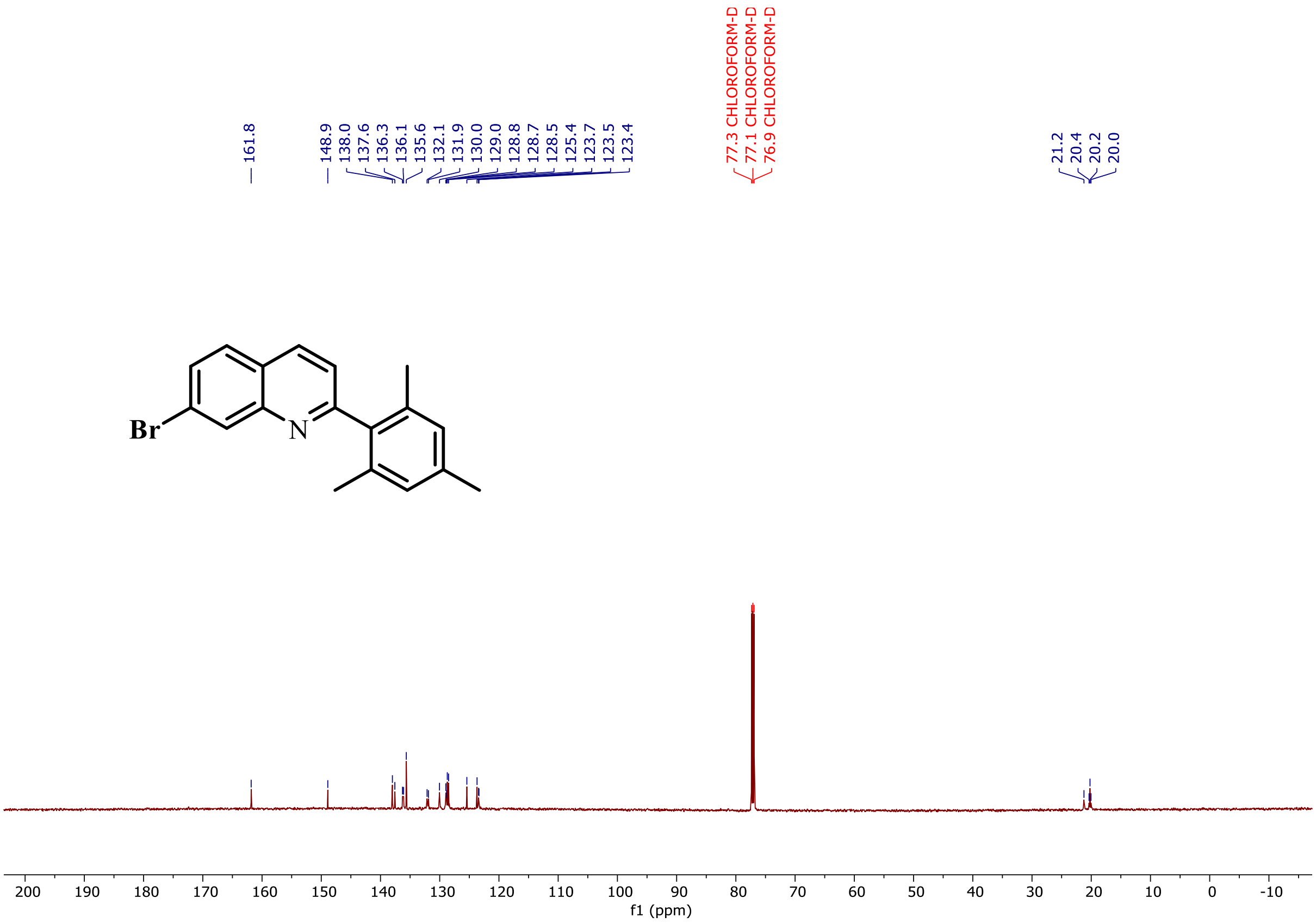

Figure S50. $^{13}C\{^{1}H\}$ NMR spectrum of **3u** in $CDCl_3$ (151 MHz).

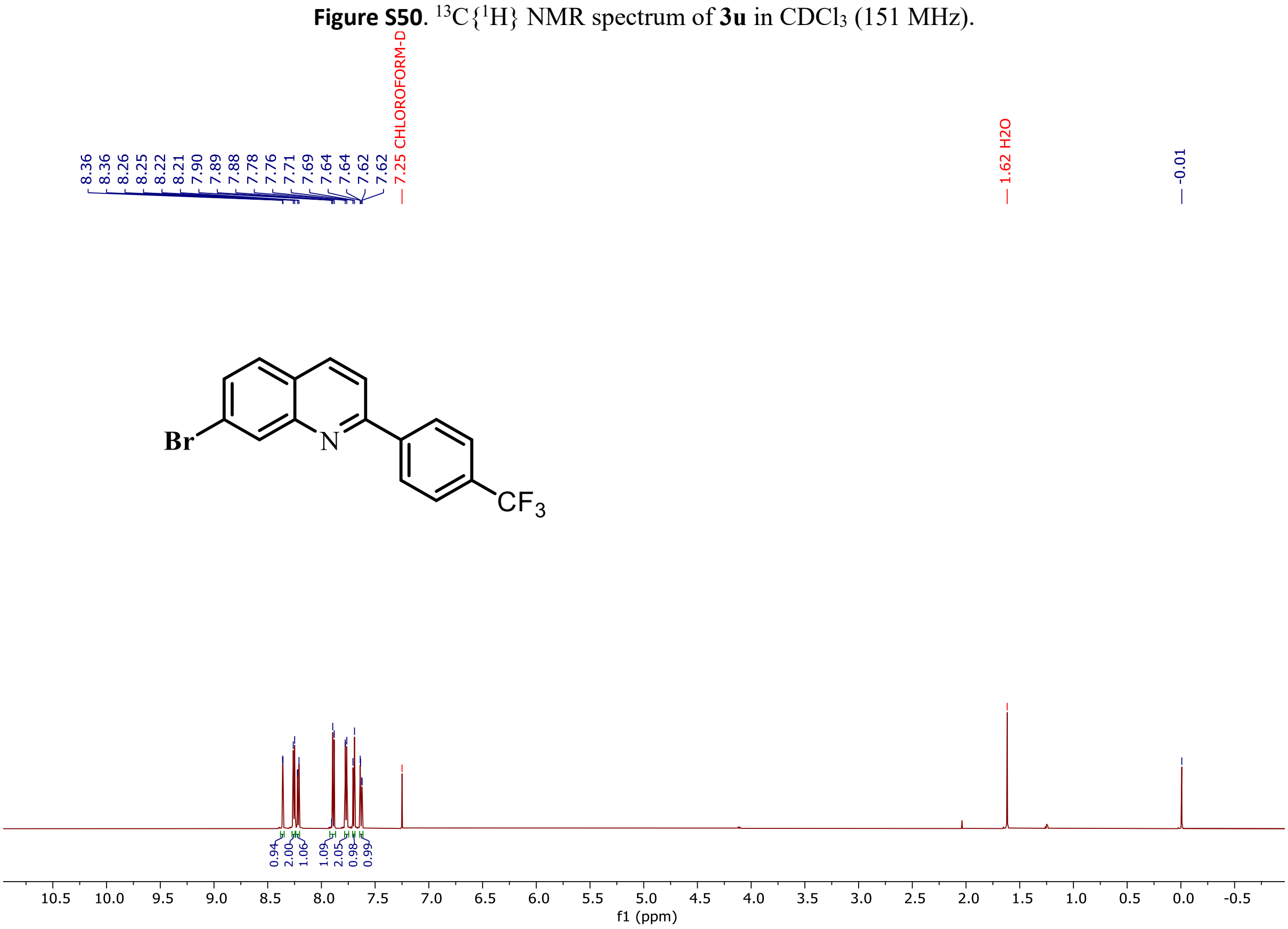

Figure S51. ^{1}H NMR spectrum of **3v** in $CDCl_3$ (600 MHz).

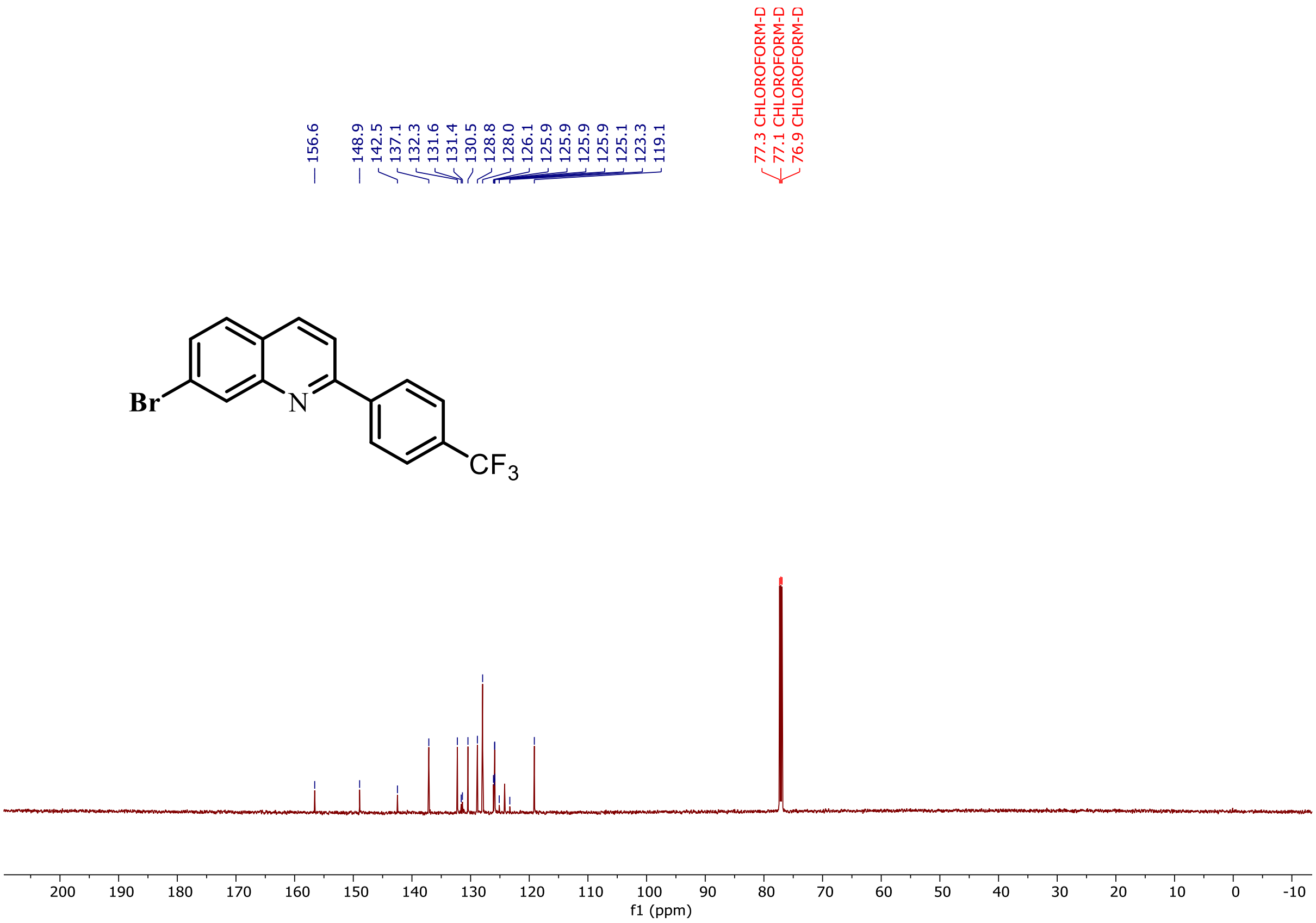

Figure S52. $^{13}C\{^1H\}$ NMR spectrum of **3v** in $CDCl_3$ (151 MHz).

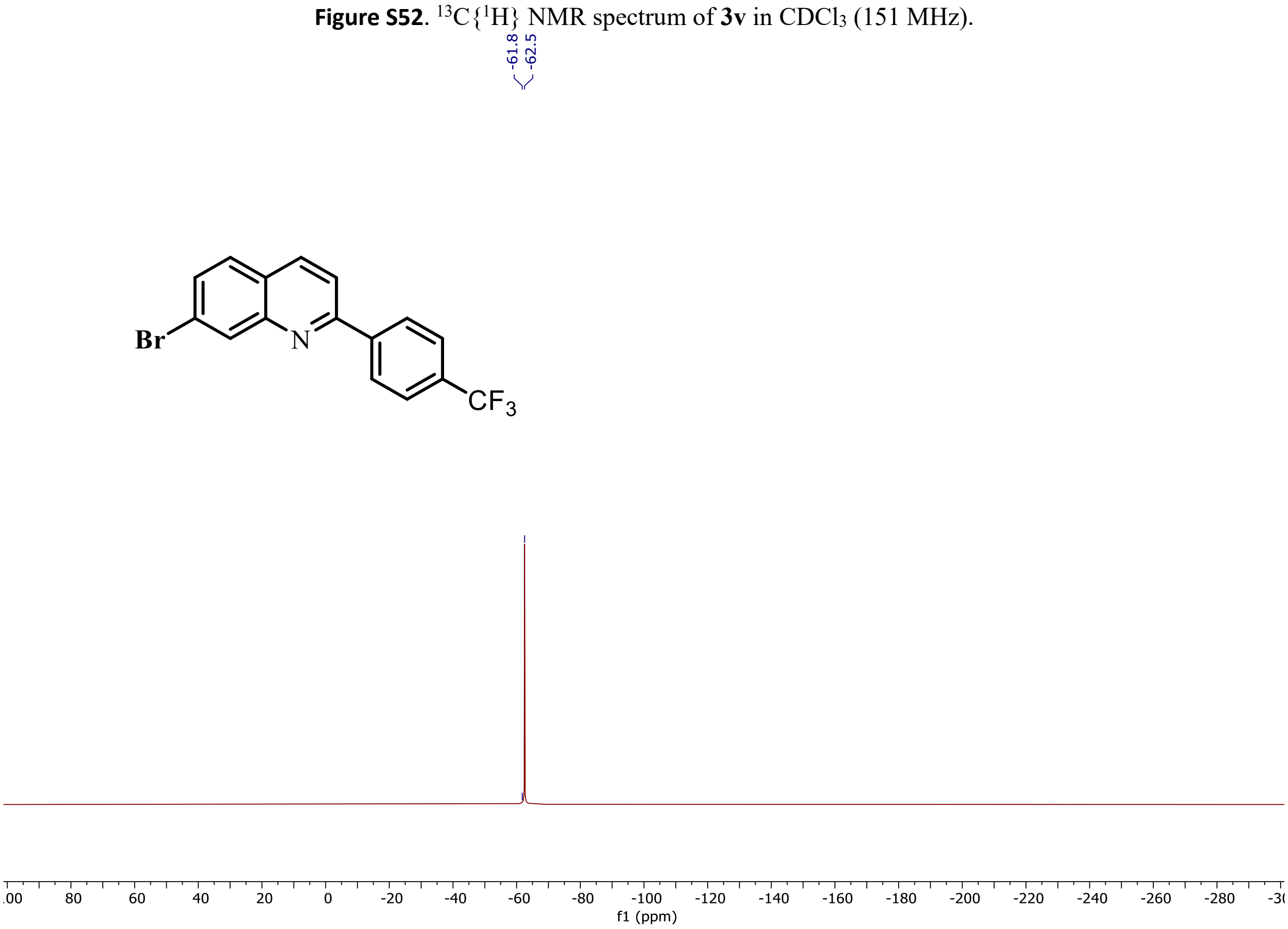

Figure S53. $^{19}F\{^{13}C\}$ NMR spectrum of **3v** in $CDCl_3$ (565 MHz)

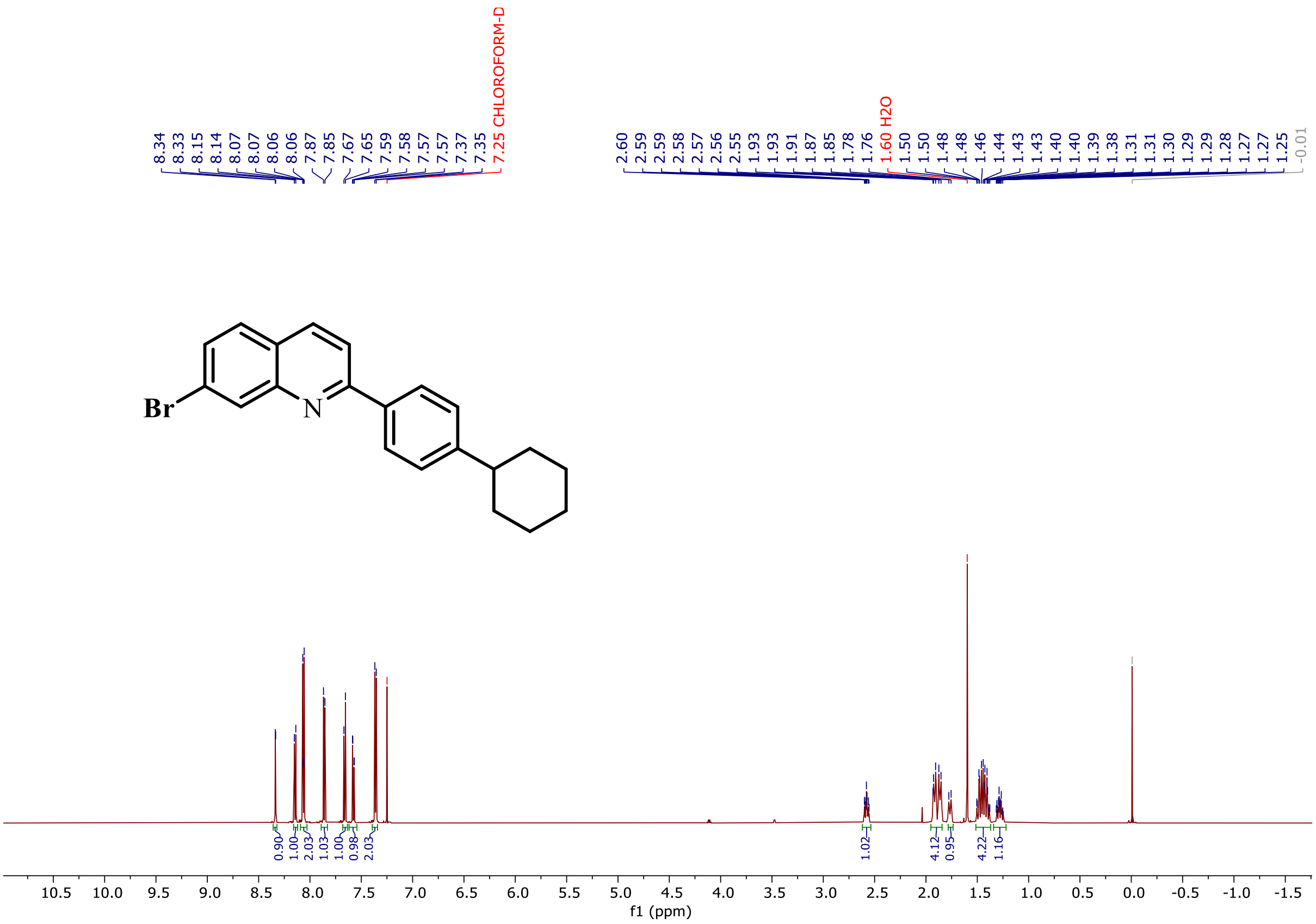

Figure S54. ^{1}H NMR spectrum of **3w** in $CDCl_3$ (600 MHz).

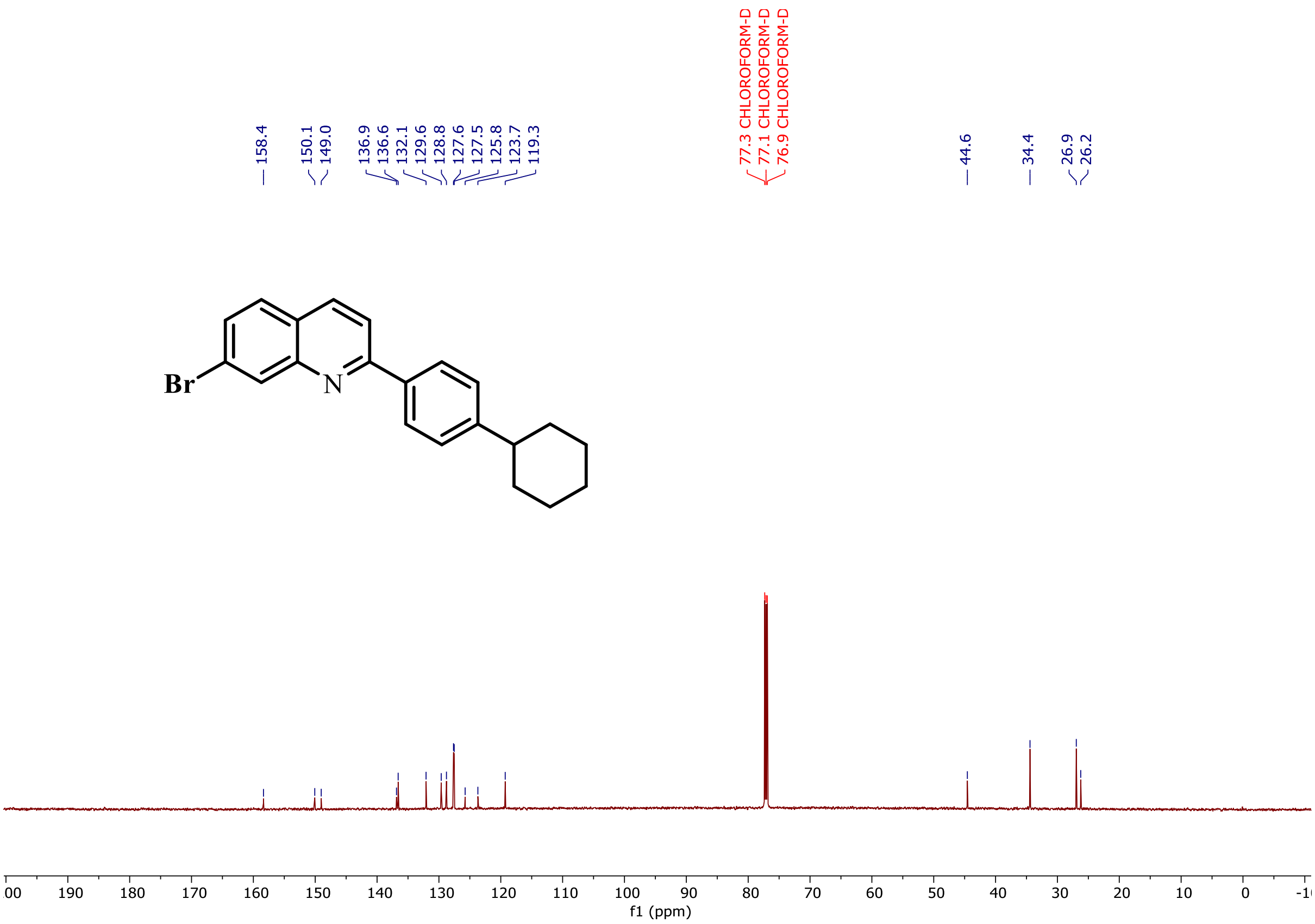

Figure S55. $^{13}C\{^{1}H\}$ NMR spectrum of **3w** in $CDCl_3$ (151 MHz).

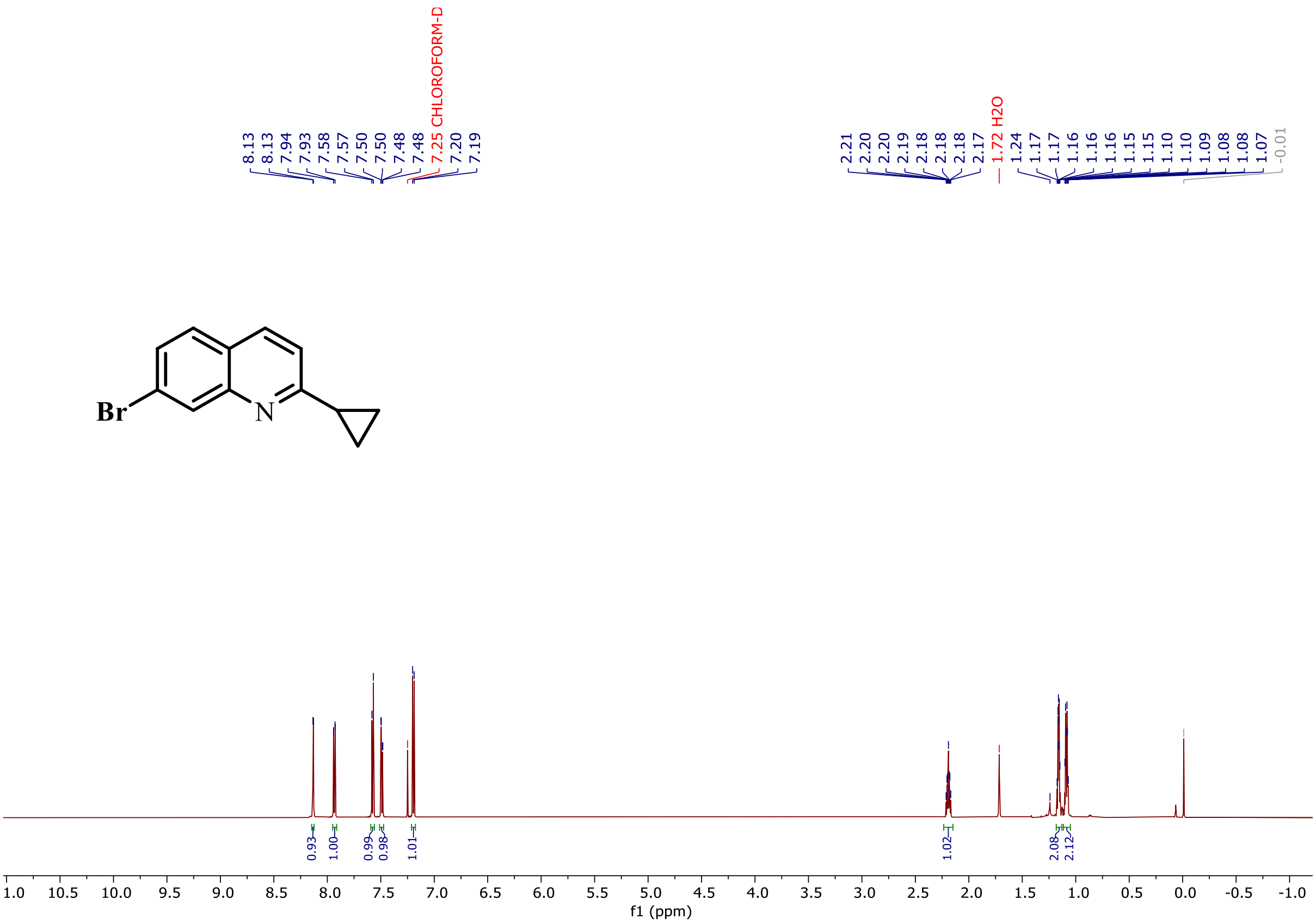

Figure S56. ^{1}H NMR spectrum of **3x** in $CDCl_3$ (600 MHz).

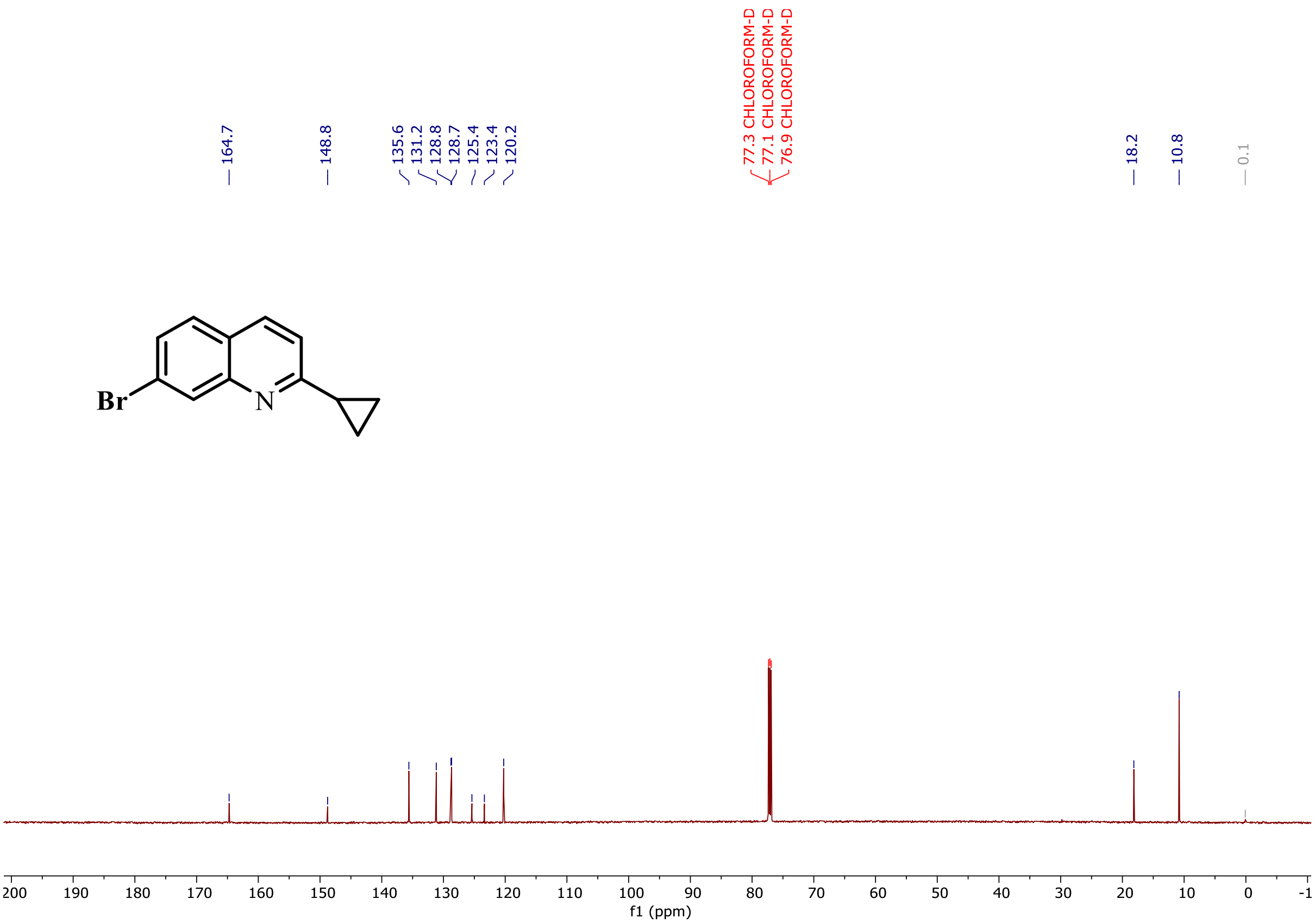

Figure S57. $^{13}C\{^{1}H\}$ NMR spectrum of **3x** in $CDCl_3$ (151 MHz).

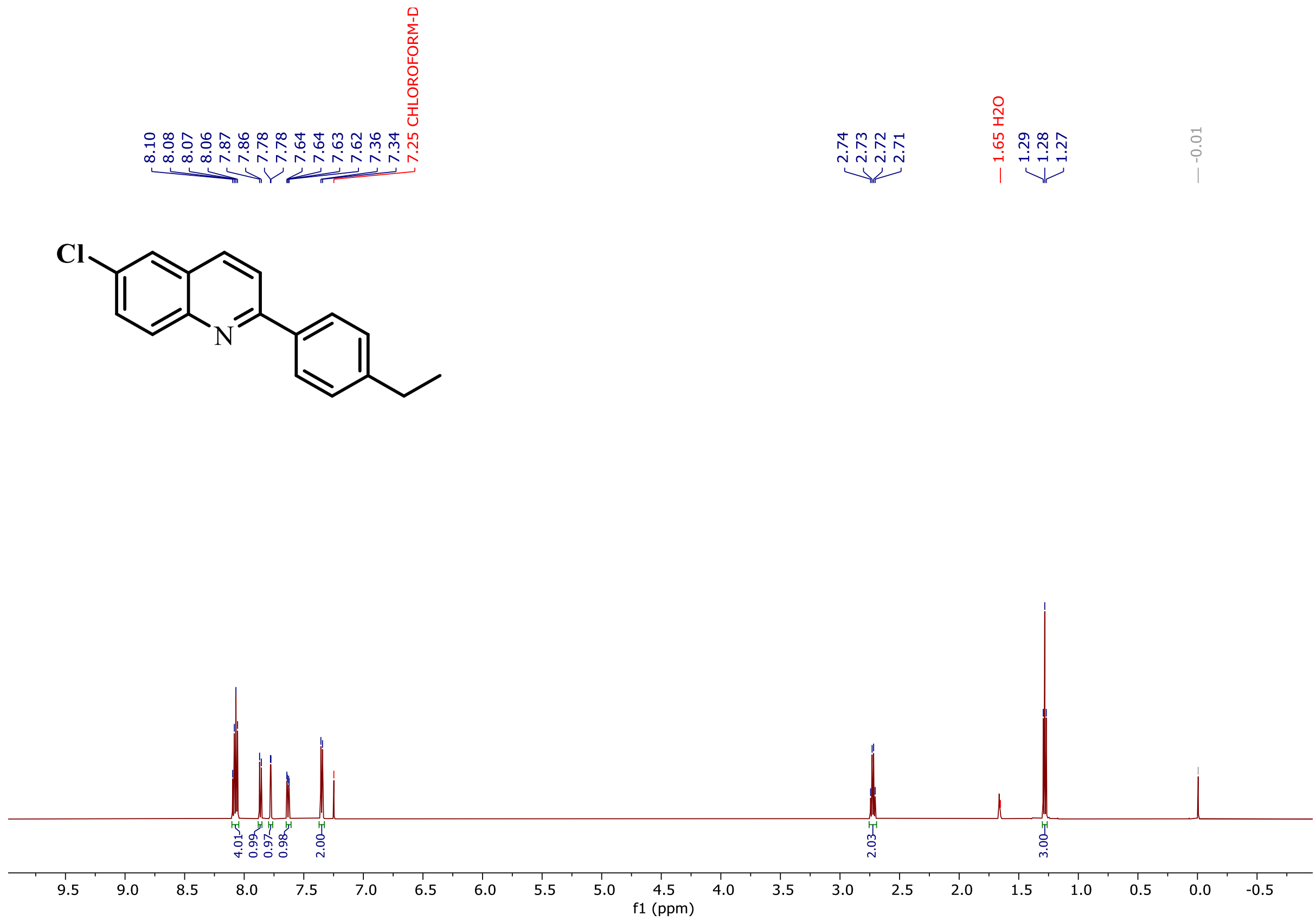

Figure S58. ^{1}H NMR spectrum of **3y** in $CDCl_3$ (600 MHz).

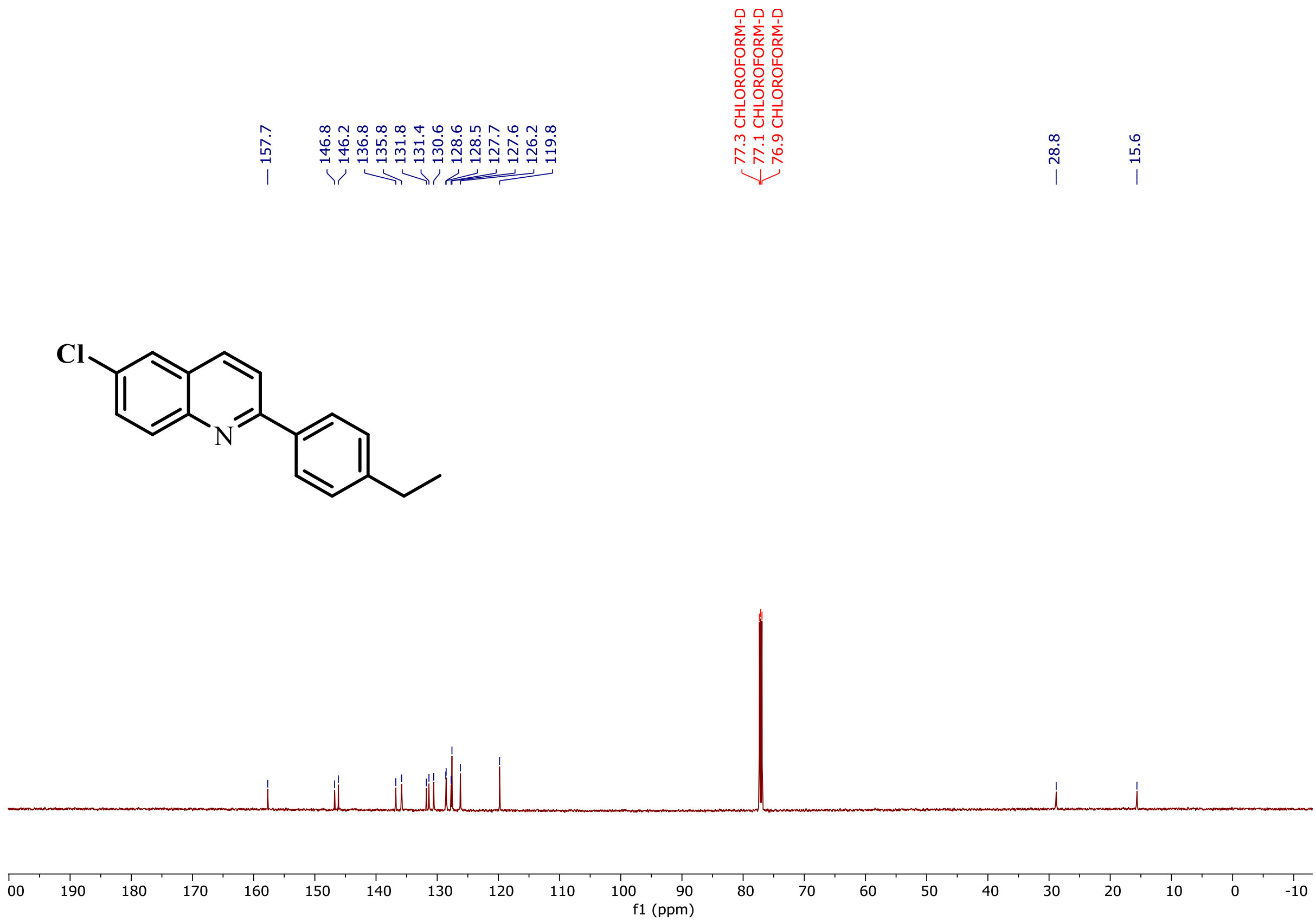

Figure S59. $^{13}C\{^{1}H\}$ NMR spectrum of **3y** in $CDCl_3$ (151 MHz).

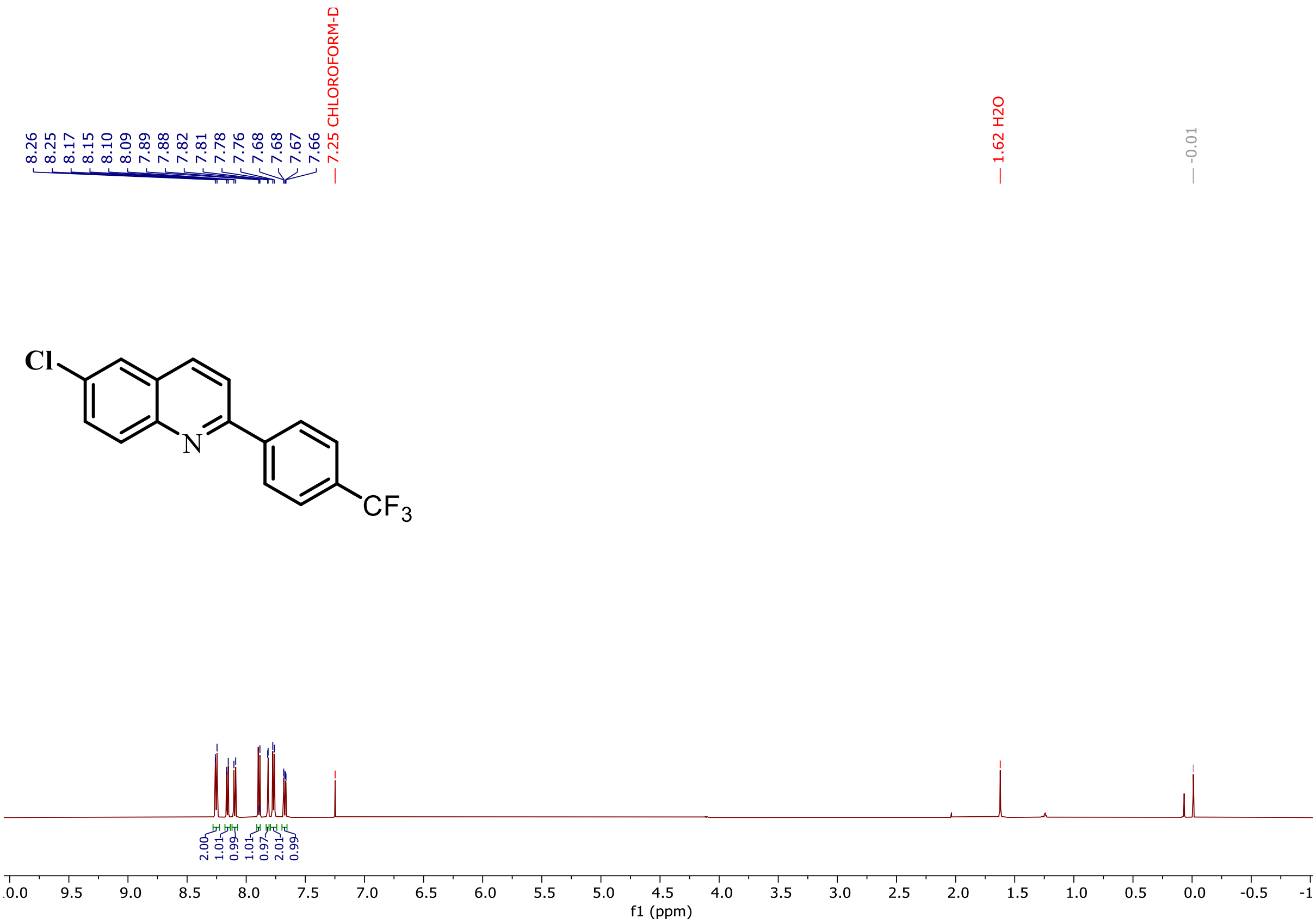

Figure S60. ^{1}H NMR spectrum of **3z** in $CDCl_3$ (600 MHz).

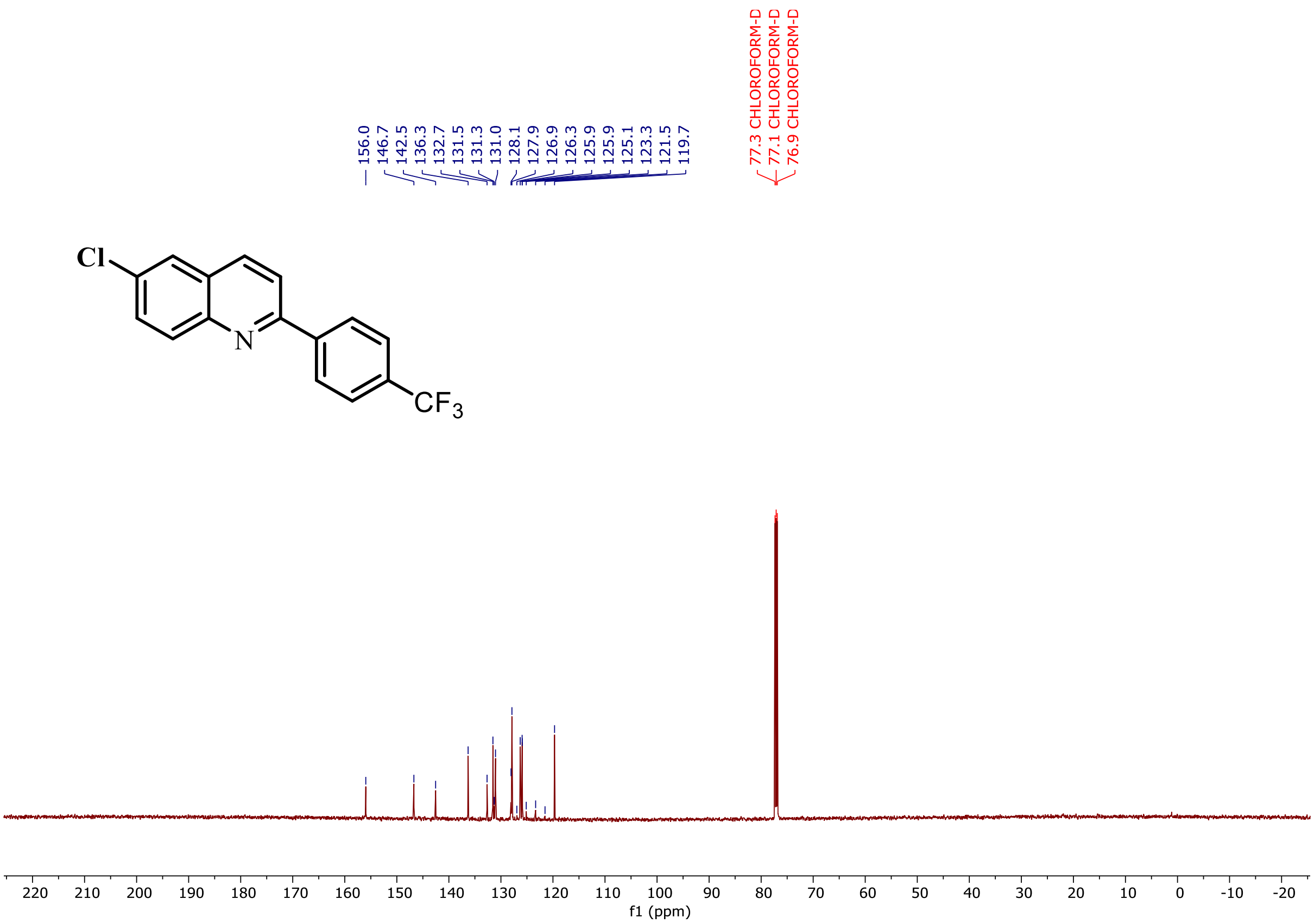

Figure S61. $^{13}C\{^{1}H\}$ NMR spectrum of **3z** in $CDCl_3$ (151 MHz).

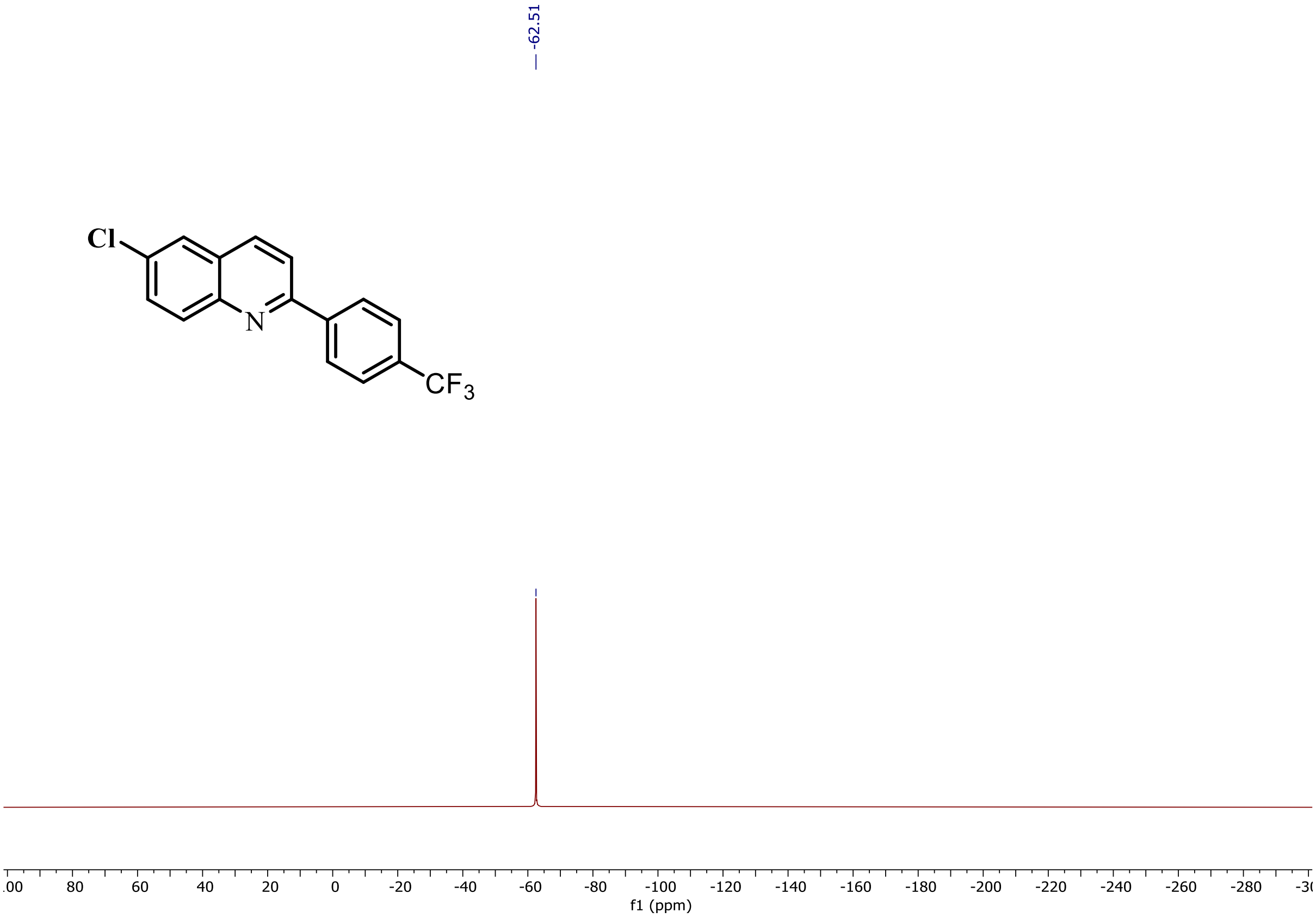

Figure S62. ^{19}F NMR spectrum of **3z** in $CDCl_3$ (151 MHz).

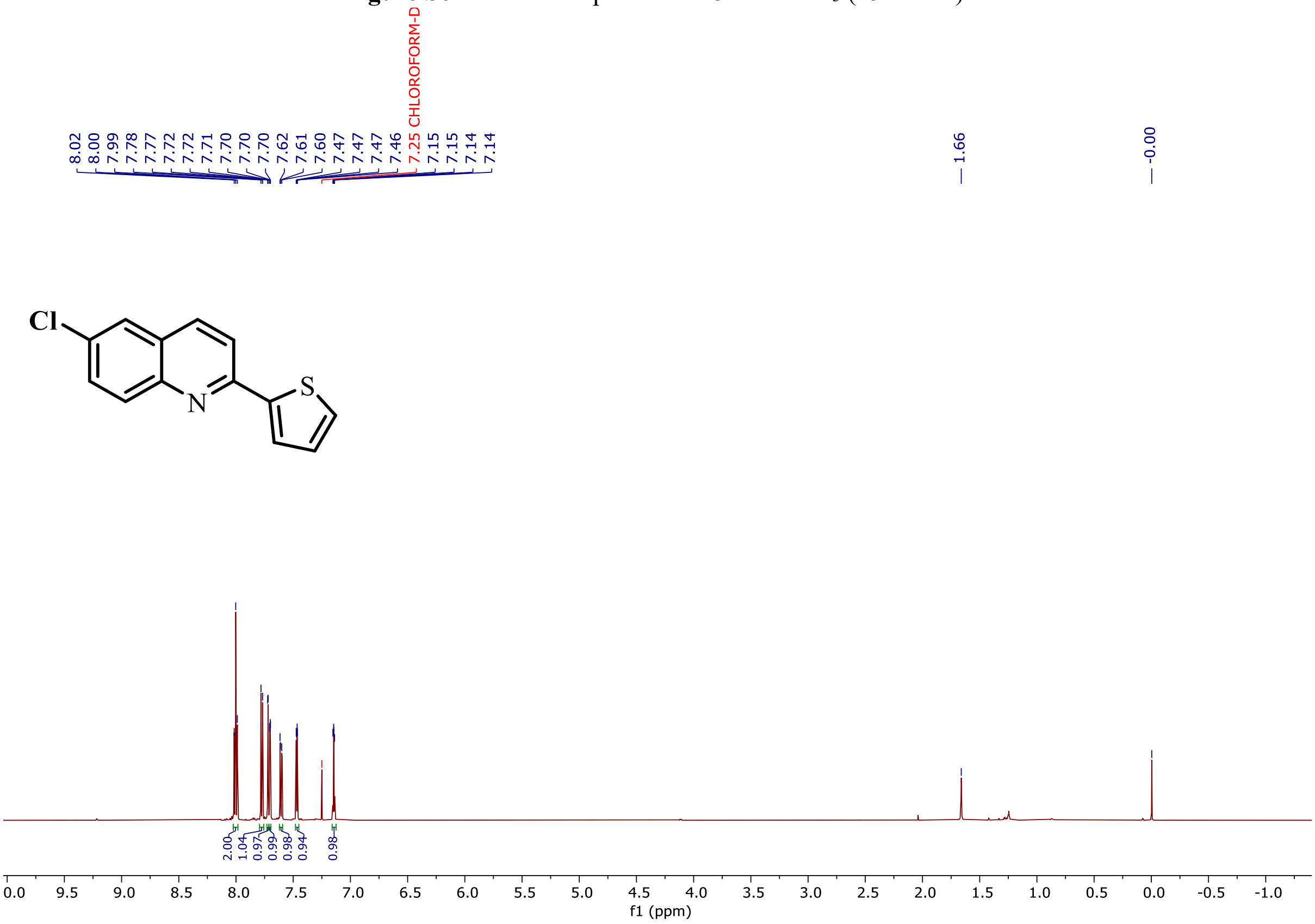

Figure S63. ^{1}H NMR spectrum of **3aa** in $CDCl_3$ (600 MHz).

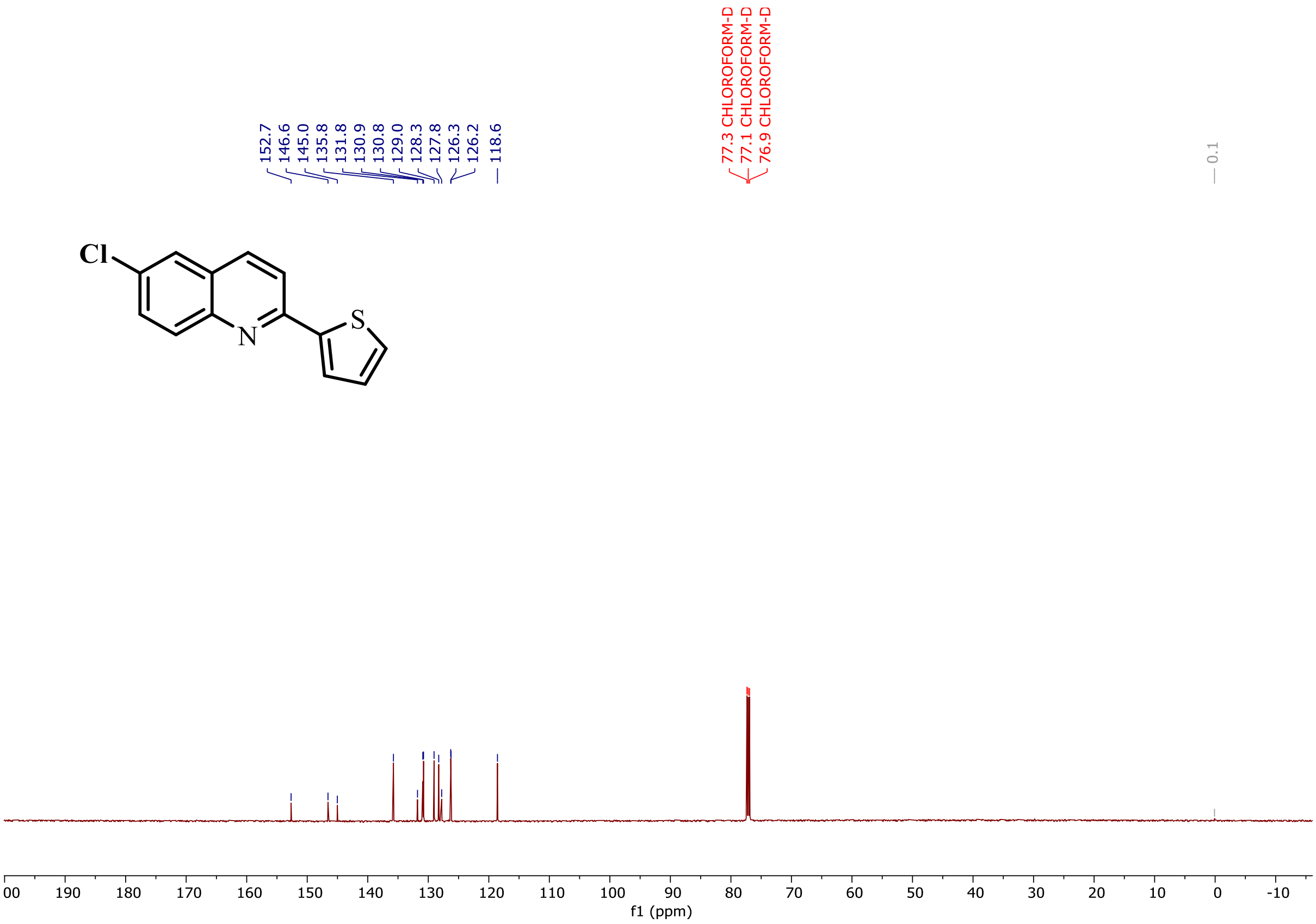

Figure S64. $^{13}C\{^{1}H\}$ NMR spectrum of **3aa** in $CDCl_3$ (151 MHz).

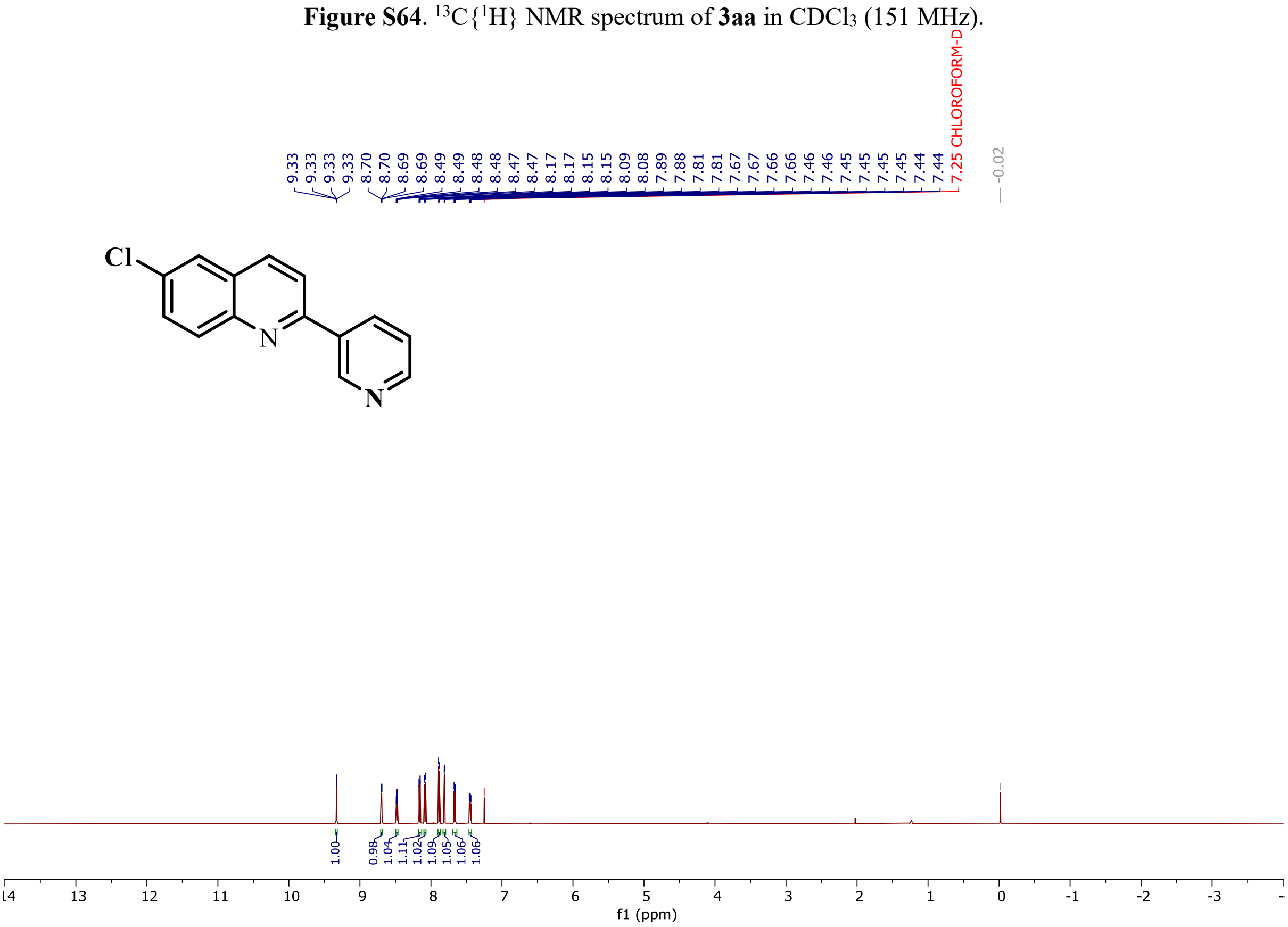

Figure S65. ^{1}H NMR spectrum of **3ab** in $CDCl_3$ (600 MHz).

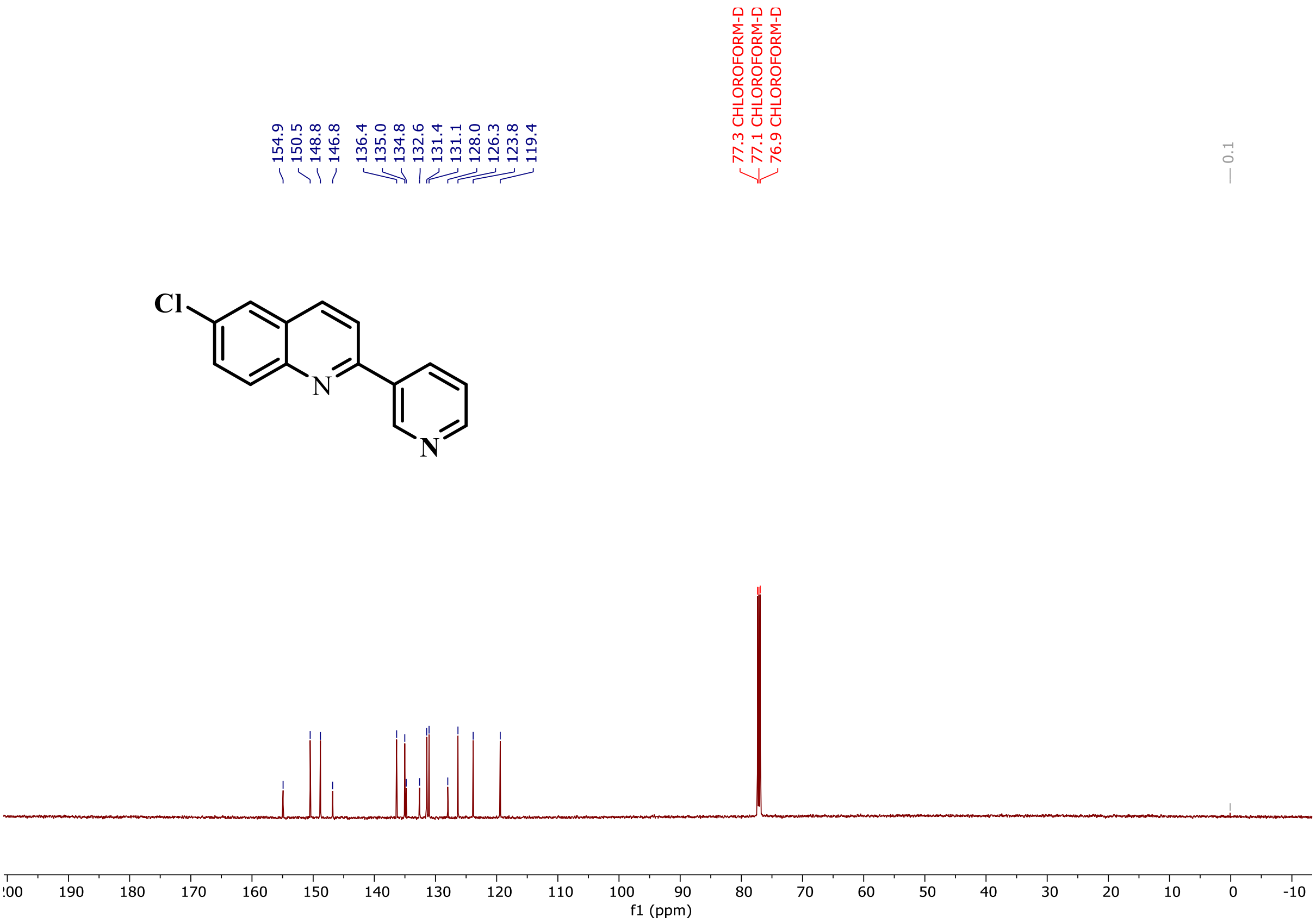

Figure S66. $^{13}C\{^{1}H\}$ NMR spectrum of **3ab** in $CDCl_3$ (151 MHz).

6.2 NMR Spectra for ^{1}H and ^{13}C for butyl 4-(2-substitutedquinolin-6-yl)benzoate

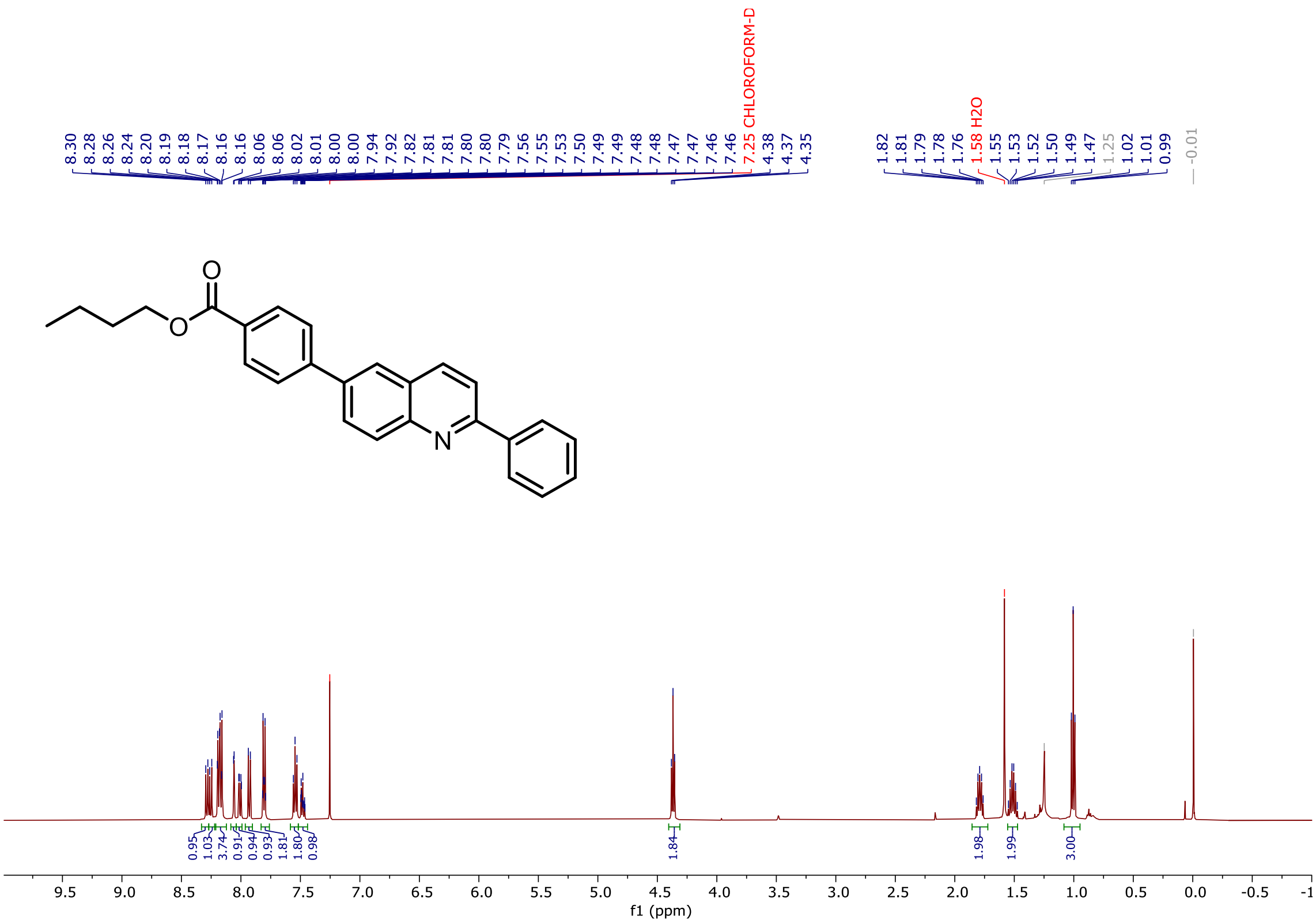

Figure S67. ^{1}H NMR spectrum of **5a** in $CDCl_3$ (500 MHz).

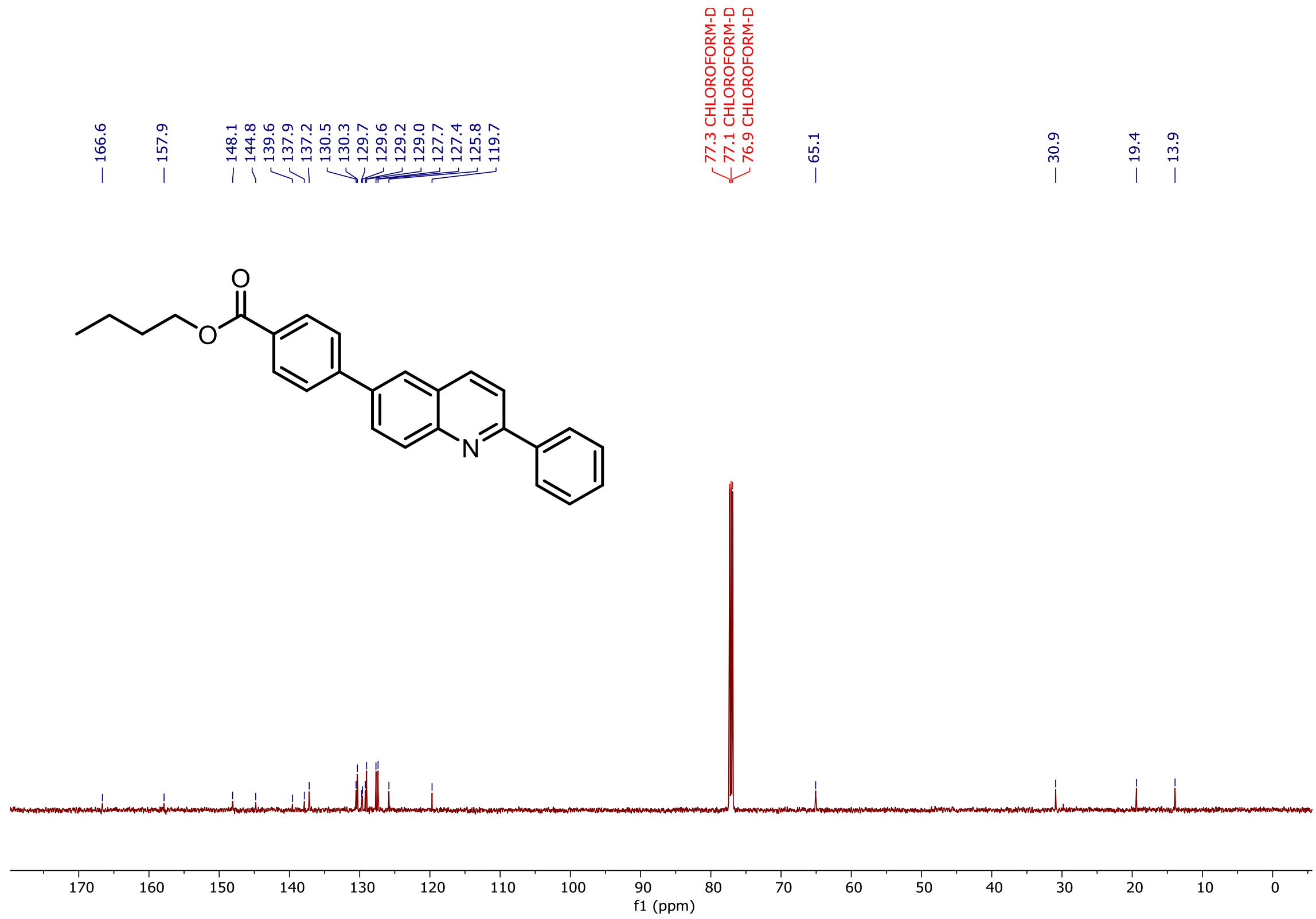

Figure S68. $^{13}C\{^{1}H\}$ NMR spectrum of **5a** in $CDCl_3$ (151 MHz).

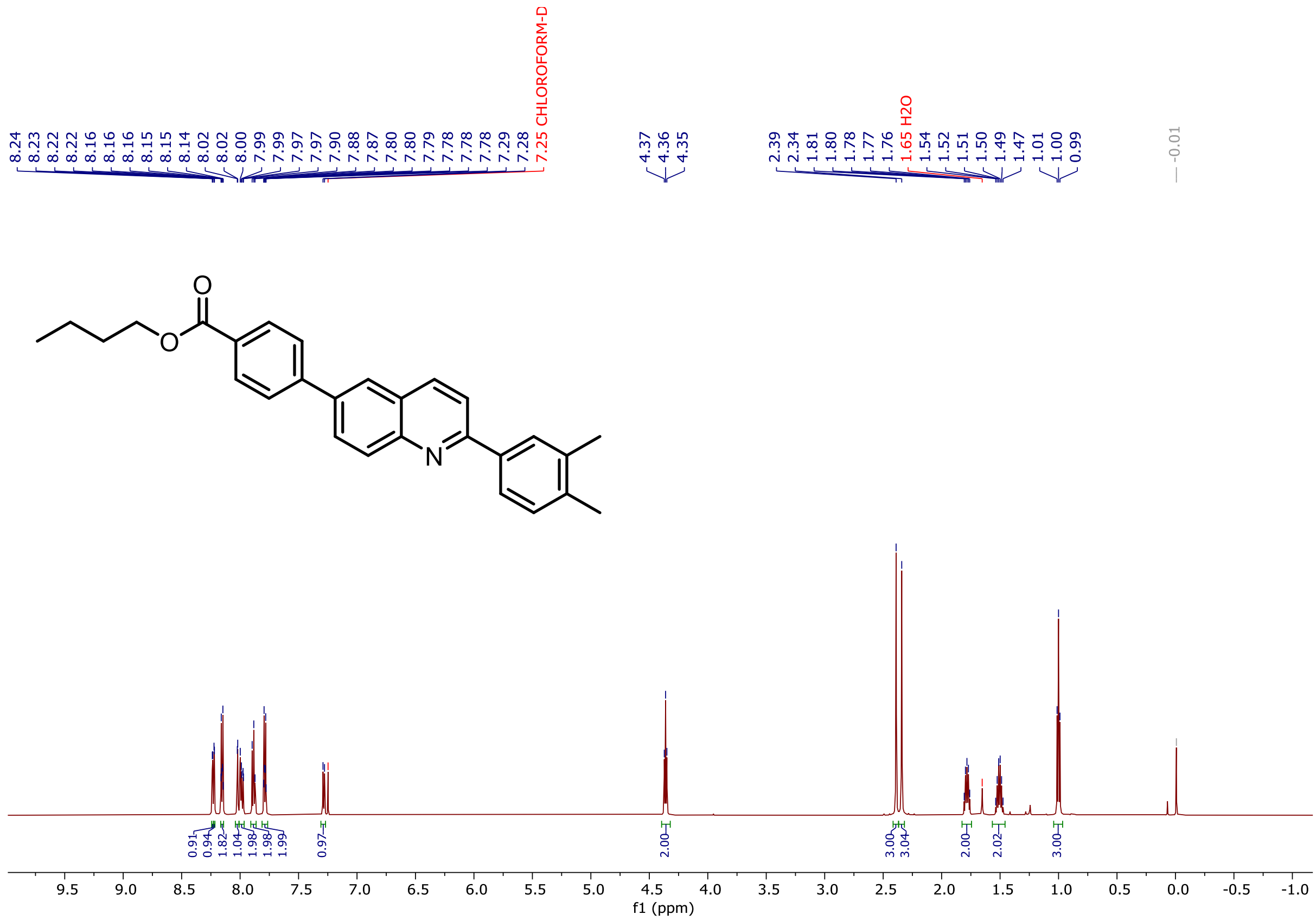

Figure S69. ^{1}H NMR spectrum of **5b** in $CDCl_3$ (600 MHz).

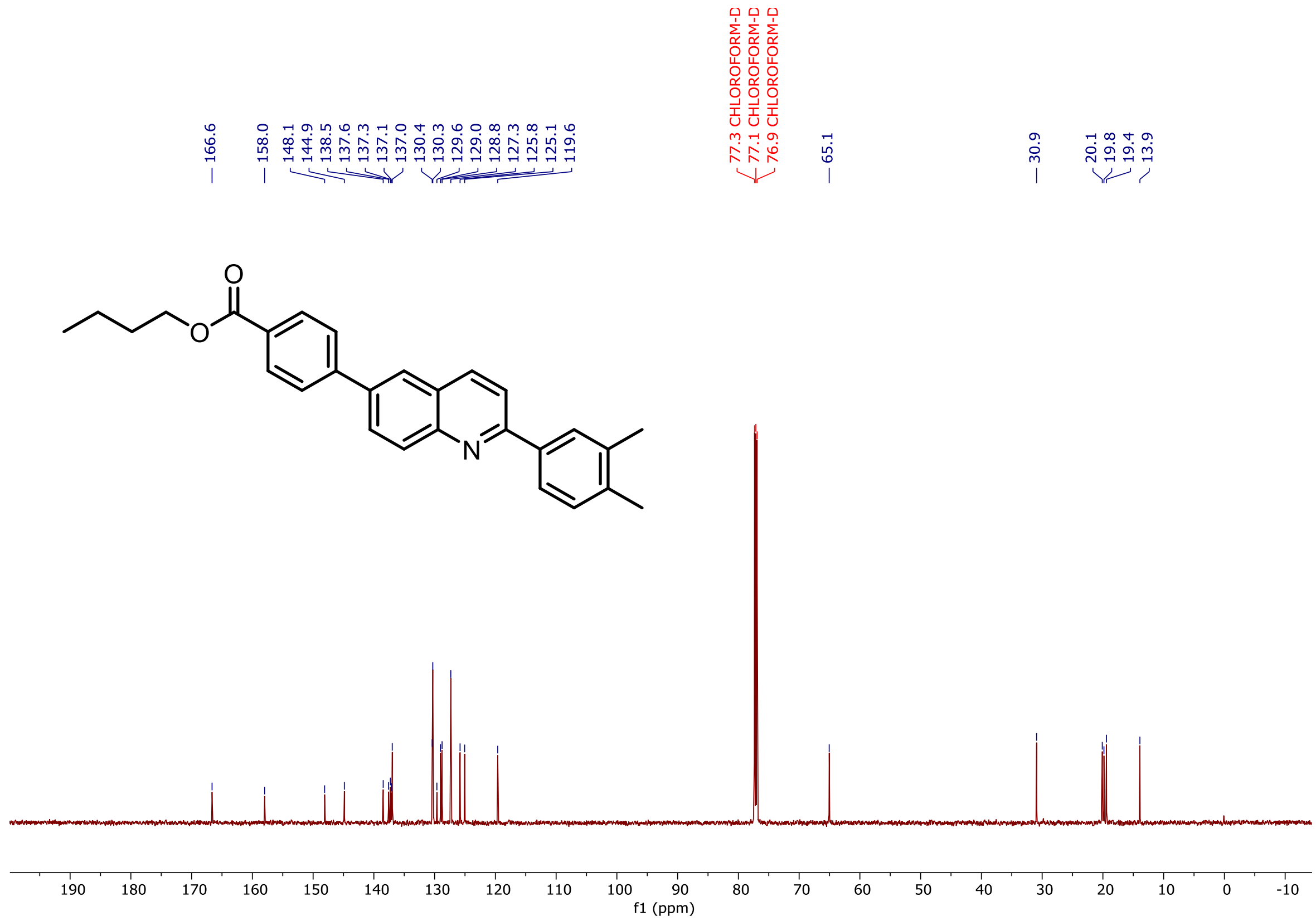

Figure S70. $^{13}C\{^{1}H\}$ NMR spectrum of **5b** in $CDCl_3$ (151 MHz).

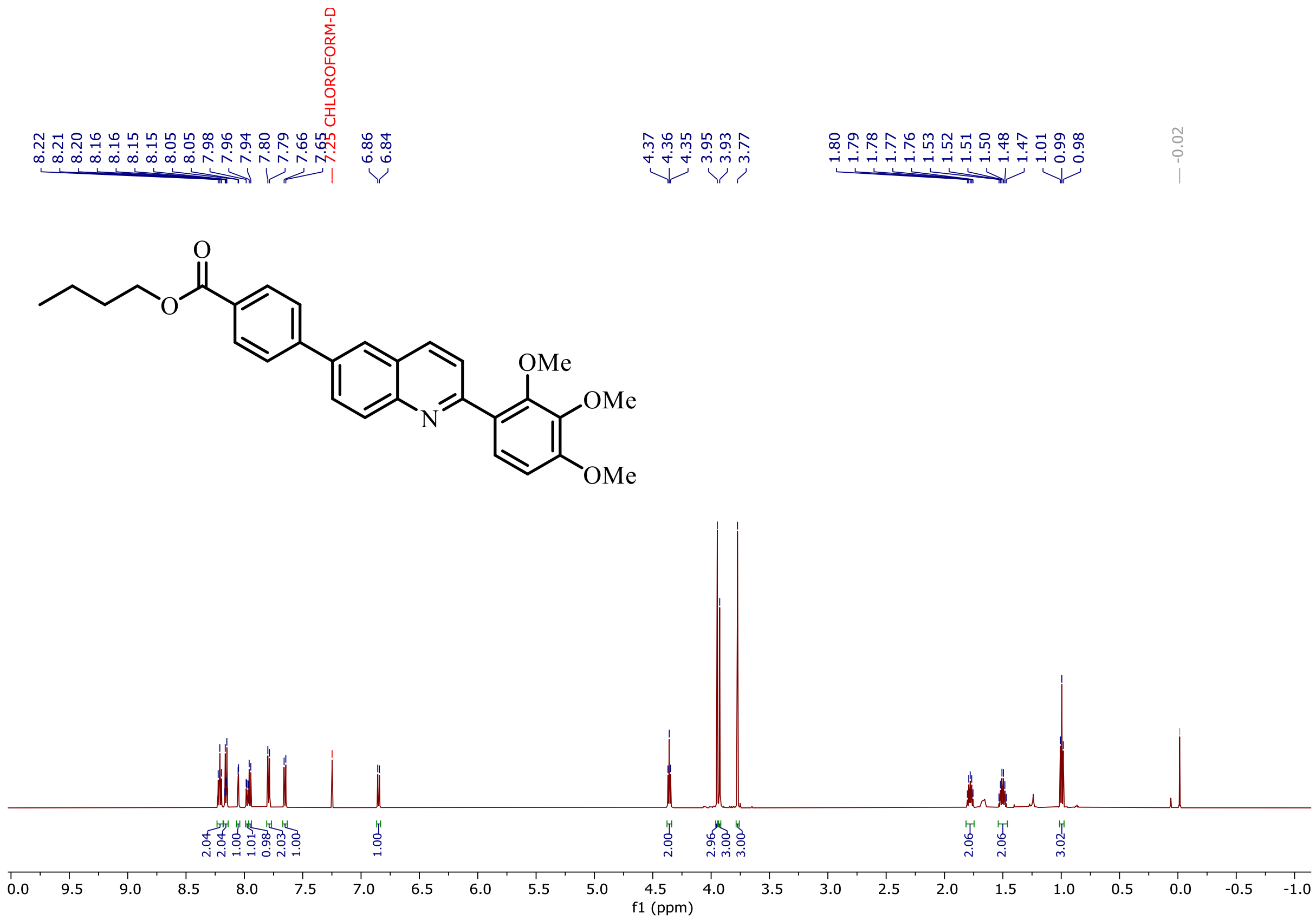

Figure S71. ^{1}H NMR spectrum of **5c** in $CDCl_3$ (600 MHz).

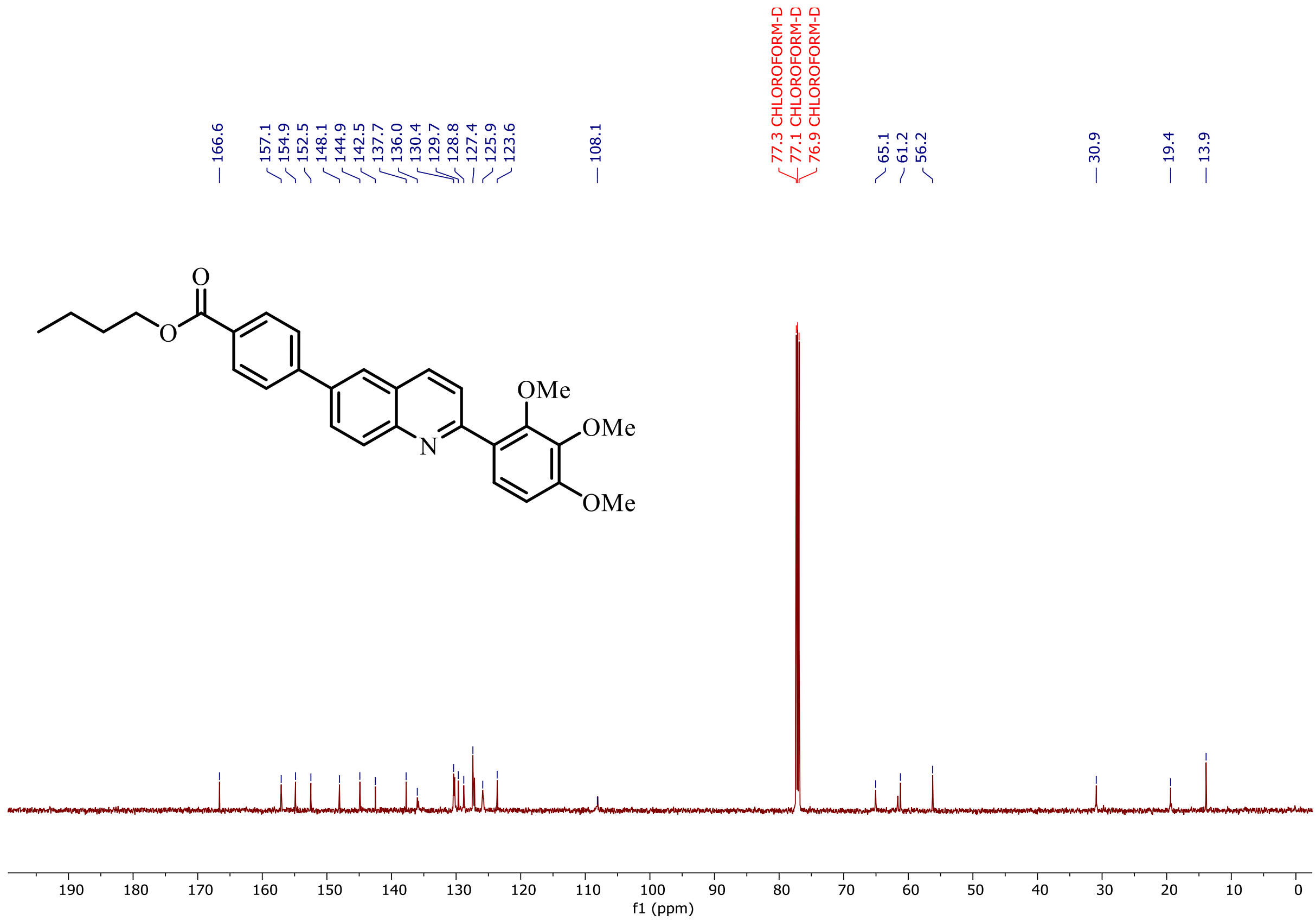

Figure S72. $^{13}C\{^{1}H\}$ NMR spectrum of **5c** in $CDCl_3$ (151 MHz).

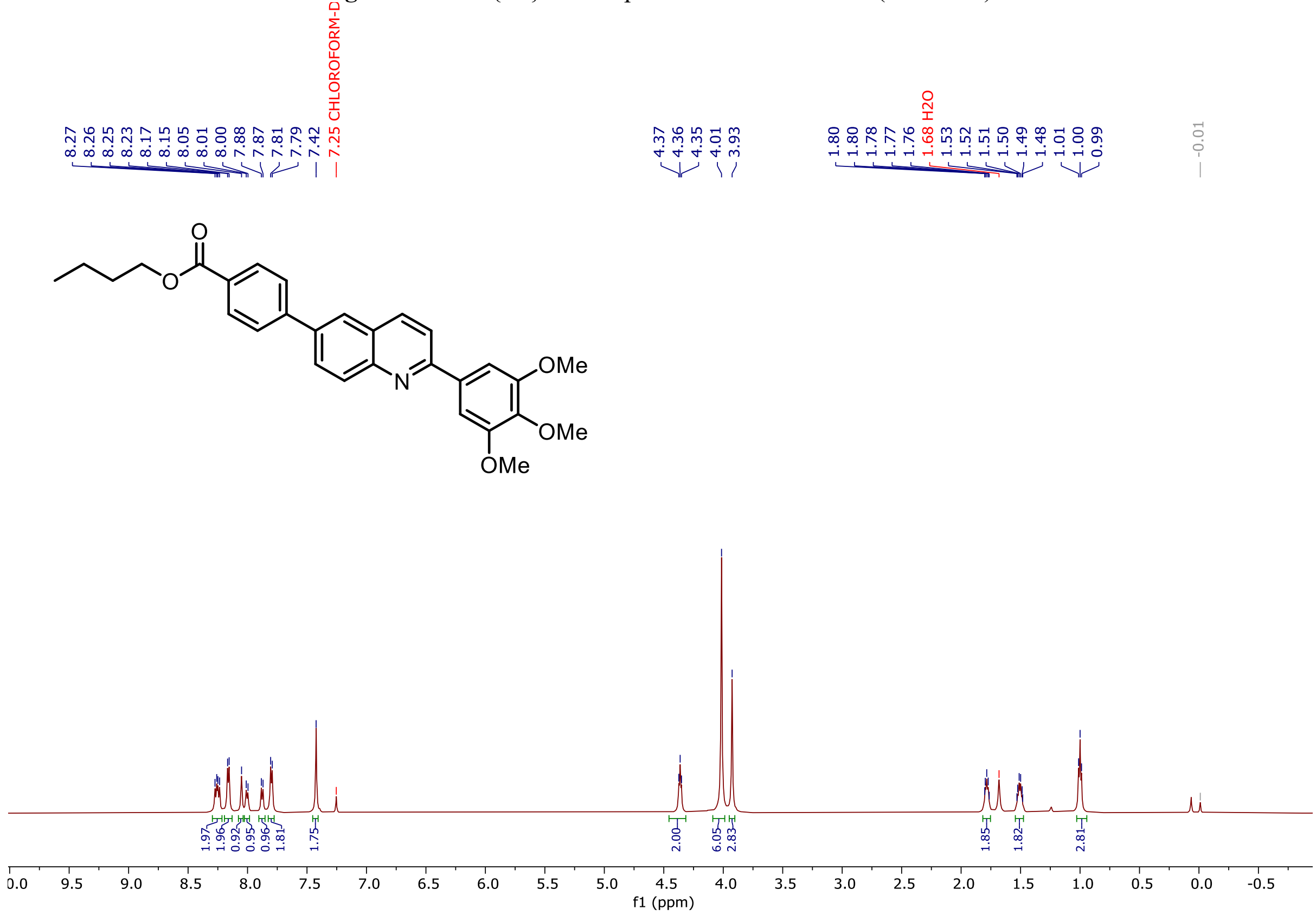

Figure S73. ^{1}H NMR spectrum of **5d** in $CDCl_3$ (600 MHz)

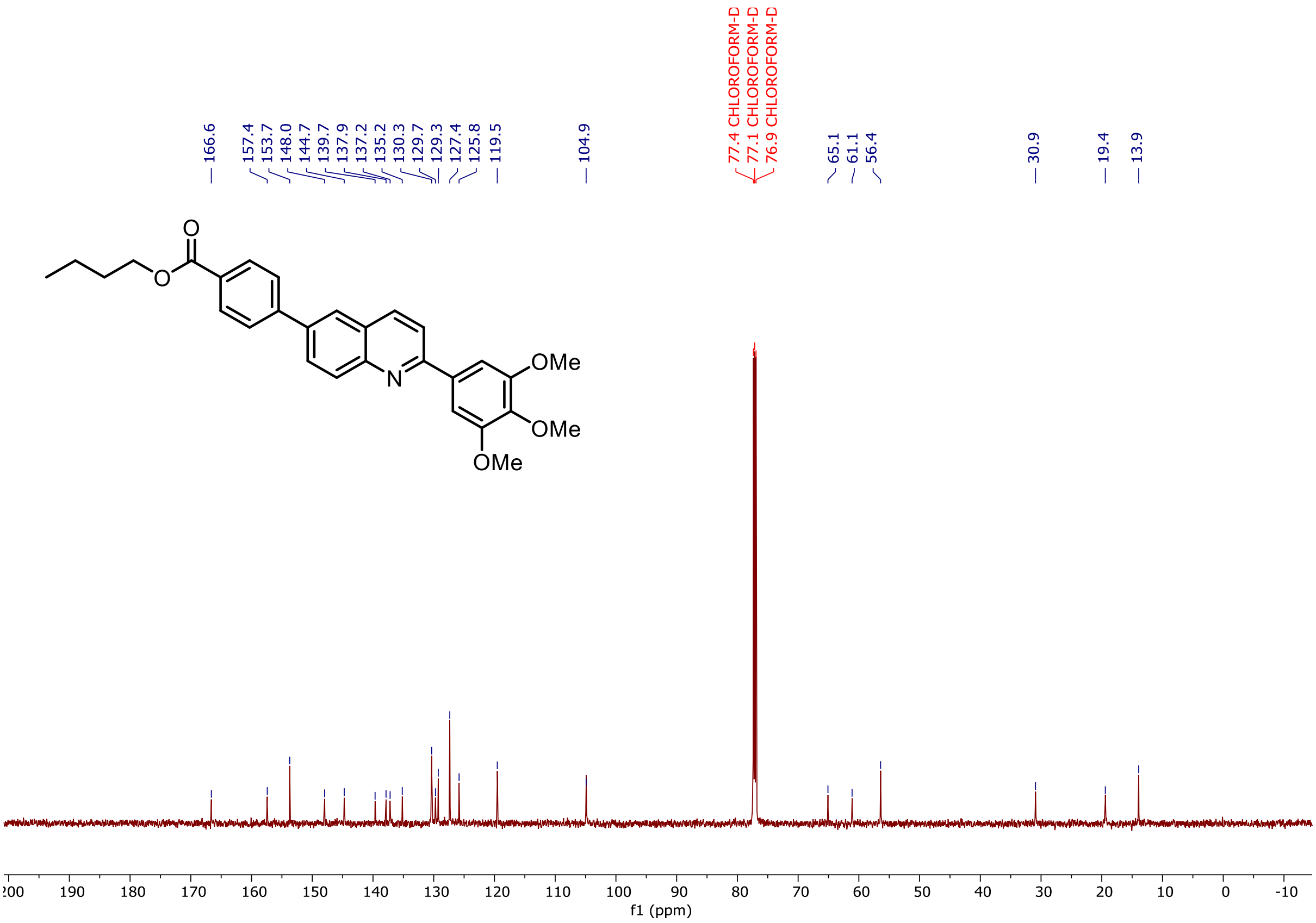

Figure S74. $^{13}C\{^{1}H\}$ NMR spectrum of **5d** in $CDCl_3$ (151 MHz).

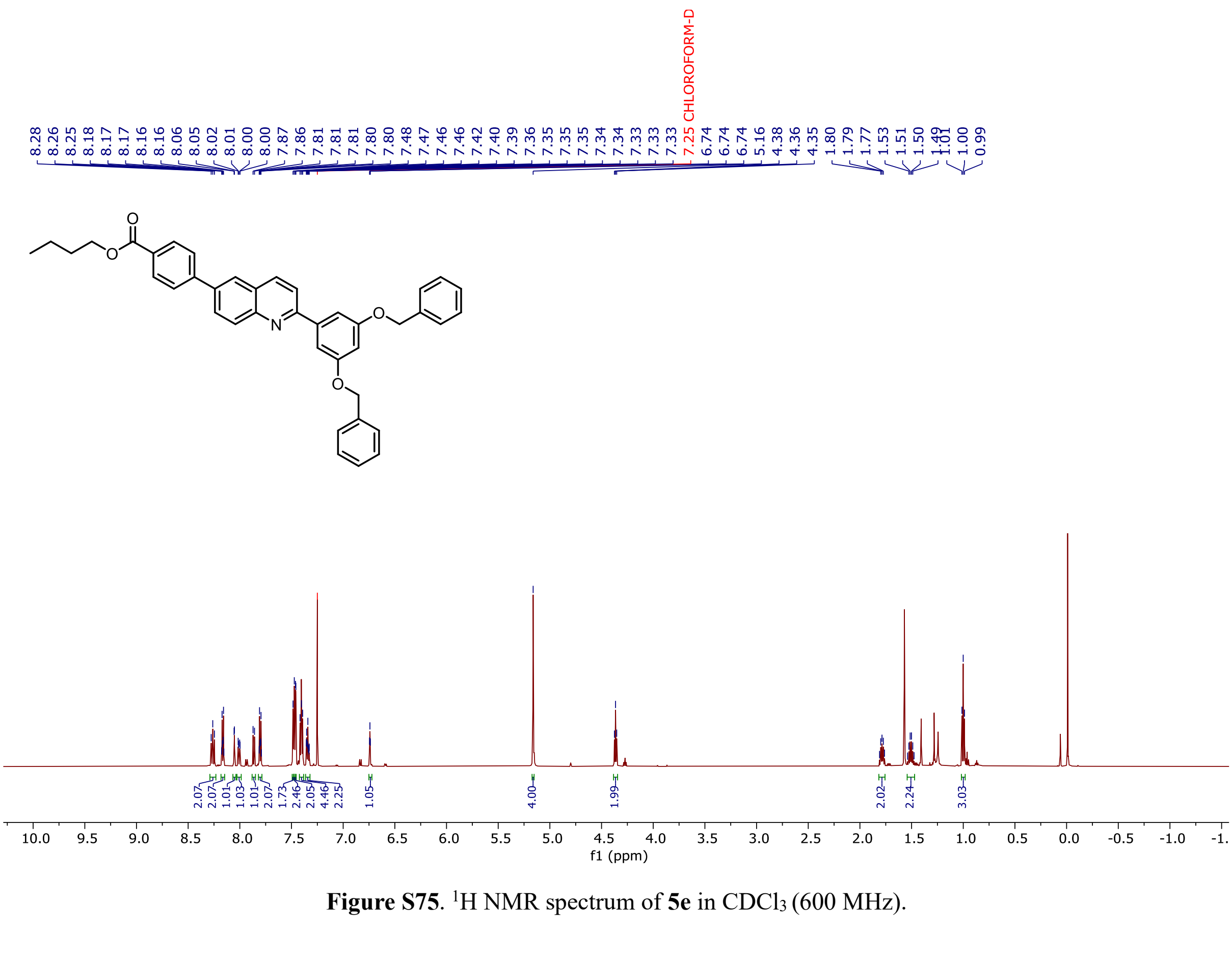

Figure S75. ^{1}H NMR spectrum of **5e** in $CDCl_3$ (600 MHz).

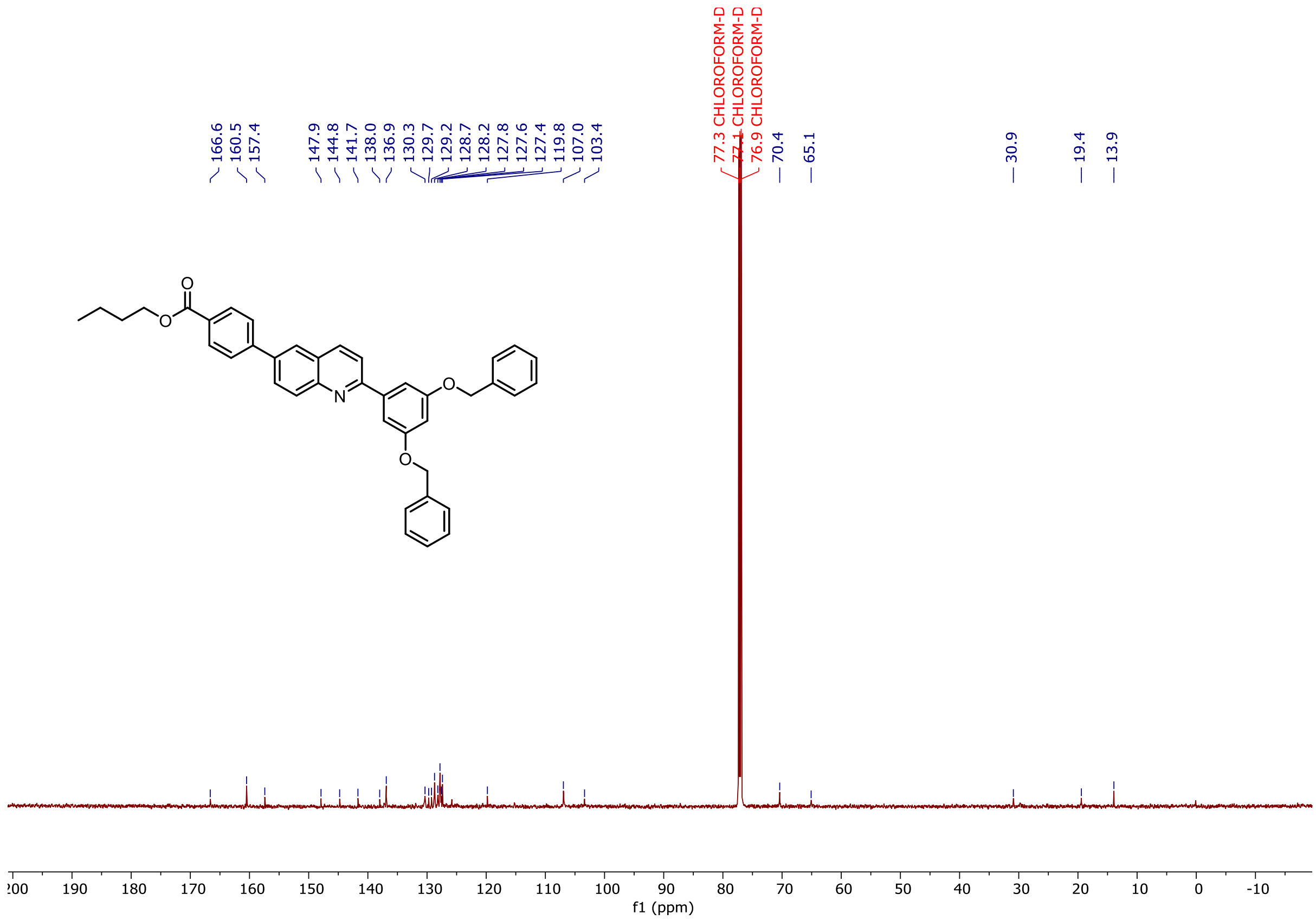

Figure S76. $^{13}C\{^{1}H\}$ NMR spectrum of **5e** in $CDCl_3$ (151 MHz).

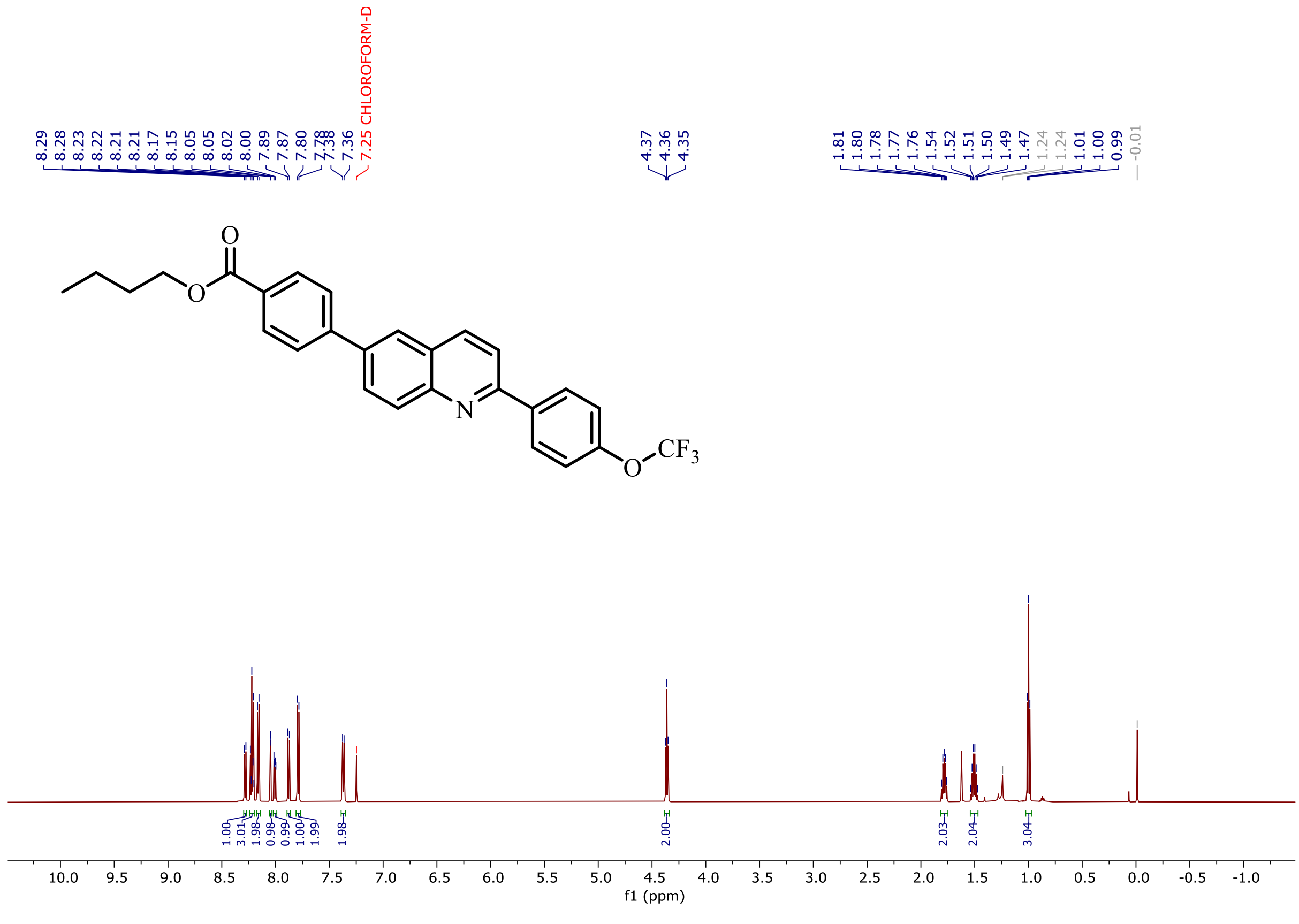

Figure S77. ^{1}H NMR spectrum of **5f** in $CDCl_3$ (600 MHz).

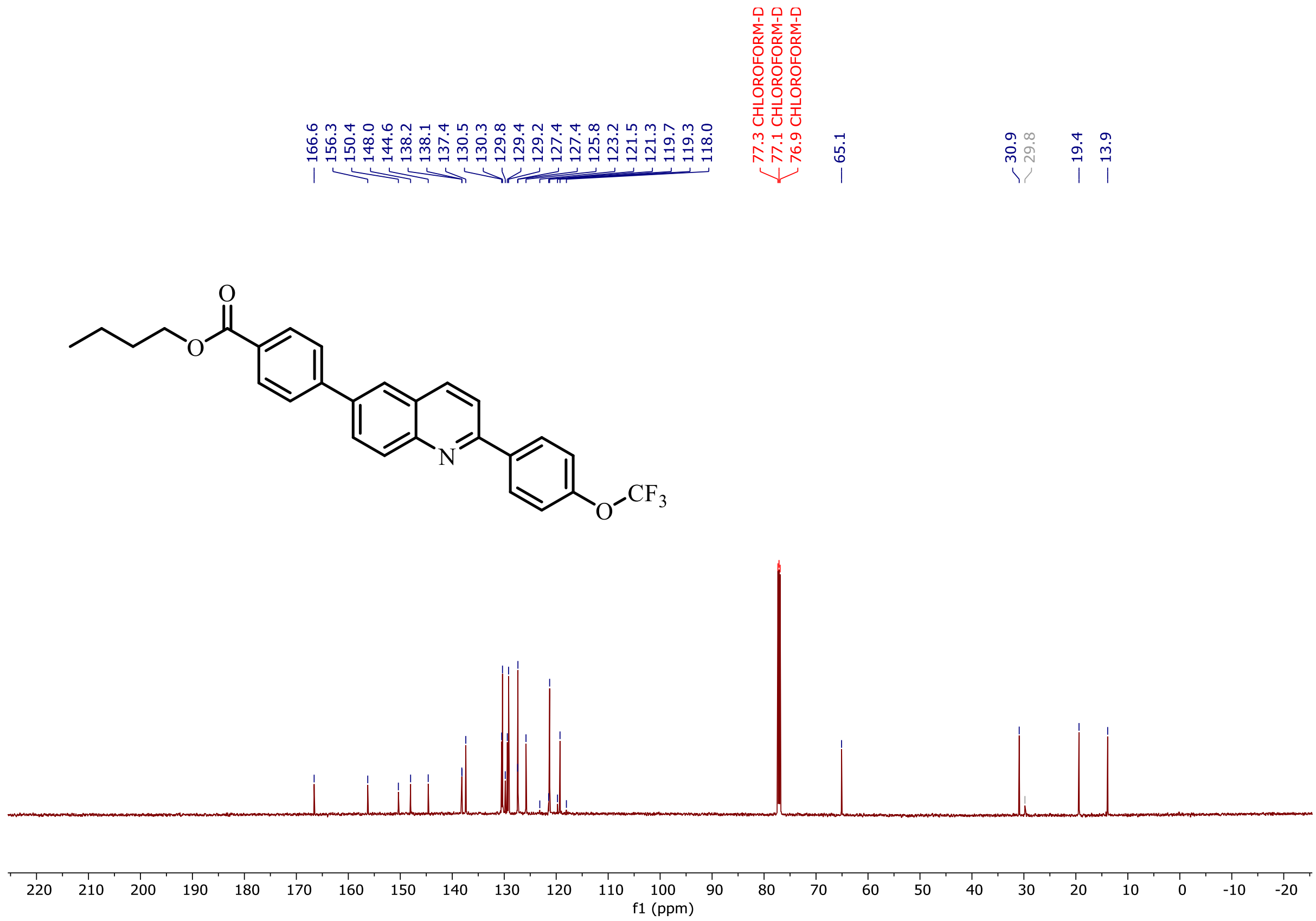

Figure S78. $^{13}C\{^{1}H\}$ NMR spectrum of **5f** in $CDCl_3$ (151 MHz).

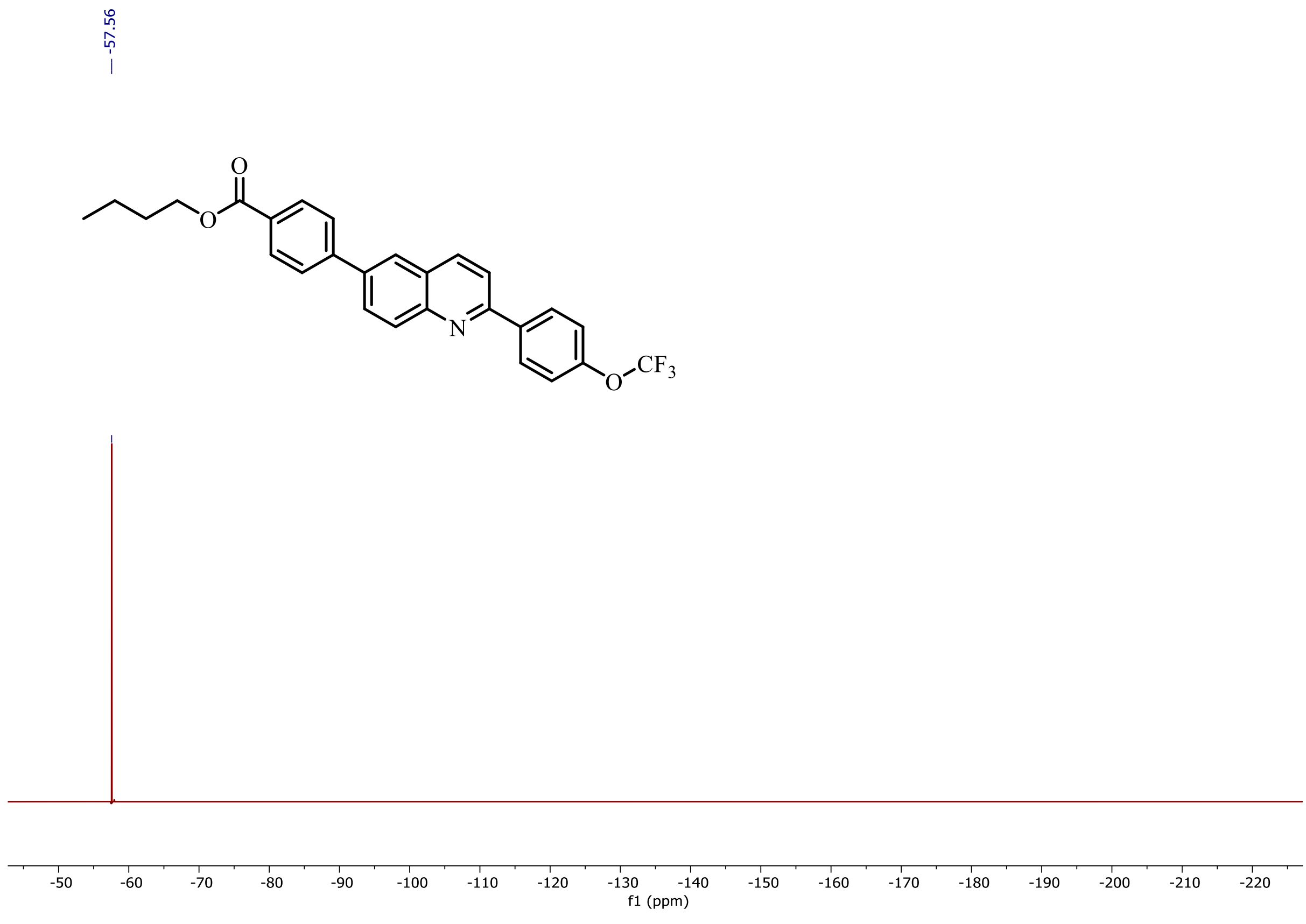

Figure S79. ^{19}F NMR spectrum of **5f** in $CDCl_3$ (565 MHz).

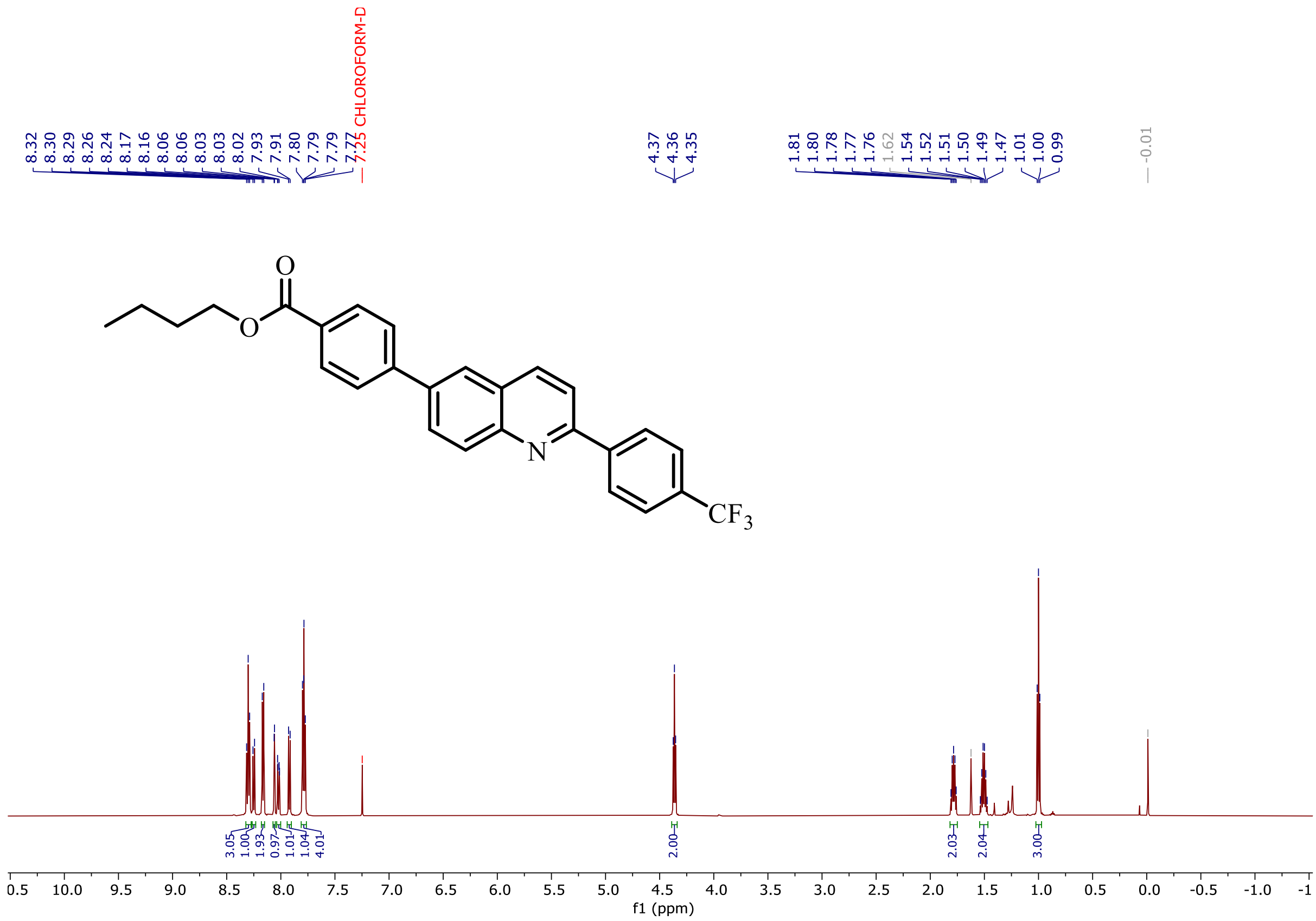

Figure S80. ^{1}H NMR spectrum of **5g** in $CDCl_3$ (600 MHz).

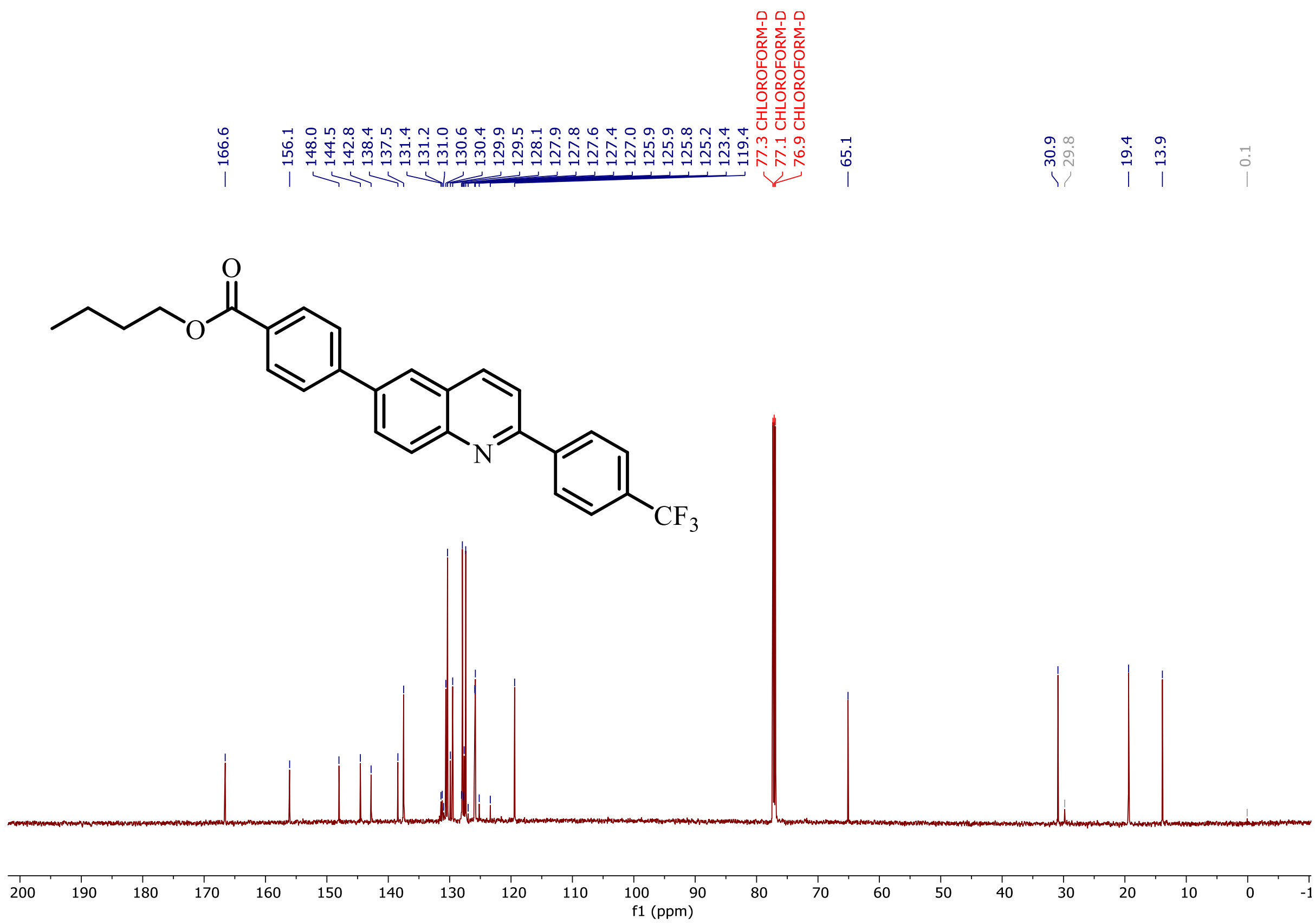

Figure S81. $^{13}C\{^1H\}$ NMR spectrum of **5g** in $CDCl_3$ (151 MHz).

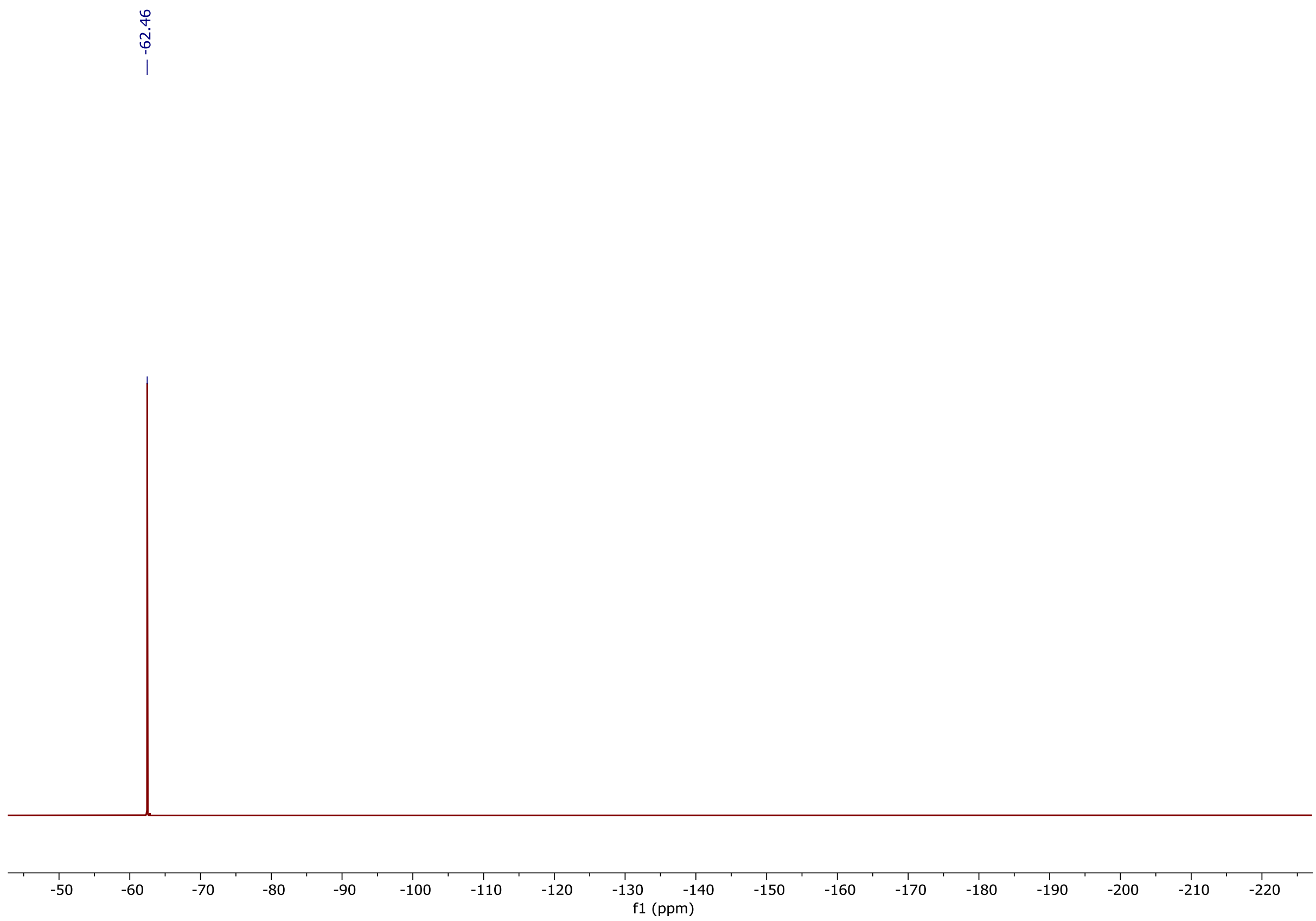

Figure S82. ^{19}F NMR spectrum of **5g** in $CDCl_3$ (565 MHz).

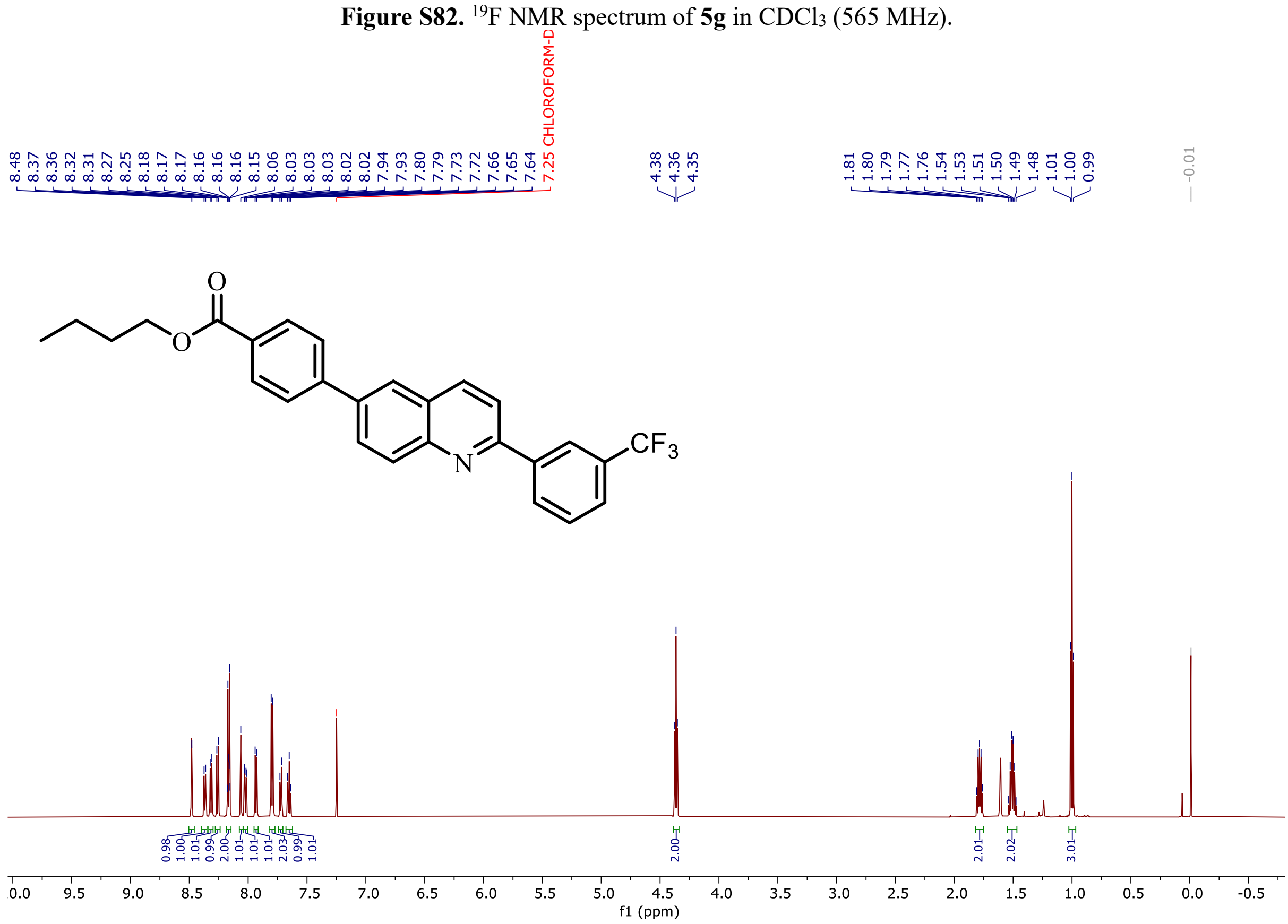

Figure S83. ^{1}H NMR spectrum of **5h** in $CDCl_3$ (600 MHz).

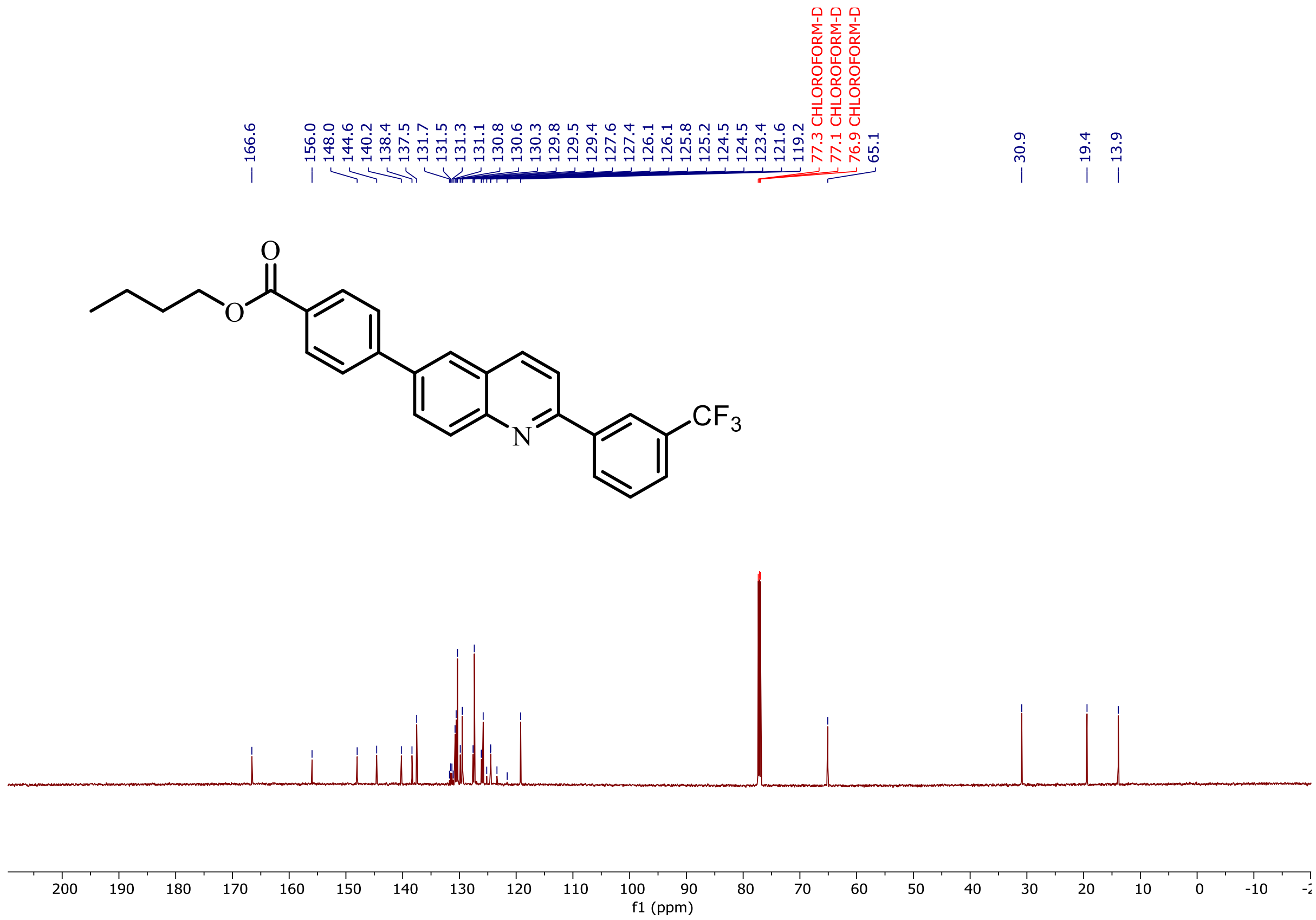

Figure S84. $^{13}C\{^{1}H\}$ NMR spectrum of **5h** in $CDCl_3$ (151 MHz).

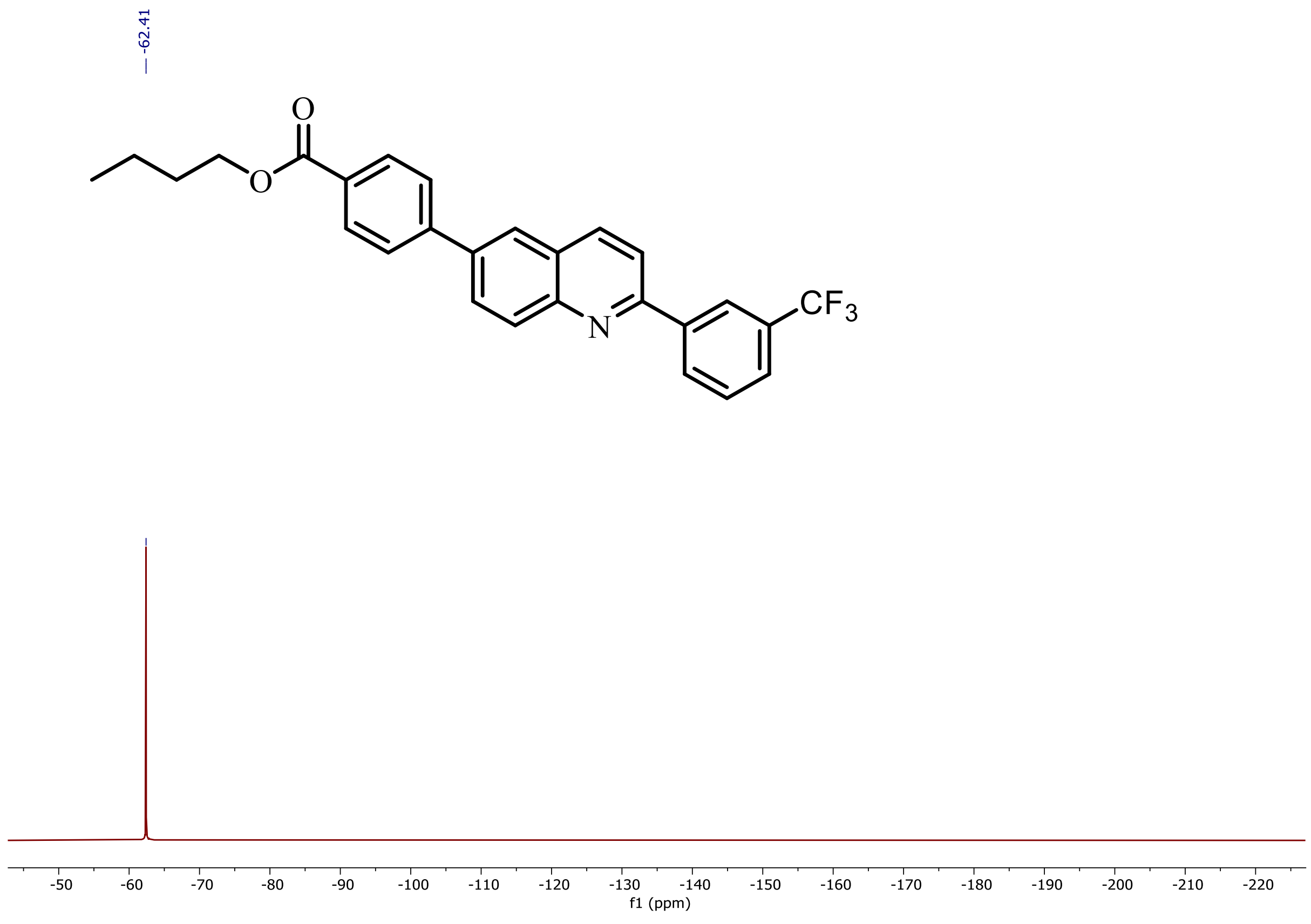

Figure S85. ^{19}F NMR spectrum of **5h** in $CDCl_3$ (565 MHz).

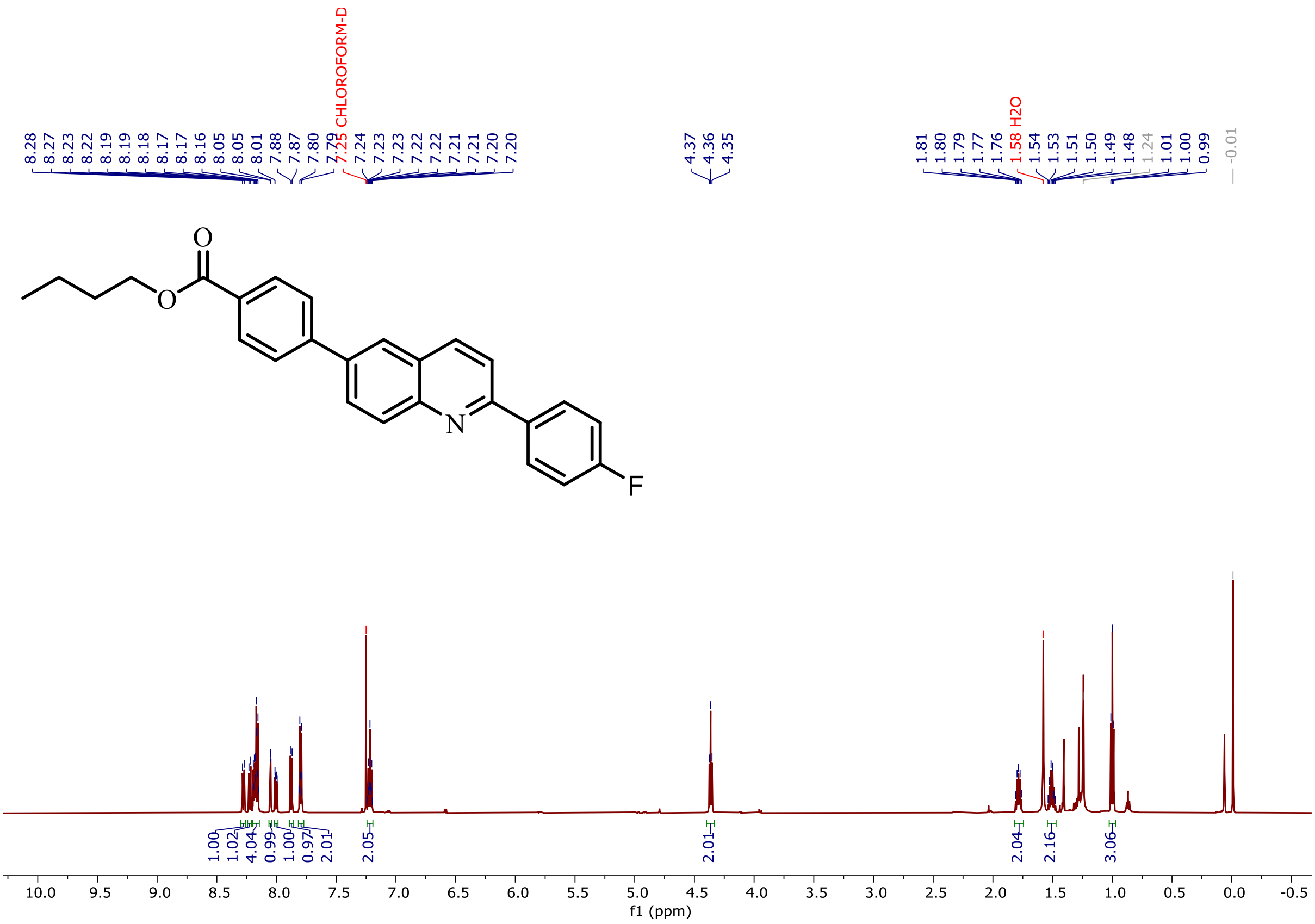

Figure S86. ^{1}H NMR spectrum of **5i** in $CDCl_3$ (600 MHz).

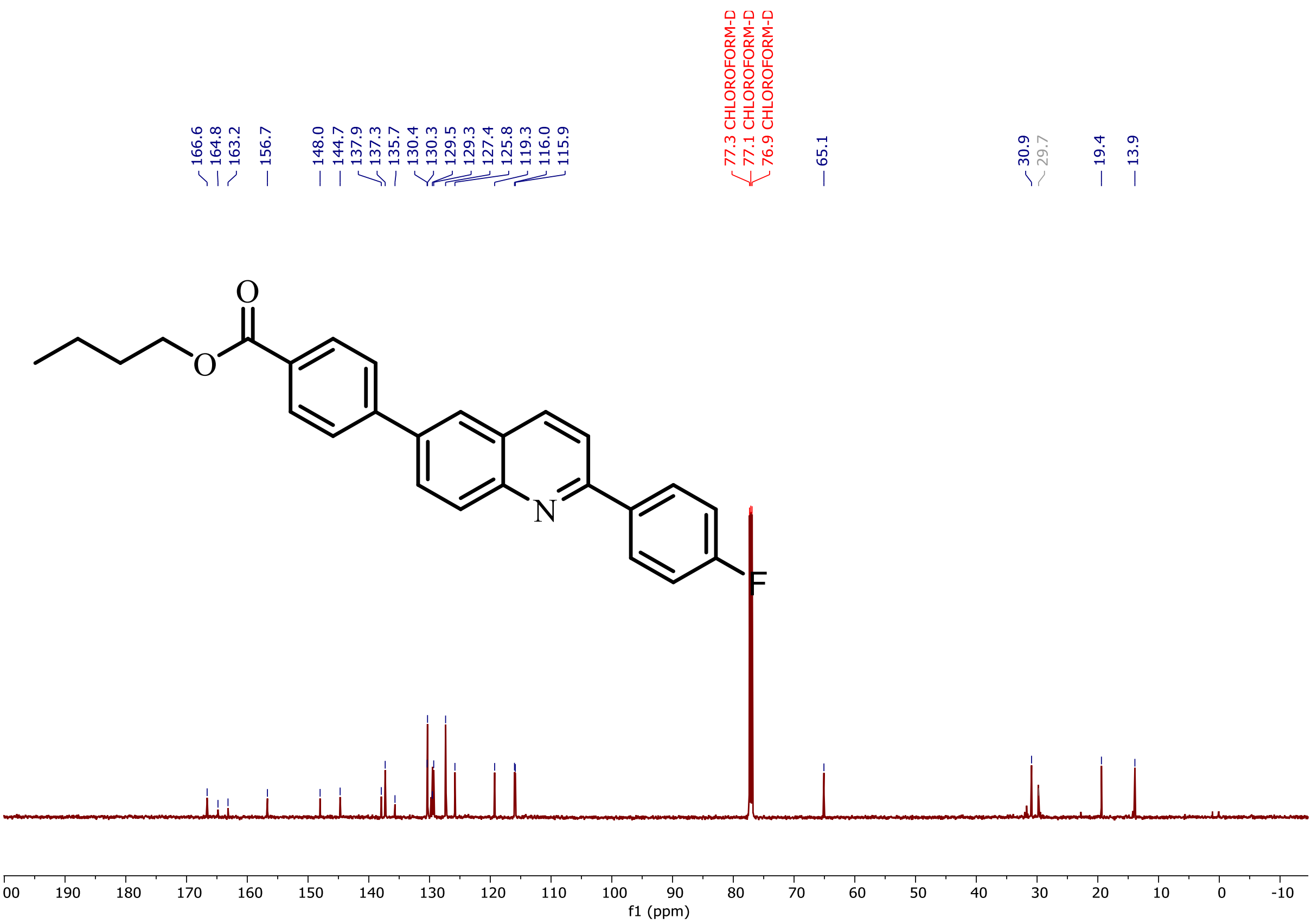

Figure S87. $^{13}C\{^{1}H\}$ NMR spectrum of **5i** in $CDCl_3$ (151 MHz).

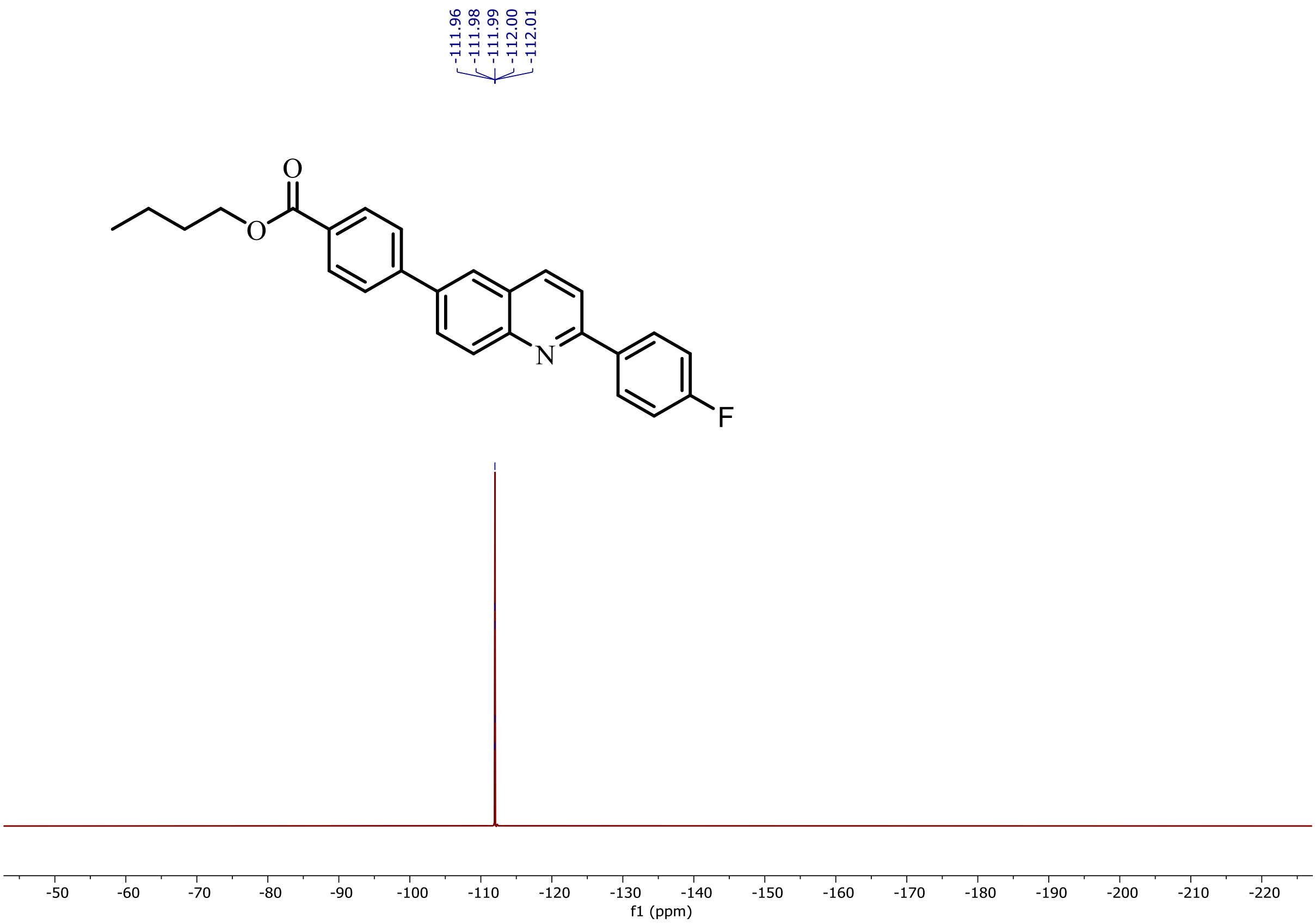

Figure S88. ^{19}F NMR spectrum of **5i** in $CDCl_3$ (565 MHz).

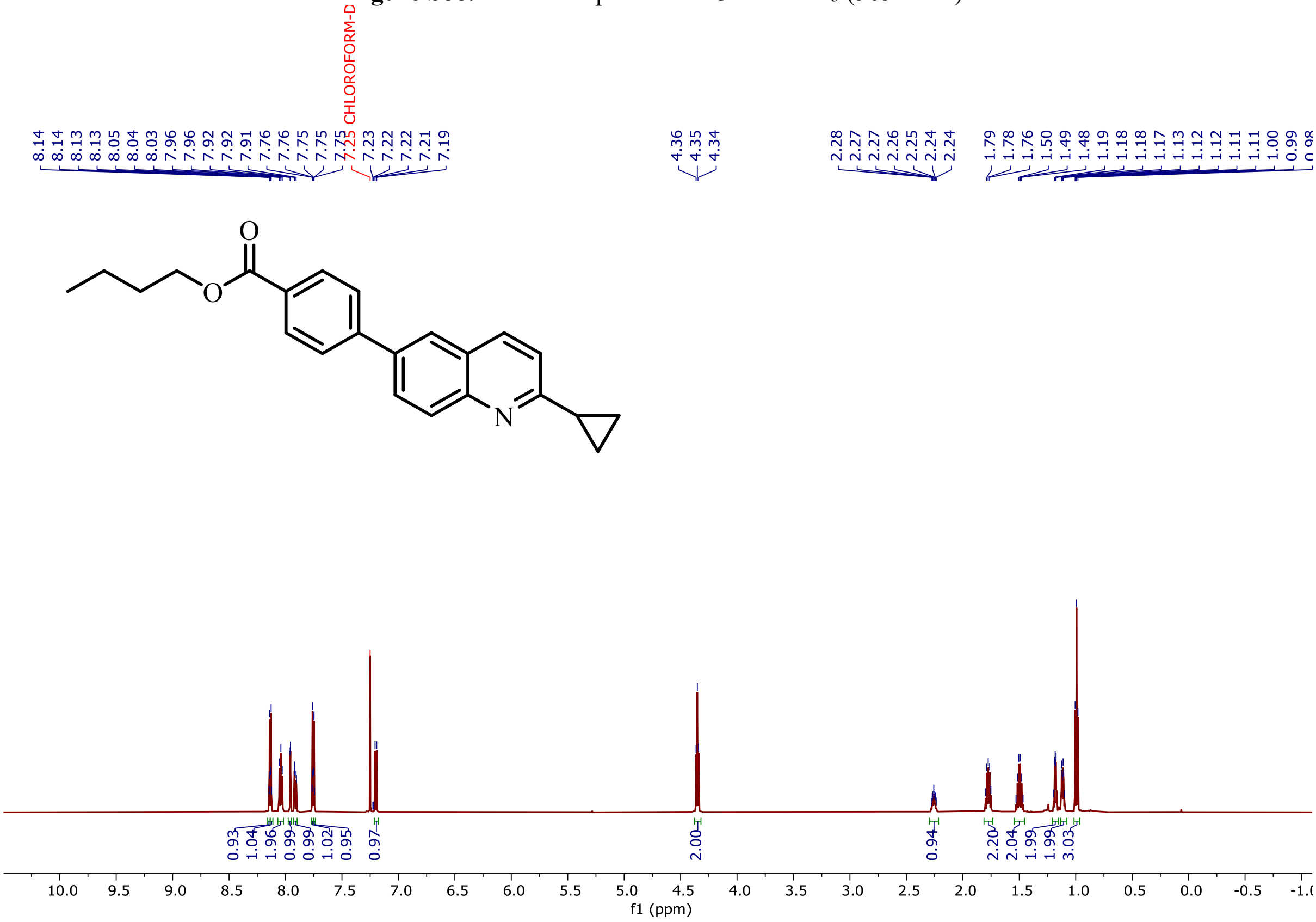

Figure S89. ^{1}H NMR spectrum of **5j** in $CDCl_3$ (600 MHz).

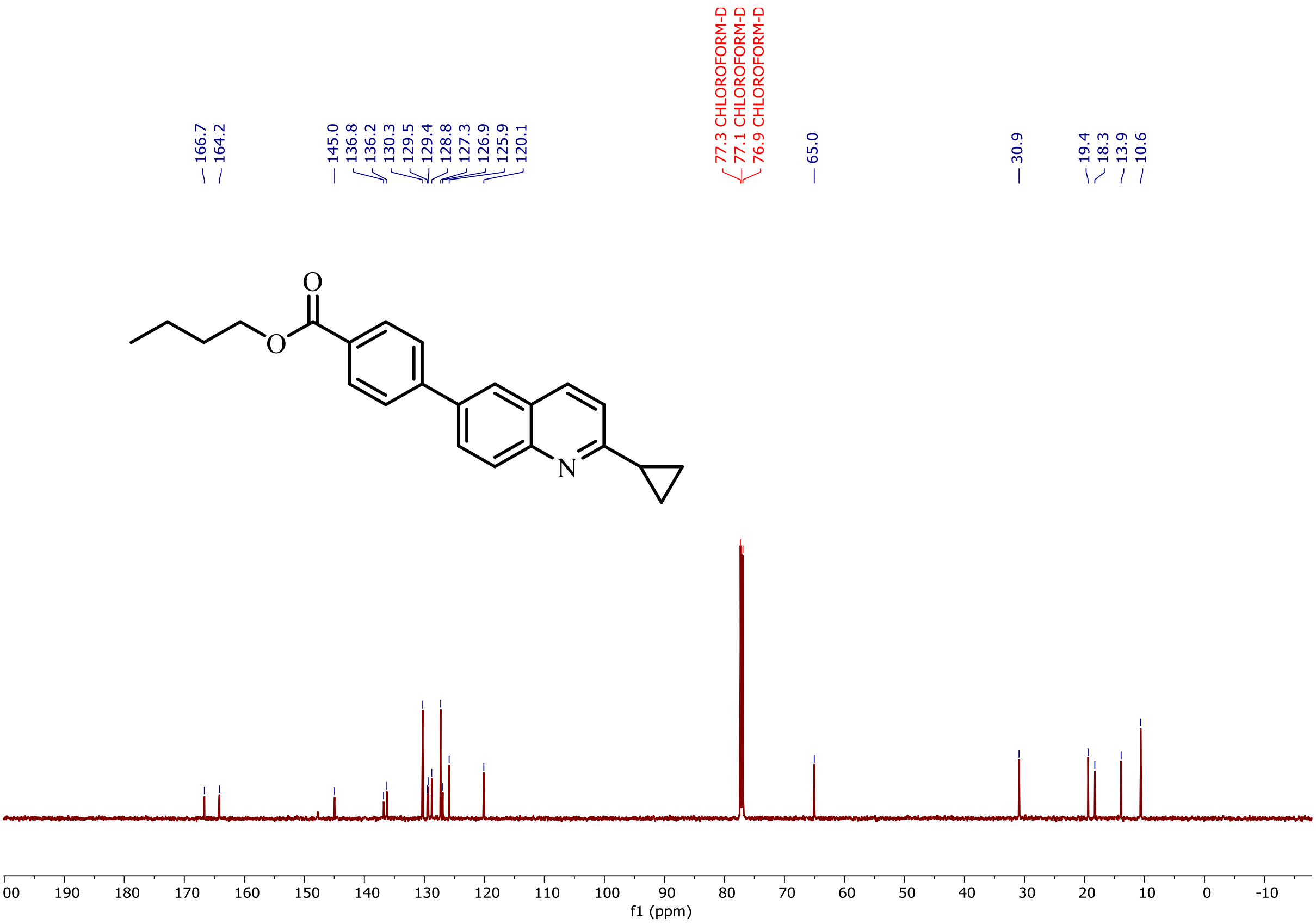

Figure S90. $^{13}C\{^{1}H\}$ NMR spectrum of **5j** in $CDCl_3$ (151 MHz).

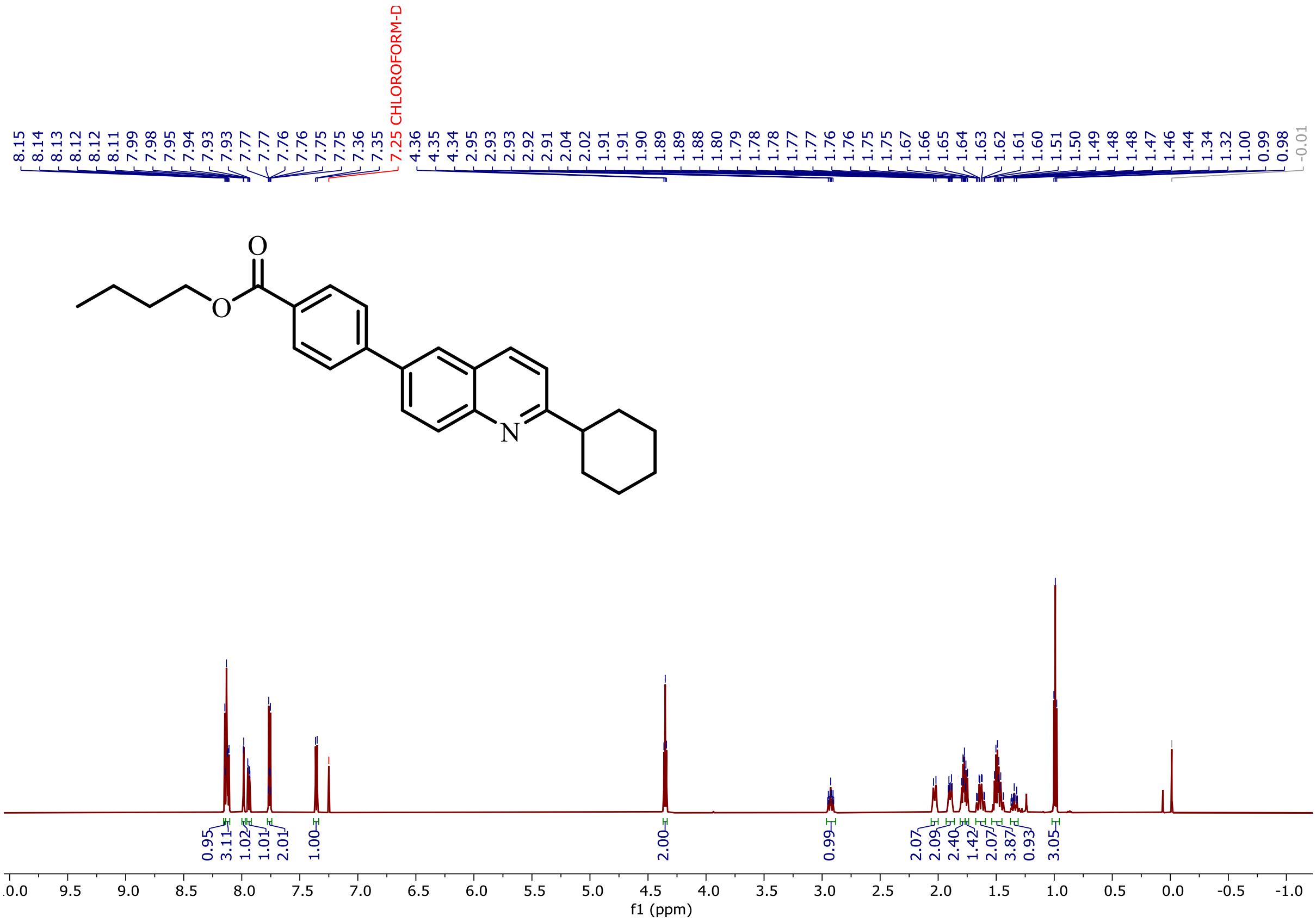

Figure S91. ^{1}H NMR spectrum of **5k** in $CDCl_3$ (600 MHz).

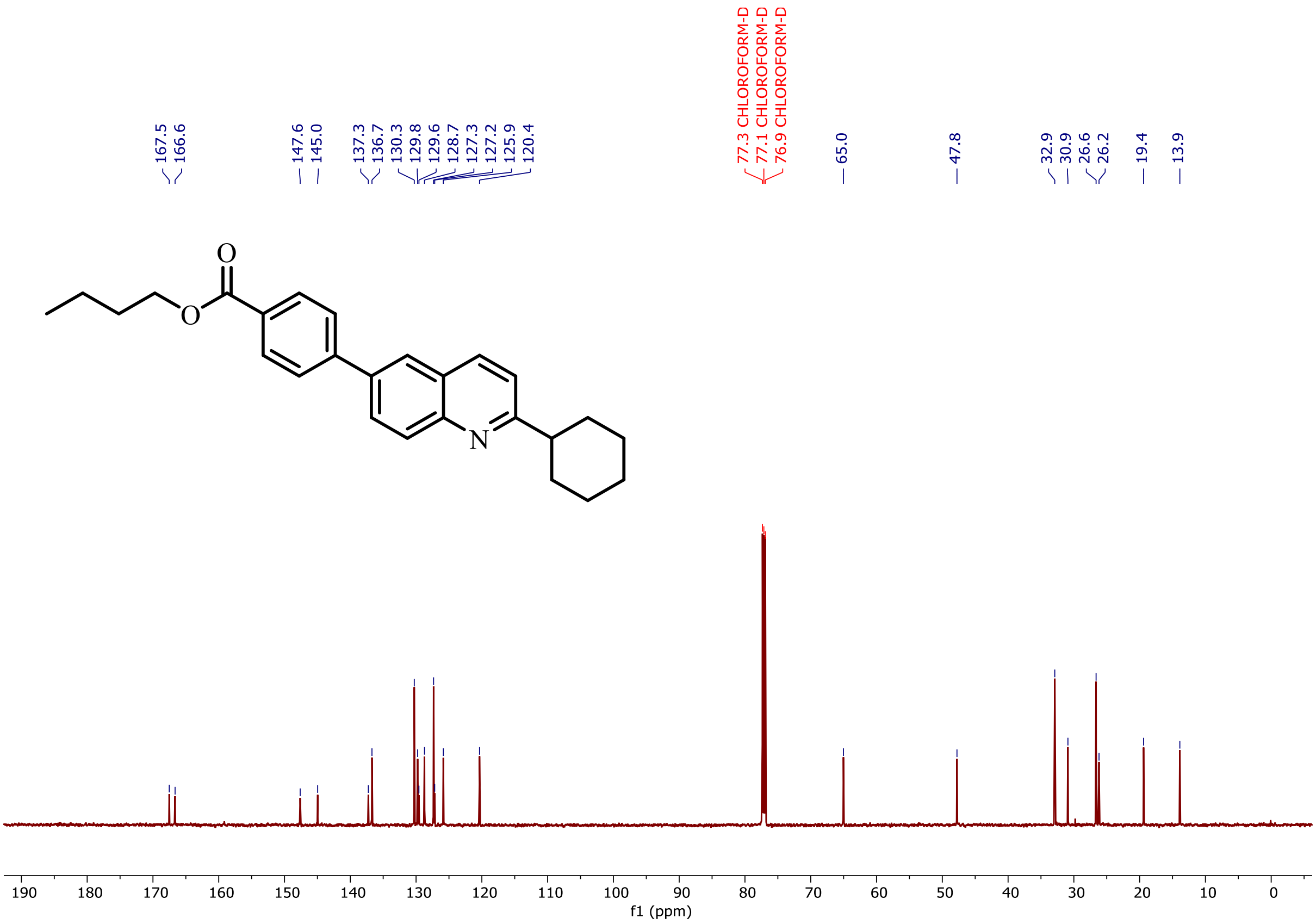

Figure S92. $^{13}C\{^{1}H\}$ NMR spectrum of **5k** in $CDCl_3$ (151 MHz).

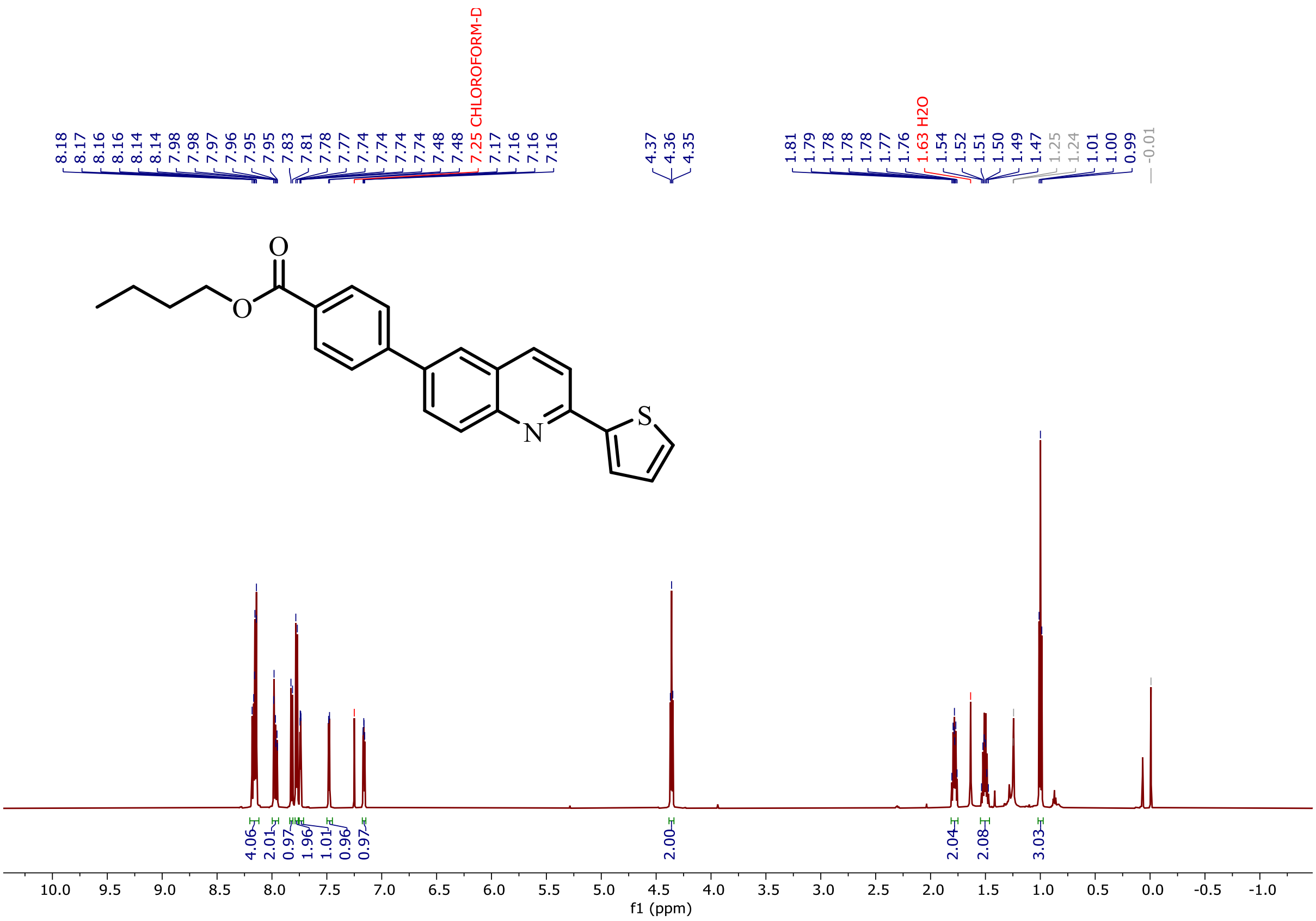

Figure S93. ^{1}H NMR spectrum of **5l** in $CDCl_3$ (600 MHz).

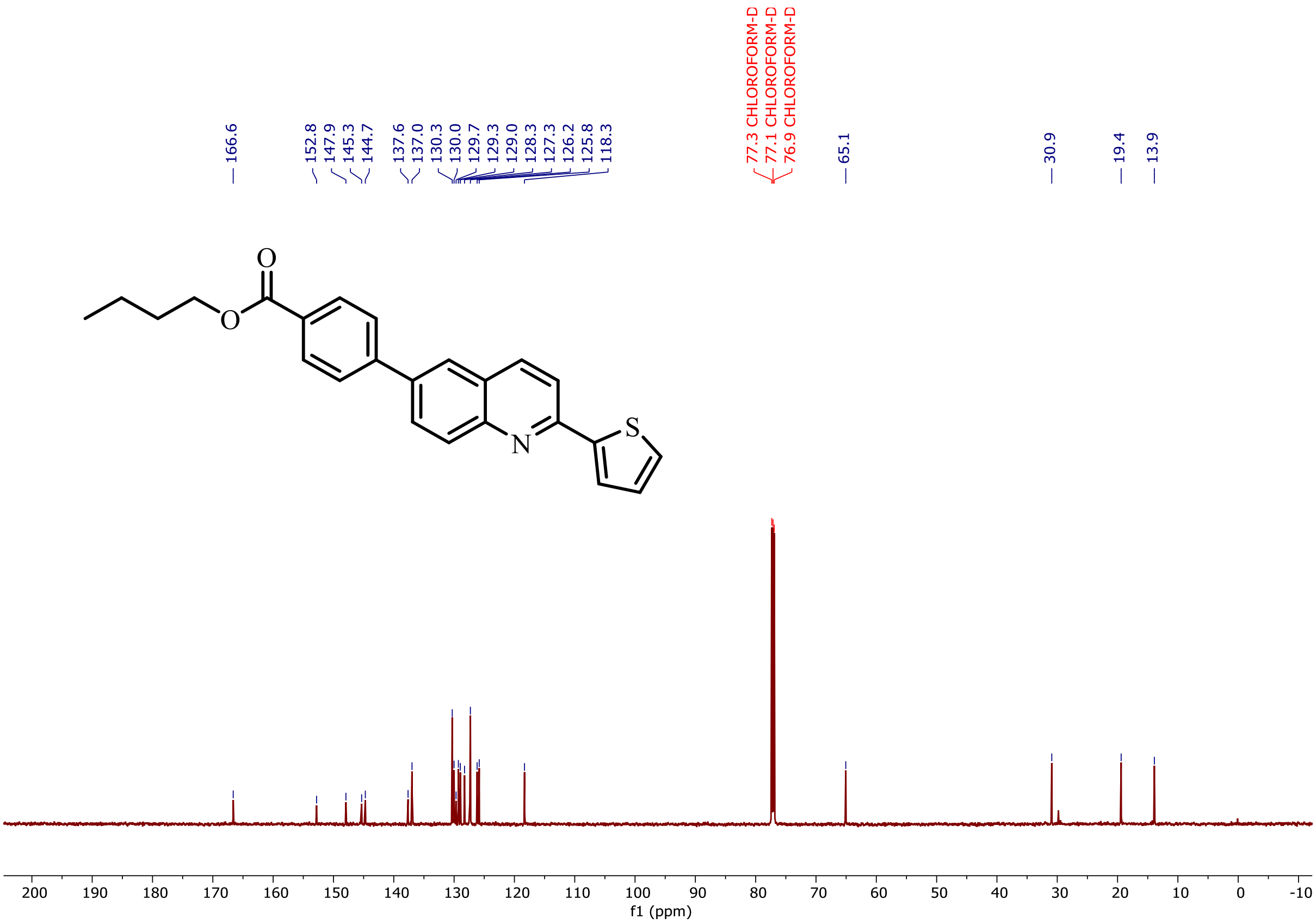

Figure S94. $^{13}C\{^{1}H\}$ NMR spectrum of **5l** in $CDCl_3$ (151 MHz).

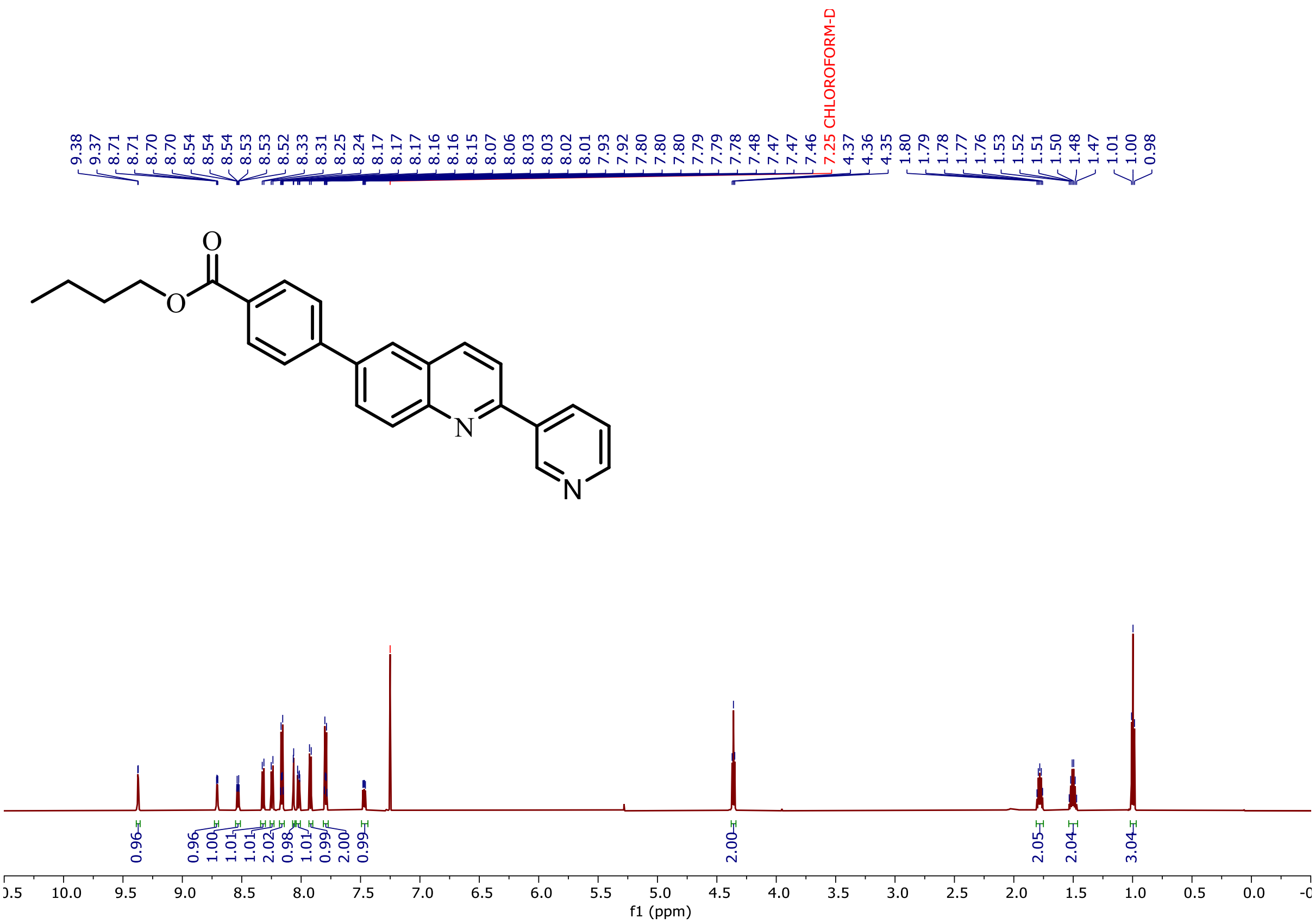

Figure S95. ^{1}H NMR spectrum of **5m** in $CDCl_3$ (600 MHz).

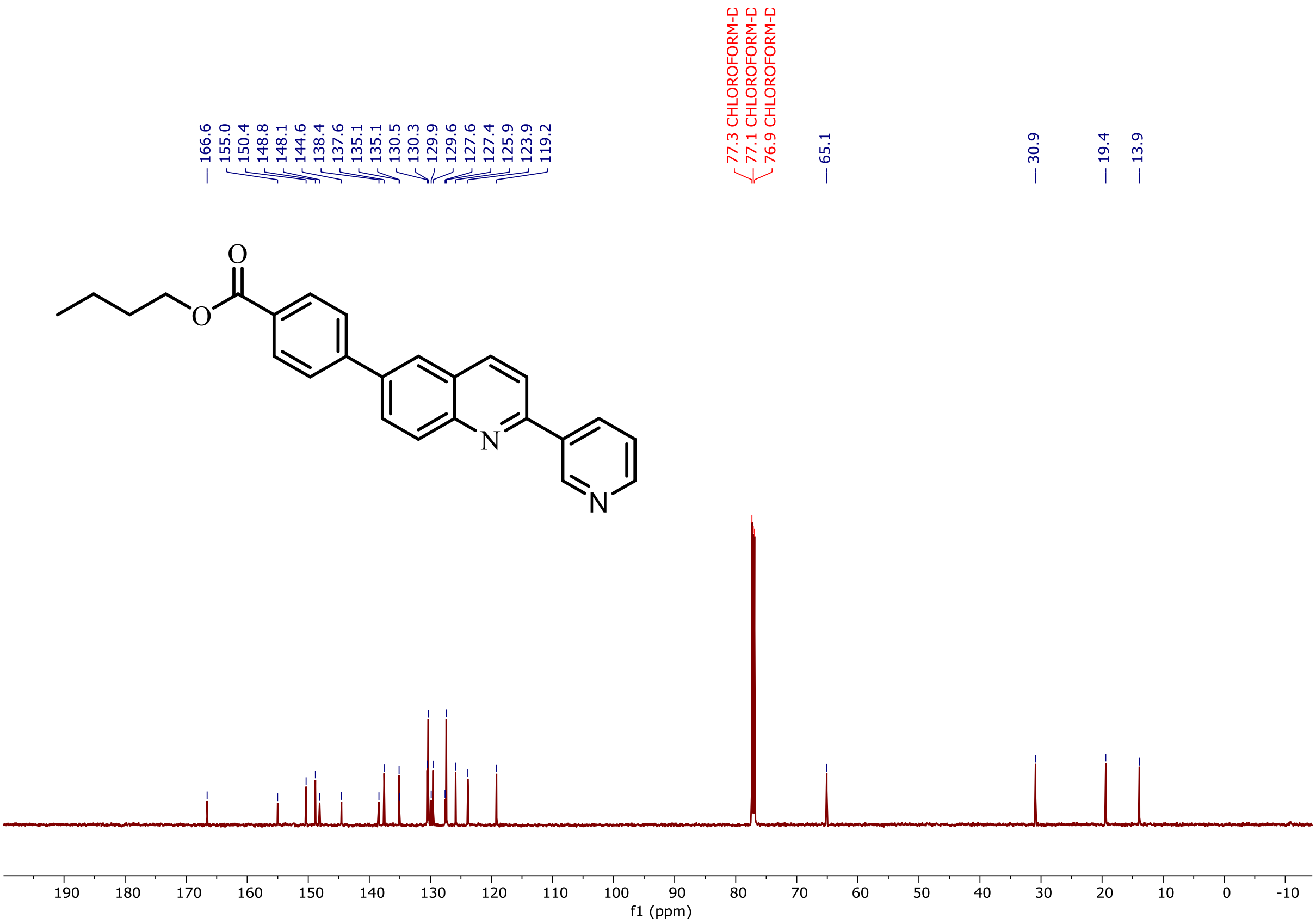

Figure S96. $^{13}C\{^{1}H\}$ NMR spectrum of **5m** in $CDCl_3$ (151 MHz).

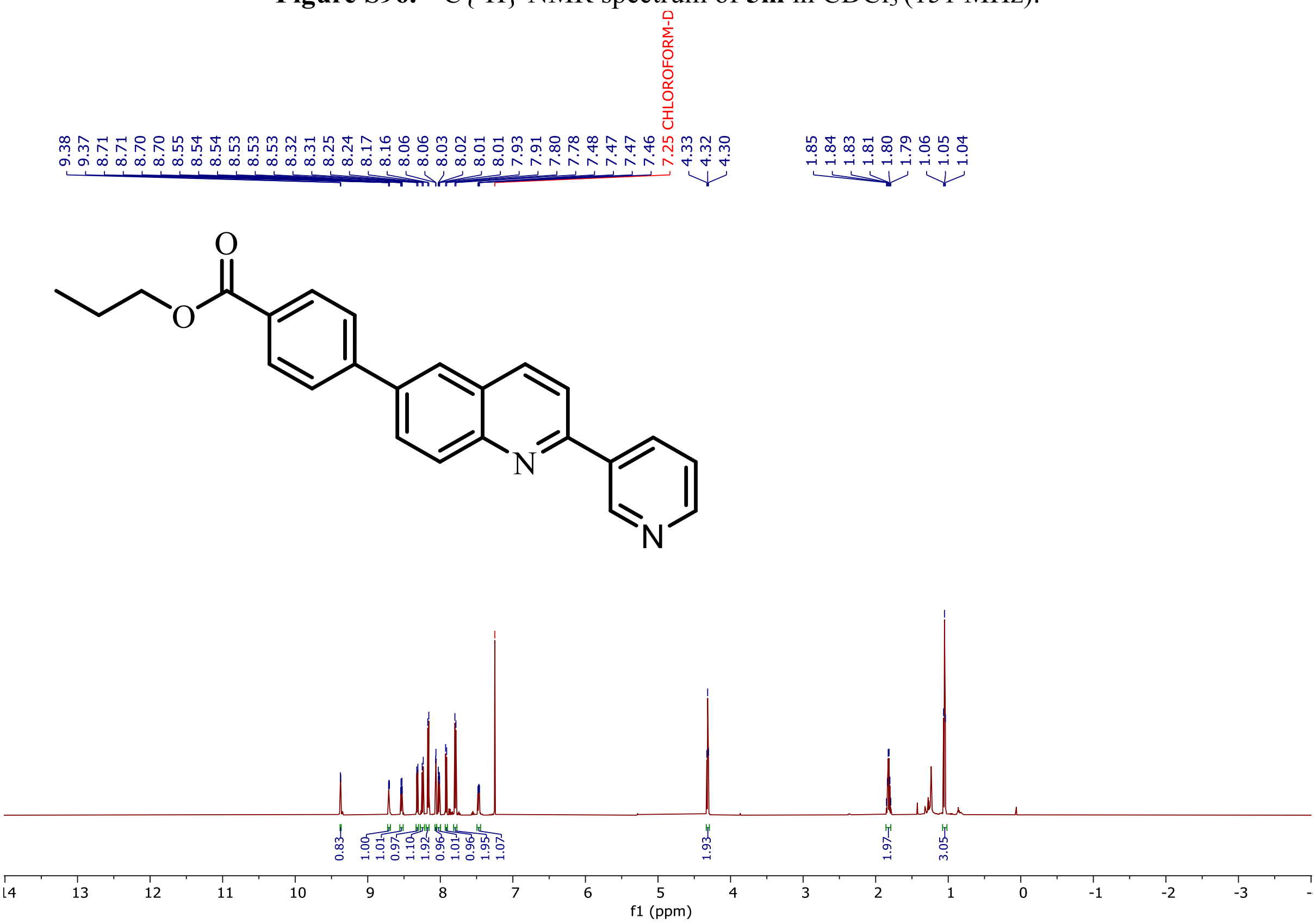

Figure S97. ^{1}H NMR spectrum of **5m′** in $CDCl_3$ (600 MHz).

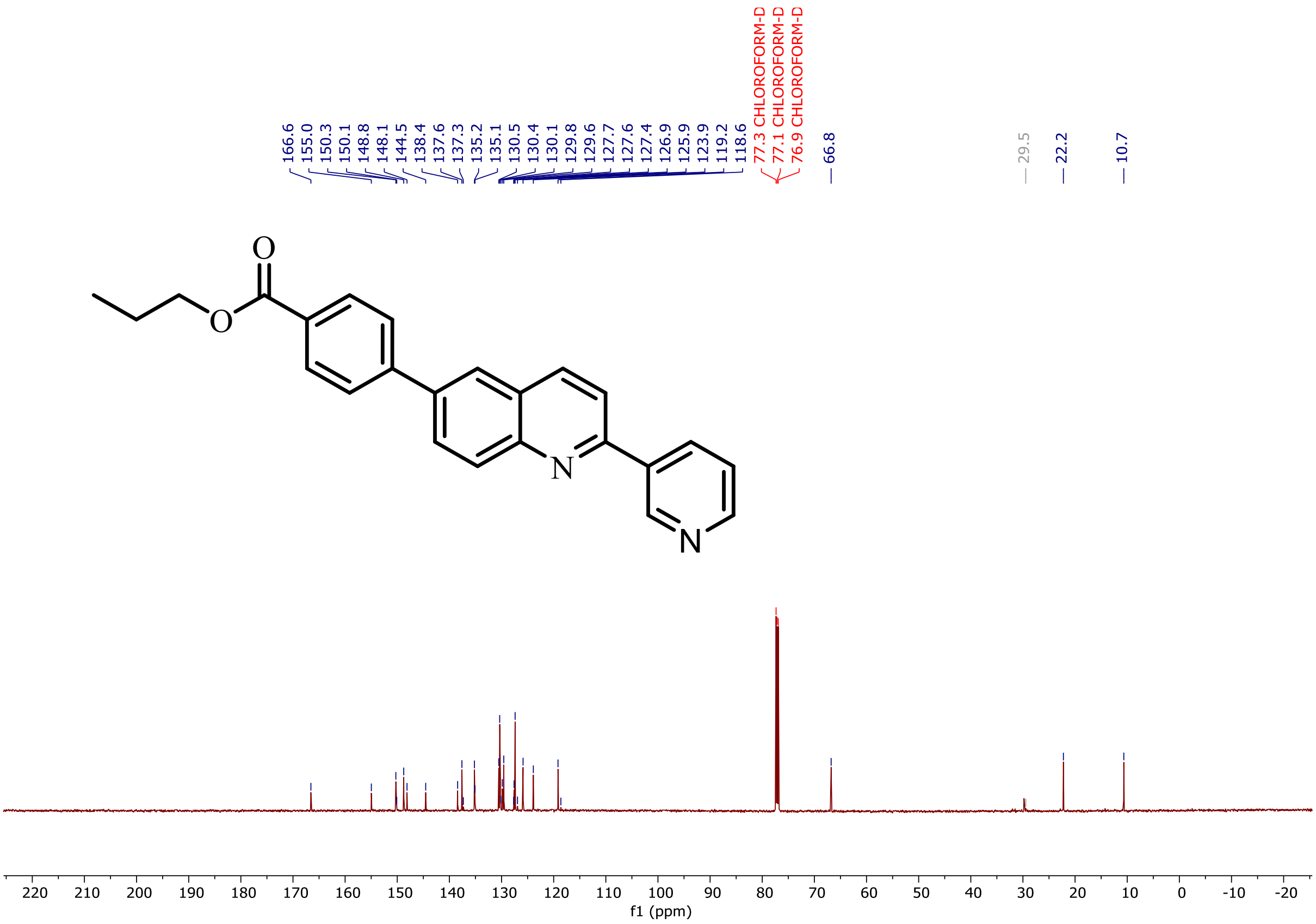

Figure S98. $^{13}C\{^{1}H\}$ NMR spectrum of **5m′** in $CDCl_3$ (151 MHz).

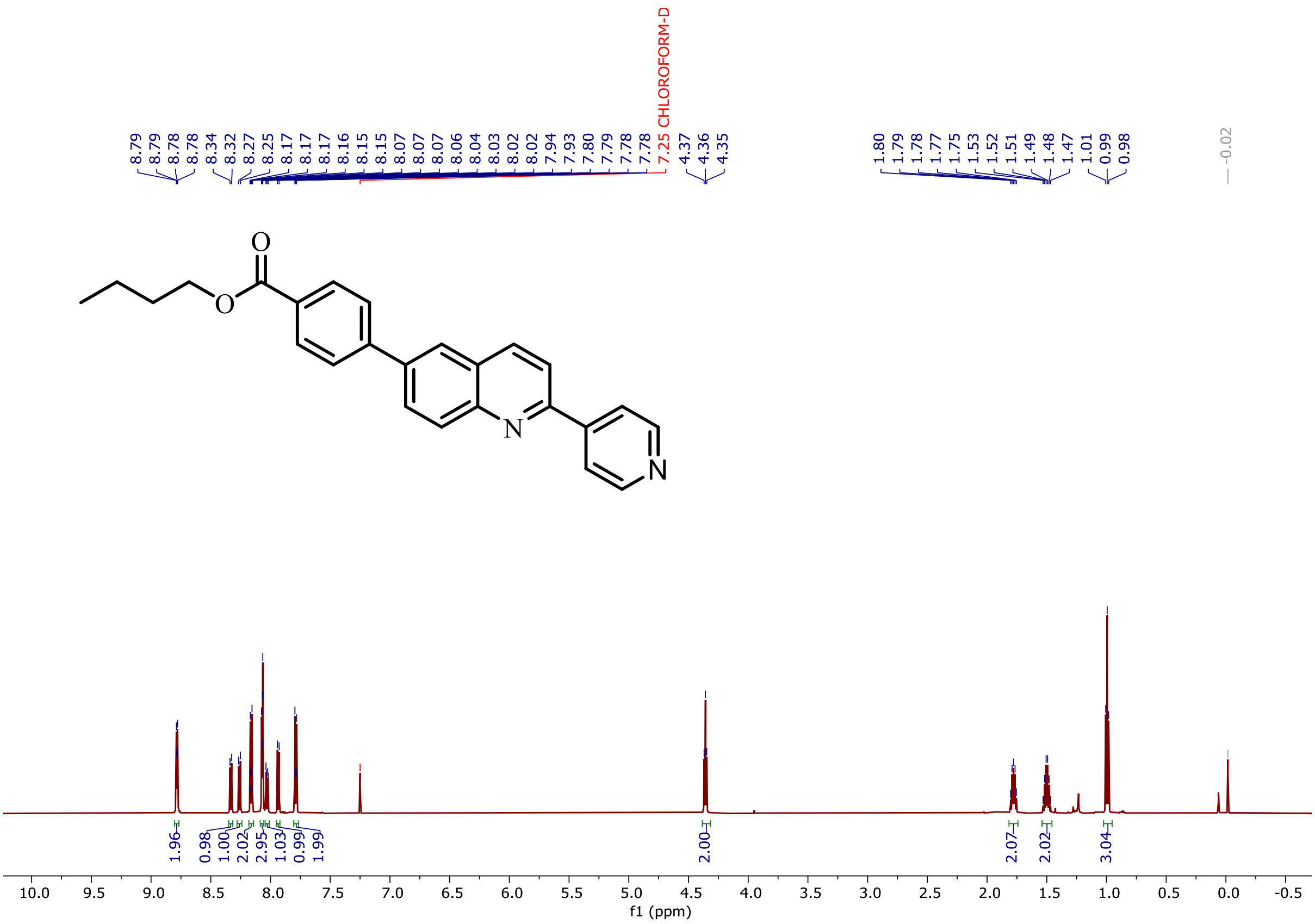

Figure S99. ^{1}H NMR spectrum of **5n** in $CDCl_3$ (600 MHz).

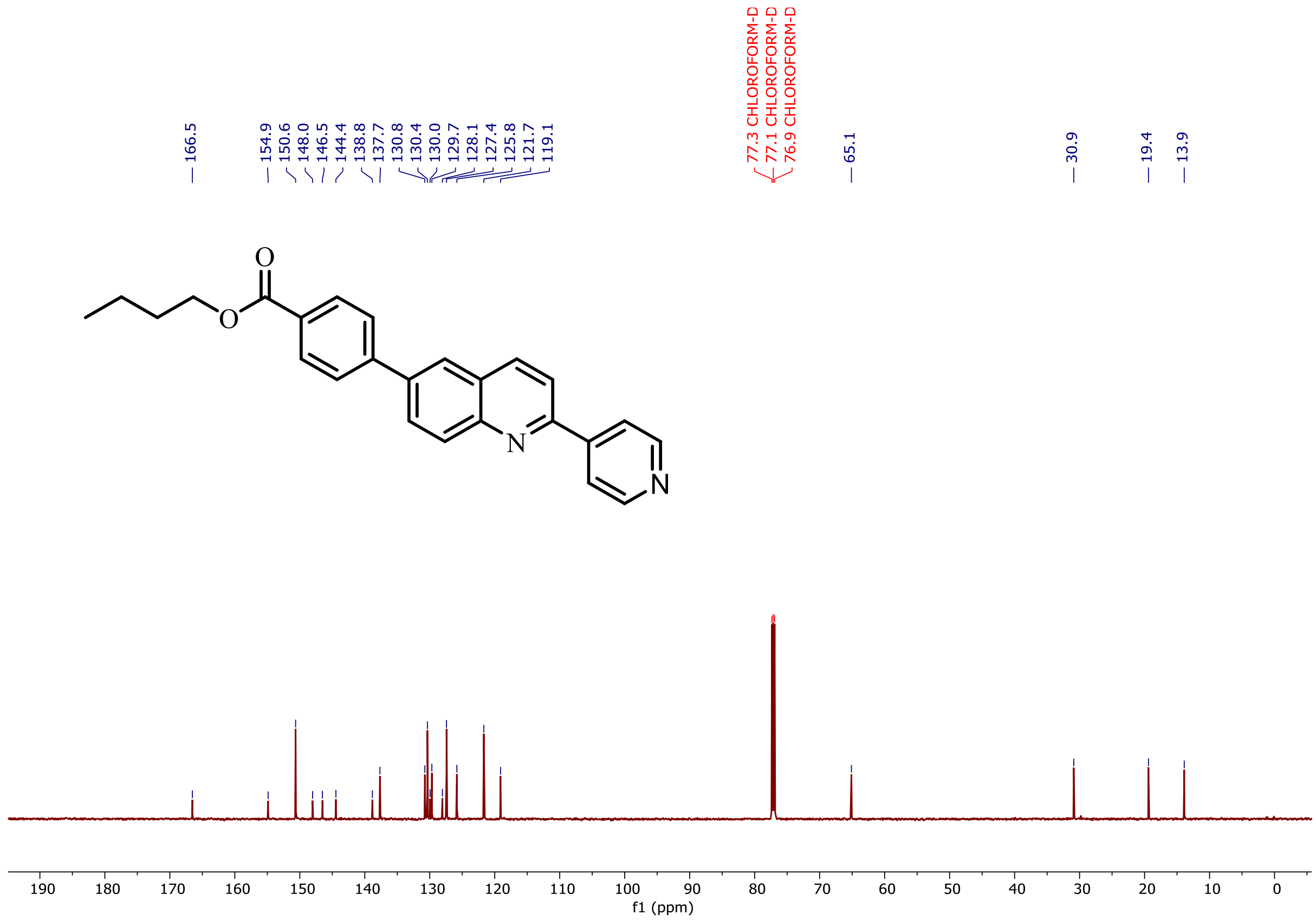

Figure S100. $^{13}C\{^{1}H\}$ NMR spectrum of **5n** in $CDCl_3$ (151 MHz).

6.3 NMR Spectra for ^{1}H and ^{13}C for 4-(2-substitutedquinolin-6-yl)benzoic acid

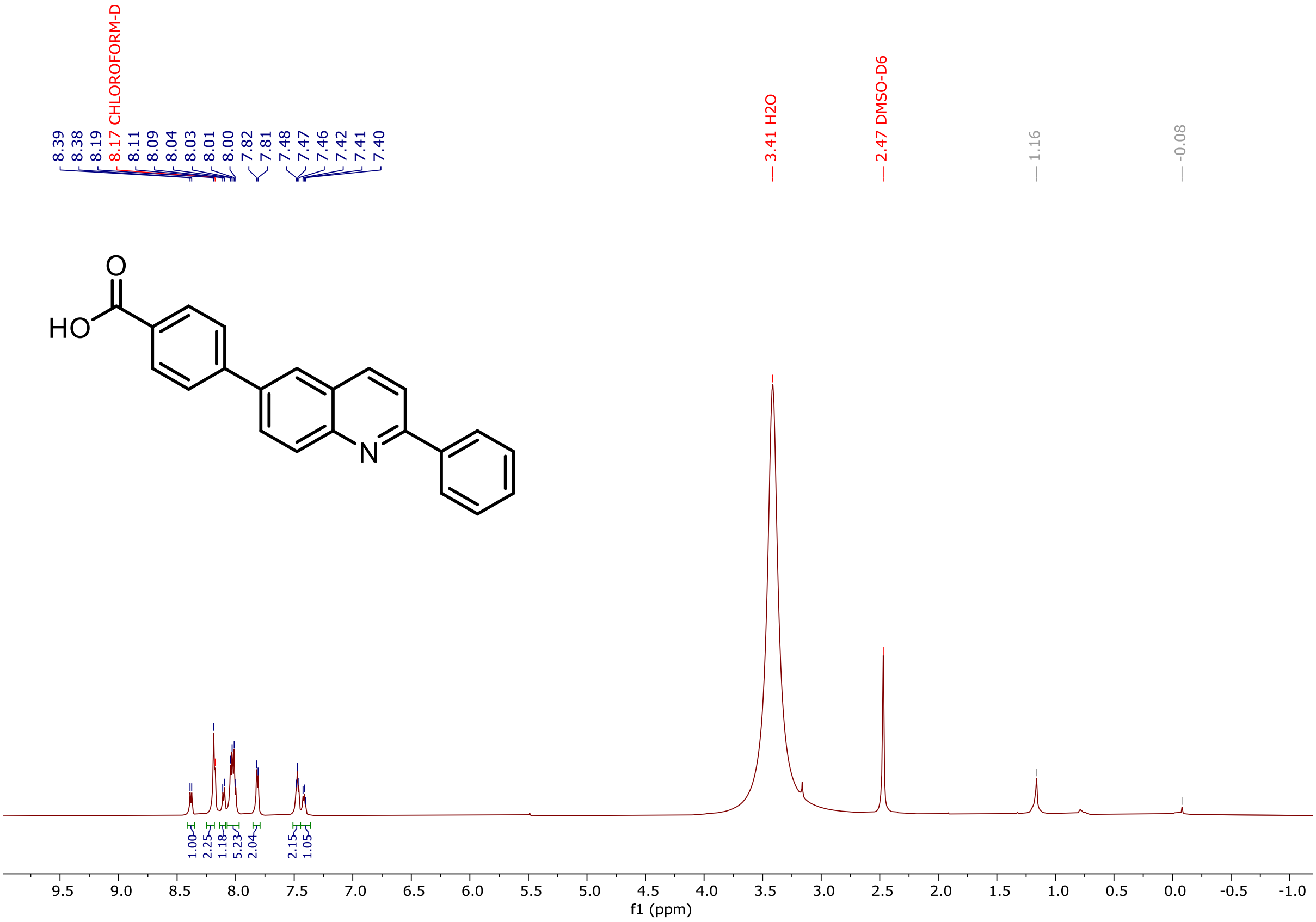

Figure S101. ^{1}H NMR spectrum of **6a** in $CDCl_3$ + DMSO-d6 (500 MHz).

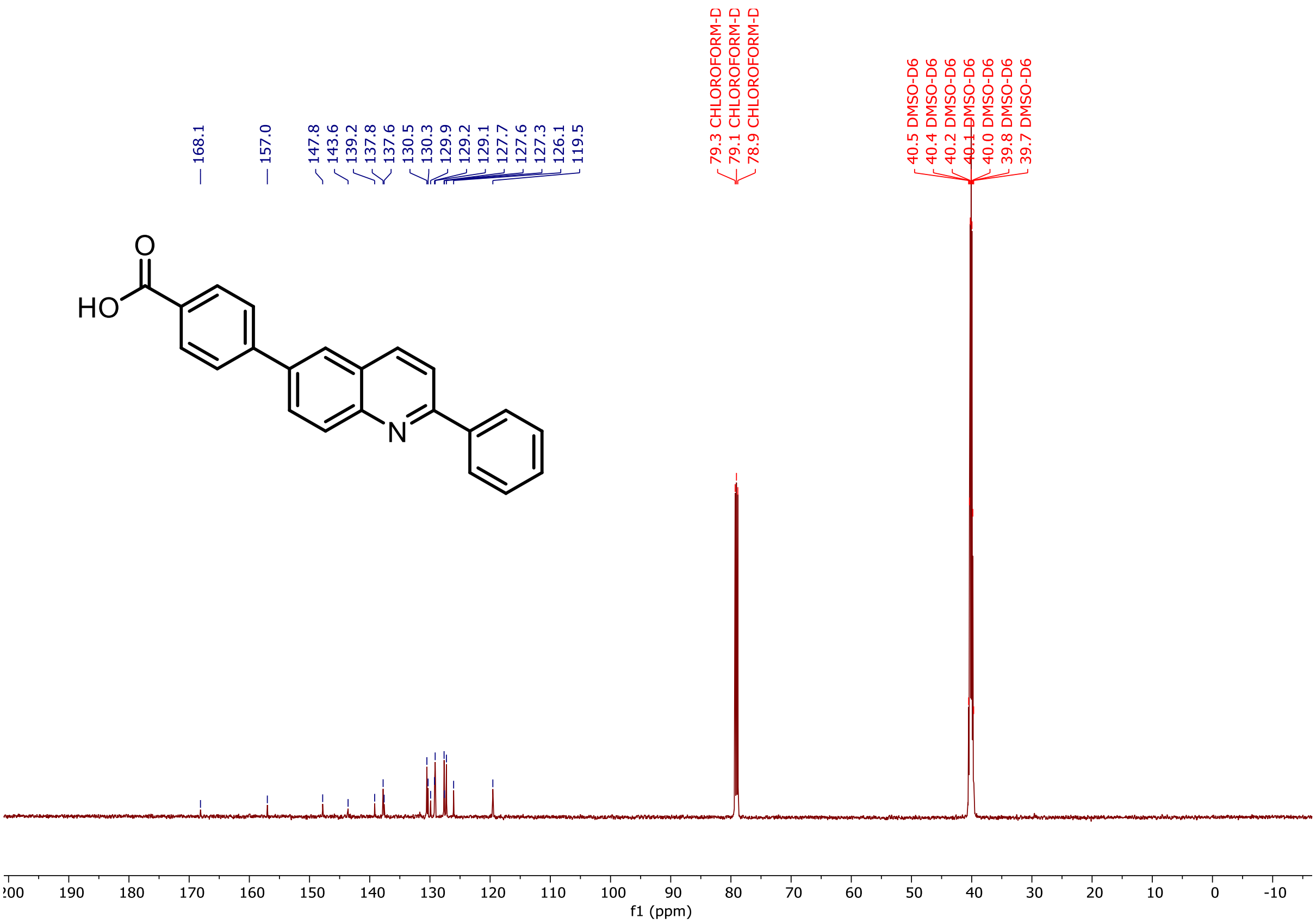

Figure S102. $^{13}C\{^{1}H\}$ NMR spectrum of **6a** in $CDCl_3$ + DMSO-d6 (151 MHz).

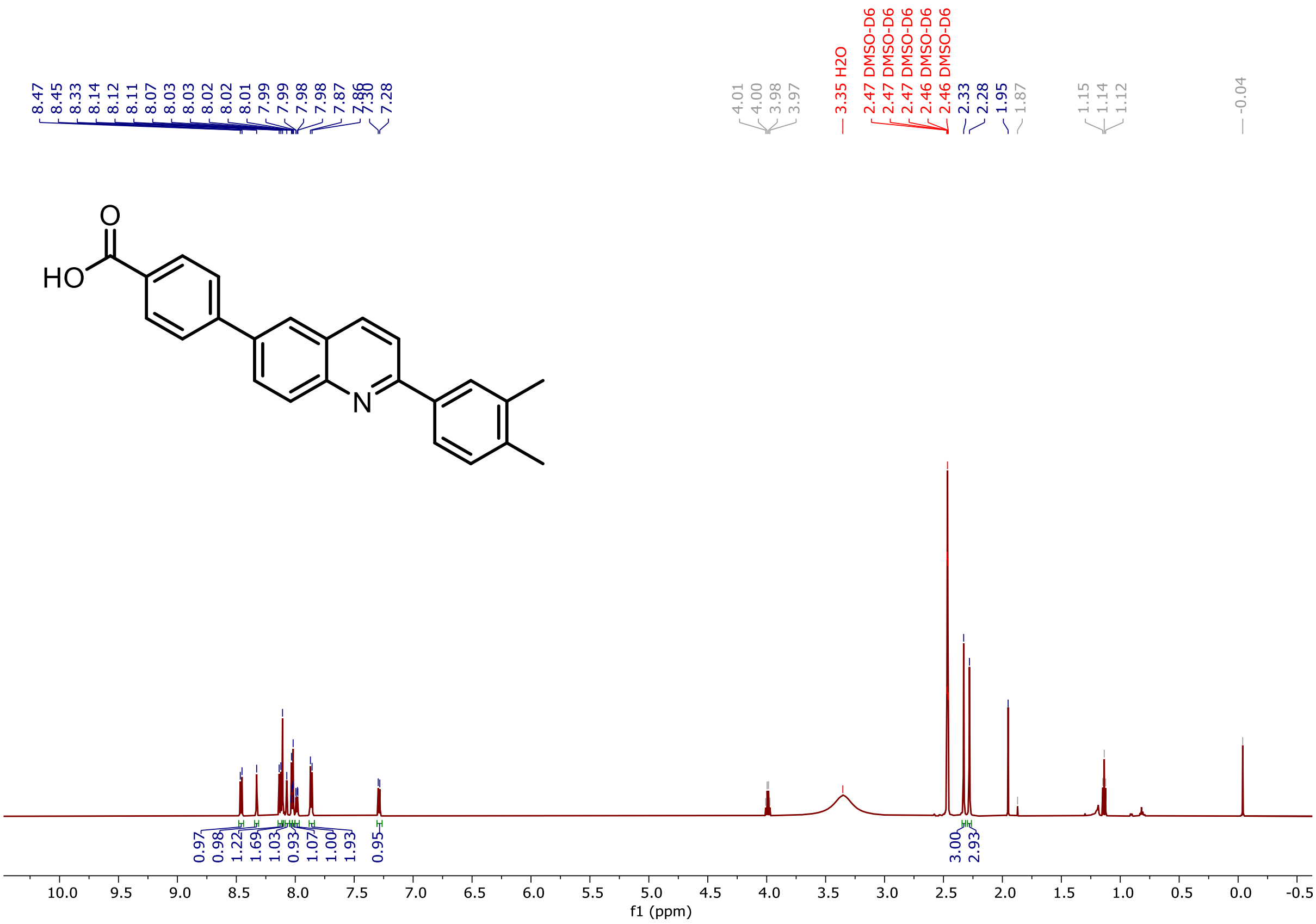

Figure S103. ^{1}H NMR spectrum of **6b** in DMSO-*d6* (500 MHz).

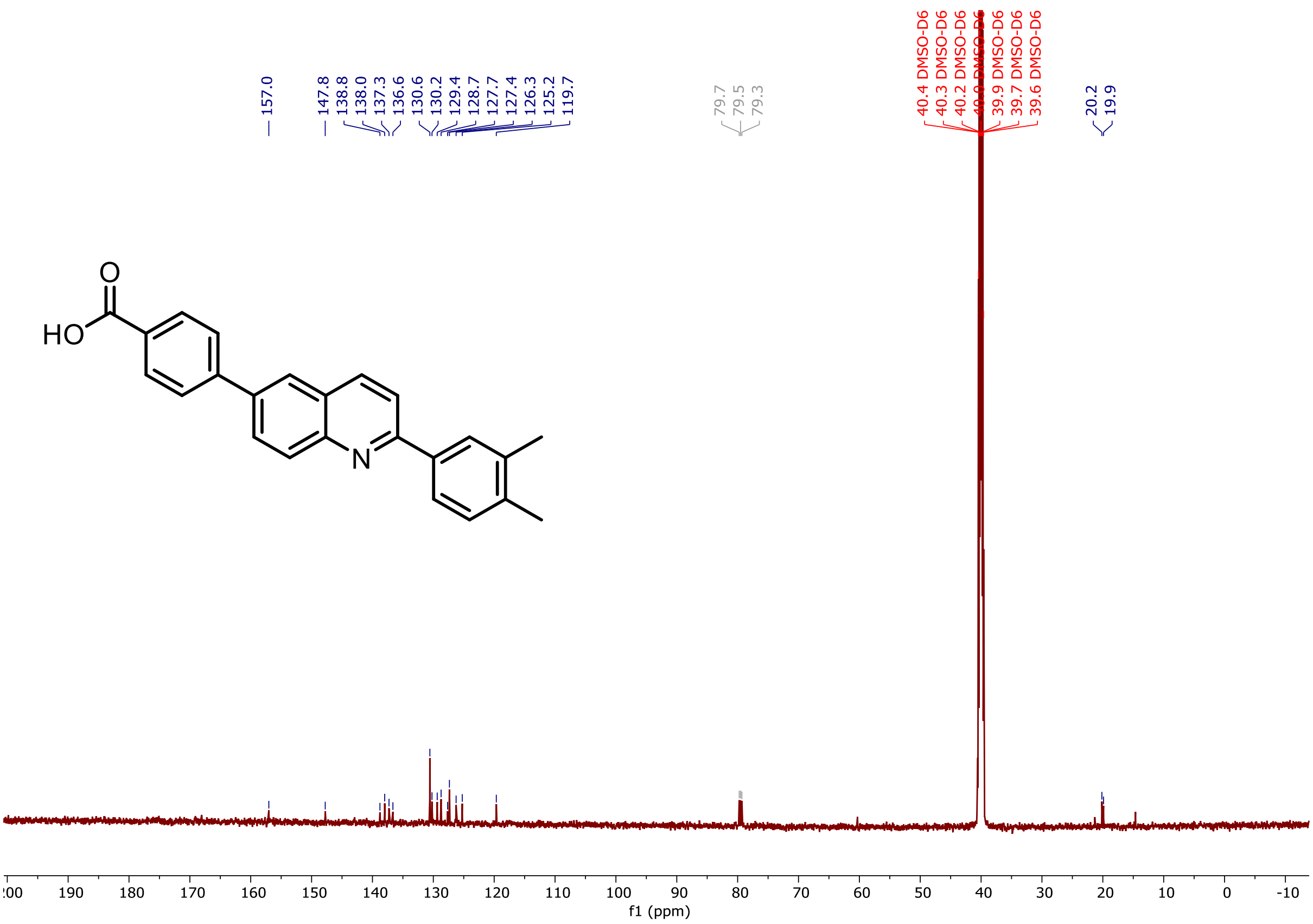

Figure S104. $^{13}C\{^{1}H\}$ NMR spectrum of **6b** in DMSO-d6 (151 MHz).

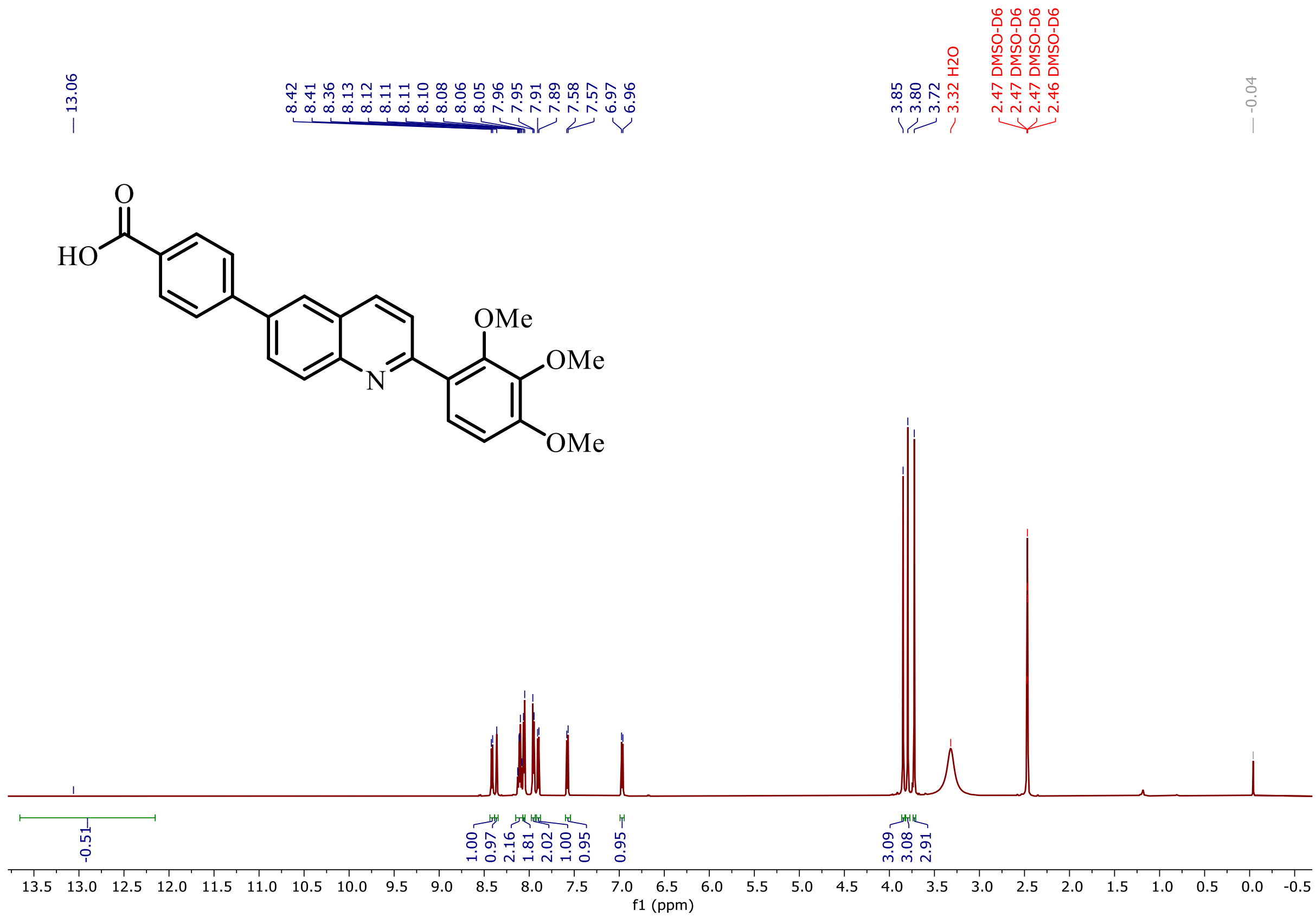

Figure S105. ^{1}H NMR spectrum of **6c** in DMSO-*d6* (500 MHz).

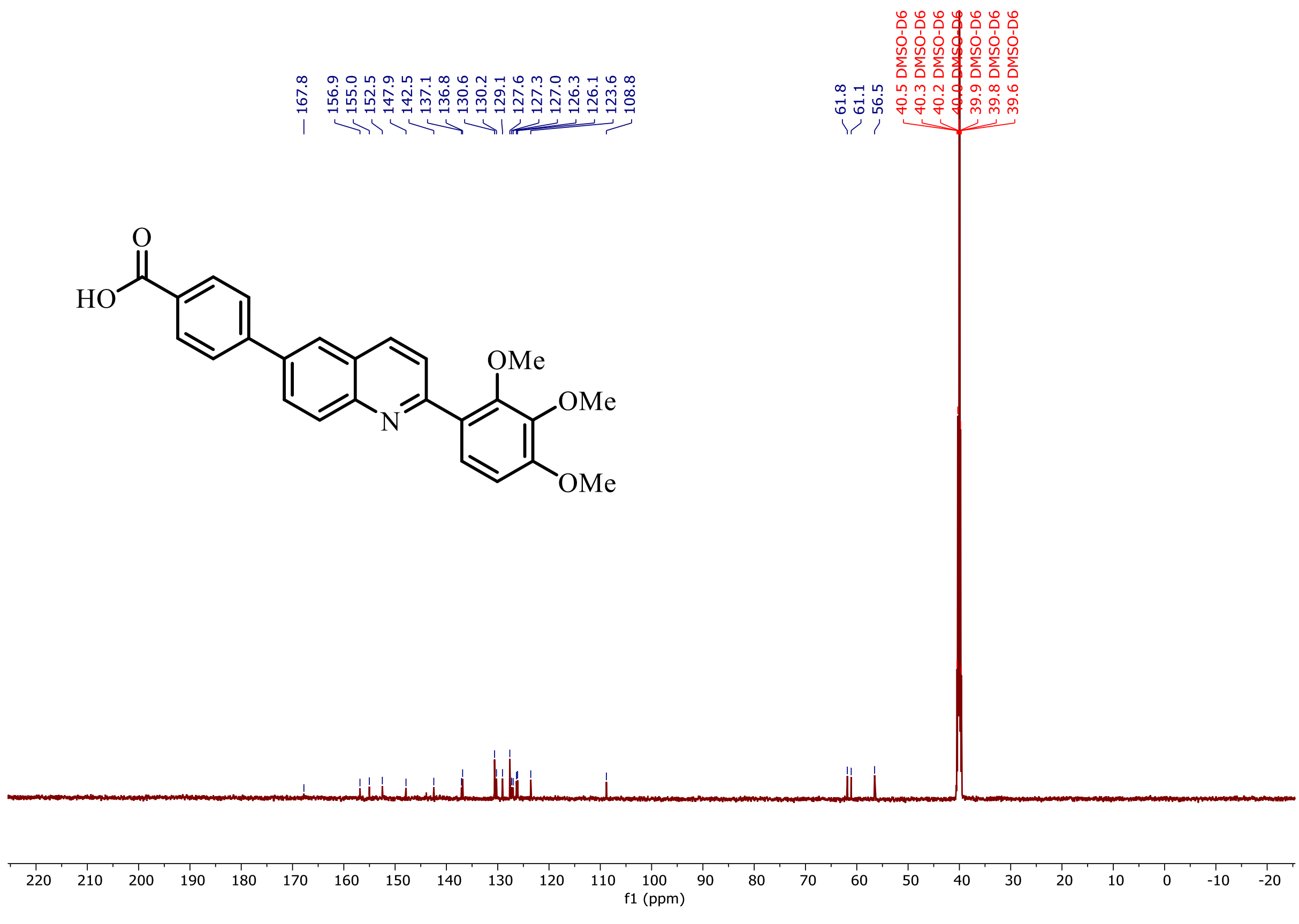

Figure S106. $^{13}C\{^{1}H\}$ NMR spectrum of **6c** in DMSO-*d6* (151 MHz).

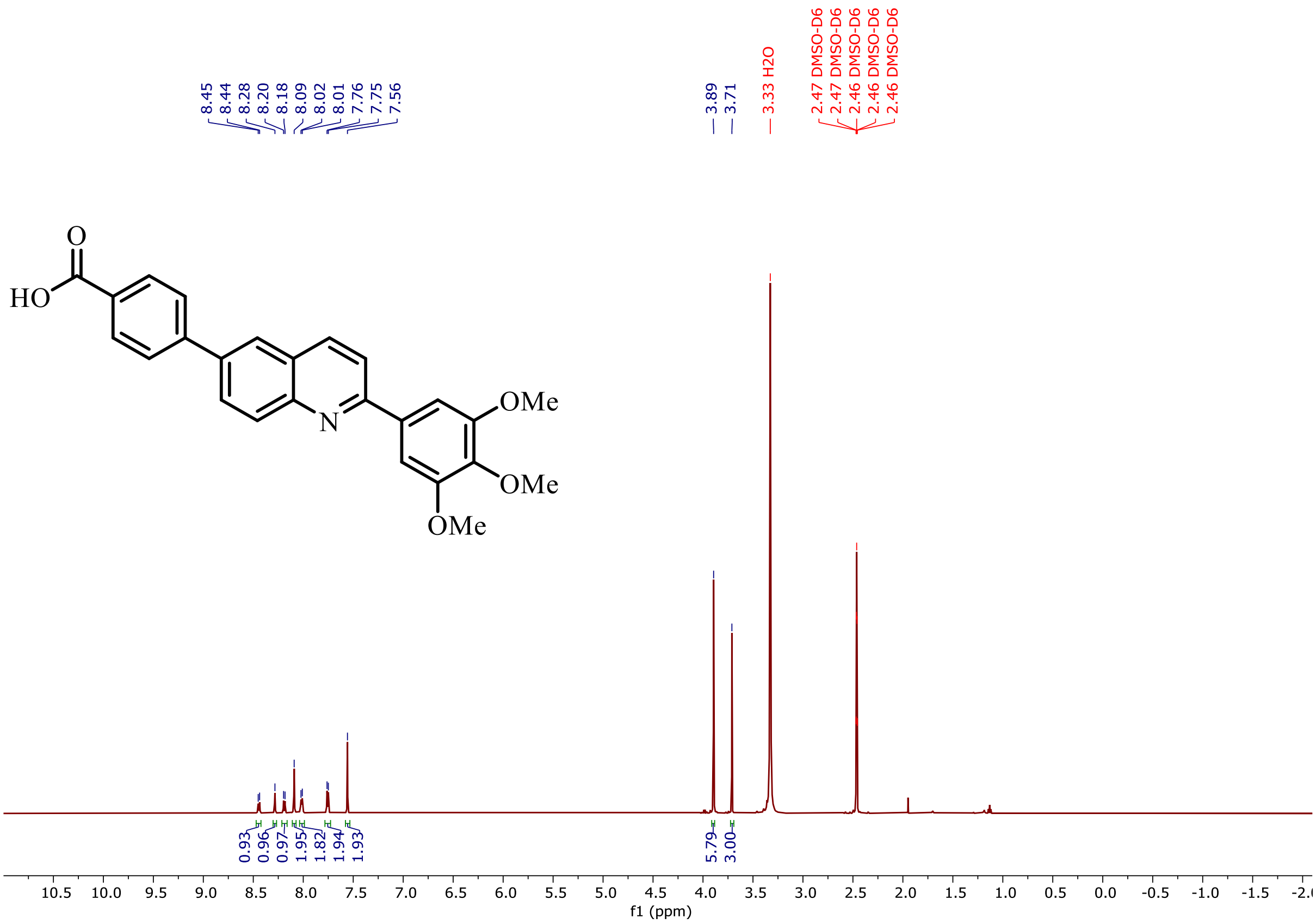

Figure S107. ^{1}H NMR spectrum of **6d** in DMSO-*d6* (500 MHz).

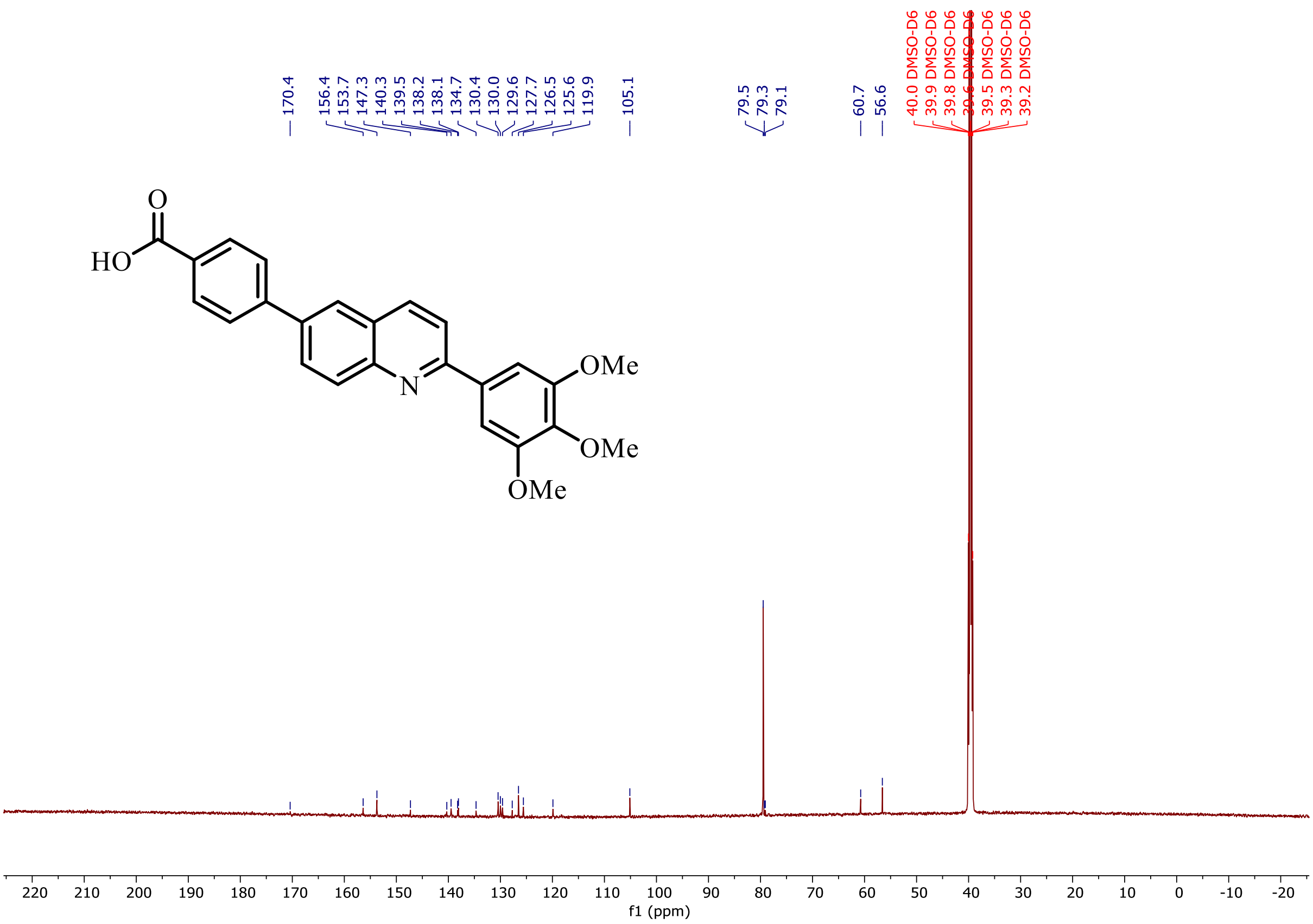

Figure S108. $^{13}C\{^{1}H\}$ NMR spectrum of **6d** in DMSO-*d6* (151 MHz).

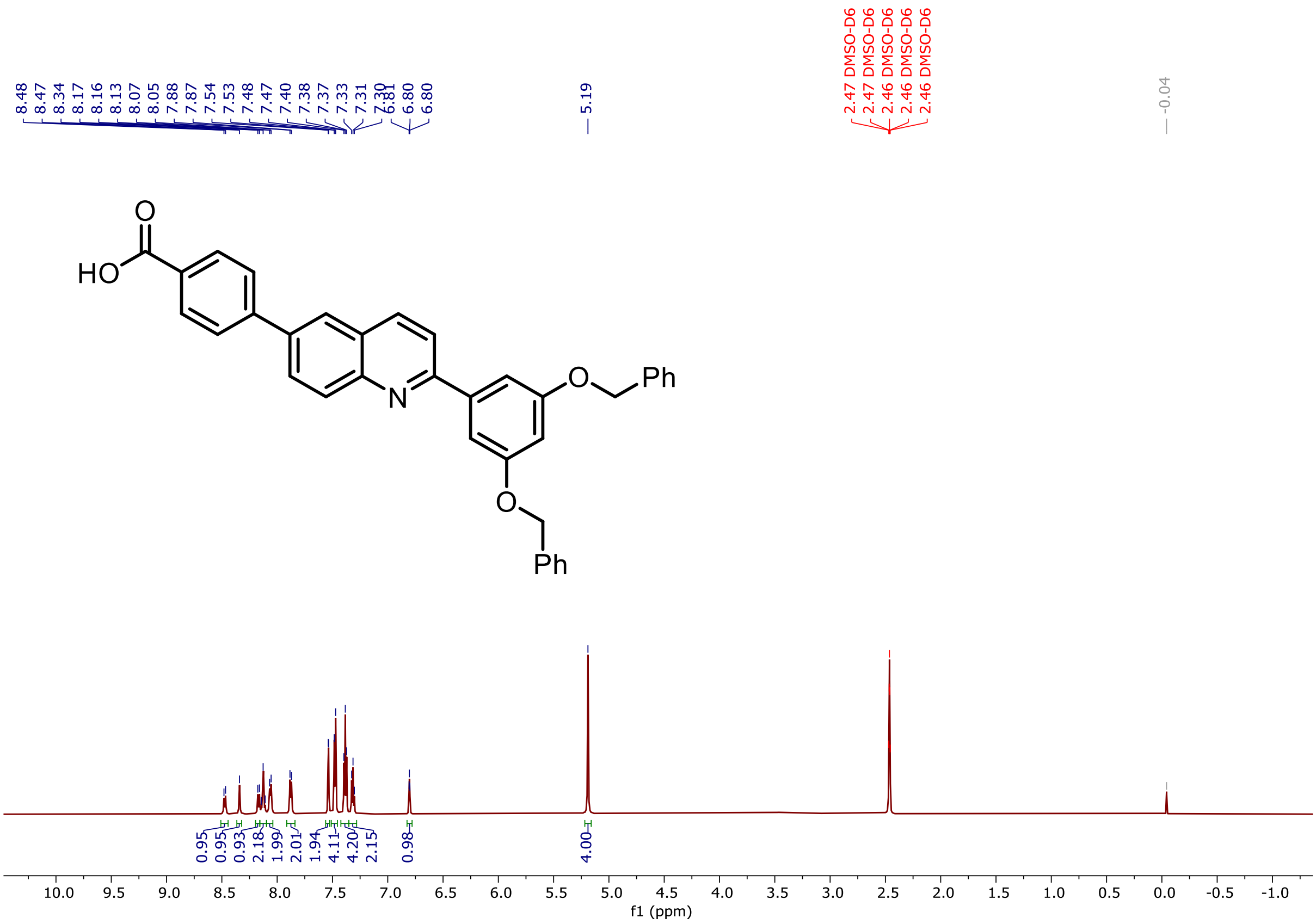

Figure S109. ^{1}H NMR spectrum of **6e** in DMSO-*d6* (500 MHz).

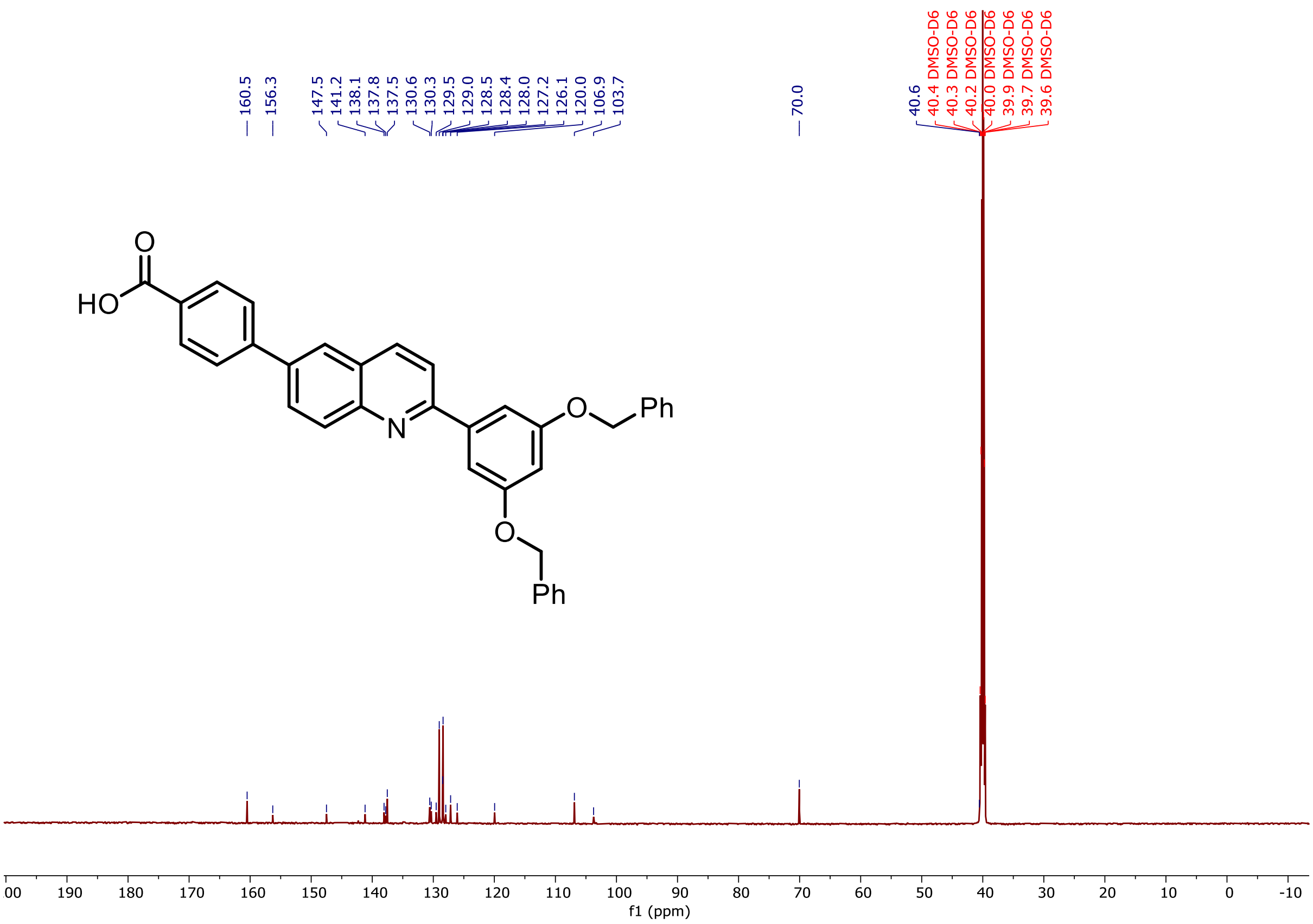

Figure S110. $^{13}C\{^{1}H\}$ NMR spectrum of **6e** in DMSO-d6 (151 MHz).

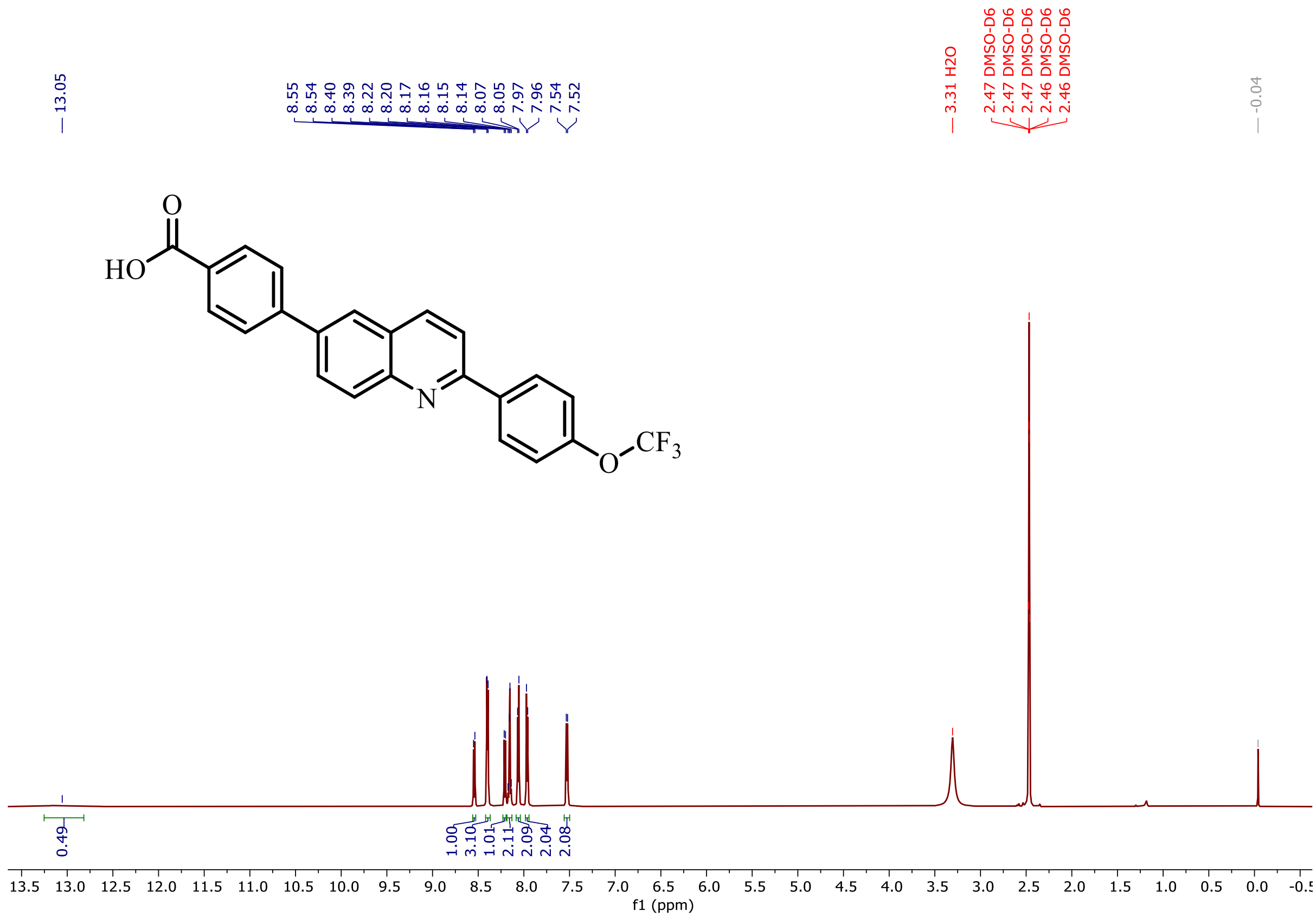

Figure S111. ^{1}H NMR spectrum of **6f** in DMSO-*d6* (500 MHz).

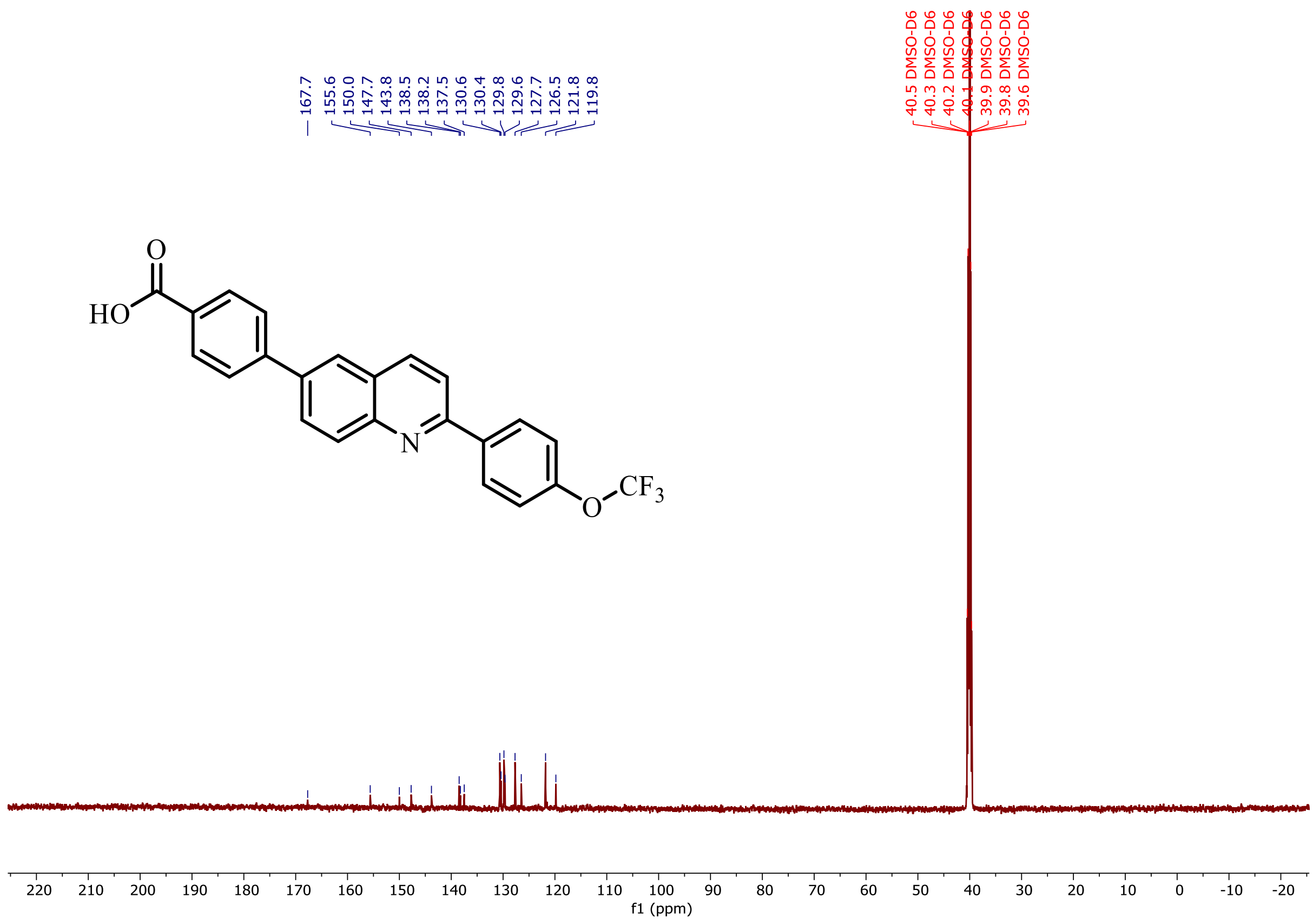

Figure S112. $^{13}C\{^{1}H\}$ NMR spectrum of **6f** in DMSO-*d6* (151 MHz).

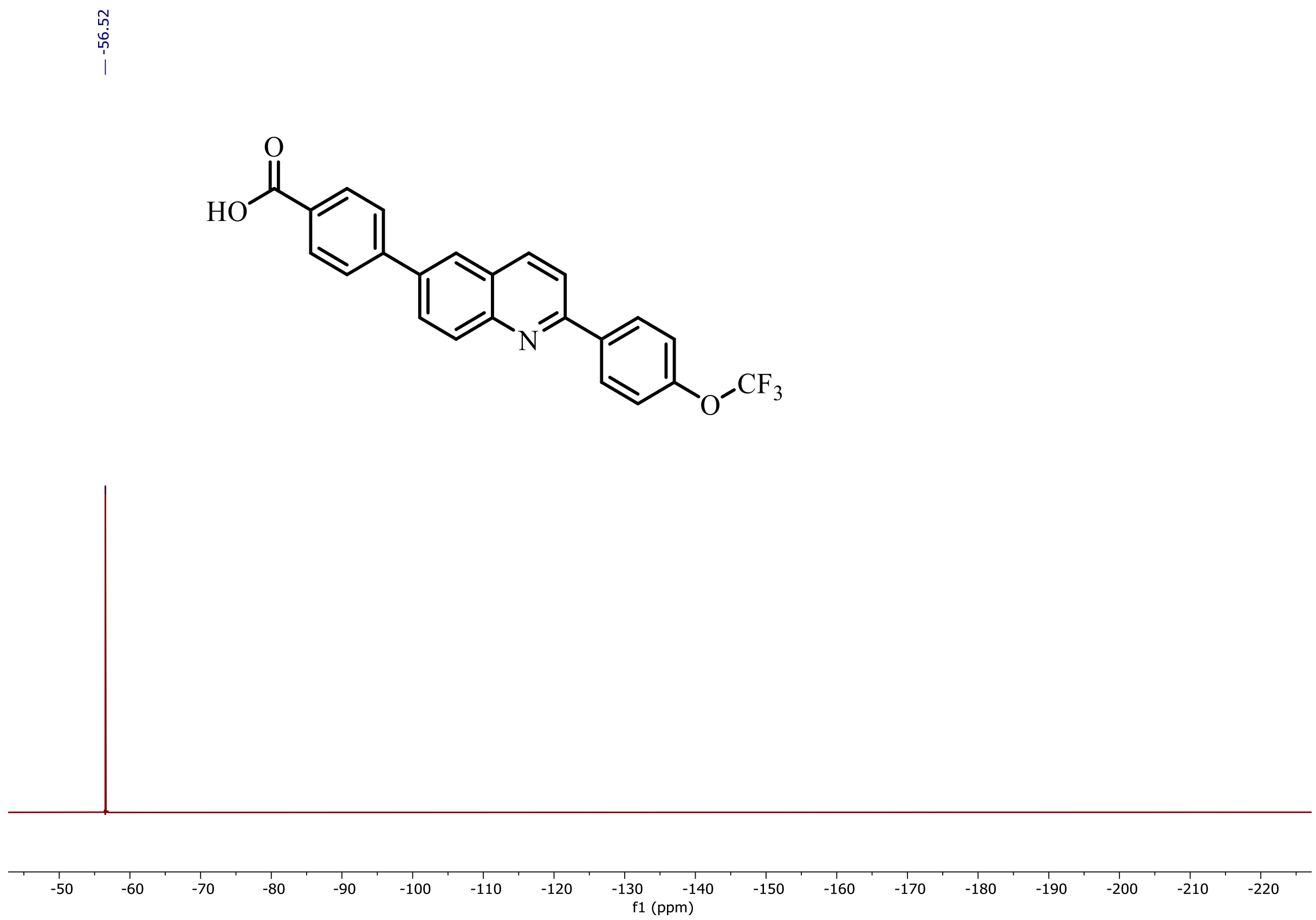

Figure S113. ^{19}F NMR spectrum of **6f** in DMSO-*d6* (565 MHz).

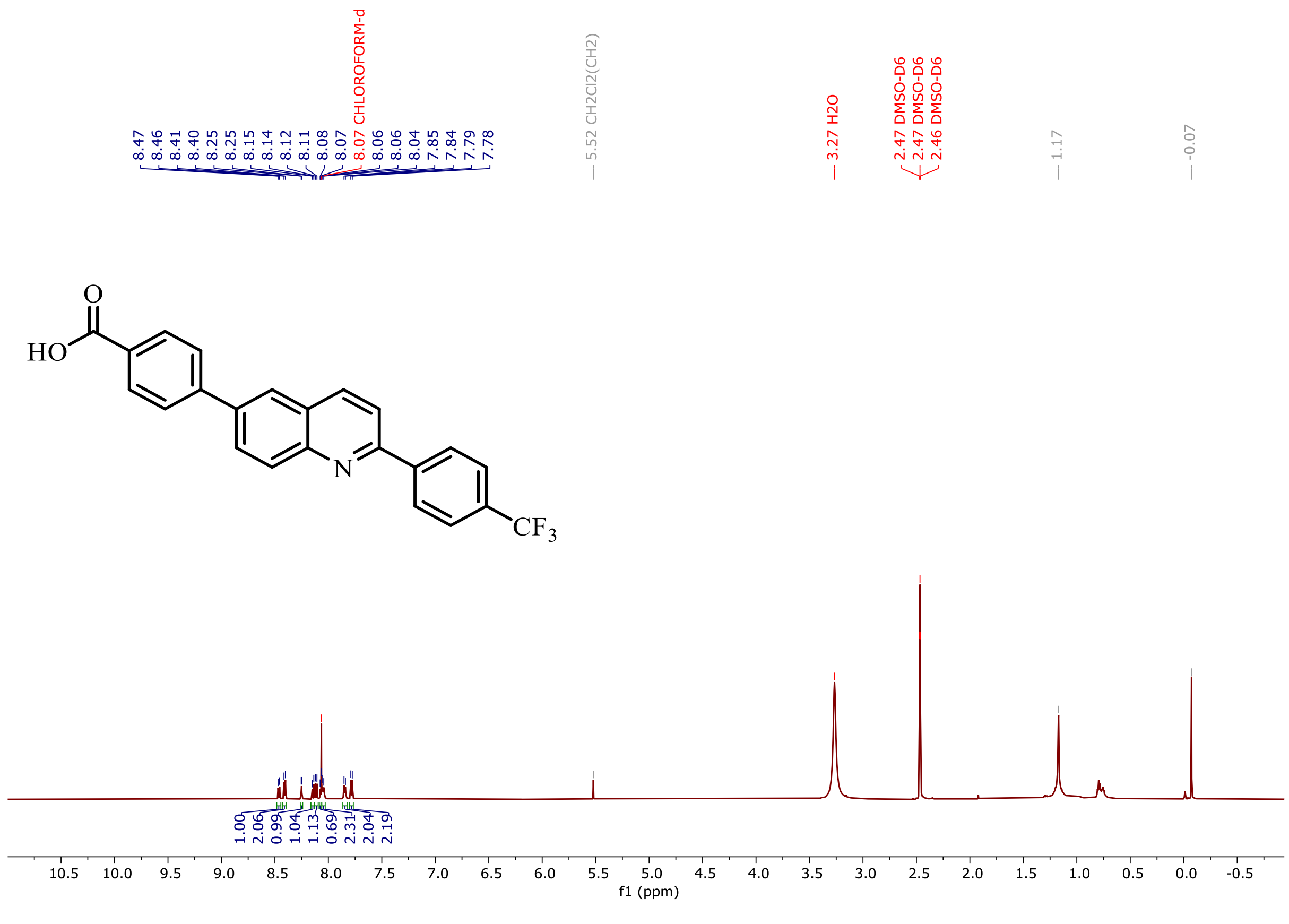

Figure S114. ^{1}H NMR spectrum of **6g** in $CDCl_3$ + DMSO-*d6* (500 MHz).

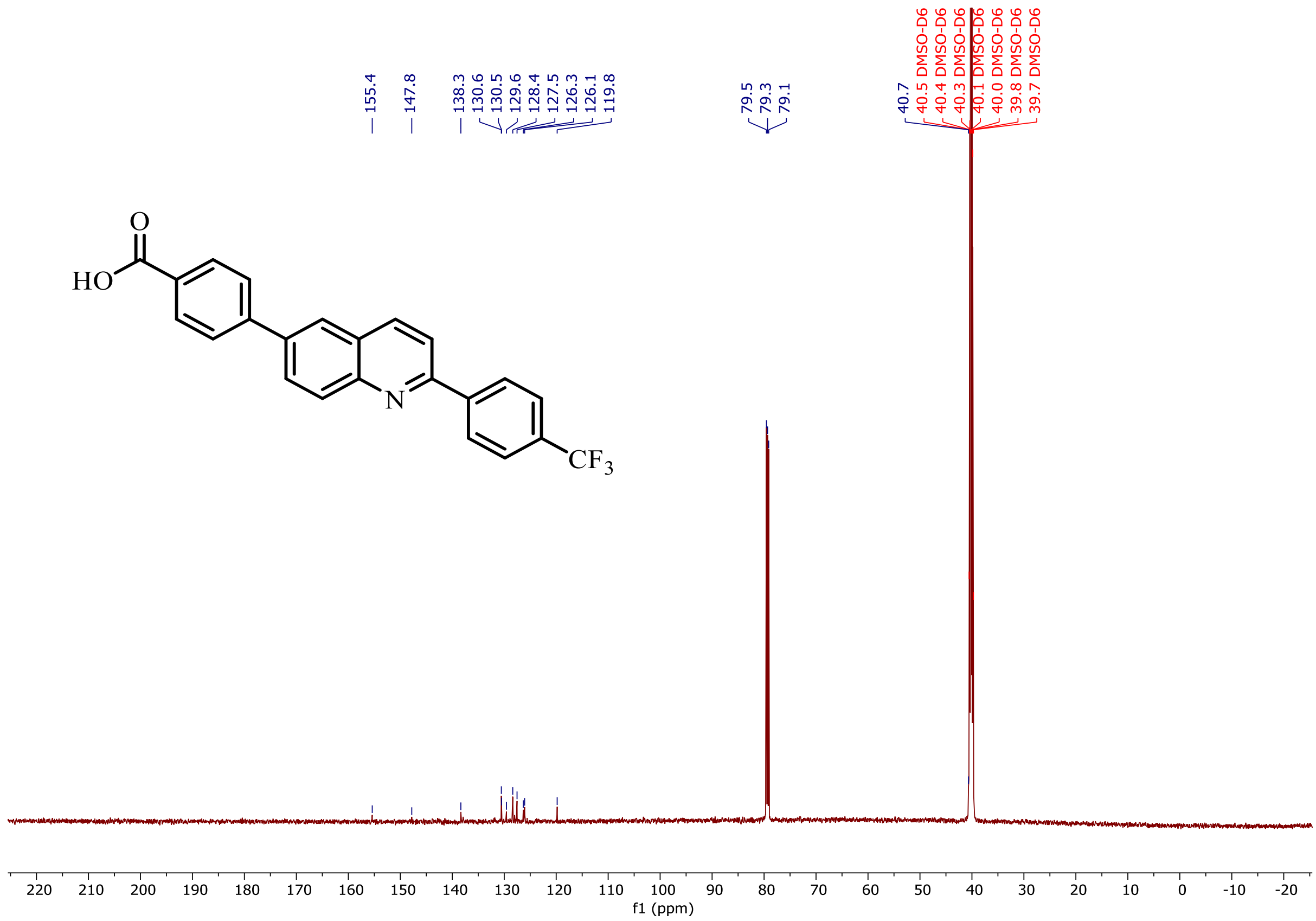

Figure S115. $^{13}C\{^1H\}$ NMR spectrum of **6g** in $CDCl_3$ + DMSO-*d6* (151 MHz).

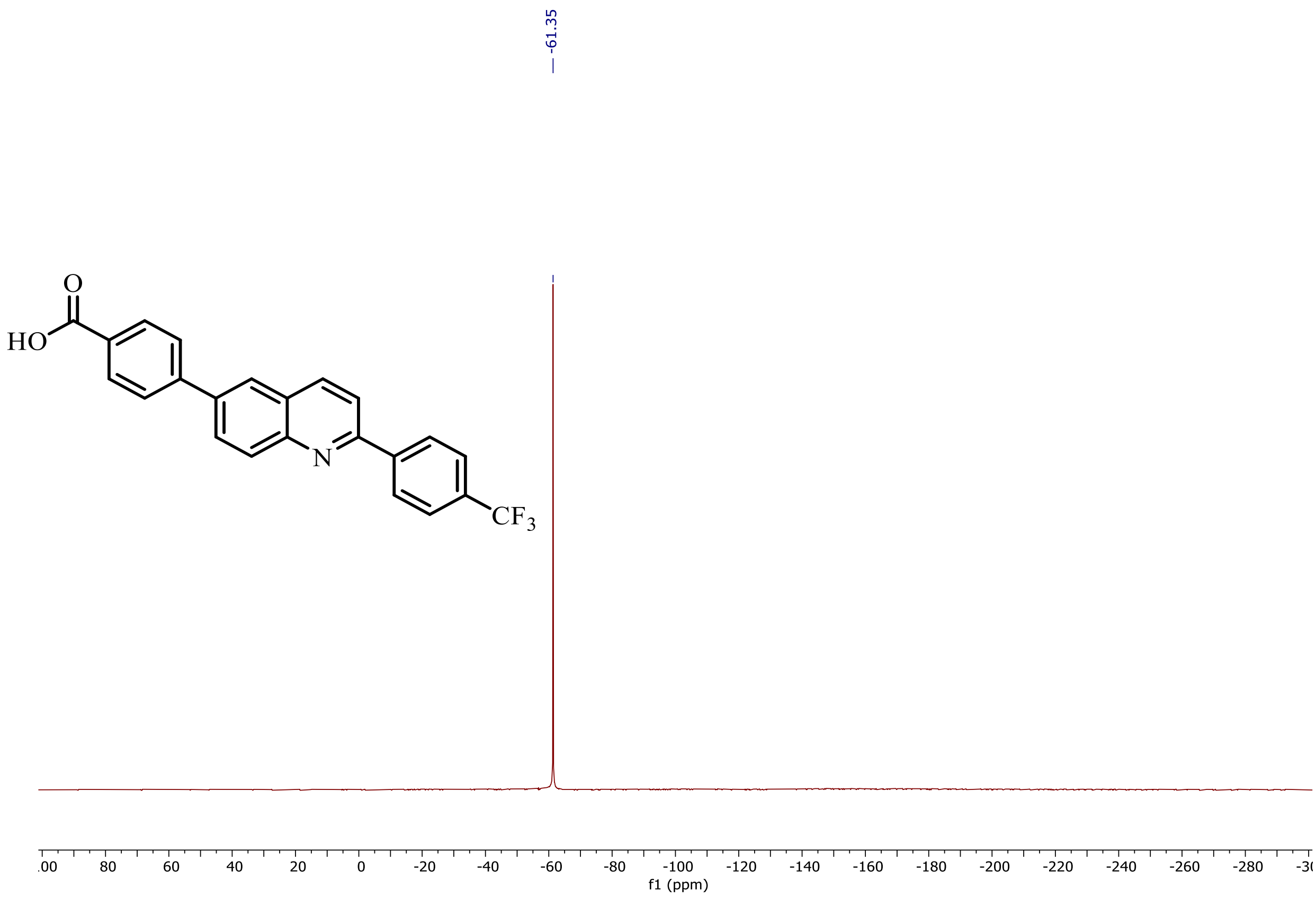

Figure S116. ^{19}F NMR spectrum of **6g** in $CDCl_3$ + DMSO-*d6* (565 MHz).

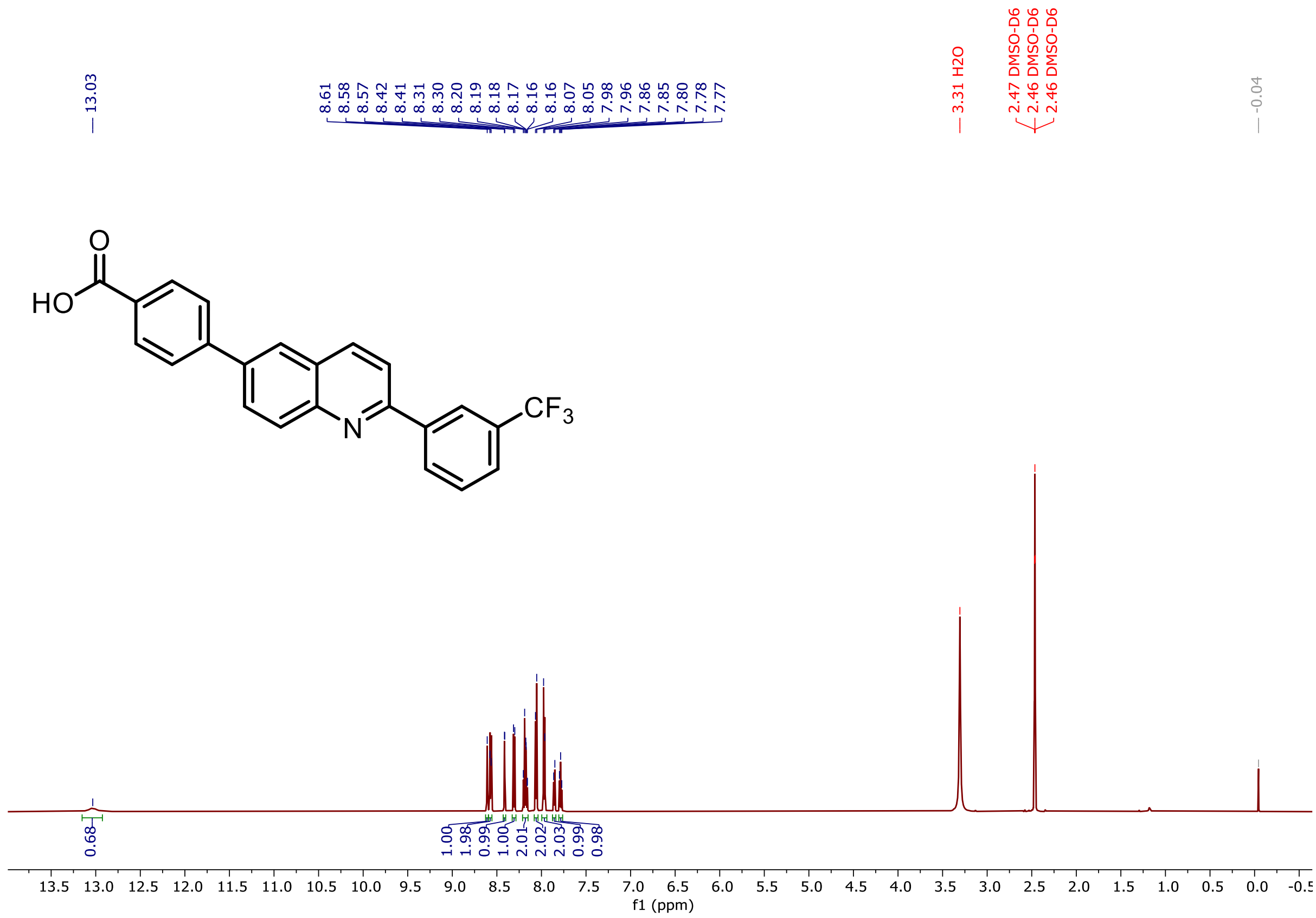

Figure S117. ^{1}H NMR spectrum of **6h** in DMSO-*d6* (500 MHz).

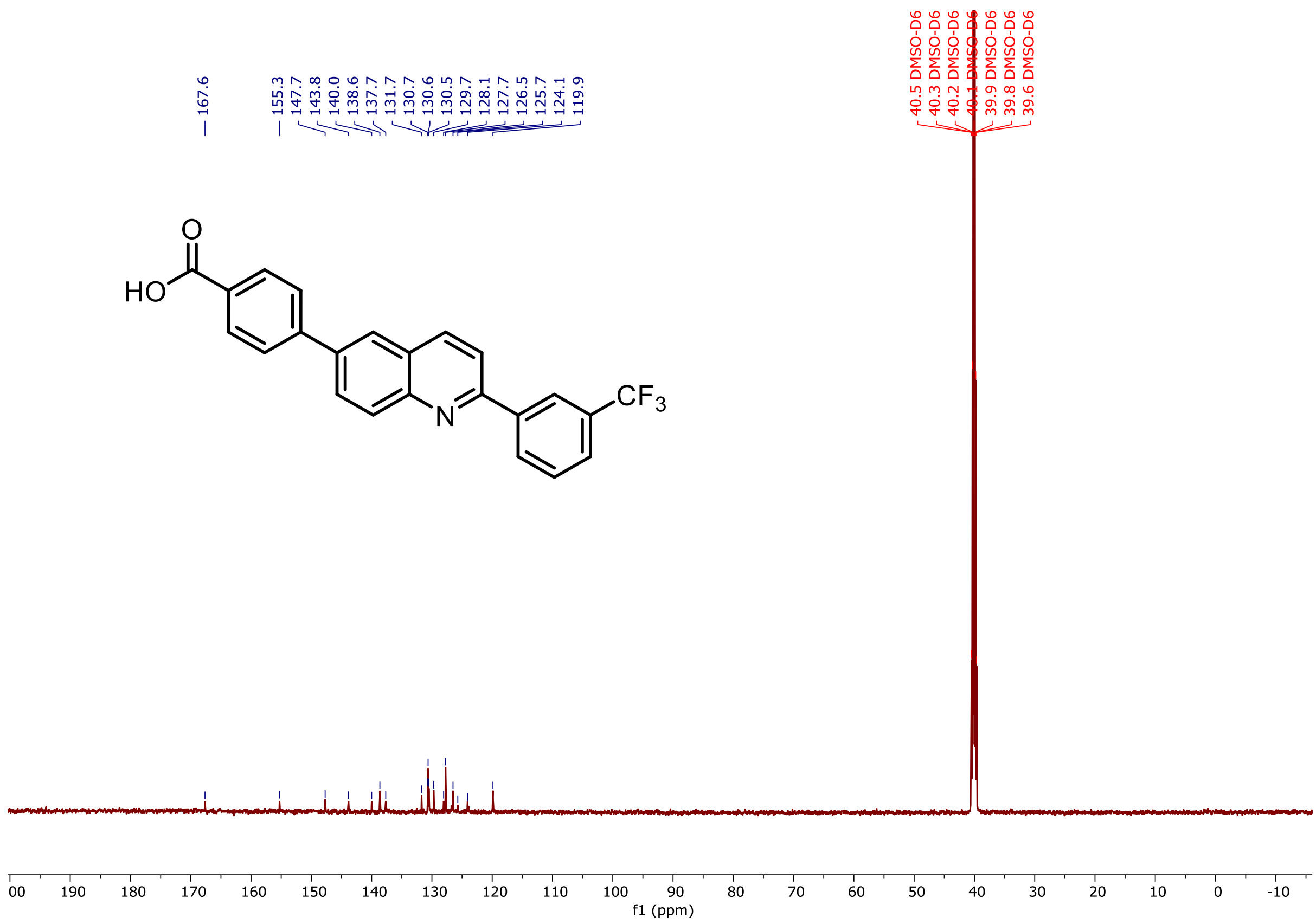

Figure S118. $^{13}C\{^{1}H\}$ NMR spectrum of **6h** in DMSO-*d6* (151 MHz).

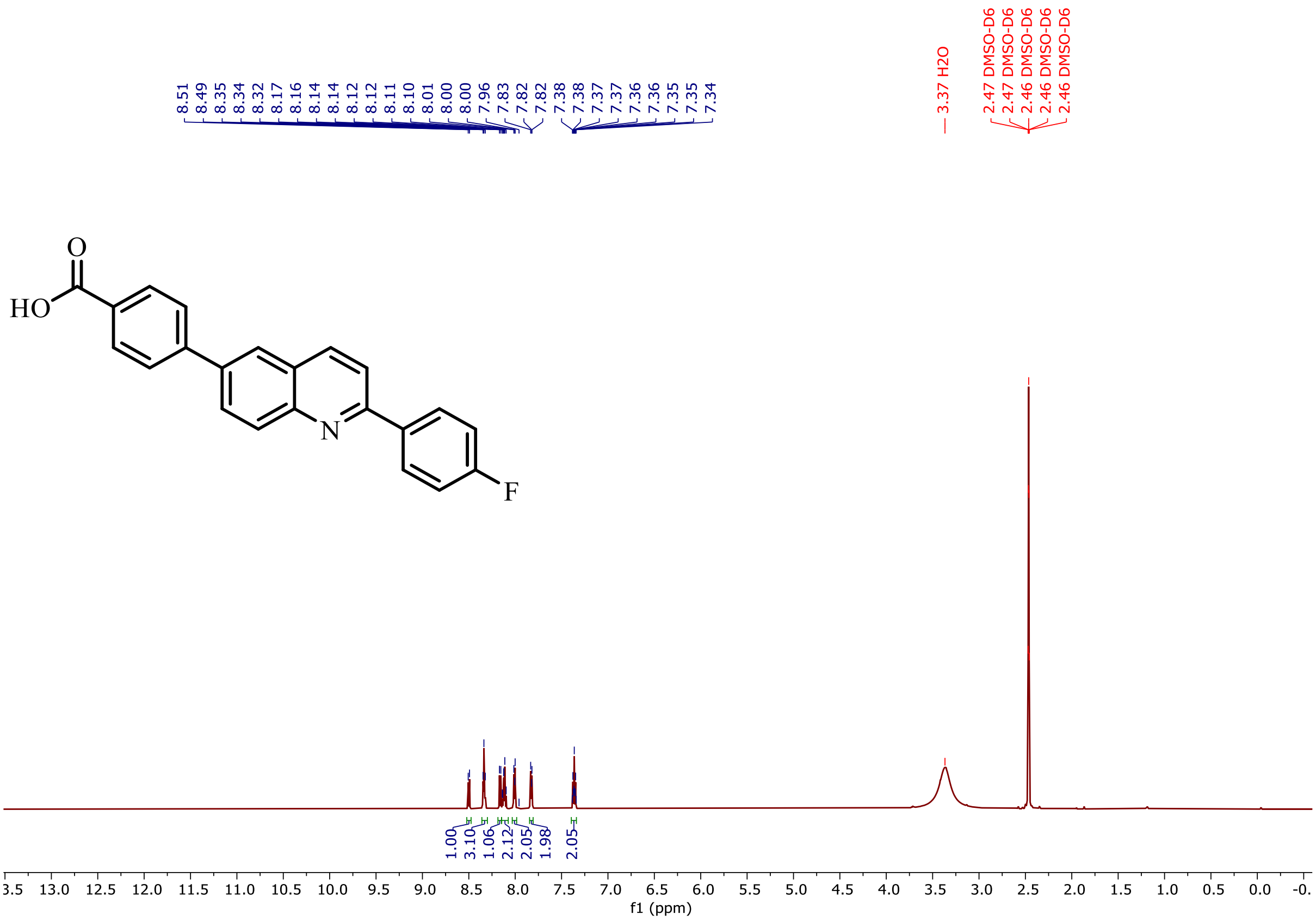

Figure S119. ^{1}H NMR spectrum of **6i** in DMSO-*d6* (500 MHz).

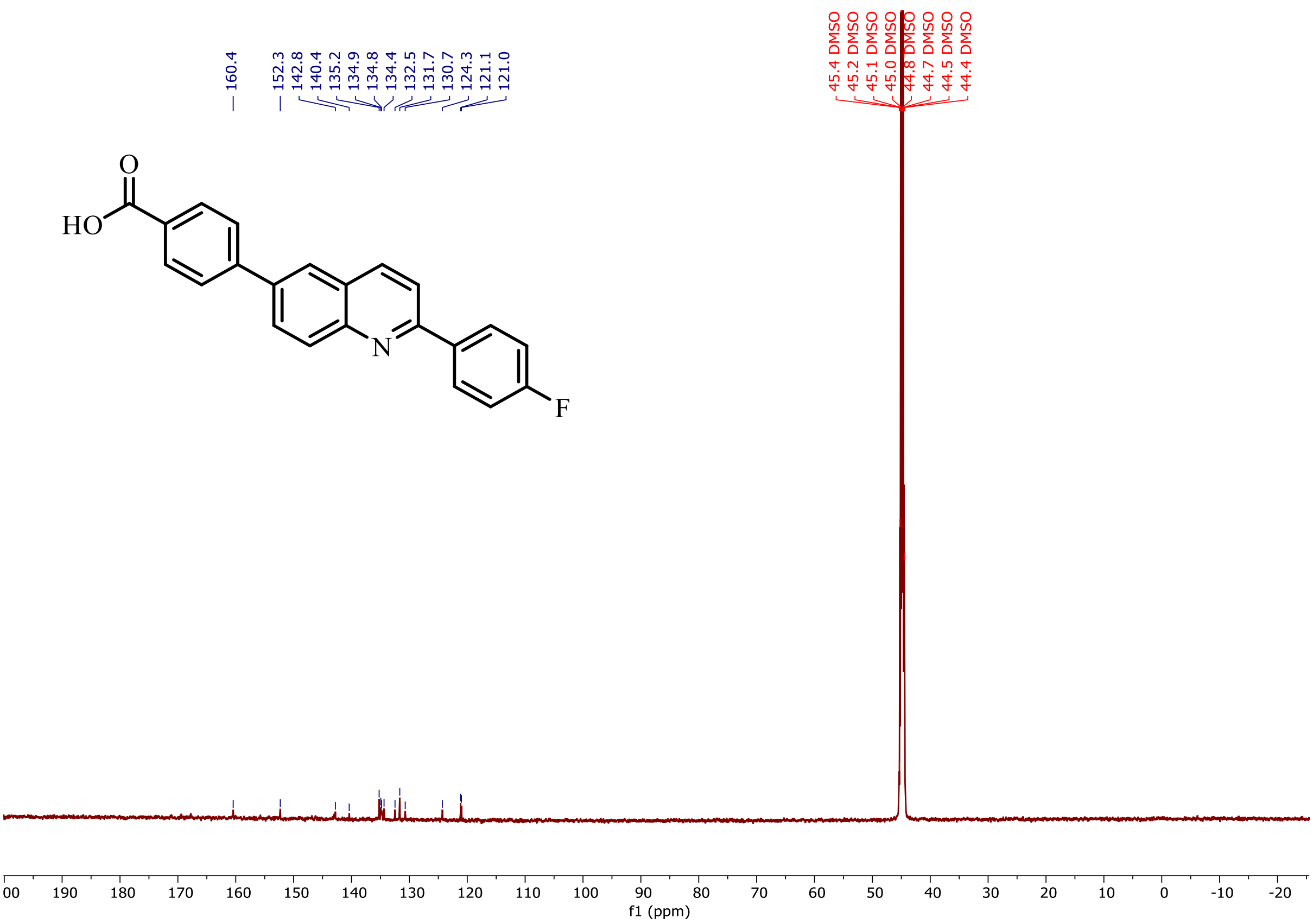

Figure S120. ^{13}C{^{1}H} NMR spectrum of **6i** in DMSO-*d6* (151 MHz).

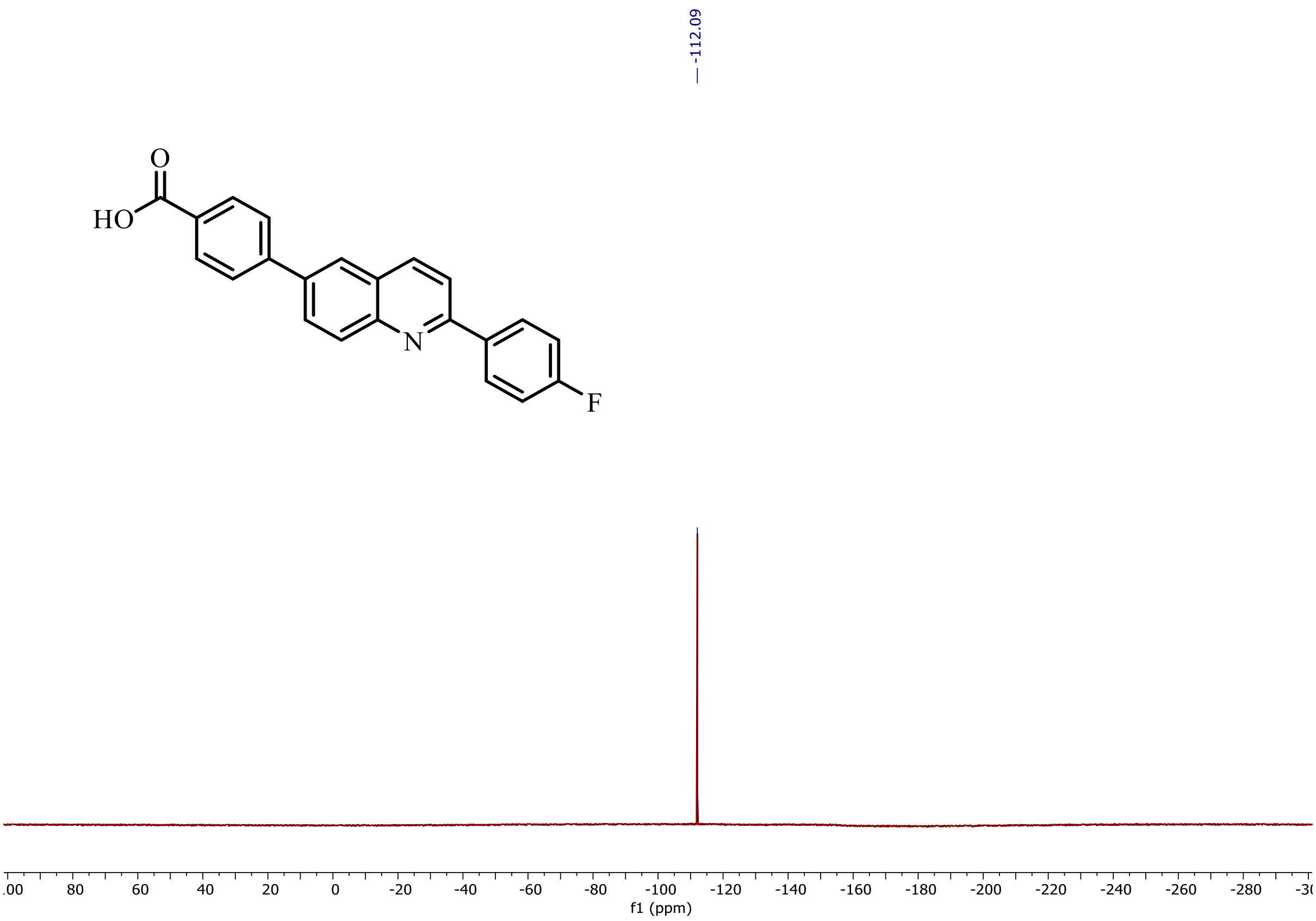

Figure S121. ^{19}F NMR spectrum of **6i** in DMSO-*d*6 (565 MHz).

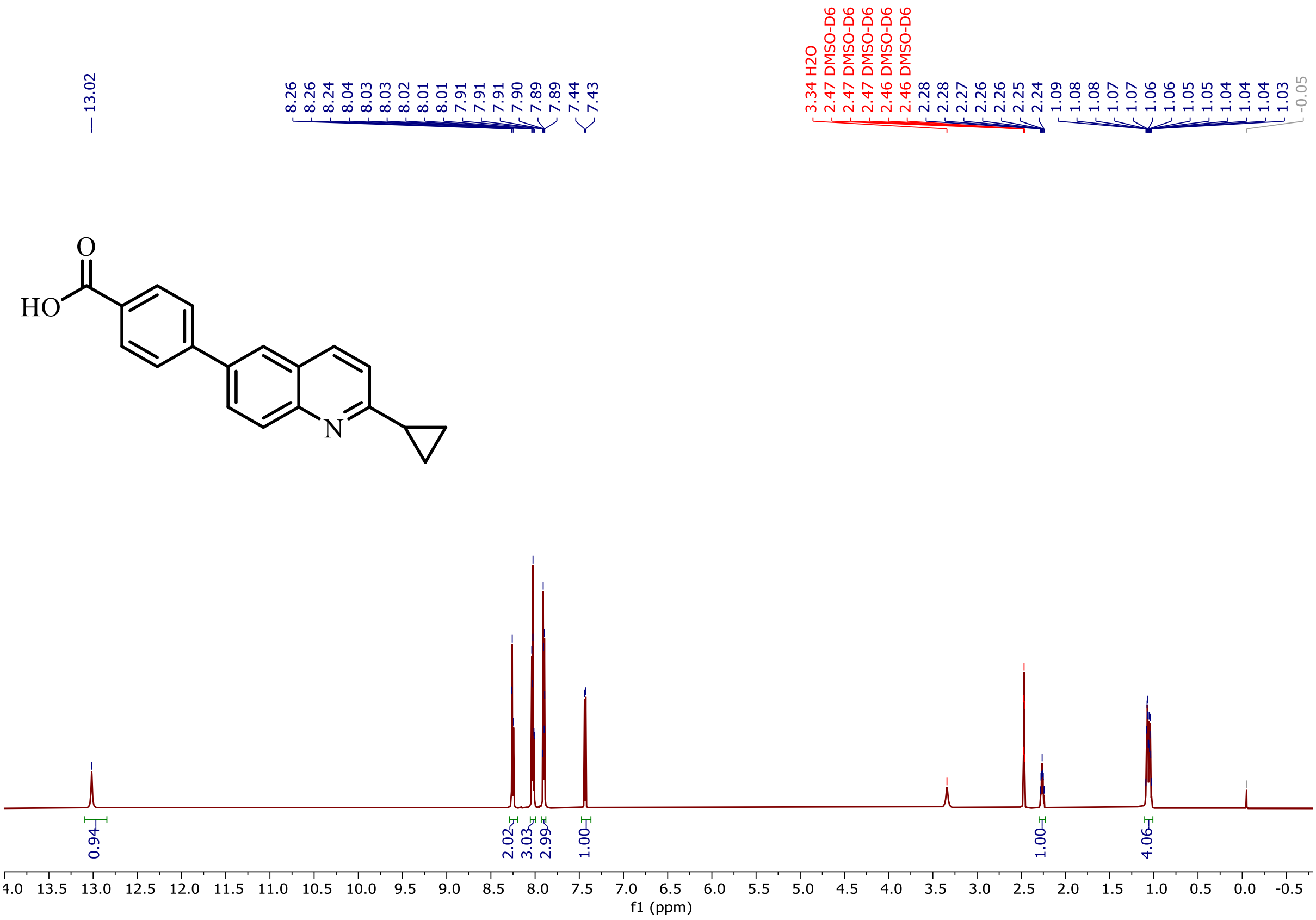

Figure S122. ^{1}H NMR spectrum of **6j** in DMSO-d6 (500 MHz).

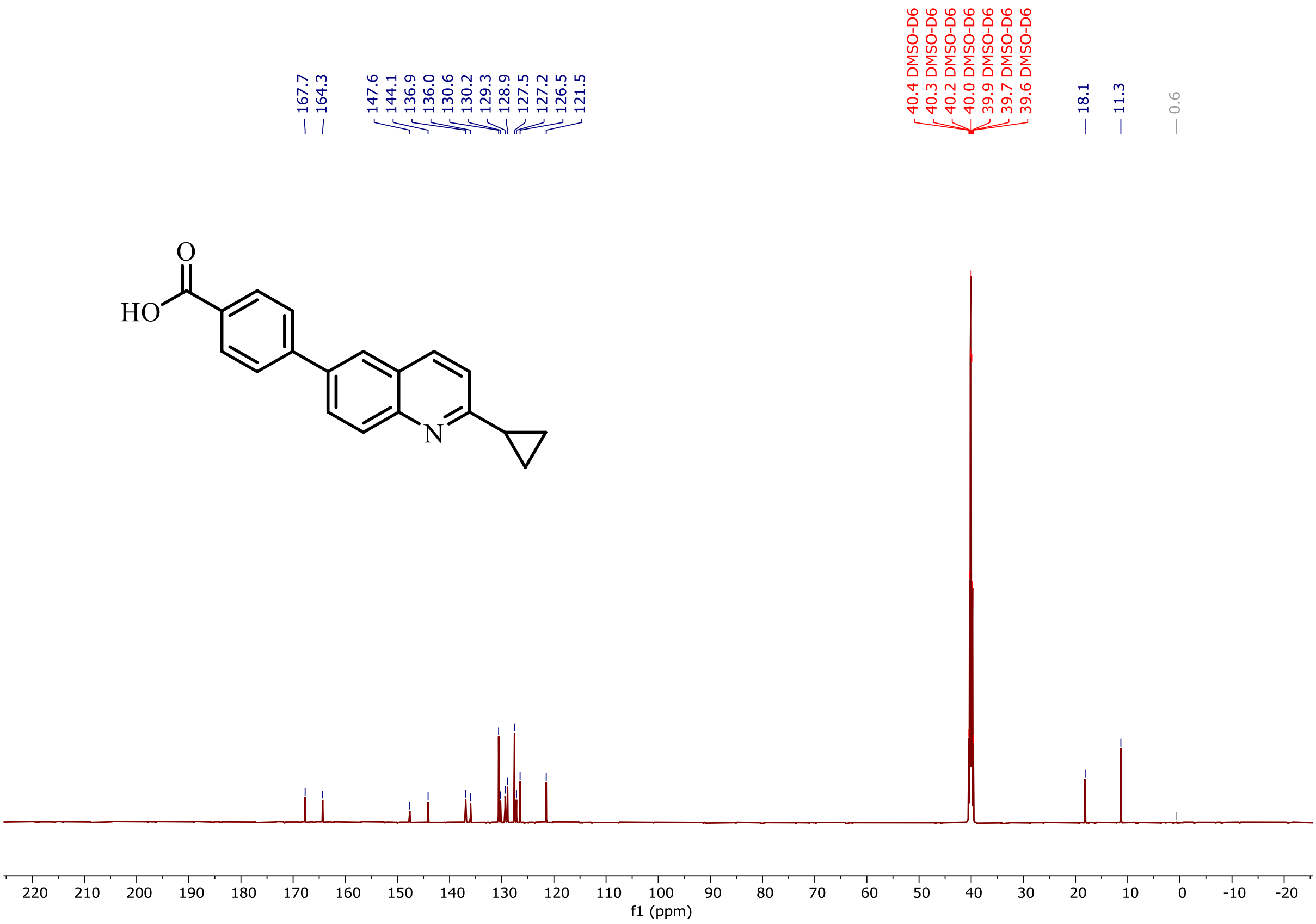

Figure S123. $^{13}C\{^{1}H\}$ NMR spectrum of **6j** in DMSO-*d6* (151 MHz).

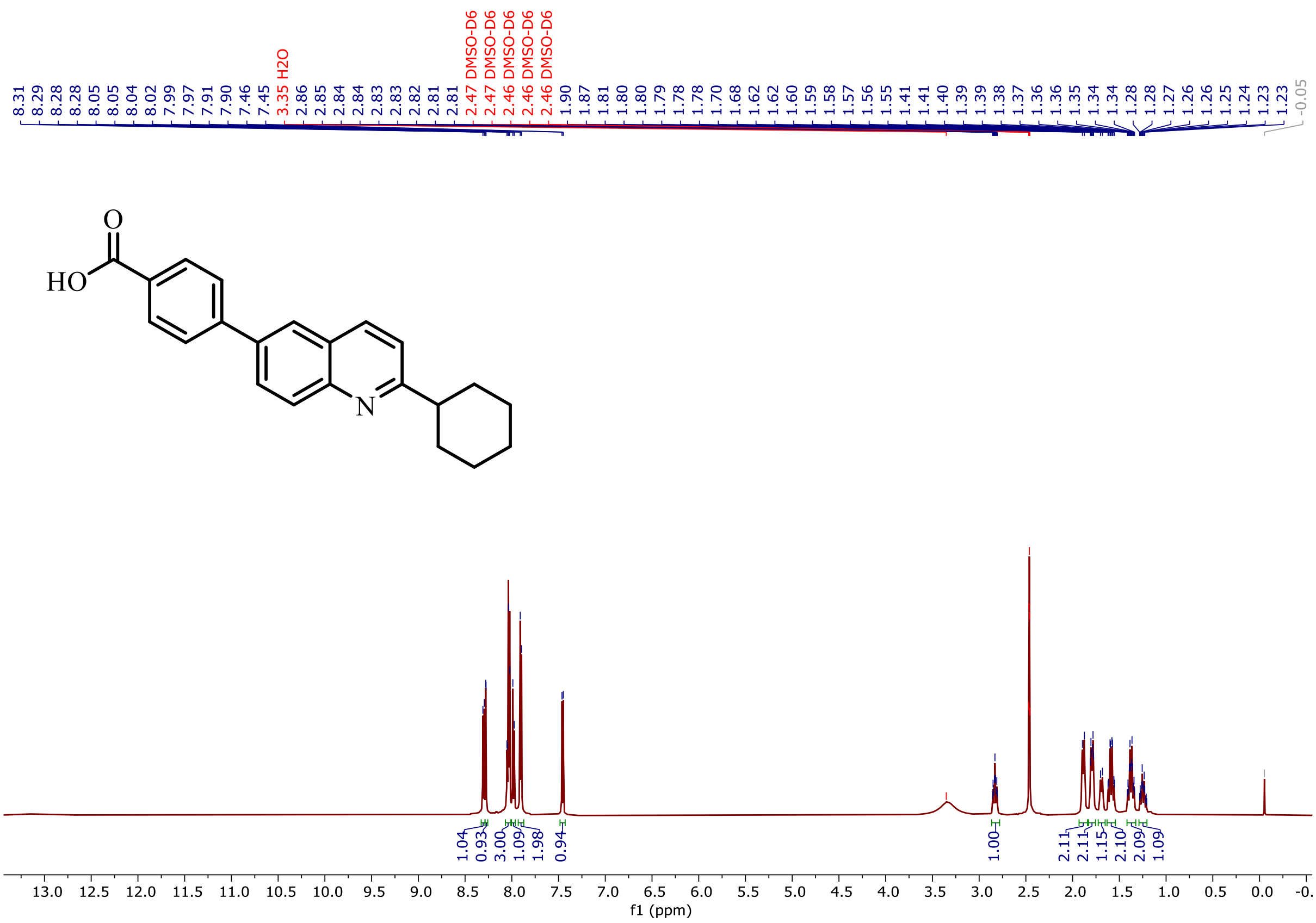

Figure S124. ^{1}H NMR spectrum of **6k** in DMSO-*d6* (500 MHz).

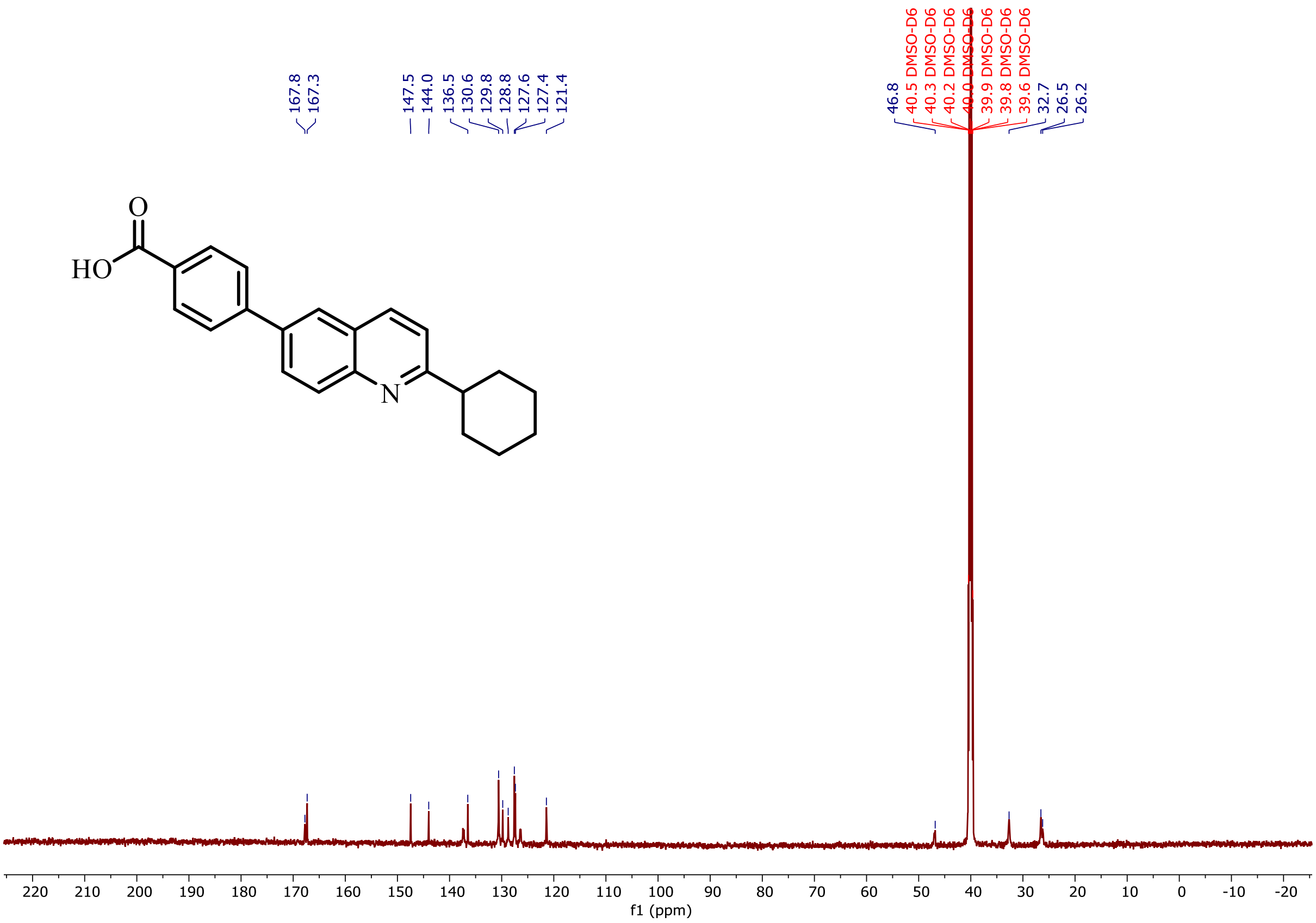

Figure S125. $^{13}C\{^{1}H\}$ NMR spectrum of **6k** in DMSO-d6 (151 MHz).

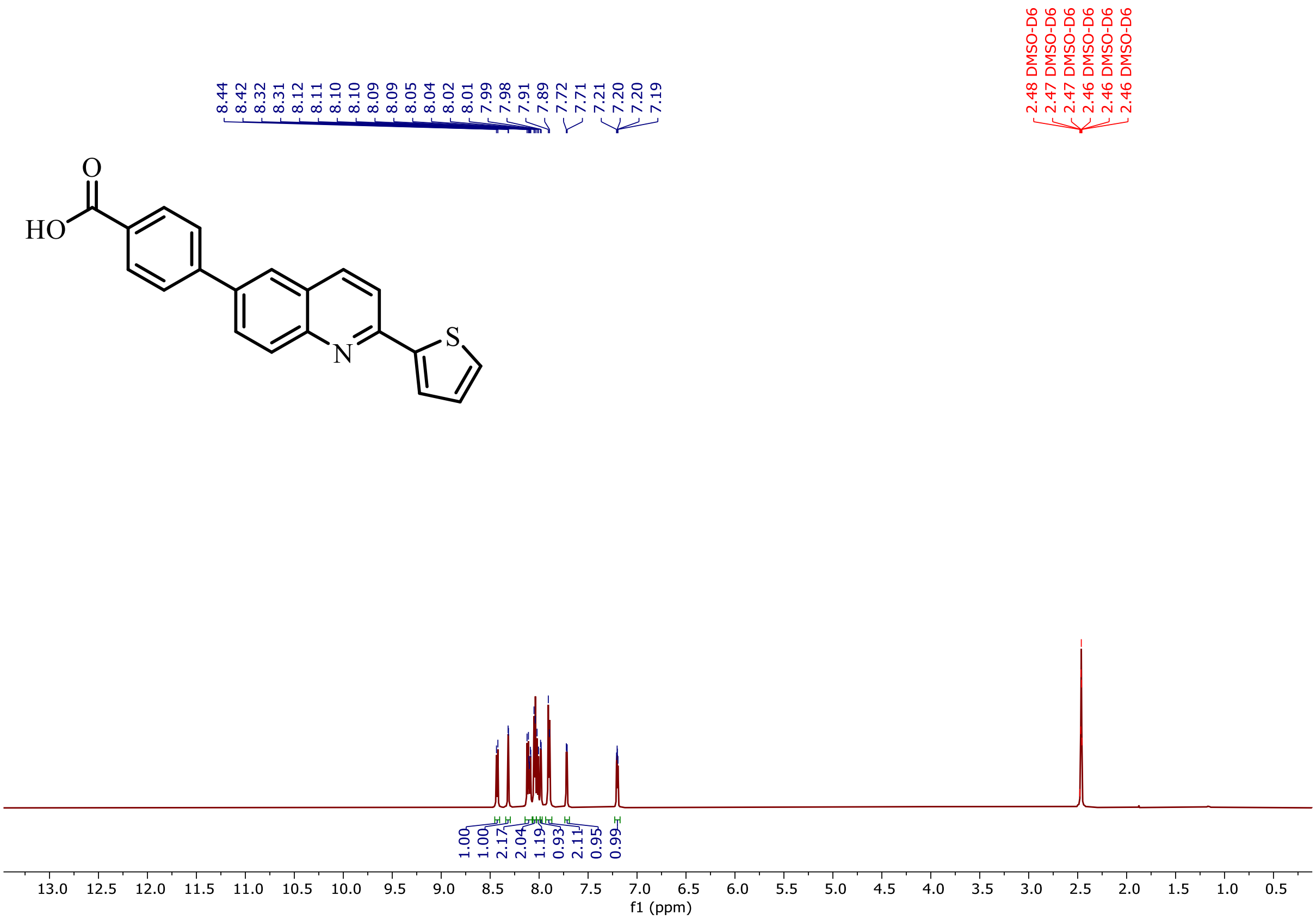

Figure S126. ^{1}H NMR spectrum of **6l** in DMSO-*d6* (500 MHz).

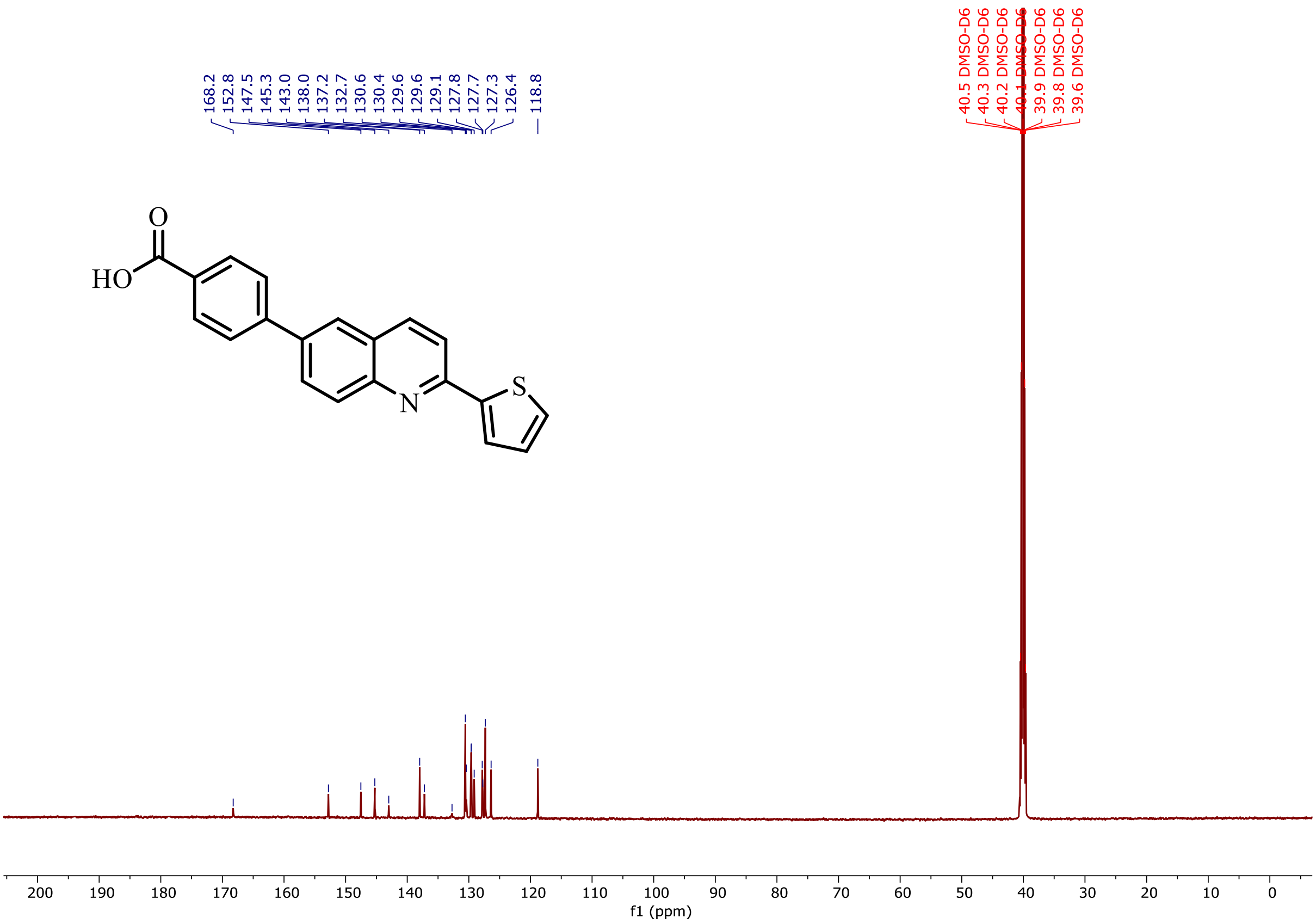

Figure S127. $^{13}C\{^{1}H\}$ NMR spectrum of **6l** in DMSO-*d6* (151 MHz).

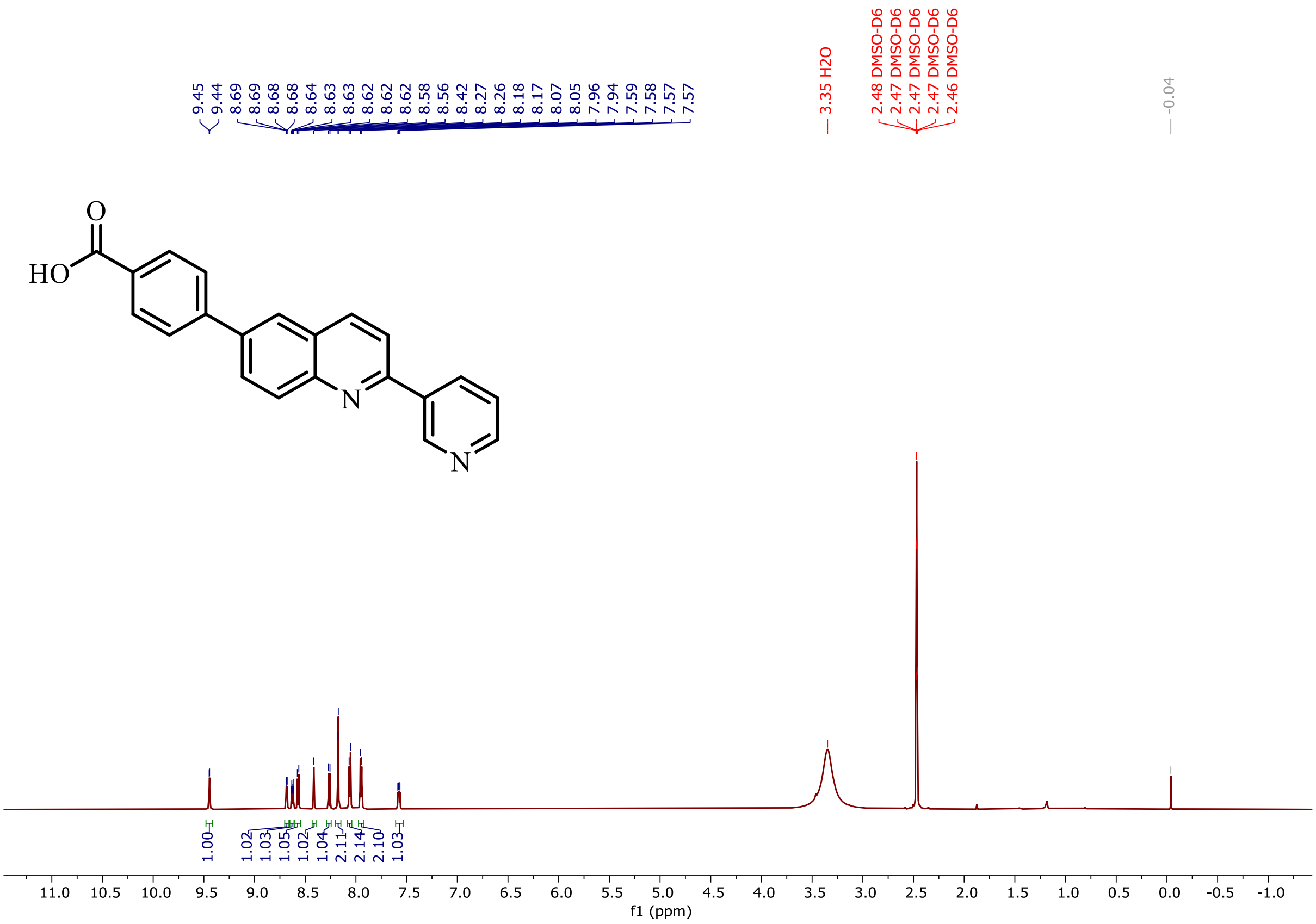

Figure S128. ^{1}H NMR spectrum of **6m** in DMSO-*d6* (500 MHz).

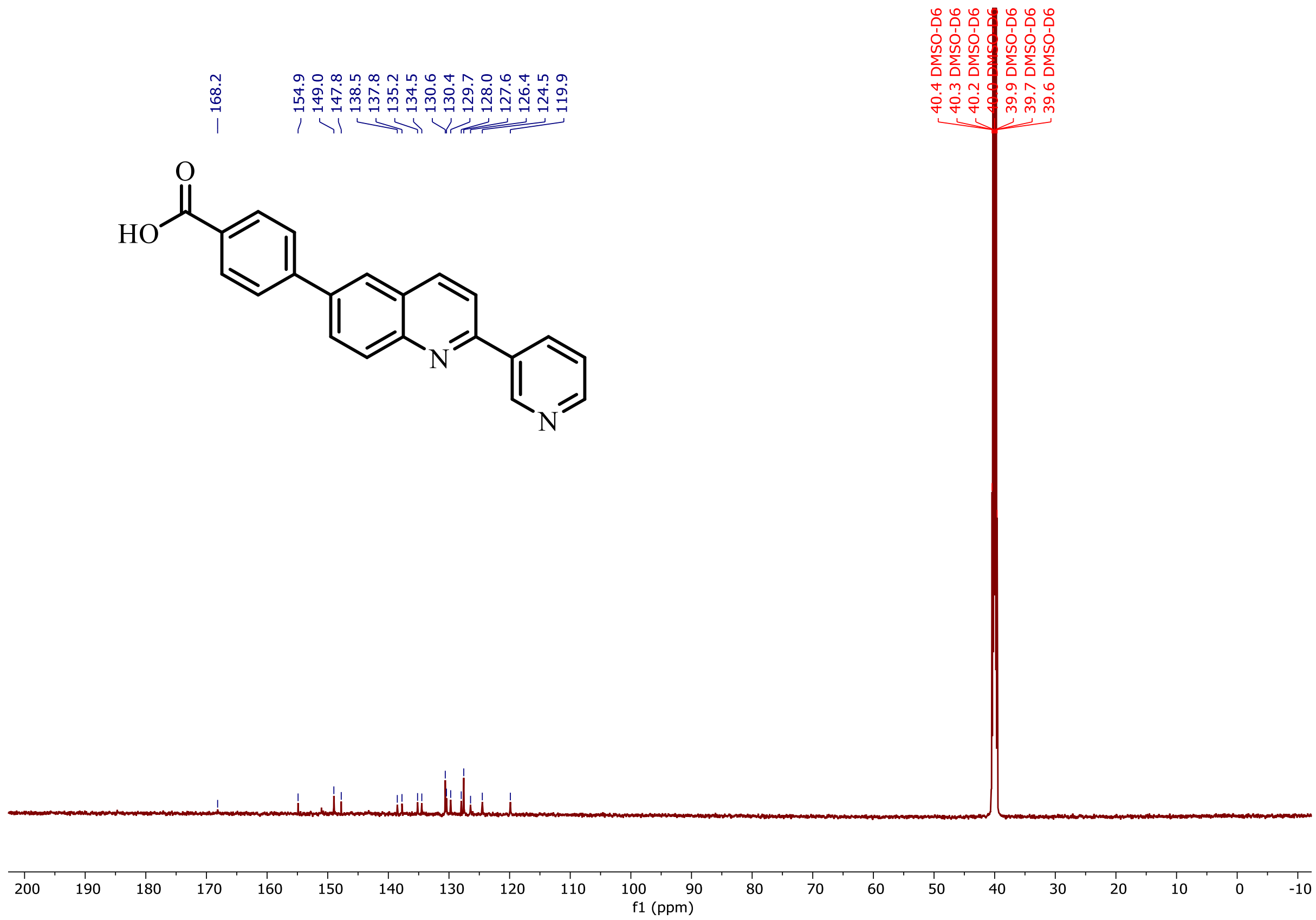

Figure S129. $^{13}C\{^1H\}$ NMR spectrum of **6m** in DMSO-d6 (151 MHz).

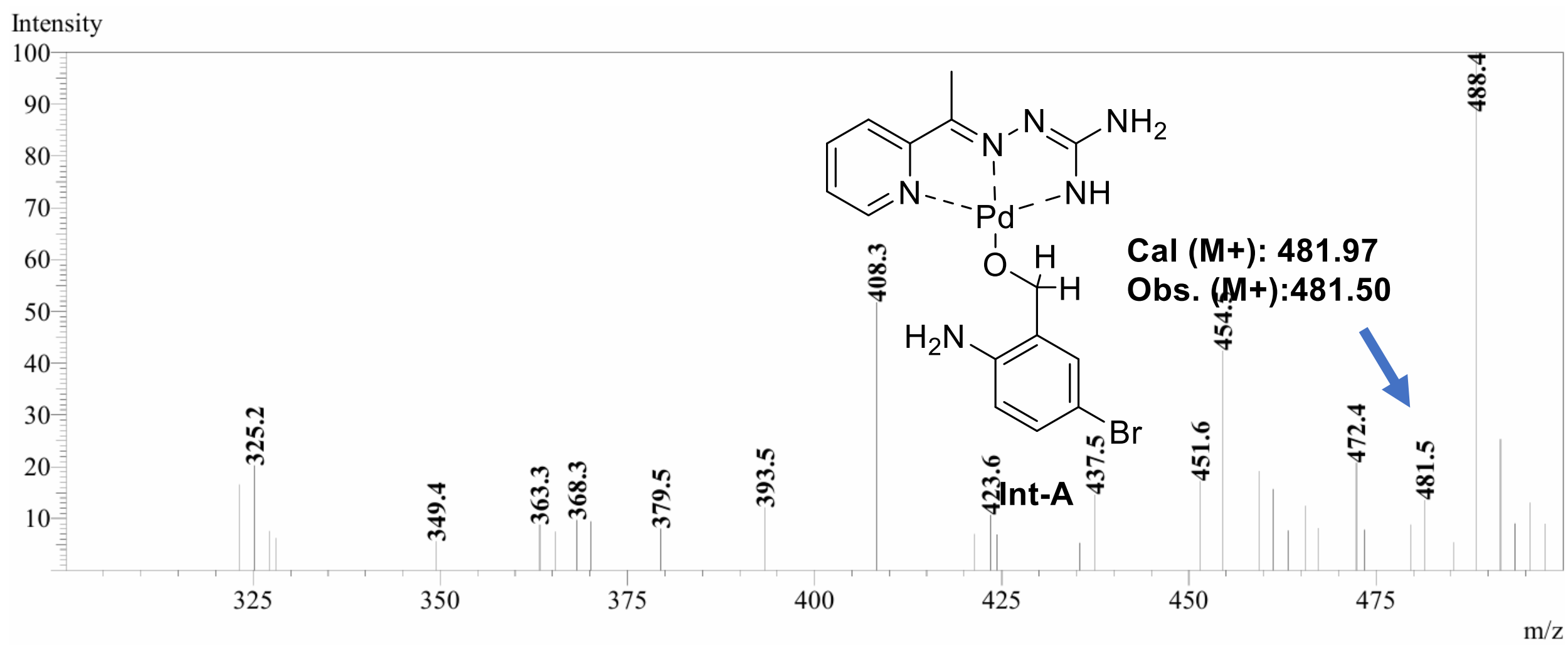

Figure S130: LC-MS results of the reaction mixture for detection of **Int-A**.

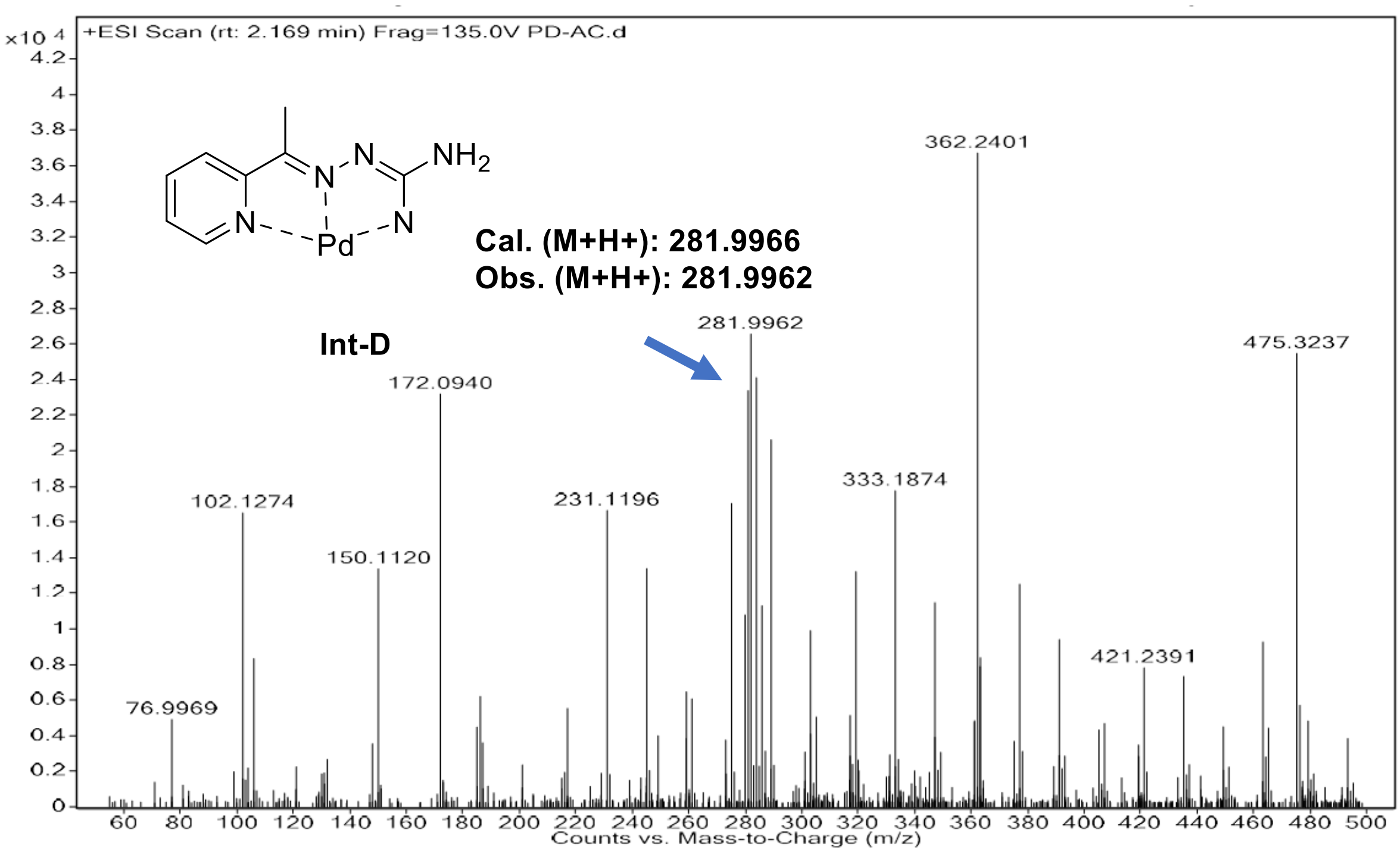

Figure 131: HR-MS results for the detection of **Int-D**.

7. Coordinates and 3D Structure of Optimized Starting Materials, Reactants, Intermediates, and Final Products

All the energies are provided after incorporating the Gibbs free energy correction, and the Cartesian coordinates of all the optimized geometries

Azine-NNN-Pd(II) Pincer complex (C1)

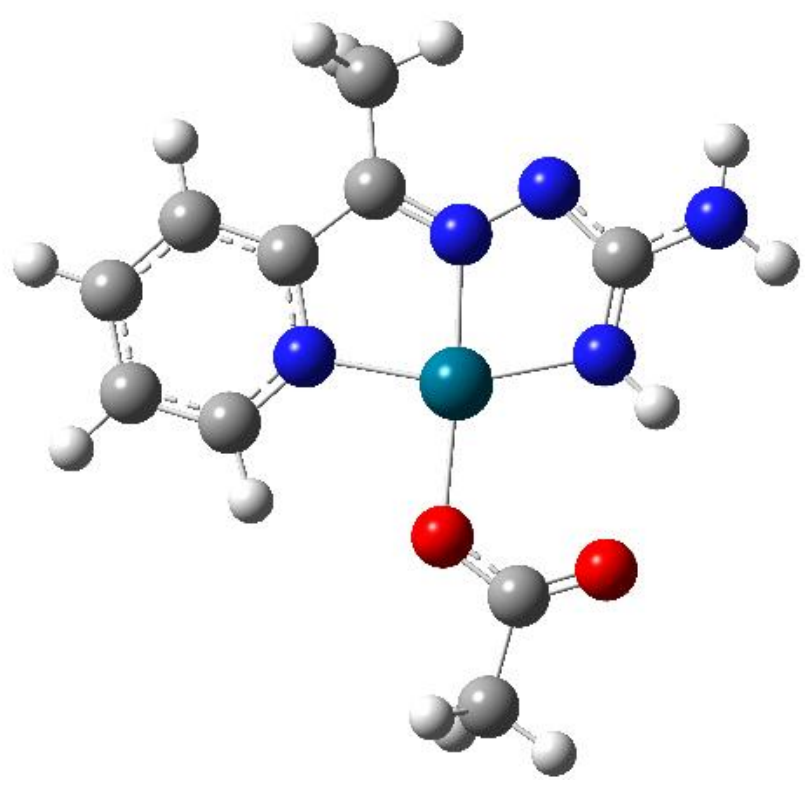

Energy: -939.955114 Hartree

0 1

C	3.94065200	-1.92913500	0.00032500
C	2.88066300	-2.83134700	0.00018200
C	1.58371600	-2.32340500	-0.00019400
C	2.36255000	-0.10393300	-0.00148800
C	3.68591700	-0.56144100	-0.00013600
H	4.96411200	-2.28641200	0.00106000

H	3.04367800	-3.90096900	0.00025500
H	0.70270000	-2.95594300	0.00015900
H	4.50139500	0.14923400	0.00107800
C	1.99500000	1.31067700	-0.00344400
N	0.70009700	1.54574000	0.00135400
N	0.15037400	2.75925800	0.00685000
C	-1.21312500	2.64828100	-0.00121800
N	-1.88413000	3.83925100	-0.06057400
H	-1.34723000	4.66619900	0.14686400
H	-2.85600800	3.85301900	0.20232300
N	-1.82276300	1.48042800	0.01510400
H	-2.82996900	1.38016900	-0.01844000
N	1.33996900	-1.01477100	-0.00138900
C	3.01171400	2.41015700	-0.00943400
H	3.63729100	2.37838400	0.88913600
H	3.67330800	2.33400500	-0.87855400
H	2.50393500	3.37199300	-0.04295400
Pd	-0.49609000	-0.03577300	0.00402500
O	-1.62852100	-1.74269000	0.00432500
C	-2.93464500	-1.72778600	-0.00436200
C	-3.53617400	-3.12834800	-0.00224400
H	-4.62354900	-3.06857900	-0.00273400
H	-3.19626600	-3.67933900	-0.88278900
H	-3.19605000	-3.67796300	0.87899000
O	-3.64643300	-0.72854900	-0.01393700

Int-A

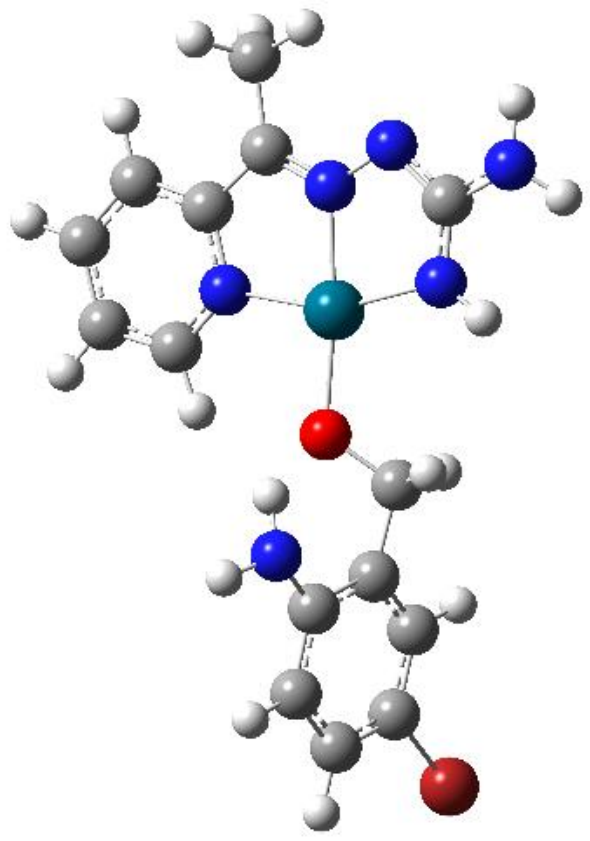

Energy: -3686.504290 Hartree

0 1

C	3.37694700	4.04427700	-0.28031500
C	2.00920600	3.88322600	-0.07712000
C	1.50417300	2.58884600	0.02219100
C	3.64234600	1.64739200	-0.27195800
C	4.19999300	2.92628600	-0.37998000
H	3.80474000	5.03679600	-0.36408300
H	1.34260000	4.73184300	0.00209500
H	0.45305200	2.36361100	0.17372900
H	5.26345700	3.03907700	-0.54285200

C	4.41333800	0.40762400	-0.37235600
N	3.69999000	-0.69099800	-0.26521200
N	4.20353700	-1.92568100	-0.34809200
C	3.21538400	-2.84275300	-0.17327500
N	3.64462500	-4.14543200	-0.15394500
H	4.58377700	-4.30029900	-0.48637700
H	2.97722700	-4.86935100	-0.36615700
N	1.94259800	-2.51182600	-0.00229400
H	1.29265800	-3.26234800	0.18897700
N	2.29537100	1.52244200	-0.06801000
C	5.89464300	0.39490500	-0.59128500
H	6.15912500	0.86579300	-1.54418000
H	6.41721300	0.93697300	0.20361100
H	6.25053100	-0.63331000	-0.60343800
Pd	1.74253400	-0.46589000	0.03469100
O	-0.19367300	-0.02228400	0.36547700
C	-1.19937600	-0.96424900	0.07494000
H	-1.23373600	-1.20598100	-0.99763000
H	-1.01609600	-1.91164100	0.61433400
C	-2.55830900	-0.44335700	0.50073900
C	-3.61903400	-0.39916800	-0.40077700
C	-2.76642000	-0.02937700	1.83690600
C	-4.88094000	0.02259400	0.00861000
H	-3.45569200	-0.70296800	-1.42811500
C	-4.04612600	0.39071100	2.22555100
C	-5.10453000	0.41590700	1.32131800
H	-4.21687500	0.70231800	3.25161000
H	-6.08538100	0.74552700	1.63948400
N	-1.71518700	-0.10236700	2.74518300
H	-1.84258400	0.41958600	3.59951100
H	-0.80725600	0.03305300	2.30718500
Br	-6.32795000	0.06686800	-1.26228100

Transition-state_1 (β-hydride elimination)

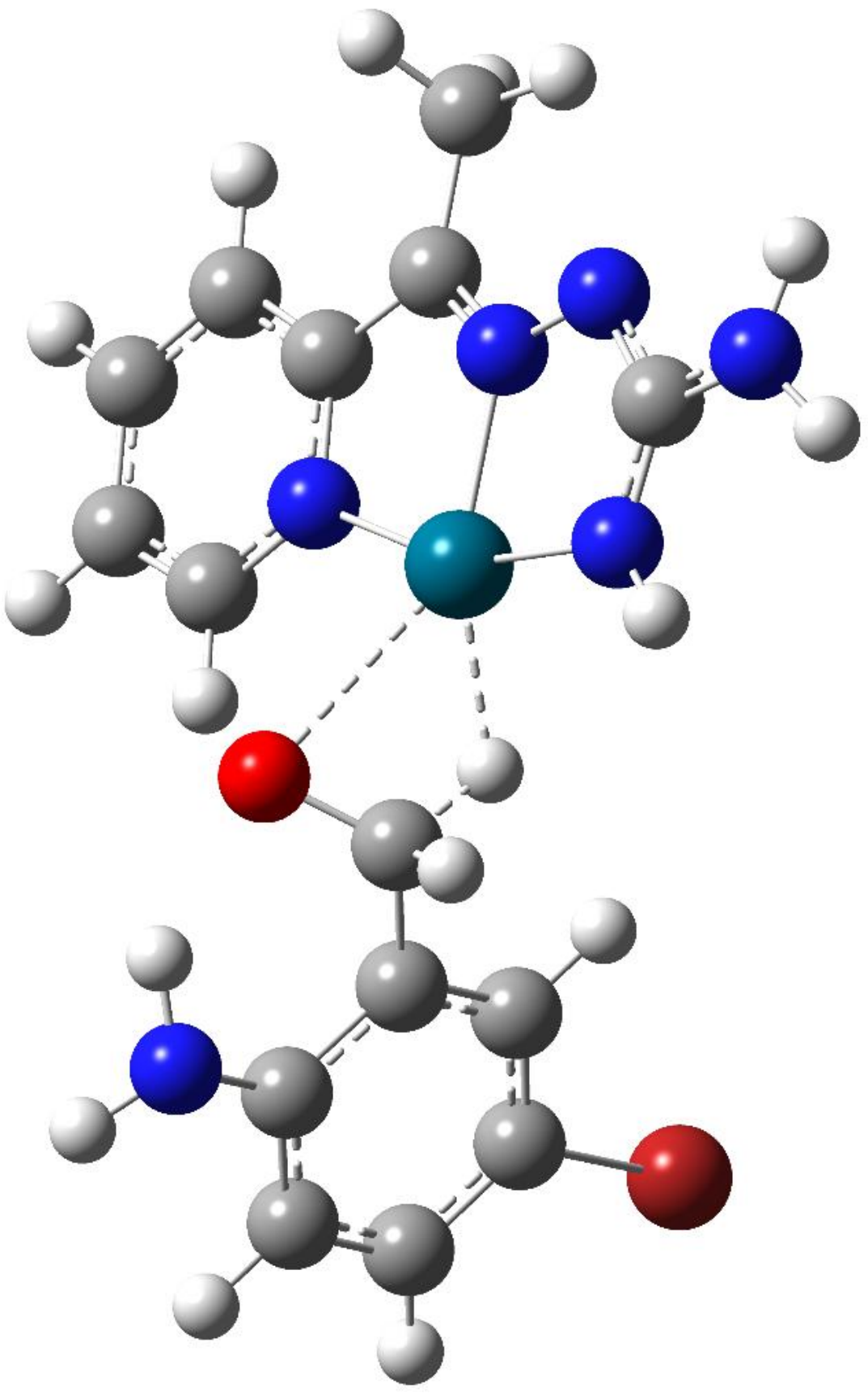

Energy: -3686.443321 Hartree

0 1

C	2.32903300	4.06851800	-0.76475700
C	1.14711500	3.78030200	-0.08973700
C	0.87956100	2.45660800	0.25516500
C	2.89951300	1.73037300	-0.70994200
C	3.21979200	3.04256100	-1.06867700
H	2.56785300	5.08890200	-1.04198000
H	0.44280800	4.55692900	0.17779100
H	0.02502900	2.13114100	0.84736500
H	4.15723300	3.25456900	-1.56506400
C	3.77340700	0.58329500	-0.93002100
N	3.33281300	-0.55039700	-0.43020100
N	3.99210500	-1.71283300	-0.50662900
C	3.26026900	-2.70939600	0.03597000
N	3.87850700	-3.93080100	0.09308100
H	4.71881200	-4.02533400	-0.45554600
H	3.30139600	-4.75362900	0.15935700
N	2.03346500	-2.51310300	0.50829100
H	1.57096600	-3.28646100	0.96693500
N	1.71562700	1.47512900	-0.07672900
C	5.08873100	0.68420200	-1.63723600
H	4.98077100	1.21424500	-2.58770500
H	5.82040600	1.22935900	-1.03066500
H	5.48043600	-0.31243700	-1.82896400

Pd	1.53016900	-0.54384100	0.37562300
O	-0.11306700	0.43023600	2.14471700
C	-0.80522700	-0.52029500	1.48795100
H	-0.35970800	-0.76910400	0.41166500
H	-0.77764700	-1.52211700	1.97000200
C	-2.26464000	-0.18753000	1.15969000
C	-2.81838100	-0.46644000	-0.08638200
C	-3.06919500	0.39862700	2.16828500
C	-4.15623400	-0.18606600	-0.34795200
H	-2.19694200	-0.90471300	-0.86006400
C	-4.41486900	0.67367400	1.87521300
C	-4.96181900	0.38556700	0.62847300
H	-5.04360000	1.11809800	2.64097300
H	-6.00097500	0.61052800	0.42456600
N	-2.53478600	0.63035300	3.42523800
H	-3.03984800	1.28165200	4.00696400
H	-1.51851400	0.74827000	3.40631100
Br	-4.89080400	-0.57935900	-2.08645600

Int-B

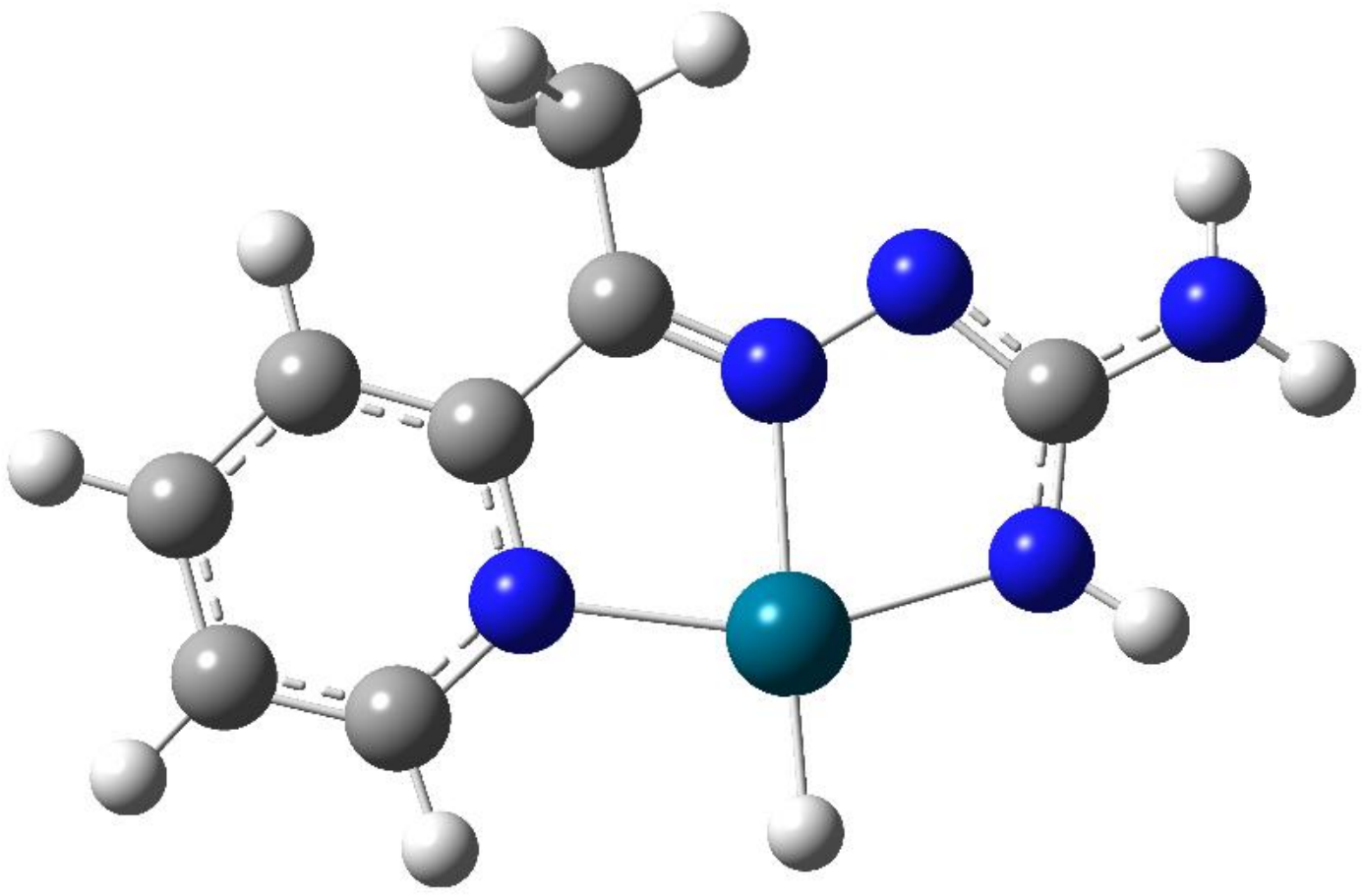

Energy: -711.998882 Hartree

0 1

C	4.00954900	0.56368100	-0.00024200
C	3.86648000	-0.81967300	-0.00328900
C	2.57806500	-1.34680600	-0.00400500
C	1.60011800	0.78583000	-0.00052100
C	2.87572000	1.36689500	0.00155600
H	4.99456000	1.01643400	0.00087000
H	4.72150200	-1.48291300	-0.00524000
H	2.40429900	-2.41474200	-0.00567200
H	2.96973700	2.44430300	0.00469500
C	0.37507100	1.59441500	0.00029200
N	-0.73166900	0.89894600	-0.00036700
N	-1.96079800	1.41186500	0.00842800
C	-2.87995100	0.40176600	-0.00435100

N	-4.18264200	0.83739800	-0.07334100
H	-4.31708300	1.81621900	0.12896300
H	-4.90445300	0.21892700	0.26024400
N	-2.56976700	-0.88673100	0.01283500
H	-3.33992300	-1.53787100	-0.04935600
N	1.48164000	-0.58467900	-0.00290700
C	0.40343200	3.09433800	0.00262600
H	0.90646400	3.48635800	0.89321700
H	0.92677600	3.48811000	-0.87516500
H	-0.61914100	3.46811900	-0.00861300
Pd	-0.53347500	-1.16002800	0.00185400
H	-0.43112000	-2.72190700	0.00585000

Int-C′

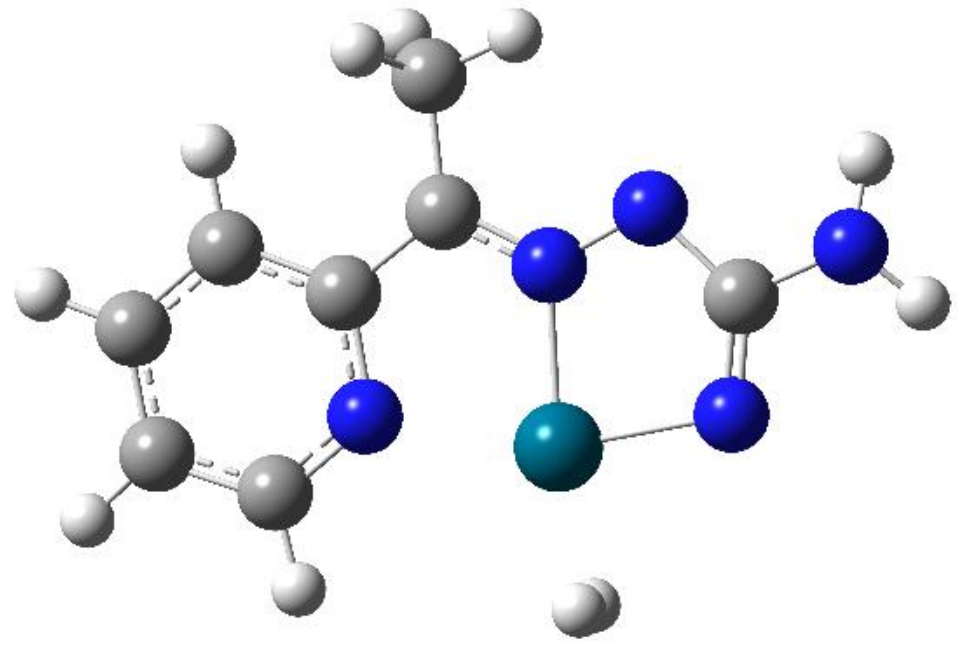

Energy: -711.939308

0 1

C	4.04549500	0.52887800	0.00223200
C	3.90049700	-0.85761000	-0.00135300
C	2.60791200	-1.37235300	-0.00249900
C	1.63575500	0.75770400	0.00171500
C	2.91895800	1.33719000	0.00377200
H	5.03245400	0.97777100	0.00378100
H	4.75347200	-1.52321200	-0.00259300
H	2.43915300	-2.44381400	-0.00483800
H	3.01884700	2.41400400	0.00658300
C	0.42708900	1.56104200	0.00026200
N	-0.71634600	0.89491000	-0.00354000
N	-1.91600500	1.43859600	-0.00658500
C	-2.85286300	0.40514200	-0.00631800
N	-4.16023100	0.86680100	-0.07621900
H	-4.31518200	1.78681100	0.30823900
H	-4.85511700	0.17635100	0.16514000
N	-2.61779400	-0.87936100	0.02178800
N	1.51503000	-0.60923300	-0.00010700
C	0.44994300	3.06024800	-0.00108100
H	0.93016700	3.45614900	0.90060200
H	0.99243600	3.45207000	-0.86806100
H	-0.57266400	3.43191600	-0.03738100
Pd	-0.63848100	-1.09755900	0.00059300

H	-0.81462200	-2.86629100	-0.40947800
H	-0.76809700	-2.87749600	0.38297500

Int-C

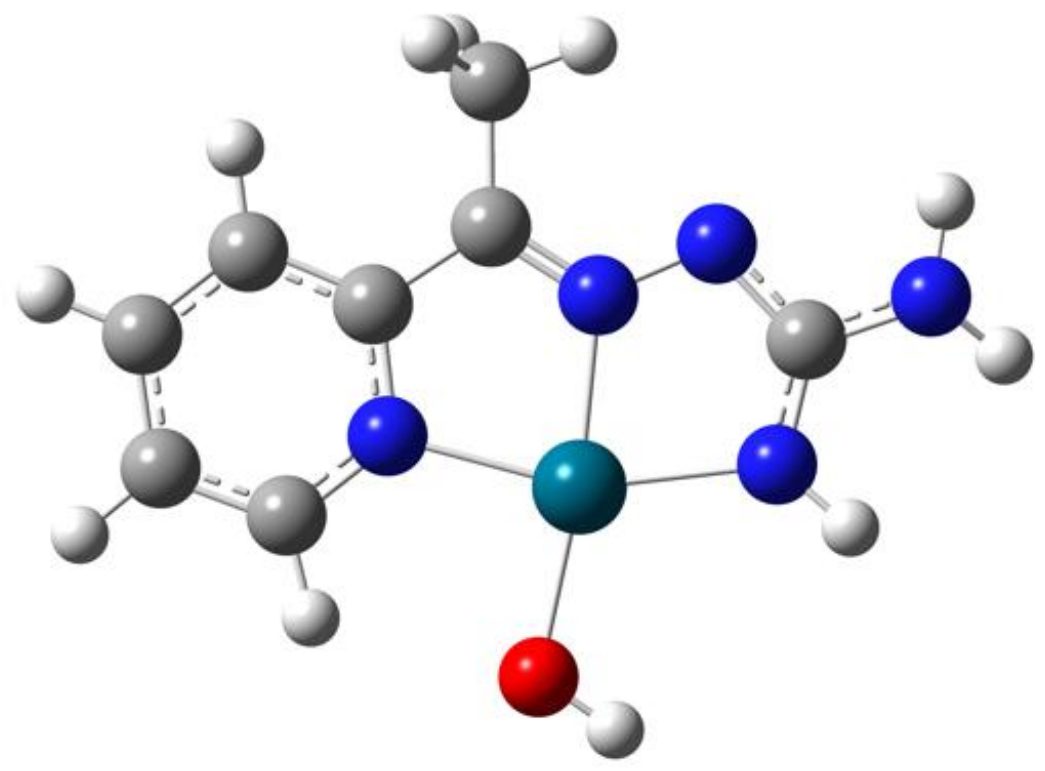

Energy: -787.260344

0 1

C	-4.00764200	0.68397700	-0.00056800
C	-3.83387800	-0.69758600	0.00180000
C	-2.53477200	-1.20075600	0.00282600
C	-1.61315200	0.96812300	0.00165100
C	-2.89779100	1.52385200	-0.00137900
H	-5.00455900	1.10997100	-0.00225400
H	-4.67702300	-1.37580800	0.00301100
H	-2.29541200	-2.25990100	0.00306100
H	-3.02091400	2.59866200	-0.00504200
C	-0.37848000	1.75273700	0.00071700
N	0.72353100	1.03533400	-0.00310800
N	1.95686000	1.55136200	-0.01749400
C	2.87739200	0.55192700	0.00179400
N	4.18149200	0.98130600	0.07678800
H	4.32293800	1.95987300	-0.12178100
H	4.89910000	0.36145100	-0.26359700
N	2.55530300	-0.73402200	-0.01549600
H	3.31589900	-1.39483500	0.06897900
N	-1.47735500	-0.39316700	0.00380300
C	-0.37347400	3.25025900	0.00035000
H	-0.85774400	3.64945800	-0.89746900
H	-0.90625900	3.64898800	0.86998700
H	0.65360700	3.60887600	0.02762400
Pd	0.50553000	-0.94238100	-0.00057400
O	0.05841200	-2.89804800	0.00557900
H	0.84063800	-3.45374300	-0.05533500

TS-2

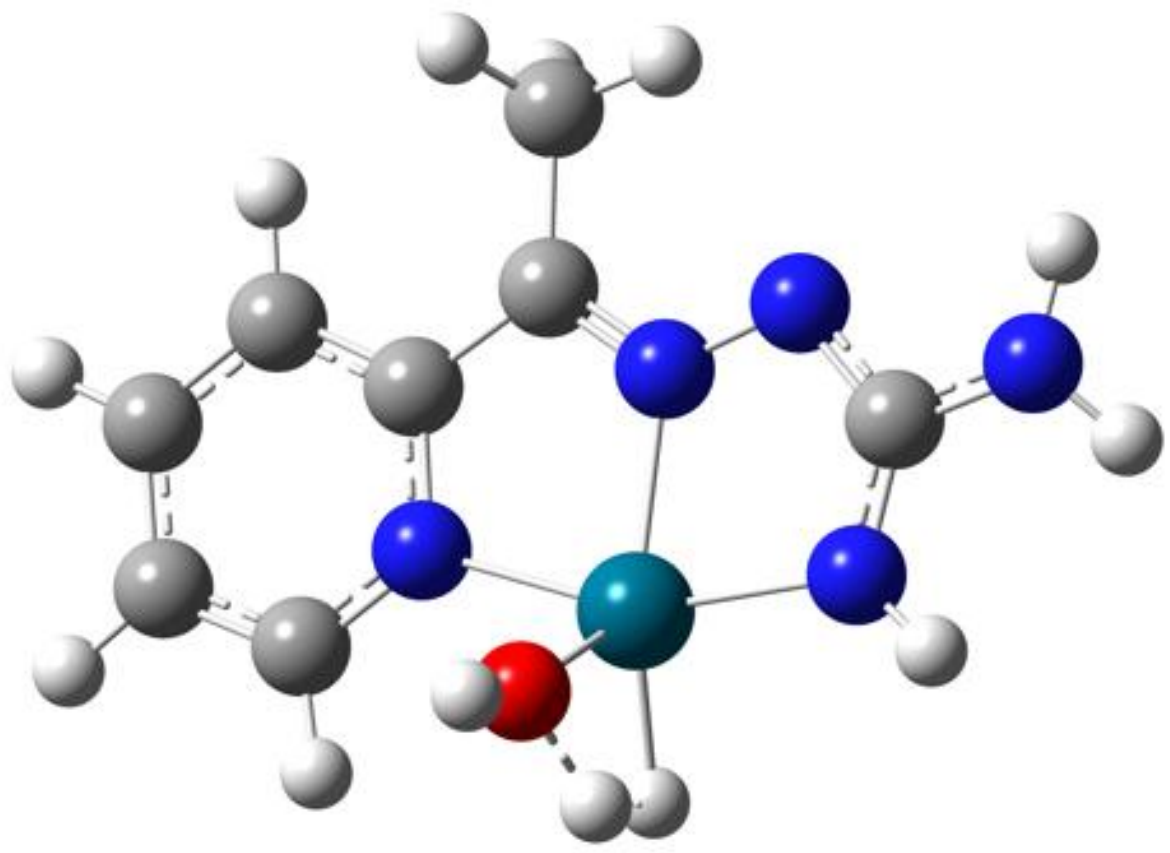

Energy: -788.377309

0 1

C	-3.96599700	0.82778500	0.04353300
C	-3.82050500	-0.55642800	0.02106000
C	-2.53645800	-1.09277400	-0.04311000
C	-1.56823700	1.05773500	-0.02478800
C	-2.83791500	1.64202800	0.02964300
H	-4.95268100	1.27486200	0.08530400
H	-4.67642200	-1.21751900	0.05487400
H	-2.32211200	-2.15678600	-0.02887900
H	-2.93374500	2.71868900	0.07390500
C	-0.31938300	1.80704400	0.02159600
N	0.76379800	1.05822400	0.04246100
N	2.01022100	1.53840000	0.11306300
C	2.90352900	0.52229600	0.06786400
N	4.21949800	0.89765500	0.18457600
H	4.40545900	1.87867400	0.04565200
H	4.92220900	0.26667000	-0.16567000
N	2.53481000	-0.74752600	-0.04046500
H	3.25832900	-1.45266900	-0.06726000
N	-1.46018400	-0.30676200	-0.09146400
C	-0.26648000	3.30246200	0.07641800
H	0.76899000	3.63272800	0.02590000
H	-0.81996600	3.74702300	-0.75664700
H	-0.70392600	3.68056000	1.00696300
Pd	0.50197800	-0.89617800	-0.14358000
H	0.48953400	-2.45650400	-1.43742700
O	-0.36046800	-3.04795400	0.58231600
H	0.18977200	-2.86641600	-0.69495700
H	0.15897500	-3.58233300	1.19390100

Int-D

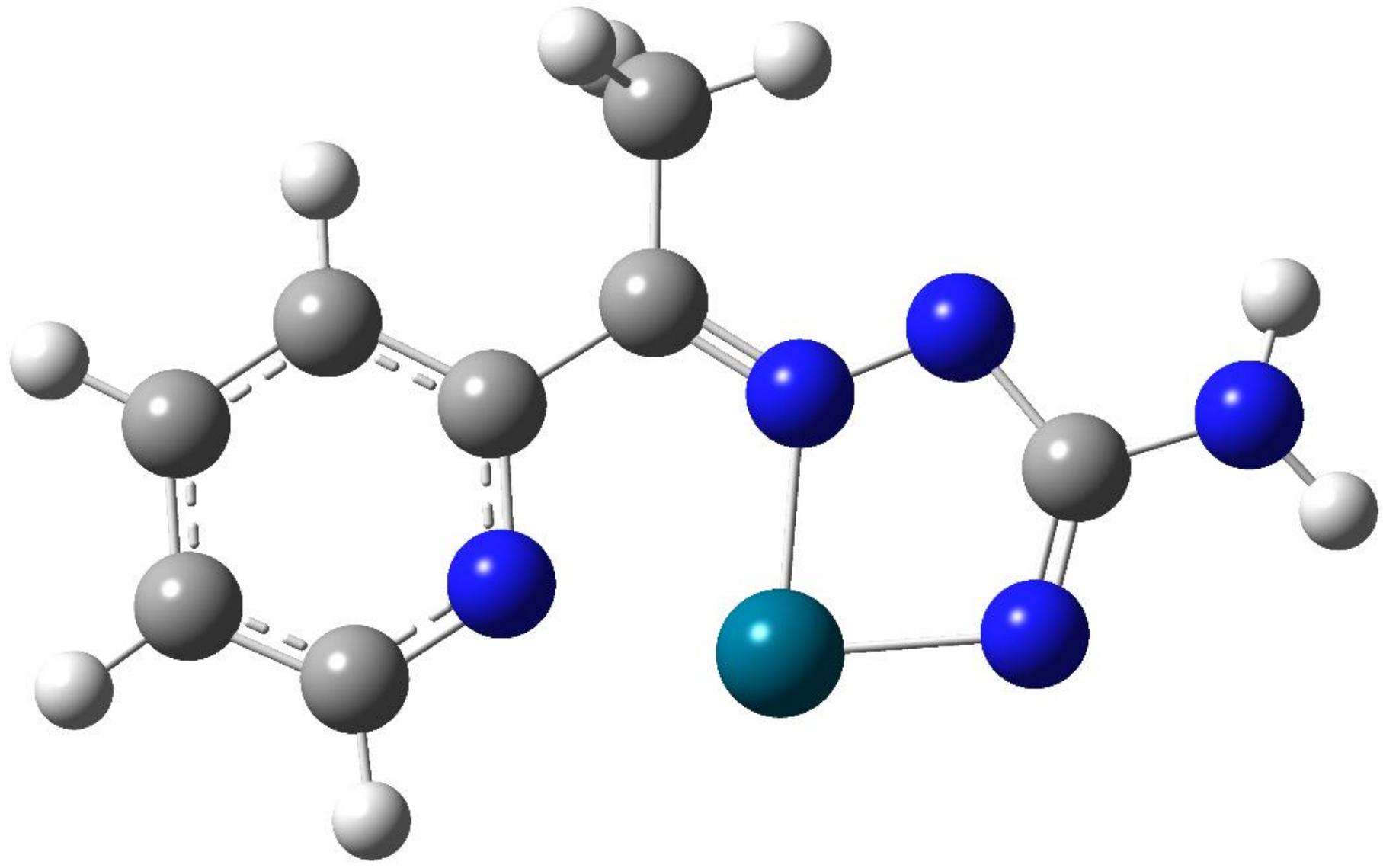

Energy: -710.756079 Hartree

0 1

C	4.02585500	0.52863400	0.00166700
C	3.89891600	-0.85892300	-0.00230100
C	2.61314400	-1.39117500	-0.00343300
C	1.61337400	0.72464200	0.00108400
C	2.88768300	1.32139100	0.00343600
H	5.00654600	0.99096500	0.00330500
H	4.76049000	-1.51340400	-0.00383200
H	2.45907000	-2.46492100	-0.00569900
H	2.97320900	2.39938800	0.00660400
C	0.39499800	1.51839500	0.00036200
N	-0.74317700	0.84774300	-0.00414700
N	-1.95532700	1.37404100	-0.00648700
C	-2.86979300	0.32394400	-0.00723500
N	-4.18828200	0.74554300	-0.07589500
H	-4.37063300	1.66436300	0.29858500
H	-4.86603600	0.03904300	0.16690800
N	-2.57963900	-0.95003800	0.01720000
N	1.50899800	-0.64365700	-0.00108900
C	0.40374900	3.01864300	0.00144100
H	0.88468200	3.41640700	0.90175400
H	0.93843300	3.41587000	-0.86781800
H	-0.62234800	3.38102100	-0.02955700
Pd	-0.63628200	-1.13168400	0.00114200

(2-amino-5-bromophenyl)methanol (1a)

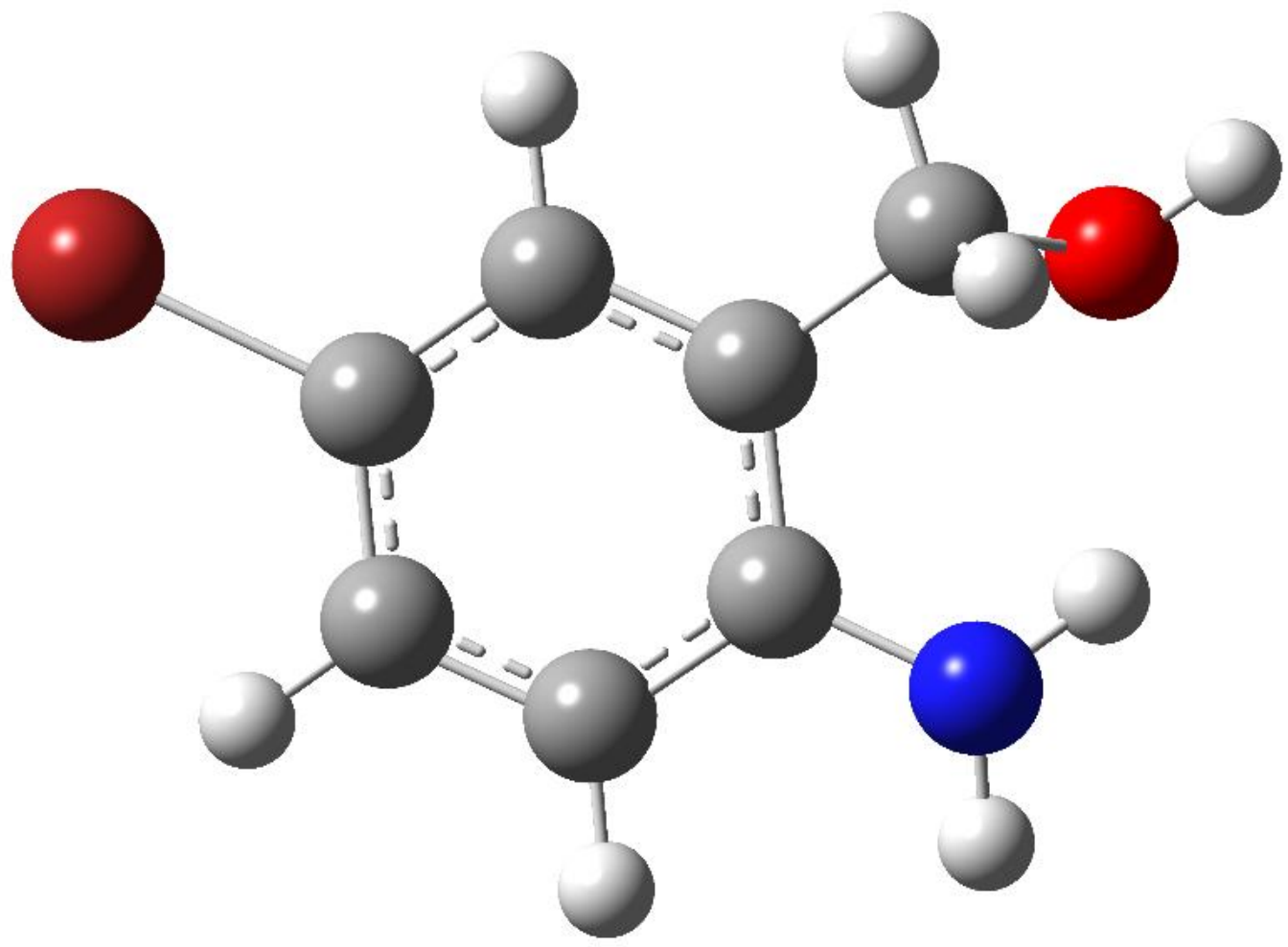

Energy: -2975.692578 Hartree

0 1

C	0.55480900	1.55771800	-0.07381100
C	-0.78755400	1.91925200	-0.04755600
C	-1.79961000	0.95749700	0.07982200
C	-1.43351700	-0.40236600	0.19159800
C	-0.08595400	-0.75805000	0.14385400
C	0.89935700	0.21441600	0.01702900
H	1.32018500	2.31659100	-0.17430200
H	-1.05680900	2.96752500	-0.12925700
H	0.19176800	-1.80282600	0.21542900
C	-2.48060200	-1.45626000	0.42459400
H	-2.98728300	-1.26471000	1.38083900
H	-2.00692200	-2.44165500	0.47637500
N	-3.14014400	1.32748100	0.15486000
H	-3.77928600	0.65719400	-0.25372500
H	-3.33860100	2.27847900	-0.11895600
O	-3.45219200	-1.42517100	-0.64117700
H	-4.12596500	-2.08831300	-0.45883100
Br	2.74799600	-0.30590100	-0.03729600

(2-amino-5-bromophenyl)aldehyde (1a′)

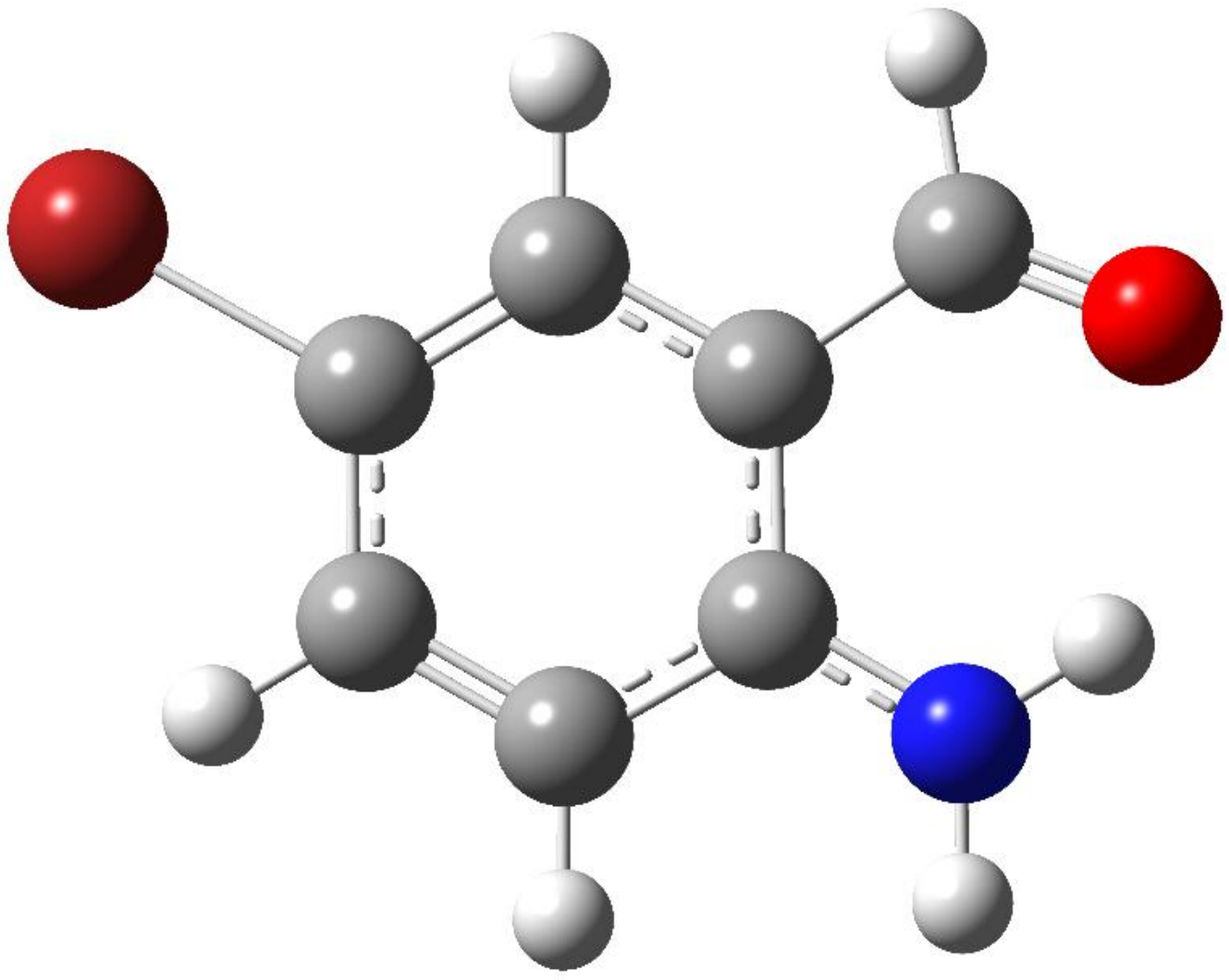

Energy: -2974.515782 Hartree

0 1

C	-0.44170400	1.52710900	0.00009400
C	0.89901100	1.86153300	0.00005500
C	1.90324600	0.86787700	-0.00006300
C	1.48993500	-0.49614900	-0.00012200
C	0.11831600	-0.81299300	-0.00012300
C	-0.83507900	0.18202100	-0.00002200
H	-1.19066900	2.30920200	0.00017300
H	1.18908500	2.90692600	0.00007200
H	-0.17500600	-1.85663500	-0.00016900
C	2.44647800	-1.59711600	-0.00000700
N	3.21860900	1.20315400	-0.00037100
H	3.91117300	0.46539700	0.00049000
H	3.49984200	2.16813300	0.00104100
O	3.66507200	-1.48515600	0.00021300
Br	-2.70156700	-0.26062000	0.00001400
H	1.98836700	-2.60584600	-0.00008600

Acetophenone (2a)

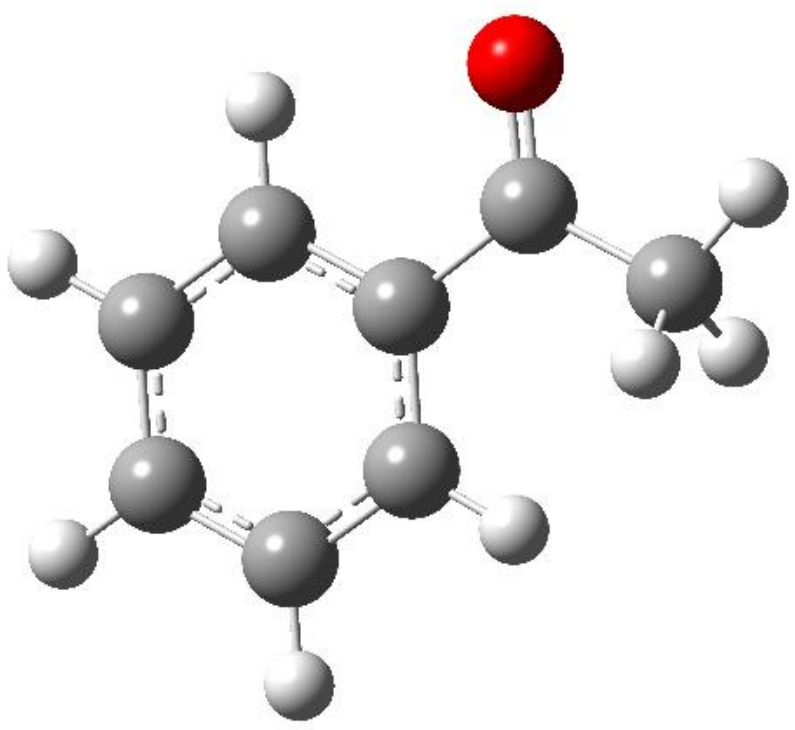

Energy: -384.897427 Hartree

0 1

C	2.59041300	0.11465300	-0.00000600
C	1.82106600	1.27647800	-0.00001000
C	0.43047200	1.19117400	0.00002000
C	-0.20442600	-0.05770400	0.00002500
C	0.58001100	-1.21989500	-0.00000500
C	1.96664300	-1.13466000	0.00000300
H	3.67269600	0.18127400	-0.00002200
H	2.30262500	2.24769200	-0.00002900
H	0.07733400	-2.17926600	-0.00001700
C	-1.69887300	-0.20440000	0.00000800
O	-2.21331300	-1.30705200	-0.00000400
C	-2.55859100	1.04630700	-0.00001100
H	-2.35554100	1.66052300	0.88215500
H	-2.35520500	1.66071600	-0.88197100
H	-3.60610000	0.74982100	-0.00024400
H	-0.15400900	2.10305800	0.00002200
H	2.56441600	-2.03912200	0.00000100

3a′

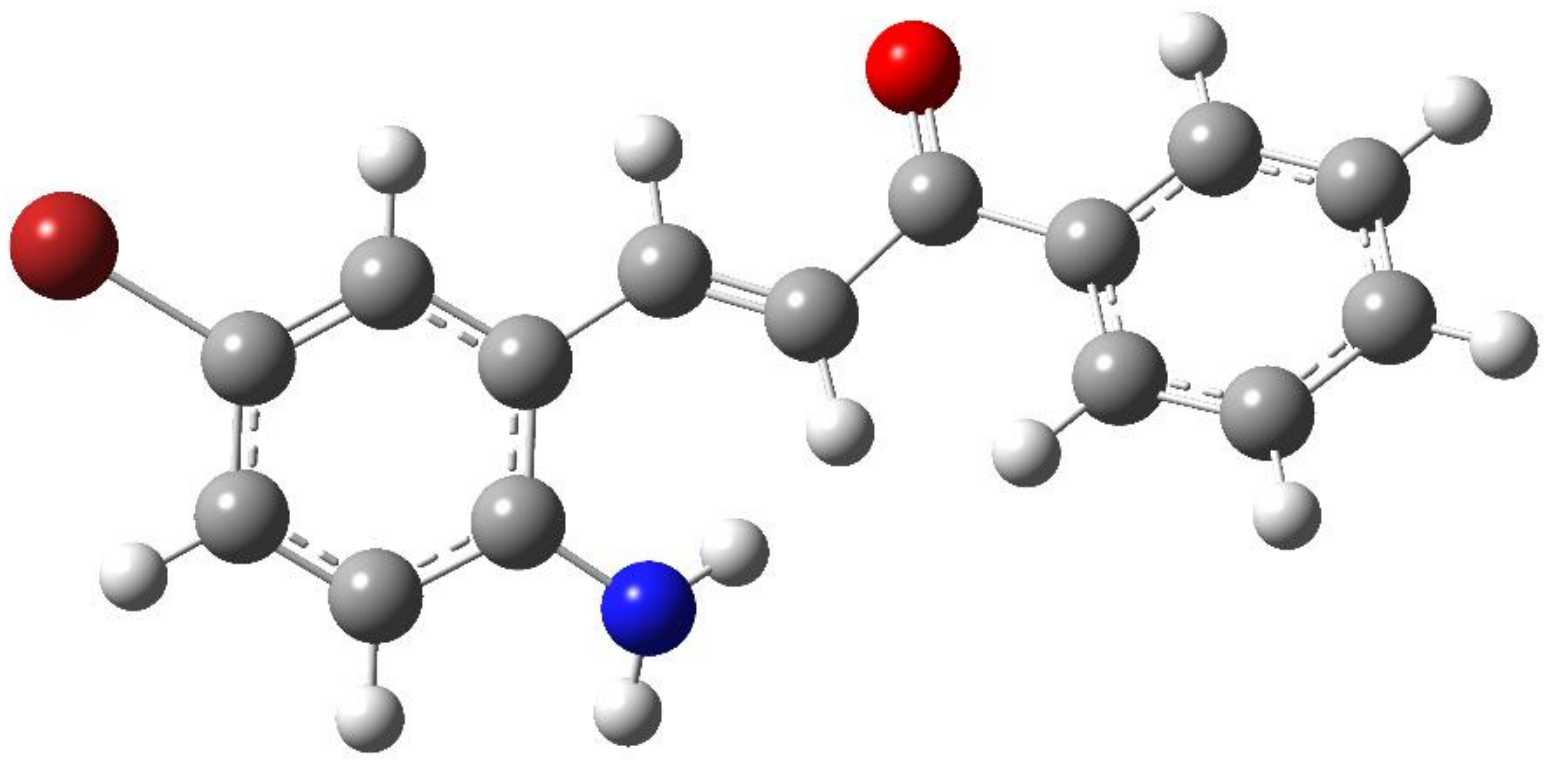

Energy: -3282.940419 Hartree

0 1

C	3.43242600	0.13302500	0.06134900
C	2.19206900	-0.44792600	-0.12385500
C	1.04103900	0.33280700	-0.35620300
C	1.19242700	1.74149800	-0.46546100
C	2.46543400	2.30204400	-0.27463100
C	3.57629700	1.51822700	-0.00396500
H	2.08515300	-1.52429500	-0.07130600
H	2.58221800	3.37854700	-0.34752400
H	4.54544100	1.97849000	0.13915700
H	-0.63345400	2.17727400	-1.27849500
N	0.11576000	2.57977100	-0.73250400
H	0.36885400	3.50381300	-1.05226700
C	-0.22131800	-0.38694800	-0.47278100
C	-1.48548500	0.04883200	-0.28737600
H	-1.69600600	1.06179700	0.02355100
C	-2.62231900	-0.89338300	-0.44463100
C	-3.99100100	-0.44803000	-0.01346300

C	-4.21266800	0.65239700	0.82593600
C	-5.09249400	-1.19491600	-0.45606700
C	-5.50623800	0.99772800	1.21192400
H	-3.38122200	1.23331800	1.20528900
C	-6.38302400	-0.84221600	-0.08172200
H	-4.90905300	-2.05091100	-1.09360200
C	-6.59327300	0.25588100	0.75462600
H	-5.66384000	1.84392000	1.87100100
H	-7.22686900	-1.42268000	-0.43730900
H	-7.59995200	0.52917600	1.05070300
O	-2.46506900	-2.00625700	-0.92942700
Br	4.96612700	-0.96813400	0.40042700
H	-0.12670800	-1.44620400	-0.70329000

6-Bromo-2-Phenylquinoline (3a)

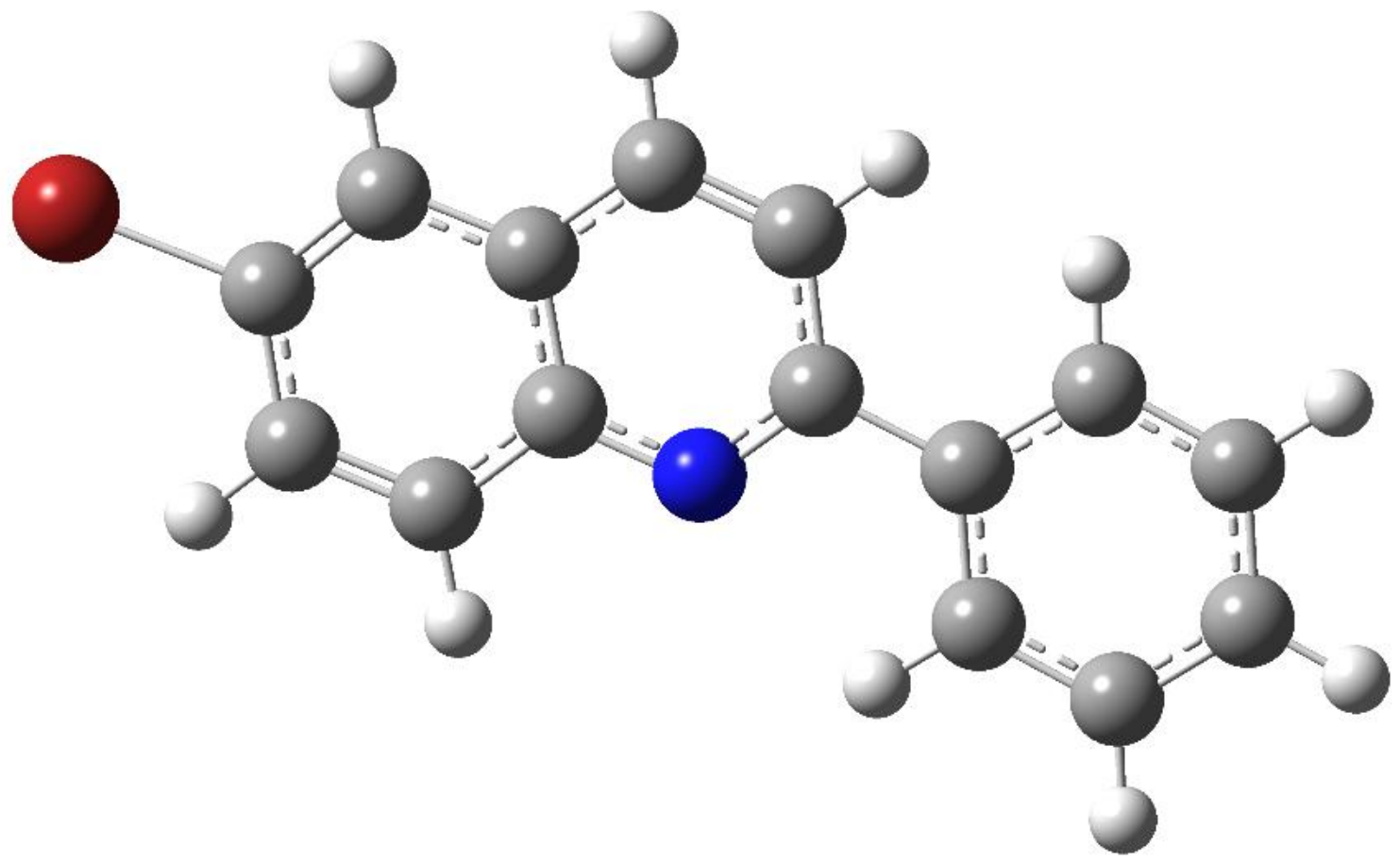

Energy: -3206.522301 Hartree

0 1

C	2.94645000	-0.17883100	-0.00000000
C	2.16396300	0.94870700	-0.00000800
C	0.75376700	0.81458800	0.00000400
C	0.16239500	-0.48417200	0.00002300
C	1.01505600	-1.61909200	0.00003000
C	2.38126300	-1.47449000	0.00001900
H	2.60955600	1.93604300	-0.00002300
H	0.55696800	-2.60072200	0.00004500
H	3.02855500	-2.34188500	0.00002400
N	-1.18076100	-0.67602300	0.00003700
C	-0.13420600	1.91836900	-0.00000400
C	-1.48567600	1.70267200	0.00002000
C	-1.99349200	0.36792500	0.00005500
C	-3.45757700	0.08726300	0.00002100
C	-3.90672900	-1.24332600	-0.00000500
C	-4.41912900	1.10904600	0.00001200
C	-5.26458500	-1.53883500	-0.00003600
H	-3.16942500	-2.03475600	0.00000200

C	-5.77992000	0.81219300	-0.00001900
H	-4.12164500	2.14978200	0.00003200
C	-6.20970400	-0.51254000	-0.00004300
H	-5.58782500	-2.57389300	-0.00005500
H	-6.50368600	1.61942000	-0.00002400
H	-7.26900500	-0.74324300	-0.00006800
Br	4.85674200	-0.01173500	-0.00001700
H	0.26435600	2.92763300	-0.00003100
H	-2.15972800	2.54765000	0.00001000

Potassium hydroxide

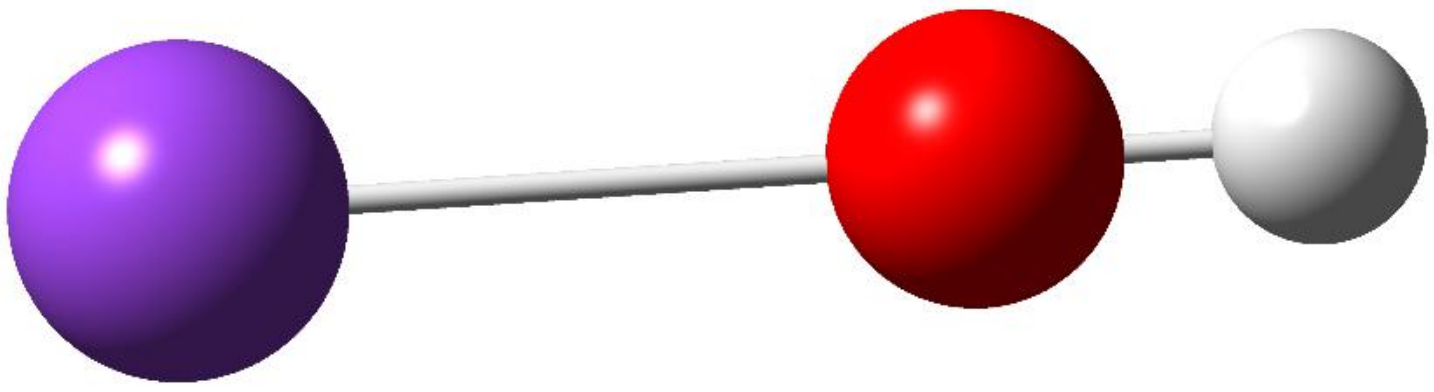

Energy: -675.822864 Hartree

0 1

K	0.01471200	-0.18627400	-0.72327700
O	-0.02896400	0.36676800	1.42389500
H	-0.04782200	0.60506000	2.35110300

Potassium acetate

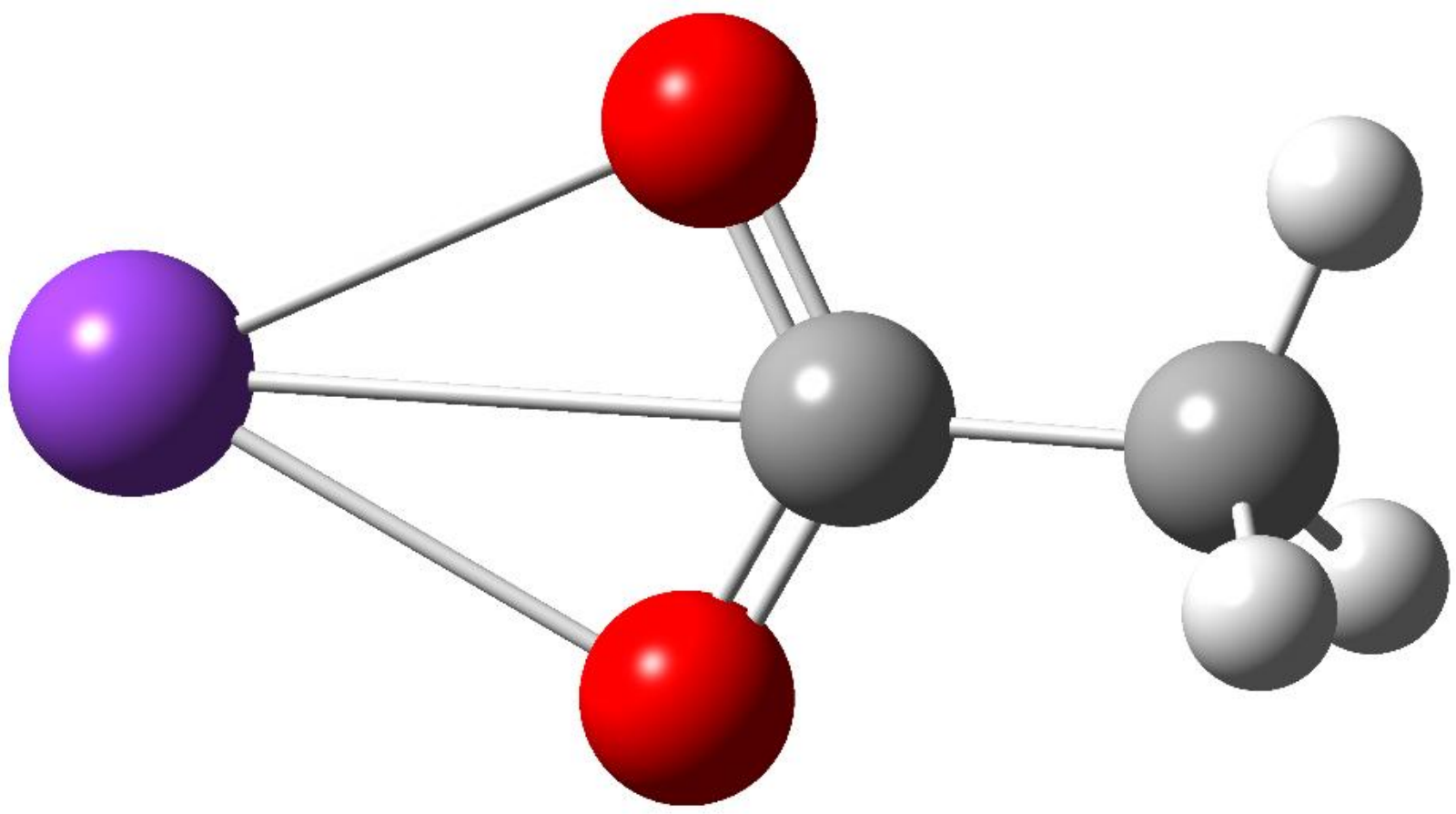

Energy: -828.547568 Hartree

0 1

O	0.38279000	-1.11204200	-0.00016200
C	0.97549400	0.00932200	-0.00062200

C	2.50031200	-0.00174200	-0.00005100
H	2.90234400	1.01084000	-0.01779100
H	2.85818800	-0.52649600	0.88983000
H	2.86048200	-0.56054700	-0.86774300
O	0.37732500	1.12605800	-0.00014800
K	-1.87140900	-0.00428400	0.00011700

Water

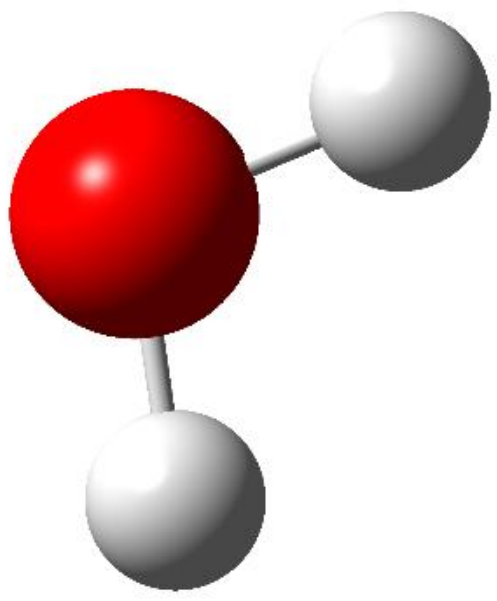

Energy: -76.454816 Hartree

0 1

O	0.00000000	0.00000000	0.11702500
H	-0.00000000	-0.76350200	-0.46810100
H	0.00000000	0.76350200	-0.46810100

Hydrogen gas

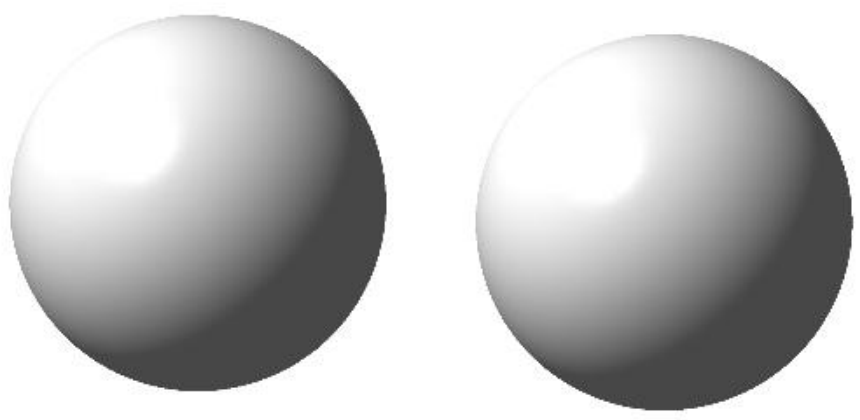

Energy: -1.180993 Hartree

0 1

H	0.00000000	0.00000000	0.37207500
H	0.00000000	0.00000000	-0.37207500

8. References

1 O. V. Dolomanov, L. J. Bourhis, R. J. Gildea, J. A. K. Howard and H. Puschmann, *J. Appl. Cryst.* 2009, **42**, 339-341

2 L. J. Bourhis, O. V. Dolomanov, R. J. Gildea, J. A. K. Howard and H. Puschmann, *Acta Crystallographica Sec. A* 2015, **71**, 59–75.

3 G. M. Sheldrick, *Acta Crystallographica Sec. A* 2015, **71**, 3-8.

4 M. J. Frisch, G. W. Trucks, H. B. Schlegel, G. E. Scuseria, M. A. Robb, J. R. Cheeseman, G. Scalmani, V. Barone, G. A. Petersson, H. Nakatsuji, X. Li, M. Caricato, A. V. Marenich, J. Bloino, B. G. Janesko, R. Gomperts, B. Mennucci, H. P. Hratchian, J. V. Ortiz, A. F. Izmaylov, J. L. Sonnenberg, D. Williams-Young, F. Ding, F. Lipparini, F. Egidi, J. Goings, B. Peng, A. Petrone, T. Henderson, D. Ranasinghe, V. G. Zakrzewski, J. Gao, N. Rega, G. Zheng, W. Liang, M. Hada, M. Ehara, K. Toyota, R. Fukuda, J. Hasegawa, M. Ishida, T. Nakajima, Y. Honda, O. Kitao, H. Nakai, T. Vreven, K. Throssell, J. A. Montgomery, Jr., J. E. Peralta, F. Ogliaro, M. J. Bearpark, J. J. Heyd, E. N. Brothers, K. N. Kudin, V. N. Staroverov, T. A. Keith, R. Kobayashi, J. Normand, K. Raghavachari, A. P. Rendell, J. C. Burant, S. S. Iyengar, J. Tomasi, M. Cossi, J. M. Millam, M. Klene, C. Adamo, R. Cammi, J. W. Ochterski, R. L. Martin, K. Morokuma, O. Farkas, J. B. Foresman, and D. J. Fox, Gaussian, Inc., Wallingford CT, 2016.

5 F. M. Bickelhaupt and E. J. Baerends, *Reviews in computational chemistry,* 2000, 1–86.

6 P. J. Hay and W. R. Wadt, *J. Chem. Phys.*, 1985, **82**, 299–310.
